# Stable and easily available sulfide surrogates allow a stereoselective activation of alcohols†

**DOI:** 10.1039/d1sc01602d

**Published:** 2021-05-06

**Authors:** Jérémy Merad, Ján Matyašovský, Tobias Stopka, Bogdan R. Brutiu, Alexandre Pinto, Martina Drescher, Nuno Maulide

**Affiliations:** Department of Organic Chemistry, University of Vienna Währinger Straße 38 1090 Vienna Austria nuno.maulide@univie.ac.at http://maulide.univie.ac.at; Univ. Lyon, Université Claude Bernard Lyon 1, CNRS CPE Lyon, INSA Lyon, ICBMS, UMR 5246 Bât. Lederer 1 rue Victor Grignard 69622 Villeurbanne France

## Abstract

Isothiouronium salts are easily accessible and stable compounds. Herein, we report their use as versatile deoxasulfenylating agents enabling a stereoselective, thiol-free protocol for synthesis of thioethers from alcohols. The method is simple, scalable and tolerates a broad range of functional groups otherwise incompatible with other methods. Late-stage modification of several pharmaceuticals provides access to multiple analogues of biologically relevant molecules. Performed experiments give insight into the reaction mechanism.

## Abstract 2

A simple and scalable method for stereoselective synthesis of thioethers directly from alcohols using isothiouronium salts is presented. The utility of this thiol-free reaction was exemplified by late-stage modification of complex molecules.

## Graphical abstract

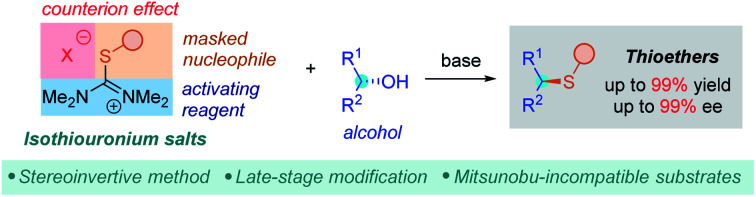

## Introduction

Sulfur is the 5th most abundant element (after C, H, O and N) in pharmaceuticals and agrochemicals. Indeed, more than 20% of recently approved drugs contain at least one sulfur atom.^1^ This widespread occurrence is likely due to this element's oxidation-state versatility, from thioethers and sulfonium salts to sulfoxides, sulfones and their derivatives.

**Scheme 1:**
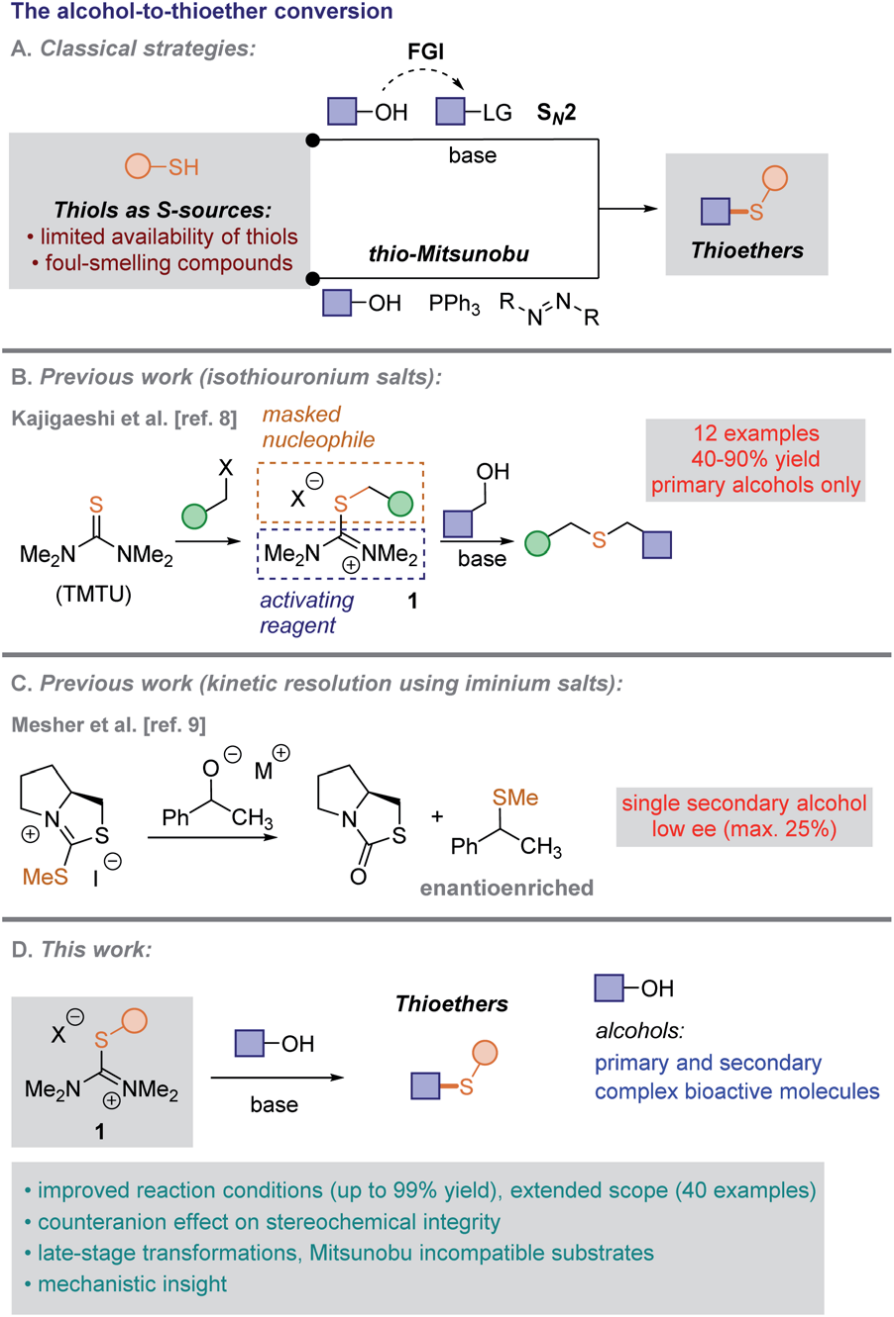
Synthetic strategies to access thioethers from alcohols.

In addition to being constitutive of multiple bioactive molecules,^1^ thioethers (or sulfides) readily participate in substitution and cross-coupling reactions.^2^ Thus, synthetic approaches allowing the incorporation of divalent sulfur have been vigorously investigated during past decades.^3^ A traditional S_N_2-type approach is still the method of choice for most synthetic applications. In fact, catalytic nucleophilic substitutions have recently emerged as alternatives to traditional approaches,^4^ relying on *in situ* activation of (secondary) alcohols, ultimately leading to amines, esters, azides or halides with high selectivities.^5^ Classical strategies, however, require prior functional group interconversion (FGI) when alcohols and thiols are considered as the reaction substrates, effectively employing two linear steps to achieve this transformation (Scheme 1A). Reactions of chiral alcohols in such a way generally provide the desired thioethers with clean inversion of configuration (unless S_N_1-type processes operate, such as with benzylic, allylic or tertiary alcohols under acid catalysis).^6^ Alternatively, the stepwise conversion of alcohols into thioethers *via* halides proceeds with retention of configuration due to twofold inversion. Thio-Mitsunobu reaction efficiently converts alcohols into thioethers, while requiring a combination of two *stoichiometric* activating agents (Scheme 1A). Again the reaction generally proceeds with stereochemical inversion of configuration for secondary alcohols.^7^ However, aliphatic thiols are mostly considered unsuitable for the Mitsunobu reaction, as the thiol group itself is not acidic enough for the reaction to take place.^7*b*^ Additionally, there are noteworthy functional group tolerance issues in Mitsunobu-type protocols (*e.g.* azides or peroxides are incompatible with the phosphine reactants typical of Mitsunobu procedures). Finally, both of these methods employ practically unsuitable thiols as the nucleophilic components. Indeed, more than being notoriously malodorant compounds and susceptible to oxidation, very few thiols are commercially available, severely limiting the diversity of readily accessible thioether derivatives.

Isothiouronium salts are odorless, stable, easily accessible compounds, which represent potentially interesting activators for the hydroxyl group. Indeed, Kajigaeshi published a general one-pot two-step convergent strategy for preparation of thioethers from primary alcohols utilizing isothiouronium salts (Scheme 1B).^8^ This work showcases 12 examples with moderate yields, but establishes precedence for using thiouronium salts. Proline-derived cyclic iminium salts were used in a similar work in an attempt to kinetically resolve secondary benzylic alcohols with mediocre results (Scheme 1C).^9^ Related iminium salts were used for preparation of alkyl(tetrazolyl)sulfides using an exogenous aryl thiol or for deoxygenative iodination of alcohols, with notorious erosion of stereochemical purity of enantiopure starting material.^10^ Analogous reagents have also found limited applications in the syntheses of 2-pyridinethioesters,^11*a*^ Barton's esters,^11*b*^*N*-thioalkenyl and *N*-(*o*-thio)aryl-benzimidazol-2-ones.^11*c*^ Thioimidazolinium salts have been used extensively for the preparation of sulfides or as cyanation agents.^12^ Hopkinson recently demonstrated that benzothiazolium reagents can be used in trifluoromethyl(alkyl) sulfide synthesis or fluorinated thioester synthesis.^13^ However, to the best of our knowledge, a successful chiral thioether synthesis using isothiouronium salts involving a configuration inversion of enantiopure secondary alcohols has not been reported. Additionally, we believe that the straightforward reactivity of uronium salts could prove advantageous in a range of synthetic contexts ranging from late-stage modification to enabling preparation of thioethers carrying sensitive functional groups. Herein, we report our findings in all of the above-mentioned endeavours (Scheme 1D).

## Results and discussion

**Scheme 2:**
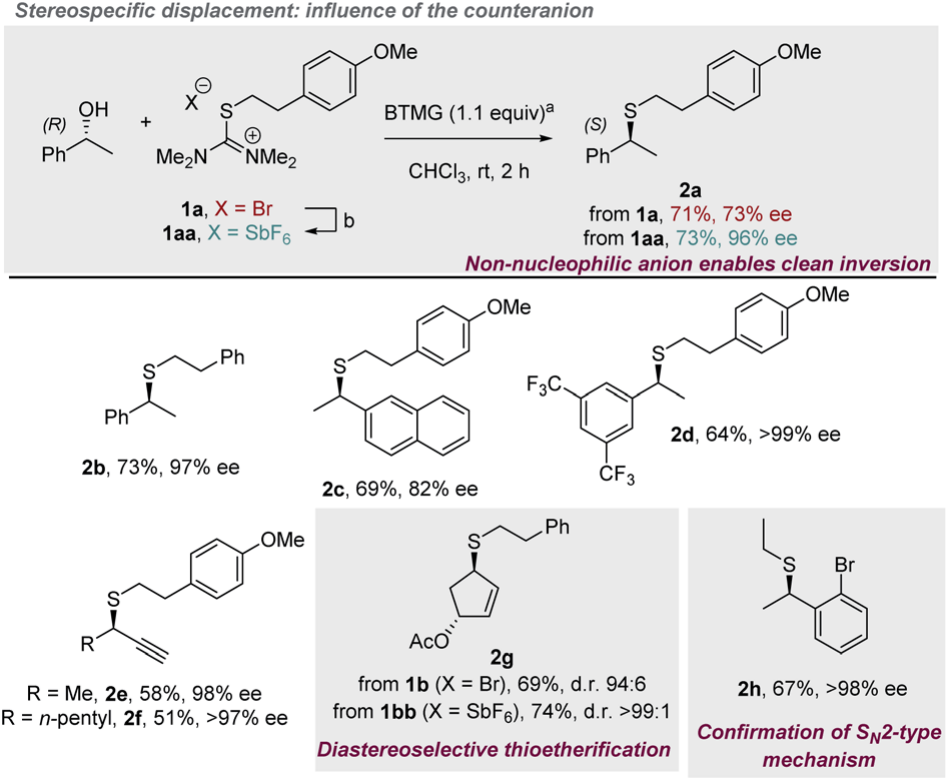
Thioetherification of optically active secondary alcohols: impact of the counteranion. ^a^Reactions were performed at rt for 2 h with alcohol (0.30 mmol, 1.0 equiv.), isothiouronium salt (0.30 mmol, 1.0 equiv.) and BTMG (0.33 mmol, 1.1 equiv.) in CHCl_3_ (0.14 M concentration). ^b^Reaction conditions: AgSbF_6_, H_2_O (0.35 M), rt, 30 min, 84% yield. Yields refer to isolated products. BTMG = 2-*tert*-butyl-1,1,3,3-tetramethylguanidine.

Taking Kajigaeshi's work^8^ into consideration, it is reasonable to assume that the thioetherification of most primary alcohols involves an S_N_2-type mechanism. However, it remained unclear how such a reaction manifold would hold for more hindered substrates. In particular, the possibility of S_N_1/S_N_2 mechanistic competition and its impact on stereocontrol appeared to be potential pitfalls, especially for secondary benzylic/allylic alcohols. After a survey of reaction conditions,^14^ deoxasulfenylation using the thiouronium bromide **1a** was performed on enantiopure (>99% ee) (*R*)-1-phenylethan-1-ol (Scheme 2) in order to discriminate between these two possible mechanisms. Thioether **2a** was obtained in 71% yield and a moderate 73% ee. While it was tempting to interpret this result as a consequence of simultaneous operation of S_N_2 and S_N_1-type pathways, we also wondered if the counterion **X−** of the isothiouronium reactant **1a** might play a non-innocent role.^9^ Indeed, bromide is a competent nucleophile while also serving as a good leaving group, an ability best expressed in Finkelstein and related reactions.^15^ Therefore, exchanging the bromide counteranion of isothiouronium **1a** for the less nucleophilic hexafluoroantimonate using AgSbF_6_ in water gave the salt **1aa** in 84% yield. Repeating the deoxysulfenylation reaction with **1aa** resulted in excellent inversion of configuration (96% ee), while the yield of the reaction remained unchanged. Additionally, the stereochemical outcome of the reaction was the same utilizing either tetrafluoroborate (**1ab**, 96% ee) or hexafluorophosphate thiouronium salts (**1ac**, 97% ee), while the overall reaction yields dropped slightly.^14^ Similarly excellent results were observed when related isothiouronium antimonate **1bb** was employed (product **2b**, 73% yield, 97% ee). Other chiral secondary alcohols were then investigated. A slight drop in ee was observed for naphtyl-bearing substrate **2c** (82% ee), while the presence of electron-withdrawing groups led to a product with >99% ee (**2d**). Excellent results were also obtained with propargylic substrates (**2e** and **2f**, >97% ee). A successful diastereoselective thioetherification of an allylic substrate **2g** provided the desired thioether as a single diastereomer when isothiouronium **1bb** was used (compared to increased amounts of a second diastereomer, when salt **1b** was used). Finally, to unambiguously verify S_N_2-type inversion of configuration, thioether **2h** was prepared in 67% yield and >98% ee. The optical rotation was in good agreement with the value reported in the literature ([*α*]^20^_D_ = −57.4 (*c* = 1, CHCl_3_); lit. [*α*]^20^_D_ = −54.7 (*c* = 1, CHCl_3_)).^16,17^

**Scheme 3:**
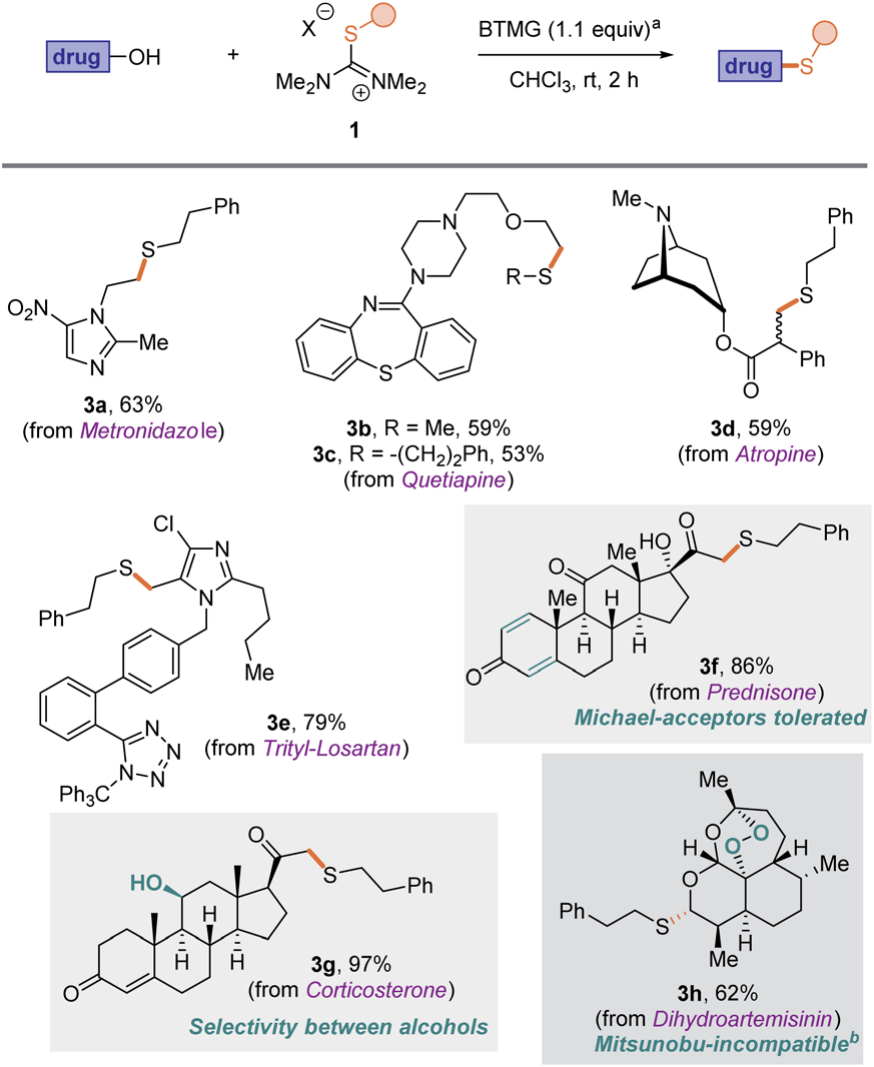
Late-stage modification of various drugs. ^a^Reactions were performed at rt for 2 h with the corresponding drug (0.30 mmol, 1.0 equiv.), isothiouronium salt (0.30 mmol, 1.0 equiv.) and BTMG (0.33 mmol, 1.1 equiv.) in CHCl_3_ (0.14 M concentration). Yields refer to isolated products. ^b^Thio-Mitsunobu conditions: dihydroartemisinin (0.2 mmol, 1.0 equiv.), thiol (0.2 mmol, 1.0 equiv.), PPh_3_ (0.3 mmol, 1.5 equiv.), diethyl azodicarboxylate (0.3 mmol, 1.5 equiv.) in THF (at 0.1 M concentration). Yield 0%.

The synthetic potential of isothiouronium salts in deoxasulfenylation reactions was then investigated in late-stage derivatisation (Scheme 3). Indeed, several clinically used drugs carry free hydroxyl groups and we were eager to probe these salts in such challenging, functional-group-rich contexts.

As shown, several nitrogen-containing bioactive compounds such as metronidazole (antibiotic), quetiapine (antipsychotic), atropine (antispasmodic) and trityl-losartan (antihypertensive) were smoothly converted to their corresponding thioethers (**3a–e**). The modification of prednisone (**3f**) and corticosterone (**3g**) proved to be perfectly chemoselective despite the presence of reactive Michael acceptors (enones) and multiple hydroxyl groups: the result with corticosterone (**3g**) additionally suggests useful chemoselectivity between primary and secondary alcohols. The derivatization of dihydroartemisinin (**3h**) is particularly relevant, since standard thio-Mitsunobu conditions led only to decomposition of this redox-sensitive substrate possessing cyclic peroxide. Notably, it has been shown that C-10 thioethers derived from artemisinine display significant anticancer activity.^18^

**Scheme 4:**
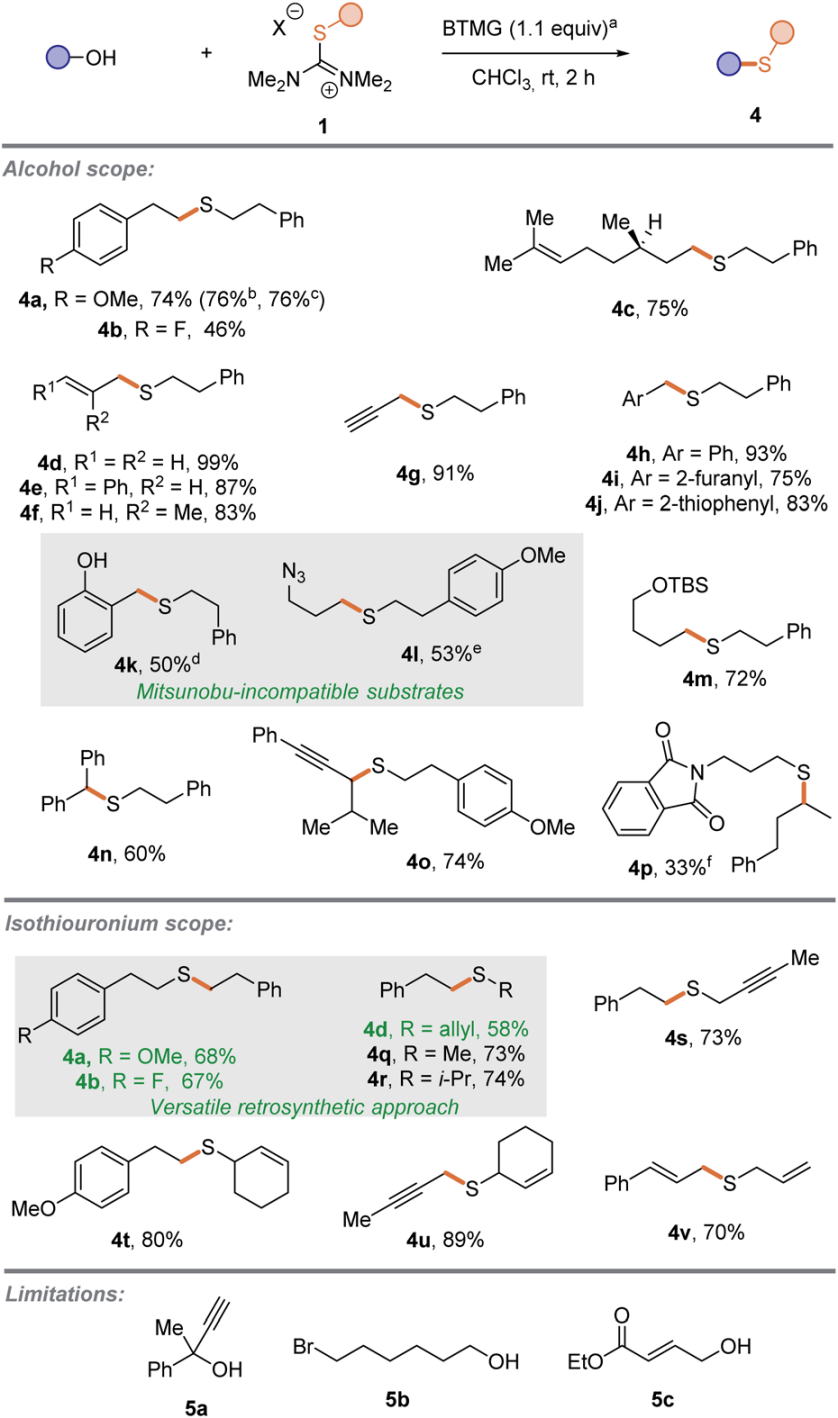
Thioetherification scope. ^a^Reactions were performed at rt for 2 h with alcohol (0.30 mmol, 1.0 equiv.), isothiouronium salt (0.30 mmol, 1.0 equiv.) and BTMG (0.33 mmol, 1.1 equiv.) in CHCl_3_ (0.14 M concentration). Yields refer to isolated products. ^b^Open-flask conditions. ^c^Gram scale. ^d^BTMG = 2.2 equiv. ^e^Thio-Mitsunobu conditions: azidoalcohol (0.2 mmol, 1.0 equiv.), thiol (0.2 mmol, 1.0 equiv.), PPh_3_ (0.3 mmol, 1.5 equiv.), diethyl azodicarboxylate (0.3 mmol, 1.5 equiv.) in THF (at 0.1 M concentration). Yield 0%. ^f^Reaction time 14 h (at 1 M concentration).

Finally, we wanted to extend the portfolio of substrates to other primary and secondary alcohols (Scheme 4).^8^ Thioetherification of diversely substituted alcohols with salt **1b** efficiently delivered products **4a** and **4b**. In order to probe robustness and reproducibility, the synthesis of thioether **4a** was also performed with technical solvents, under open-flask conditions as well as on a gram scale without any erosion of the yield. β-Citronellol led to the desired thioether **4c** in 75% yield. Deoxasulfenylation of several allylic (**4d–f**), propargylic (**4g**) and α-(heteroaryl) alcohols (**4h–j**) occurred smoothly with perfect regioselectivity (no products resulting from S_N_2′-type reaction were observed). Pleasingly, the transformation remains efficient in the presence of phenols (**4k**) and azides (**4l**). Such substrates are indeed challenging in classical Mitsunobu reactions, due to the nucleophilicity of phenoxide anion and the fast reaction between azides and phosphines. Preparation of **4l** was nevertheless attempted under thio-Mitsunobu conditions, showing no product being formed. Moreover, a silylether is also tolerated (**4m**). The methodology was successfully extended to secondary alcohols to afford the corresponding thioethers **4n** and **4o** in satisfying yields. Aliphatic secondary alcohols did not react as efficiently, providing the desired sulfide in 33% yield using slightly modified reaction conditions (**4p**). We next turned our attention to the structure of the transferable S-containing residues (Scheme 4). This switch led us to prepare thioethers **4a**, **4b** and **4d** by forging the opposite C–S bond. This demonstrates the versatility of this approach, enabling access to the same product by either of two disconnections depending on the accessibility of the starting materials. Thioethers incorporating methyl (**4q**), isopropyl (**4r**), and propargyl (**4s**) groups were prepared in good yields. Also, several allylic thiols were transferred to access polyunsaturated compounds **4t–v**. However, no conversion was observed with tertiary alcohols (**5a**), likely due to high steric hindrance. Additionally, the reaction did not result in desired products when either hydroxyalkyl halide (**5b**) or crotonate derived alcohol (**5c**) were employed as substrates.^19^

**Scheme 5:**
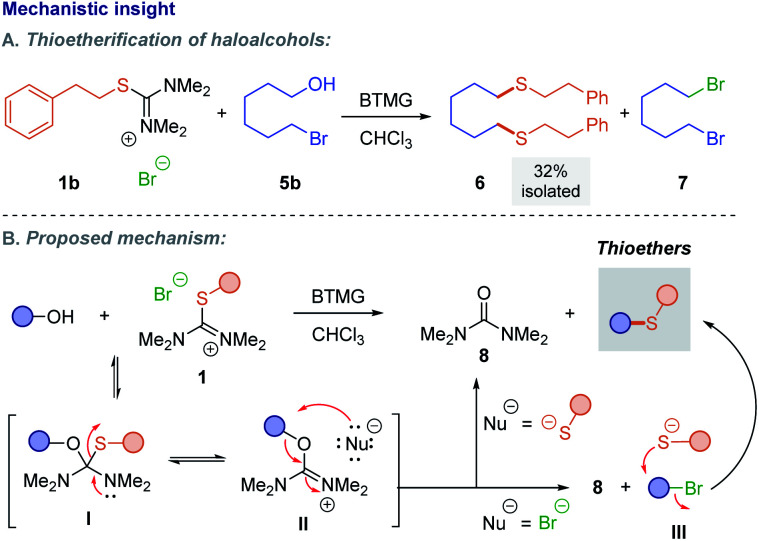
Mechanistic insight into thioetherification of alcohols using isothiouronium salts.

The result of the reaction of alcohol **5b**, namely the formation of thioether **6** and dibromide **7** (*cf.*Scheme 5) resulting from two consecutive substitutions, prompted us to reevaluate the reaction mechanism. The same result was obtained even when SbF_6_^−^ isothiouronium salt **1bb** was used in the reaction, suggesting that the thiol/thiolate liberated during the reaction can react either at the carbon carrying the bromide or the isouronium moiety (Scheme 5A).^20^ Based on these findings, we propose a modified reaction mechanism. Addition of alcohol onto **1** provides hemithioacetal **I**, in equilibrium with ion pair **II** (Nu = SR). At this stage, any halide anion present can act as a competing nucleophile, causing formation of intermediate **III**. This effect is eliminated when the SbF_6_^−^ salt is used. Finally, irreversible nucleophilic displacement of the isouronium in **II** (leading to urea derivative **8**) or the halide in **III** provides the desired thioethers (Scheme 5B). The double substitution products of Scheme 5A appear to be the consequence of bromide displacement by thiolate competing with S_N_2-collapse of ion pair **II**.

## Conclusions

In summary, we report that easily preparable isothiouronium salts serve as convenient deoxasulfenylating reagents for alcohols. Stereoinversion of chiral secondary alcohols was shown to be counteranion dependent, leading to clean inversion of configuration. Late-stage modification of several biologically relevant substances is enabled by this procedure. This method offers straightforward access to thioethers and constitutes a valuable alternative to the established methods. This is particularly visible on substrates otherwise incompatible with thio-Mitsunobu reaction protocols. Finally, our findings provided mechanistic insight into the reaction. We believe that these reactions of isothiouronium salts might be of interest as rapid and mild methods for conjugation.

## Author contributions

The work was conceptualized by J. Merad and N. M. The first draft of the manuscript was written by J. Merad and J. Matyašovský, all of the authors contributed to the final version of the manuscript. The experiments were performed by J. Merad, J. Matyašovský, T. S., B. R. B., A. P. and M. D. N. M. was involved in securing funding, manuscript editing and finalizing and overall supervision of the project.

## Conflicts of interest

There are no conflicts to declare.

## Supplementary Material

SC-012-D1SC01602D-s001
