# Supplementary material for: Stable and easily available sulfide surrogates allow a stereoselective activation of alcohols
Source: Chem Sci. 2021 May 6;12(22):7770–4. doi: 10.1039/d1sc01602d (PMC8188487; doi:10.1039/d1sc01602d)

Electronic Supplementary Material (ESI) for Chemical Science.
This journal is © The Royal Society of Chemistry 2021

Supporting Information

for

Stable and Easily Available Sulfide Surrogates Allow a Stereoselective Activation of Alcohols

Jérémy Merad[+,a,b], Ján Matyašovský[+,a], Tobias Stopka[+,a], Bogdan R. Brutiu[⁑,a], Alexandre Pinto[⁑,a], Martina Drescher[a] and Nuno Maulide[*,a]

Abstract: Isothiouronium salts are easily accessible and stable compounds. Herein, we report their use as versatile deoxasulfenylating agents enabling a stereoselective, thiol-free protocol for synthesis of thioethers from alcohols. The method is simple, scalable and tolerates a broad range of functional groups otherwise incompatible with other methods. Late-stage modification of several pharmaceuticals provides access to multiple analogues of biologically relevant molecules. Performed experiments give insight into the reaction mechanism.

[a] Dr. J. Merad[*], Dr. J. Matyašovský[*], Dr. T. Stopka[*], B. R. Brutiu[⁑], Dr. A. Pinto[⁑], M. Drescher, Prof. Dr. N. Maulide
Department of Organic Chemistry, University of Vienna
Währinger Straße 38, 1090 Vienna (Austria)
E-Mail: nuno.maulide@univie.ac.at
Homepage: http://maulide.univie.ac.at

[b] Dr. J. Merad
Université Lyon I, CNRS, INSA
1 Rue Victor Grignard, 69622 Villeurbanne cedex (France)

[*] These authors contributed equally to this manuscript.
[⁑] These authors contributed equally to this manuscript.

Table of Contents

Table of Contents 2
I. General information 3
II. Optimization of reaction conditions 4
III. Isothiouronium salts synthesis 5
IV. Stereospecific thioetherification of optically active alcohols 8
V. Late-stage transformation of biologically relevant molecules 20
VI. Thioetherification of primary and secondary alcohols 24
VII. References 31
VIII. NMR spectra 32

I. General information

Unless otherwise stated, all glassware was flame-dried before use and all reactions were performed under an atmosphere of argon. All solvents were distilled from appropriate drying agents prior to use or directly taken from commercial sealed bottles under an atmosphere of argon. All reagents and some substrates (alcohols, carboxylic acids, amines and alkyl halides) were used as received from commercial suppliers unless otherwise stated. Reaction progress was monitored by thin layer chromatography (TLC) performed on aluminum plates coated with silica gel F254 with 0.2 mm thickness. Chromatograms were visualized by fluorescence quenching with UV light at 254 nm or by staining using potassium permanganate. Flash column chromatography was performed using silica gel 60 (230-400 mesh, Merck and co.). Neat infra-red spectra were recorded using a Brucker Vertex 70 FT-IR spectrometer. Wavenumbers are reported in cm^{-1}. Mass spectra were obtained using a Brucker maXis UHR-TOF spectrometer, using electrospray ionization (ESI) and by Agilent 7200B GC/Q-TOF spectrometer, using electron impact (EI). All ^{1}H NMR, ^{13}C NMR and ^{19}F NMR spectra were recorded using a Bruker AV III 400, AV NEO 500, AV III 600 or AV III HD 700 spectrometer in $CDCl_3$. Chemical shifts were given in parts per million (ppm, δ), referenced to the solvent peak of $CDCl_3$, defined at δ = 7.26 ppm (^{1}H NMR) and δ = 77.16 (^{13}C NMR). Coupling constants are quoted in Hz (J). ^{1}H NMR splitting patterns were designated as singlet (s), doublet (d), triplet (t), quartet (q), pentet (p). Splitting patterns that could not be interpreted or easily visualized were designated as multiplet (m), apparent (app) or broad (br). Enantiomeric excess was measured on a Shimadzu LC-8A preparative HPLC system using Lux Cellulose-1 or Lux Cellulose-3 chiral columns.

II. Optimization of reaction conditions

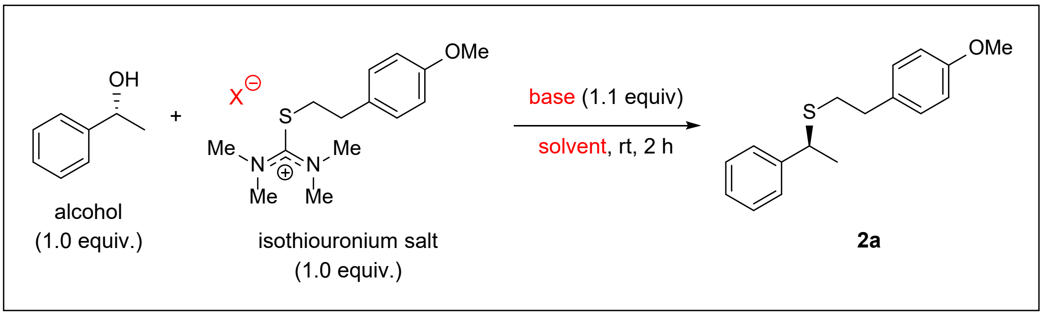

A base (0.33 mmol, 1.1 equiv.) was added dropwise to a solution of (R)-1-phenylethan-1-ol (37 mg, 0.30 mmol, 1.0 equiv., >99% ee) and the corresponding isothiouronium salt (0.30 mmol, 1.0 equiv.) in anhydrous solvent (2.2 mL) under an Ar atmosphere. The reaction mixture was then stirred at rt for 2 h before excess base was quenched by addition of a saturated solution of NH_4Cl. The mixture was then extracted with CH_2Cl_2 (3x), the combined organic phases were dried over $MgSO_4$, filtered and concentrated under reduced pressure. Purification on silica gel (heptane/Et_2O = 24:1). Enantiomeric excess was determined using chiral HPLC.

Table 1: Optimization of reaction conditions

Entry	X^-	Base	Solvent	Yield	ee
1	Br^-	BTMG[a]	$CHCl_3$	71%	73%
2	SbF_6^-	BTMG	$CHCl_3$	73%	96%
3	SbF_6^-	BTMG	Toluene	0%	n.d.[b]
4	SbF_6^-	BTMG	THF	20%[c]	n.d.
5	SbF_6^-	BTMG	CH_3CN	38%	86%
6	SbF_6^-	BTMG	DMF	52%	91%
7	SbF_6^-	NEt_3	$CHCl_3$	0%	n.d.
8	SbF_6^-	DBU[d]	$CHCl_3$	23%[c]	n.d.
9	SbF_6^-	2,6-lutidine	$CHCl_3$	0%	n.d.
10	SbF_6^-	imidazole	$CHCl_3$	0%	n.d.
11	SbF_6^-	TMG[e]	$CHCl_3$	28%[c]	n.d.
12	BF_4^-	BTMG	$CHCl_3$	53%	96%
13	PF_6^-	BTMG	$CHCl_3$	58%	97%

[a] 2-*tert*-Butyl-1,1,3,3-tetramethylguanidine, Barton's base; [b] not determined; [c] NMR yield based on crude material, determined using trimethoxybenzene as an internal standard; [d] 1,8-Diazabicyclo[5.4.0]undec-7-en; [e] 1,1,3,3-Tetramethyguanidine.

III. Isothiouronium salts synthesis

a. General procedure A : synthesis of isothiouronium salts

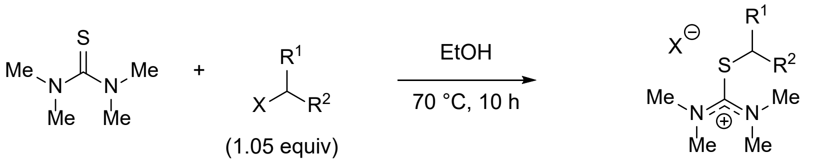

1

The procedure was adapted from the literature with changes.[1] Alkyl halide (1.05-5.0 equiv.) was added onto a solution of *N*,*N*,*N*,*N*-tetramethylthiourea (TMTU) (1.0 equiv.) in the EtOH [1 M]. The mixture was then heated at 70 ˚C for 10 h. After cooling to room temperature, the volatiles were evaporated on a rotavap followed by further drying under high vacuum to give the desired salt with acceptable purity.

b. Isothiouronium salts characterization

2-(4-methoxyphenethyl)-1,1,3,3-tetramethylisothiouronium bromide (1a)

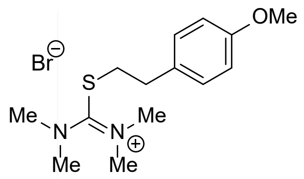

1a

Prepared according to general procedure **A** from TMTU (132 mg, 1.0 mmol, 1.0 equiv.) and 1-(2-bromoethyl)-4-methoxybenzene (226 mg, 1.05 mmol, 1.05 equiv.). White solid (340 mg, 0.98 mmol, 98% yield).
^{1}H-NMR (400 MHz, $CDCl_3$): δ 7.22 – 7.16 (m, 2H), 6.90 – 6.83 (m, 2H), 3.78 (s, 3H), 3.45 (t, *J* = 7.1 Hz, 2H), 3.39 (s, 12H), 3.00 (t, *J* = 7.1 Hz, 2H).; **^{13}C-NMR** (101 MHz, $CDCl_3$): δ 176.1, 158.9, 130.1, 129.9, 114.4, 55.5, 44.9, 37.2, 34.6; **IR** (neat): ν 1595, 1511, 1393, 1246, 1178, 1111, 1032, 726, 696 cm^{-1}; **HRMS** (ESI^+, m/z): [M^+] calculated for [$C_{14}H_{23}N_2OS^+$]: 267.1526, found: 267.1522. Mp.: 105–107 °C.

1,1,3,3-tetramethyl-2-phenethylisothiouronium bromide (1b)

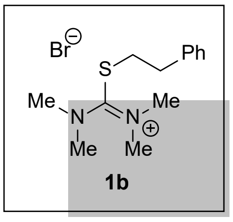

1b

Prepared according to general procedure **A** from TMTU (661 mg, 5.0 mmol, 1.0 equiv.) and (2-bromoethyl)benzene (0.75 mL, 5.5 mmol, 1.1 equiv.). White solid (1.56 g, 4.9 mmol, 99% yield).
^{1}H-NMR (400 MHz, $CDCl_3$): δ 7.38 – 7.21 (m, 5H), 3.53 (t, *J* = 7.1 Hz, 2H), 3.40 (s, 12H), 3.10 (t, *J* = 7.1 Hz, 2H); **^{13}C-NMR** (101 MHz, $CDCl_3$): δ 175.9, 138.1, 129.0, 128.9, 127.3, 44.9, 36.9, 35.5; **IR** (neat) ν 1264, 750, 732, 701 cm^{-1}; **HRMS** (ESI^+, m/z): [M^+] calculated for [$C_{13}H_{21}N_2S^+$]: 237.1420, found: 237.1419. Mp.: 146–148 °C.

2-ethyl-1,1,3,3-tetramethylisothiouronium bromide (1c)

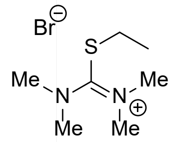

1c

Prepared according to general procedure **A** from TMTU (530 mg, 4.0 mmol, 1.0 equiv.) and ethyl bromide (1.50 mL, 20.0 mmol, 5.0 equiv.). White crystalline solid (950 mg, 3.9 mmol, 98% yield).
^{1}H NMR (600 MHz, $CDCl_3$) δ 3.41 (s, 12H), 3.12 (q, J = 7.5 Hz, 2H), 1.33 (t, J = 7.5 Hz, 3H); **^{13}C NMR** (151 MHz, $CDCl_3$) δ 175.5, 44.9, 29.8, 14.8; **IR** (neat) ν 2921, 2852,

1460, 1377, 761 cm^{-1}; **HRMS** (ESI$^+$, m/z): [M$^+$] calculated for [$C_7H_{17}N_2S^+$]: 161.1107, found: 161.1115. Mp.: 90–92 °C.

2-(4-fluorophenethyl)-1,1,3,3-tetramethylisothiouronium bromide (1d)

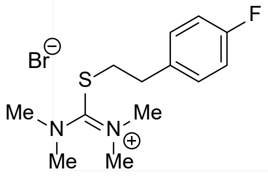

1d

Prepared according to general procedure **A** from TMTU (264 mg, 2.0 mmol, 1.0 equiv.) and 1-(2-bromoethyl)-4-fluorobenzene (447 mg, 2.2 mmol, 1.1 equiv.). White solid (658 mg, 2.0 mmol, 98% yield).

^{1}H-NMR (400 MHz, $CDCl_3$): δ 7.34 – 7.28 (m, 2H), 7.07 – 6.92 (m, 2H), 3.52 (t, J = 7.3 Hz, 2H), 3.39 (s, 12H), 3.06 (t, J = 7.3 Hz, 2H); **^{13}C-NMR** (101 MHz, $CDCl_3$): δ 176.1, 162.1 (d, J = 245.7 Hz), 134.0 (d, J = 3.2 Hz), 130.6 (d, J = 7.9 Hz), 115.8 (d, J = 21.3 Hz), 45.0, 37.1, 34.7; **IR** (neat): ν 1264, 729, 700 cm^{-1}; **HRMS** (ESI$^+$, m/z): [M$^+$] calculated for [$C_{13}H_{20}FN_2S^+$]: 255.1326, found: 255.1322. Mp.: 117–119 °C.

2-allyl-1,1,3,3-tetramethylisothiouronium bromide (1e)

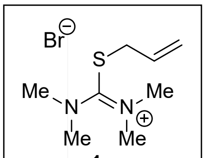

1e

Prepared according to a modified general procedure **A** from TMTU (400 mg, 3.0 mmol, 1.0 equiv.) and ally bromide (0.52 mL, 6.0 mmol, 2.0 equiv.). Slightly brown solid (758 mg, 3.0 mmol, quant. yield).

^{1}H NMR (400 MHz, $CDCl_3$) δ 5.88 (ddt, J = 17.0, 10.0, 7.2 Hz, 1H), 5.35 (dd, J = 17.0, 1.0 Hz, 1H), 5.24 (dd, J = 10.0, 1.0 Hz, 1H), 3.78 (d, J = 7.2 Hz, 2H), 3.45 (s, 12H); **^{13}C NMR** (101 MHz, $CDCl_3$) δ 174.8, 131.1, 121.1, 45.0, 38.0; **IR** (neat): ν 3503, 1602, 1398, 1262, 963, 884, 731 cm^{-1}; **HRMS** (ESI$^+$, m/z): [M$^+$] calculated for [$C_8H_{17}N_2S^+$]: 173.1107, found: 175.1108. Mp.: 143–145 °C.

1,1,2,3,3-pentamethylisothiouronium iodide (1f)

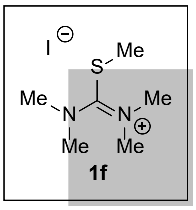

1f

Prepared according to a slightly modified general procedure **A** from TMTU (400 mg, 3.0 mmol, 1.0 equiv.) and iodomethane (0.93 mL, 3.0 mmol, 5.0 equiv.). Orange solid (566 mg, 2.1 mmol, 69% yield).

^{1}H NMR (400 MHz, $CDCl_3$) δ 3.43 (s, 12H), 2.71 (s, 3H); **^{13}C NMR** (101 MHz, $CDCl_3$) δ 177.1, 45.4, 18.6; **IR** (neat) ν 3444, 3001, 2923, 2852, 1595, 1509, 1465, 1395, 1321, 1255, 1206, 1171, 1114, 1062, 873, 726 cm^{-1}; **HRMS** (ESI$^+$, m/z): [M$^+$] calculated for [$C_6H_{15}N_2S^+$]: 147.0950, found: 147.0949. Mp.: 132–134 °C.

2-isopropyl-1,1,3,3-tetramethylisothiouronium bromide (1g)

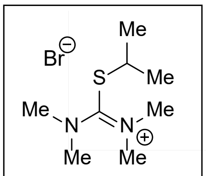

1g

Prepared according to a modified general procedure **A** from TMTU (400 mg, 3.0 mmol, 1.0 equiv.) and 3-bromopropane (1.4 mL, 15 mmol, 5.0 equiv.) in *tert*-amyl alcohol at 80 °C. After cooling at rt the solvent was evaporated under vacuum and the crude product was washed several times with EtOAc to give the desired salt as a white solid (275 mg, 1.1 mmol, 36% yield).

^{1}H NMR (400 MHz, $CDCl_3$) δ 3.67 – 3.56 (m, 1H), 3.48 (s, 12H), 1.36 (d, ^3J = 6.7 Hz, 6H); **^{13}C NMR** (101 MHz, $CDCl_3$) δ 174.2, 45.0, 41.1, 23.7; **IR** (neat) ν 3376, 2971, 2932, 1594, 1505, 1452, 1394, 1246, 1206, 1167, 1113, 1055 cm^{-1}; **HRMS** (ESI$^+$, m/z): [M$^+$] calculated for [$C_8H_{19}N_2S^+$]: 175.1263, found: 175.1263.

2-(but-2-yn-1-yl)-1,1,3,3-tetramethylisothiouronium bromide (1h)

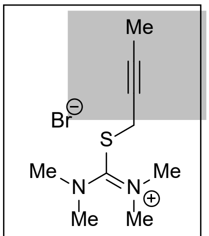

1h

Prepared according to general procedure **A** from TMTU (264 mg, 2.0 mmol, 1.0 equiv.) and 1-bromobut-2-yne (279 mg, 2.1 mmol, 1.05 equiv.). Yellow solid (528 mg, 2.0 mmol, quant. yield).

^{1}H-NMR (400 MHz, $CDCl_3$): δ 3.89 (q, *J* = 2.5 Hz, 2H), 3.49 (s, 12H), 1.85 (t, *J* = 2.5 Hz, 3H); **^{13}C-NMR** (101 MHz, $CDCl_3$): δ 174.3, 83.0, 71.4, 45.0, 24.4, 4.0; **IR** (neat): ν 1600, 1395, 1263, 1056, 763, 749, 726, 697 cm^{-1}; **HRMS** (ESI$^+$, m/z): [M$^+$] calculated for [$C_9H_{17}N_2S^+$]: 185.1107, found: 185.1103.

2-(cyclohex-2-en-1-yl)-1,1,3,3-tetramethylisothiouronium bromide (1i)

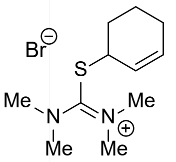

1i

Prepared according to general procedure **A** from TMTU (400 mg, 3.0 mmol, 1.0 equiv.) and 3-bromocyclohexene (0.36 mL, 3.15 mmol, 1.05 equiv.). White solid (850 mg, 2.9 mmol, 97% yield).

^{1}H NMR (400 MHz, $CDCl_3$) δ 6.00 – 5.91 (m, 1H), 5.67 – 5.57 (m, 1H), 4.13 – 3.99 (m, 1H), 3.48 (s, 12H), 2.12 – 1.98 (m, 3H), 1.92 – 1.58 (m, 3H); **^{13}C NMR** (101 MHz, $CDCl_3$) δ 174.2, 134.2, 123.7, 46.4, 45.1, 29.8, 24.5, 19.1; **IR** (neat) ν 3394, 3023, 2928, 1596, 1504, 1446, 1392, 1256, 1206, 1168, 1112, 1058, 1036, 993, 866, 749, 612 cm^{-1}; **HRMS** (ESI$^+$, m/z): [M$^+$] calculated for [$C_{11}H_{21}N_2S^+$]: 213.1420, found: 213.1416.

2-(3-(1,3-dioxo-2,3-dihydro-1H-inden-2-yl)propyl)-1,1,3,3-tetramethylisothiouronium bromide (1j)

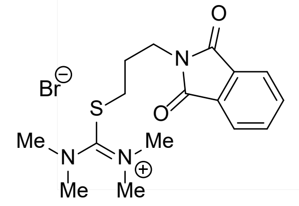

1j

Prepared according to general procedure **A** from TMTU (793 mg, 6.0 mmol, 1.0 equiv.) and N-(3-bromopropyl)phthalimide (1.77 g, 6.6 mmol, 1.1 equiv.). White solid (2.15 g, 5.4 mmol, 90% yield).

^{1}H NMR (600 MHz, $CDCl_3$) δ 7.83 (app dd, *J* = 5.4, 3.1 Hz, 2H), 7.74 (app dd, *J* = 5.5, 3.0 Hz, 2H), 3.85 (t, *J* = 6.4 Hz, 2H), 3.48 (s, 12H), 3.18 (t, *J* = 6.8 Hz, 2H), 2.11 (p, *J* = 6.6 Hz, 2H).; **^{13}C NMR** (151 MHz, $CDCl_3$) δ 175.8, 168.5, 134.5, 131.9, 123.6, 45.0, 36.1, 32.9, 28.6.; **IR** (neat) ν 3390, 2937, 1768, 1703, 2597, 1504, 1435, 1393, 1258, 1189, 1169, 1106, 1057, 1015, 871, 722 cm^{-1}; **HRMS** (ESI$^+$, m/z): [M$^+$] calculated for [$C_{16}H_{22}N_3O_2S^+$]: 320.1427, found: 320.1425. Mp.: 61–63 °C.

IV. Stereospecific thioetherification of optically active alcohols

a. General procedure B : Counteranion exchange of isothiouronium salts (synthesis of SbF_6^-, BF_4^- and PF_6^- salts)

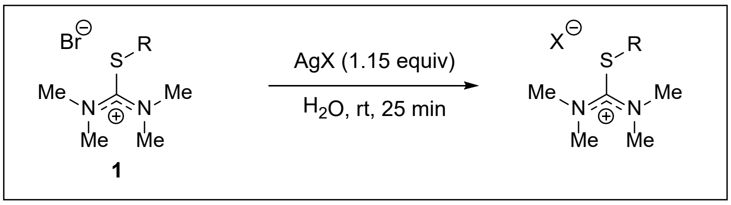

Corresponding silver salt (AgX, 1.15 equiv.) was added in one portion into a solution of bromide salt of isothiouronium **1** (1 equiv.) in H_2O (0.35 M) and the solution was stirred at rt for 30 min. Formed precipitate was filtered off and the mixture was then extracted with CH_2Cl_2 (3x), the combined organic phases were dried over $MgSO_4$, filtered and concentrated under reduced pressure to provide X^- thiouronium salt.

b. Thiouronium salt characterization

2-(4-methoxyphenethyl)-1,1,3,3-tetramethylisothiouronium hexafluoroantimonate (1aa)

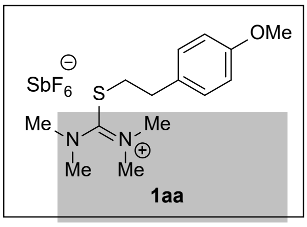

1aa

Prepared according to general procedure **B** from **1a** (800 mg, 2.30 mmol, 1.0 equiv.) and $AgSbF_6$ (910 mg, 2.65 mmol, 1.15 equiv.) as a thick yellowish oil (976 mg, 1.94 mmol, 84% yield).
^{1}H-NMR (700 MHz, $CDCl_3$) δ 7.14 (d, J = 8.6 Hz, 2H), 6.87 (d, J = 8.6 Hz, 2H), 3.78 (s, 3H), 3.24 (t, J = 7.1 Hz, 2H), 3.21 (s, 12H), 2.95 (t, J = 7.1 Hz, 2H); **^{13}C-NMR** (176 MHz, $CDCl_3$) δ 176.1, 159.0, 129.9, 129.8, 114.5, 55.5, 44.0, 36.6, 34.6; **^{19}F NMR** (659 MHz, $CDCl_3$) δ -112.59 – -134.63 (m); **IR** (neat): ν 2950, 2921, 2853, 1455, 1376, 1247, 760 cm^{-1}; **HRMS** (ESI$^+$, m/z): [M$^+$] calculated for $[C_{14}H_{23}N_2OS]^+$: 267.1526, found: 267.1523.

2-(4-methoxyphenethyl)-1,1,3,3-tetramethylisothiouronium tetrafluoroborate (1ab)

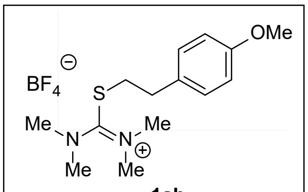

1ab

Prepared according to general procedure **B** from **1a** (695 mg, 2.0 mmol, 1.0 equiv.) and $AgBF_4$ (448 mg, 2.3 mmol, 1.15 equiv.) as a white-off solid (683 mg, 1.93 mmol, 96% yield).
^{1}H-NMR (400 MHz, $CDCl_3$) δ 7.15 (t, J = 5.7 Hz, 2H), 6.91 – 6.83 (m, 2H), 3.79 (s, 3H), 3.33 – 3.24 (m, 14H), 2.98 (dd, J = 7.8, 6.4 Hz, 2H).; **^{13}C-NMR** (101 MHz, $CDCl_3$) δ 176.0, 158.9, 130.1, 129.9, 114.4, 55.5, 44.1, 36.6, 34.6; **^{19}F NMR** (376 MHz, $CDCl_3$) δ -153.08 – -153.21 (m); **IR** (neat): ν 2938, 1598, 1512, 1467, 1397, 1246, 1050, 1034, 822, 536 cm^{-1}; **HRMS** (ESI$^+$, m/z): [M$^+$] calculated for $[C_{14}H_{23}N_2OS]^+$: 267.1526, found: 267.1523. Mp.: 89–91 °C.

2-(4-methoxyphenethyl)-1,1,3,3-tetramethylisothiouronium hexafluorophosphate (1ac)

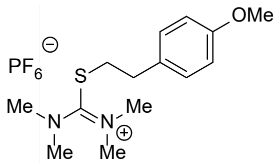

1ac

Prepared according to general procedure **B** from **1a** (764 mg, 2.20 mmol, 1.0 equiv.) and $AgPF_6$ (640 mg, 2.53 mmol, 1.15 equiv.) as a thick yellowish oil (740 mg, 1.97 mmol, 82% yield).
^{1}H-NMR (600 MHz, $CDCl_3$) δ 7.14 (d, *J* = 8.6 Hz, 2H), 6.86 (d, *J* = 8.6 Hz, 2H), 3.78 (s, 3H), 3.27 – 3.22 (m, 2H), 3.22 (s, 12H), 2.95 (t, *J* = 7.1 Hz, 2H).; **^{13}C-NMR** (176 MHz, $CDCl_3$) δ 176.0, 158.9, 130.0, 129.8, 114.4, 55.5, 44.0, 36.5, 34.5; **^{19}F NMR** (565 MHz, $CDCl_3$) δ -72.87 (d, *J* = 713.1 Hz); ^{31}P NMR (243 MHz, $CDCl_3$) δ -134.57 – -154.28 (m). **IR** (neat): v 2937, 1595, 1512, 1394, 1247, 1112, 1031, 829, 750, 555 cm^{-1}; **HRMS** (ESI$^+$, m/z): [M$^+$] calculated for $[C_{14}H_{23}N_2OS]^+$: 267.1526, found: 267.1522.

1,1,3,3-tetramethyl-2-(2-phenylethyl)isothiouronium hexafluoroantimonate (1bb)

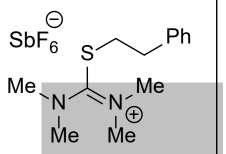

1bb

Prepared according to general procedure **B** from **1b** (317 mg, 1.00 mmol, 1.0 equiv.) and $AgSbF_6$ (395 mg, 1.15 mmol, 1.15 equiv.) as a thick yellowish oil (460 mg, 0.97 mmol, 97% yield).
^{1}H NMR (600 MHz, $CDCl_3$) δ 7.33 (t, *J* = 7.5 Hz, 2H), 7.25 (t, *J* = 7.4 Hz, 1H), 7.22 (d, *J* = 7.4 Hz, 2H), 3.28 (t, *J* = 7.1 Hz, 2H), 3.19 (s, 12H), 3.02 (t, *J* = 7.1 Hz, 2H); **^{13}C NMR** (151 MHz, $CDCl_3$) δ 176.1, 138.0, 129.1, 128.8, 127.5, 44.0, 36.3, 35.5; ^{19}F NMR (565 MHz, $CDCl_3$) δ -112.14 – -133.82 (m); **IR** (neat) v 2923, 2851, 1593, 1505, 1454, 1394, 1257, 1206, 1166, 1112, 1055, 869, 7753, 701, 651 cm^{-1}; **HRMS** (ESI$^+$, m/z): [M$^+$] calculated for $[C_{13}H_{21}N_2S]^+$: 237.1420, found: 237.1427.

2-ethyl-1,1,3,3-tetramethylisothiouronium hexafluoroantimonate (1cc)

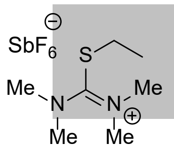

1cc

Prepared according to general procedure **B** from **1c** (957 mg, 3.97 mmol, 1.0 equiv.) and $AgSbF_6$ (1570 mg, 4.56 mmol, 1.15 equiv.) as a thick colorless oil (1010 mg, 2.54 mmol, 64% yield).
^{1}H-NMR (600 MHz, $CDCl_3$) δ 3.47 (s, 12H), 3.22 (q, J = 7.5 Hz, 2H), 1.42 (t, J = 7.4 Hz, 3H); **^{13}C-NMR** (151 MHz, $CDCl_3$) δ 176.1, 45.6, 30.6, 14.9; **^{19}F NMR** (377 MHz, $CDCl_3$) δ -106.52 – -139.77 (m); **IR** (neat): v 3503, 2941, 1595, 1396, 1255, 1057, 619 cm^{-1}; **HRMS** (ESI$^+$, m/z): [M$^+$] calculated for $[C_7H_{17}N_2S]^+$: 161.1107, found: 161.1106.

c. <u>General procedure C : Thioetherification of chiral secondary alcohols</u>

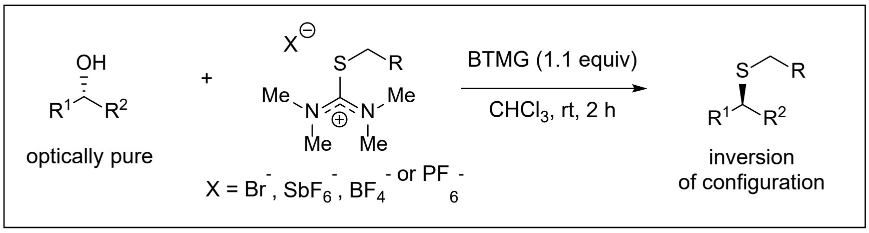

BTMG (2-*tert*-butyl-1,1,3,3-tetramethylguanidine, Barton's base) (68 µL, 0.33 mmol, 1.1 equiv.) was added dropwise to a solution of alcohol (0.30 mmol, 1.0 equiv.) and isothiouronium salt (0.30 mmol, 1.0 equiv.) in dry $CHCl_3$ (2.2 mL) under an Ar atmosphere. The reaction mixture was then stirred at rt for 2 h before excess base was quenched by addition of a saturated solution of NH_4Cl. The mixture was then extracted

with CH_2Cl_2 (3x), the combined organic phases were dried over $MgSO_4$, filtered and concentrated under reduced pressure. The residue was purified by column chromatography.

d. Chiral thioether characterization

(*S*)-(4-methoxyphenethyl)(1-phenylethyl)sulfane (2a)

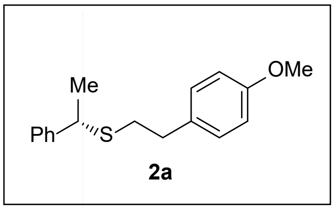

2a

Prepared according to general procedure **C** from (*R*)-1-phenylethan-1-ol (37 mg, 0.30 mmol, 1.0 equiv., >99% ee) and **1a** or **1aa** (1.0 equiv.). Purification on silica gel (heptane/Et_2O = 24:1). Colorless oil (from **1a**: 58 mg, 0.21 mmol, 71%, 73% ee; from **1aa**: 60 mg, 0.22 mmol, 73% yield, 96% ee). **^{1}H NMR** (600 MHz, $CDCl_3$) δ 7.35 – 7.30 (m, 4H), 7.25 – 7.21 (m, 1H), 7.00 (d, *J* = 8.6 Hz, 2H), 6.80 (d, *J* = 8.6 Hz, 2H), 3.95 (q, *J* = 7.0 Hz, 1H), 3.77 (s, 3H), 2.77 – 2.60 (m, 2H), 2.59 – 2.46 (m, 2H), 1.56 (d, *J* = 7.0 Hz, 3H); **^{13}C-NMR** (151 MHz, $CDCl_3$) δ 158.2, 144.2, 132.9, 129.5, 128.6, 127.5, 127.2, 113.9, 55.4, 44.4, 35.4, 33.2, 22.7; **IR** (neat): ν 2960, 2833, 1611, 1510, 1451, 1244, 1176, 1034, 848, 763, 698 cm^{-1}; **HRMS** (ESI$^+$, m/z): $[M+Na]^+$ calculated for $[C_{17}H_{20}OSNa]^+$: 295.1127, found: 295.1126.

Racemic standard:

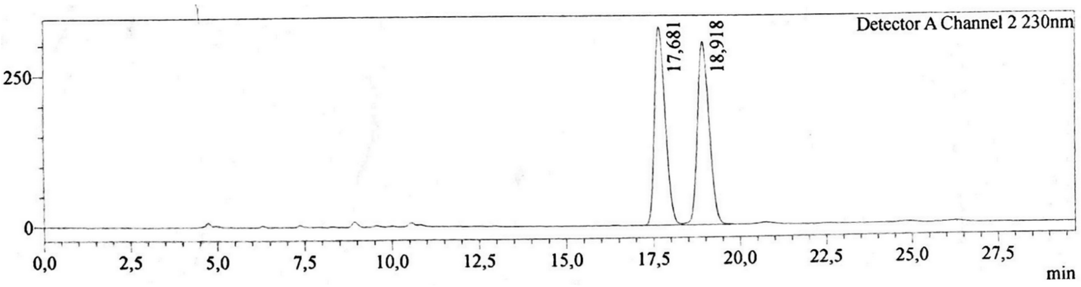

Method Description:
Column:Lux-3 Cellulose-3
250x4,6mm Particle Size 3 micrometer
Solvent System: n-Heptan/IPA 9:1
Flow=0.7 ml/min T=25°C

Detector A Channel 2 230nm

Peak#	Ret. Time	Area	Area%
1	17,681	7064813	49,665
2	18,918	7160182	50,335
Total		14224995	100,000

From **1a**:

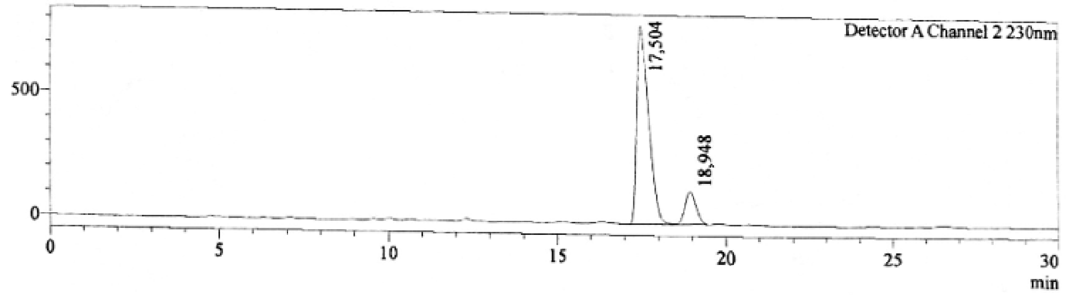

Method Description:
Column:Lux-3 Cellulose-3
250x4,6mm Particle Size 3 micrometer
Solvent System: n-Heptan/IPA 9:1
Flow=0.7 ml/min T=25°C

Detector A Channel 2 230nm

Peak#	Ret. Time	Area	Area%
1	17,504	19126452	86,385
2	18,948	3014521	13,615
Total		22140973	100,000

From **1aa**:

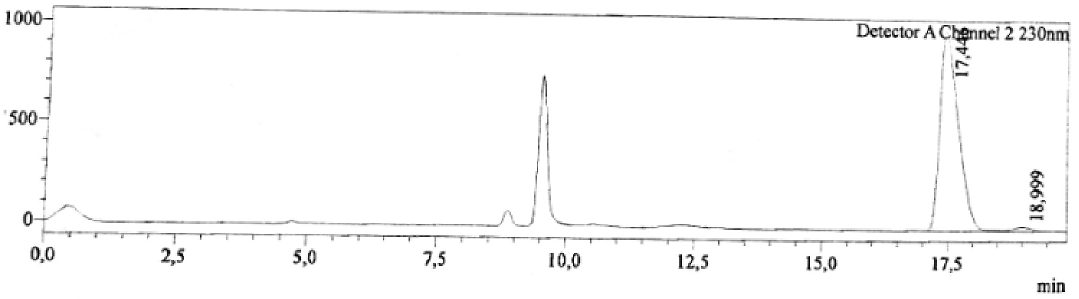

Method Description:
Column:Lux-3 Cellulose-3
250x4,6mm Particle Size 3 micrometer
Solvent System: n-Heptan/IPA 9:1
Flow=0.7 ml/min T=25°C

Detector A Channel 2 230nm

Peak#	Ret. Time	Area	Area%
1	17,446	25906098	98,049
2	18,999	515569	1,951
Total		26421667	100,000

From **1ab**:

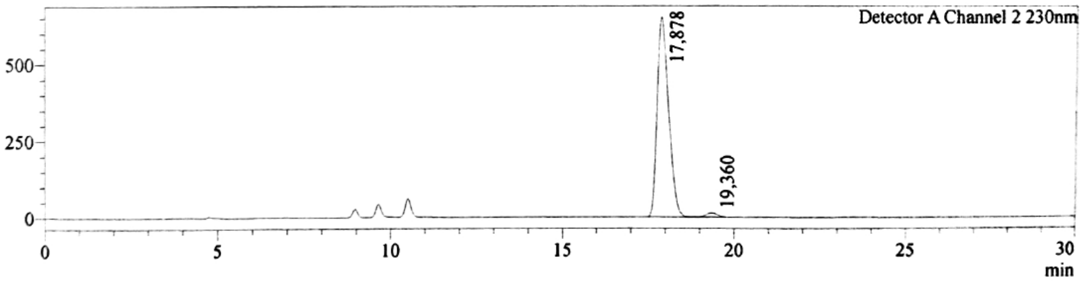

Method Description:
Column:Lux-3 Cellulose-3
250x4,6mm Particle Size 3 micrometer
Solvent System: n-Heptan/IPA 9:1
Flow=0.7 ml/min T=25°C

Detector A Channel 2 230nm

Peak#	Ret. Time	Area	Area%
1	17,878	15410471	97,870
2	19,360	335350	2,130
Total		15745821	100,000

From **1ac**:

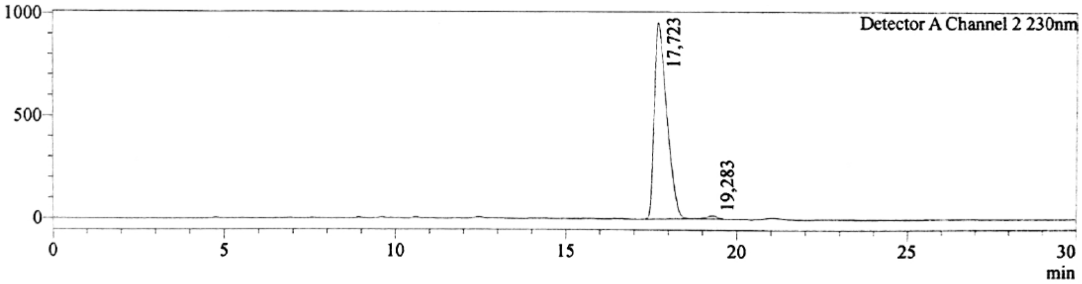

Method Description:
Column:Lux-3 Cellulose-3
250x4,6mm Particle Size 3 micrometer
Solvent System: n-Heptan/IPA 9:1
Flow=0.7 ml/min T=25°C

Detector A Channel 2 230nm

Peak#	Ret. Time	Area	Area%
1	17,723	24212075	98,828
2	19,283	287147	1,172
Total		24499222	100,000

CH_3CN used as reaction solvent (from **1aa**):

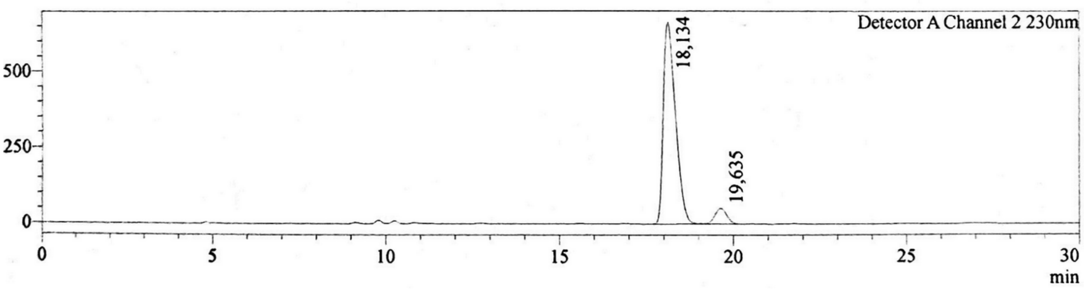

Method Description:
Column:Lux-3 Cellulose-3
250x4,6mm Particle Size 3 micrometer
Solvent System: n-Heptan/IPA 9:1
Flow=0.7 ml/min T=25°C

Detector A Channel 2 230nm

Peak#	Ret. Time	Area	Area%
1	18,134	15999757	92,838
2	19,635	1234277	7,162
Total		17234035	100,000

DMF used as reaction solvent (from **1aa**):

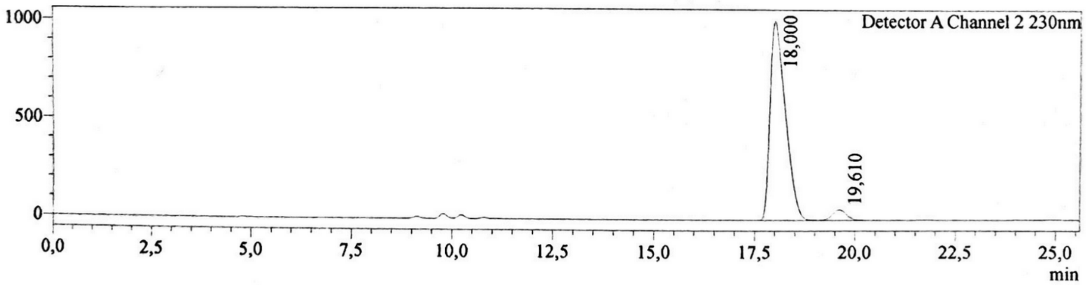

Method Description:
Column:Lux-3 Cellulose-3
250x4,6mm Particle Size 3 micrometer
Solvent System: n-Heptan/IPA 9:1
Flow=0.7 ml/min T=25°C

Detector A Channel 2 230nm

Peak#	Ret. Time	Area	Area%
1	18,000	26633172	95,212
2	19,610	1339398	4,788
Total		27972569	100,000

(*S*)-(4-methoxyphenethyl)(1-phenylethyl)sulfane (2b)

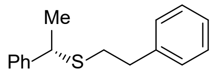

2b

Prepared according to general procedure **C** from (*R*)-1-phenylethan-1-ol (37 mg, 0.30 mmol, 1.0 equiv., >99% ee) and **1bb** (1.0 equiv.)**.** Purification on silica gel (heptane/EtOAc = 99:1). Colorless oil (56 mg, 0.22 mmol, 77%, 97% ee).

^{1}H NMR (600 MHz, $CDCl_3$) δ 7.35 – 7.30 (m, 4H), 7.27 – 7.22 (m, 3H), 7.21 – 7.17 (m, 1H), 7.11 – 7.07 (m, 2H), 3.96 (q, *J* = 7.0 Hz, 1H), 2.84 – 2.68 (m, 2H), 2.61 – 2.51 (m, 2H), 1.57 (d, *J* = 7.0 Hz, 3H); **^{13}C-NMR** (150 MHz, $CDCl_3$): δ 144.1, 140.8, 128.64, 128.60, 128.5, 127.5, 127.2, 126.4, 44.4, 36.3, 32.9, 22.7; **IR** (neat): v 1255, 748, 723, 699 cm^{-1}; **HRMS** (ESI^+, m/z): $[M+Na]^+$ calculated for $[C_{16}H_{18}SNa]^+$: 265.1021, found: 265.1019.

Racemic standard:

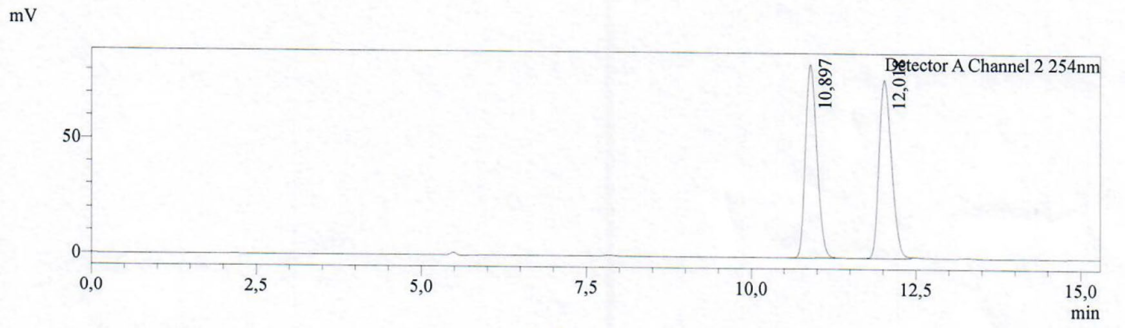

Detector A Channel 2 254nm

Peak#	Ret. Time	Area	Area%
1	10,897	1013158	49,894
2	12,018	1017467	50,106
Total		2030626	100,000

Method Description:
Column:Lux-3 Cellulose-3
250x4,6mm Particle Size 3 micrometer
Solvent System: n-Heptan/EtOH 97:3
Flow=0,7 ml/min T=25°C

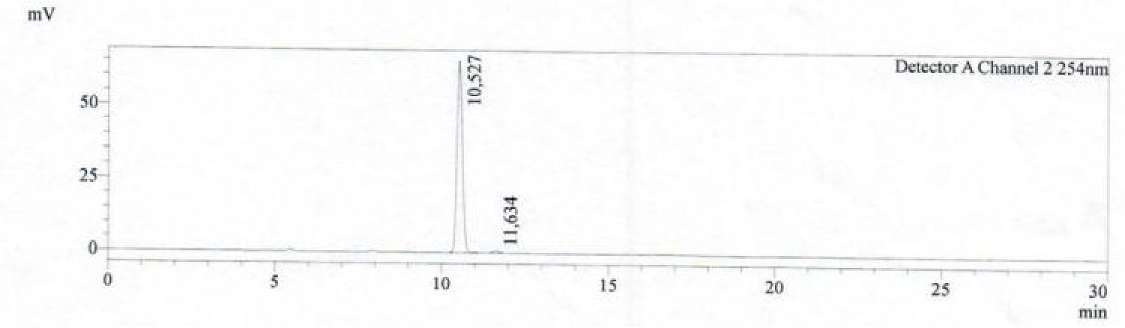

Method Description:
Column:Lux-3 Cellulose-3
250x4,6mm Particle Size 3 micrometer
Solvent System: n-Heptan/EtOH 97:3
Flow=0,7 ml/min T=25°C

Detector A Channel 2 254nm

Peak#	Ret. Time	Area	Area%
1	10,527	752767	98,694
2	11,634	9958	1,306
Total		762725	100,000

(*R*)-(4-methoxyphenethyl)(1-(naphthalen-2-yl)ethyl)sulfane (2c)

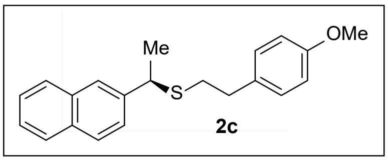

Prepared according to general procedure **C** from (*S*)-1-(naphthalen-2-yl)ethan-1-ol (52 mg, 0.30 mmol, 1.0 equiv., >98% ee) and **1aa** (151 mg, 0.30 mmol, 1.0 equiv.)**.** Purification on silica gel (heptane/Et_2O = 24:1). Colorless oil (67 mg, 0.21 mmol, 69% yield, 82% ee).

^{1}H NMR (600 MHz, $CDCl_3$) δ 7.85 – 7.78 (m, 3H), 7.67 (bs, 1H), 7.55 (dd, *J* = 8.5, 1.8 Hz, 1H), 7.50 – 7.44 (m, 2H), 6.97 (d, *J* = 8.6 Hz, 2H), 6.77 (d, *J* = 8.6 Hz, 2H), 4.11 (q, *J* = 7.0 Hz, 1H), 3.76 (s, 3H), 2.77 – 2.62 (m, 2H), 2.58 – 2.46 (m, 2H), 1.64 (d, *J* = 7.0 Hz, 3H); **^{13}C-NMR** (151 MHz, $CDCl_3$) δ 158.2, 141.4, 133.3, 132.9, 132.8, 129.5, 128.7, 127.8, 127.8, 126.3, 126.0, 125.9, 125.5, 113.9, 55.4, 44.7, 35.4, 33.2, 22.6; **IR** (neat): ν 2951, 2833, 1610, 1510, 1440, 1243, 1175, 1034, 817, 747 cm^{-1}; **HRMS** (ESI^+, m/z): $[M+Na]^+$ calculated for $[C_{21}H_{22}OSNa]^+$: 345.1284, found: 345.1282.

Racemic standard:

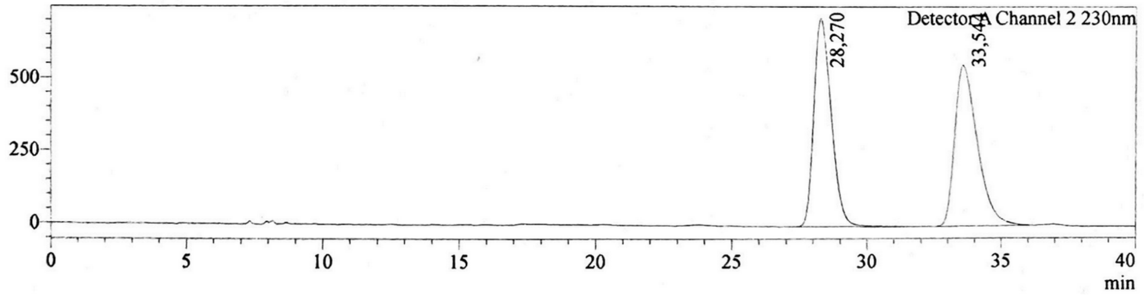

Method Description:
Column:Lux-3 Cellulose-3
250x4,6mm Particle Size 3 micrometer
Solvent System: n-Heptan/IPA 8:2
Flow=0,7 ml/min T=25°C

Detector A Channel 2 230nm

Peak#	Ret. Time	Area	Area%
1	28,270	32190806	49,534
2	33,544	32795841	50,466
Total		64986647	100,000

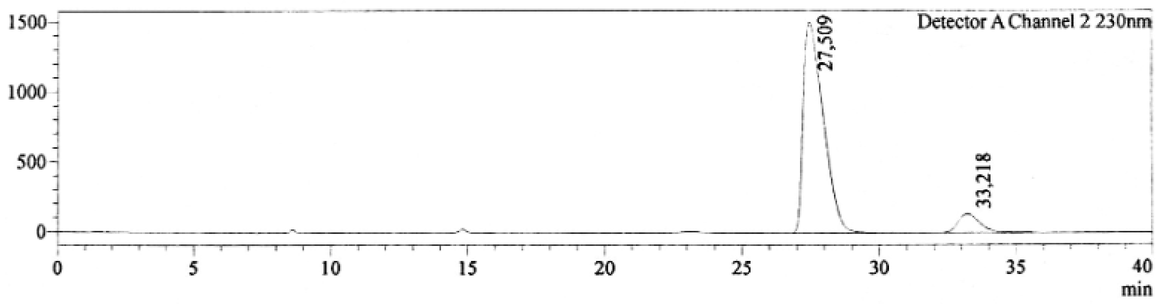

Method Description:
Column:Lux-3 Cellulose-3
250x4,6mm Particle Size 3 micrometer
Solvent System: n-Heptan/IPA 8:2
Flow=0,7 ml/min T=25°C

Detector A Channel 2 230nm

Peak#	Ret. Time	Area	Area%
1	27,509	76665151	91,093
2	33,218	7495929	8,907
Total		84161080	100,000

(*S*)-(1-(3,5-bis(trifluoromethyl)phenyl)ethyl)(4-methoxyphenethyl)sulfane (2d)

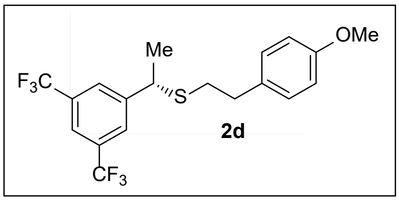

Prepared according to general procedure **C** from (*R*)-1-(3,5-bis(trifluoromethyl)phenyl)ethan-1-ol (77 mg, 0.30 mmol, 1.0 equiv., >98% ee) and **1aa** (151 mg, 0.30 mmol, 1.0 equiv.). Purification on silica gel (heptane/Et_2O = 24:1). Colorless oil (78 mg, 0.19 mmol, 64% yield, >99% ee).

^{1}H NMR (600 MHz, $CDCl_3$) δ 7.75 (s, 3H), 7.02 (d, *J* = 8.7 Hz, 2H), 6.82 (d, *J* = 8.7 Hz, 2H), 3.96 (q, *J* = 7.1 Hz, 1H), 3.78 (s, 3H), 2.77 – 2.71 (m, 2H), 2.57 – 2.48 (m, 2H), 1.56 (d, *J* = 7.1 Hz, 3H); **^{13}C-NMR** (151 MHz, $CDCl_3$) δ 158.4, 147.2, 132.2, 131.9 (q, *J* = 33.2 Hz), 129.6, 127.7 (d, *J* = 2.6 Hz), 123.4 (q, *J* = 273.3 Hz), 121.3 (dt, *J* = 7.5, 3.6), 114.1, 55.4, 43.8, 35.3, 33.3, 22.7; **^{19}F NMR** (659 MHz, $CDCl_3$) δ -62.79. **IR** (neat): v 2929, 1612, 1512, 1373, 1301, 1246, 1168, 1126, 1036, 896, 705, 680 cm^{-1}; **HRMS** (ESI$^+$, m/z): $[M+Na]^+$ calculated for $[C_{19}H_{18}F_6OSNa]^+$: 431.0875, found: 431.0878.

Racemic standard:

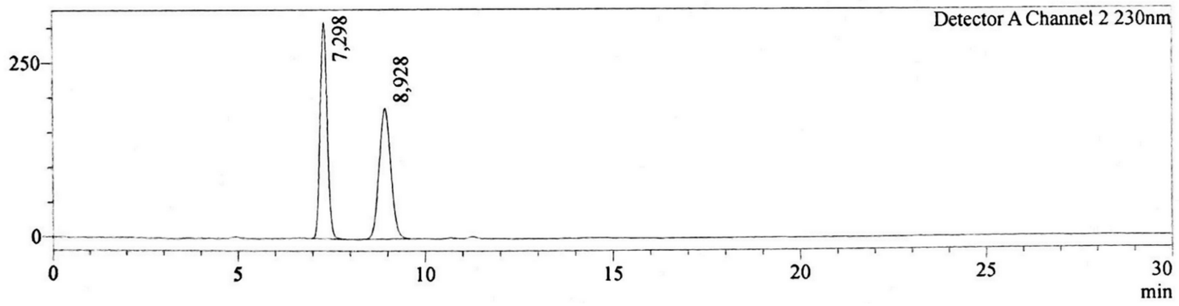

Method Description:
Column:Lux-3 Cellulose-3
150x4,6mm Particle Size 3 micrometer
Solvent System: n-Heptan/IPA 95:5
Flow=0,7 ml/min T=25°C

Detector A Channel 2 230nm

Peak#	Ret. Time	Area	Area%
1	7,298	4011556	49,749
2	8,928	4051994	50,251
Total		8063551	100,000

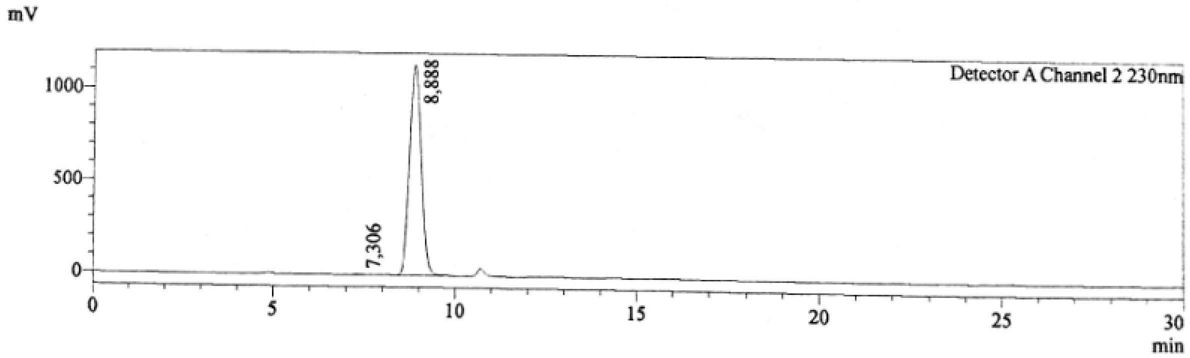

Method Description:
Column:Lux-3 Cellulose-3
250x4.6mm Particle Size 3 micrometer
Solvent System: n-Heptan/IPA 95:5
Flow=0,7 ml/min T=25°C

Detector A Channel 2 230nm

Peak#	Ret. Time	Area	Area%
1	7,306	38262	0,149
2	8,888	25578937	99,851
Total		25617199	100,000

(*R*)-but-3-yn-2-yl(4-methoxyphenethyl)sulfane (2e)

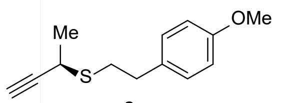

2e

Prepared according to general procedure **C** from (*S*)-3-butyn-2-ol (21 mg, 0.30 mmol, 1.0 equiv., >98% ee) and **1aa** (151 mg, 0.30 mmol, 1.0 equiv.). Purification on silica gel (heptane/Et_2O = 49:1). Colorless oil (38 mg, 0.17 mmol, 58% yield, 98% ee).

^{1}H NMR (600 MHz, $CDCl_3$) δ 7.14 (d, *J* = 8.5 Hz, 2H), 6.84 (d, *J* = 8.5 Hz, 2H), 3.79 (s, 3H), 3.61 (qd, *J* = 7.0, 2.3 Hz, 1H), 3.02 – 2.94 (m, 1H), 2.93 – 2.84 (m, 3H), 2.36 (d, *J* = 2.4 Hz, 1H), 1.50 (d, *J* = 7.0 Hz, 3H); **^{13}C-NMR** (151 MHz, $CDCl_3$) δ 158.3, 132.7, 129.6, 114.0, 84.8, 71.0, 55.4, 35.2, 33.2, 29.1, 21.6; **IR** (neat): ν 3286, 2930, 1610, 1511, 1442, 1244, 1176, 1034, 848, 635 cm^{-1}; **HRMS** (ESI^+, m/z): $[M+Na]^+$ calculated for $[C_{13}H_{16}OSNa]^+$: 243.0814, found: 243.0814.

Racemic standard:

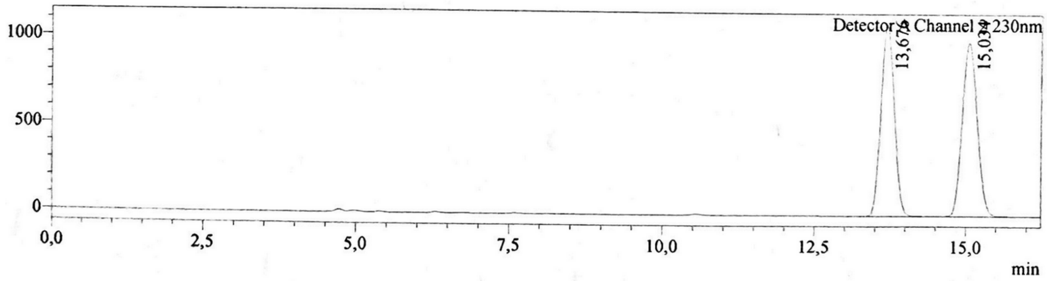

Method Description:
Column:Lux-3 Cellulose-3
250x4,6mm Particle Size 3 micrometer
Solvent System: n-Heptan/IPA 9:1
Flow=0.7 ml/min T=25°C

Detector A Channel 2 230nm

Peak#	Ret. Time	Area	Area%
1	13,676	16941297	49,685
2	15,034	17156249	50,315
Total		34097547	100,000

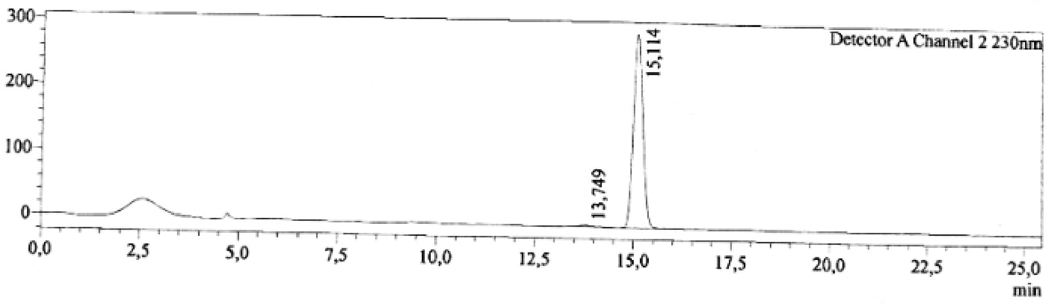

Method Description:
Column:Lux-3 Cellulose-3
250x4,6mm Particle Size 3 micrometer
Solvent System: n-Heptan/IPA 9:1
Flow=0.7 ml/min T=25°C

Detector A Channel 2 230nm

Peak#	Ret. Time	Area	Area%
1	13,749	50165	1,030
2	15,114	4820965	98,970
Total		4871130	100,000

(*R*)-(4-methoxyphenethyl)(oct-1-yn-3-yl)sulfane (2f)

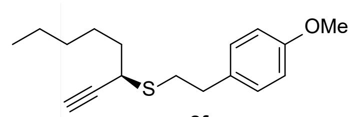

2f

Prepared according to general procedure **C** from (*S*)-1-Octyn-3-ol (38 mg, 0.30 mmol, 1.0 equiv., 99% ee) and **1aa** (151 mg, 0.30 mmol, 1.0 equiv.). Purification on silica gel (heptane/Et_2O = 49:1). Colorless oil (42 mg, 0.15 mmol, 51% yield, >97% ee).

^{1}H NMR (700 MHz, $CDCl_3$) δ 7.14 (d, *J* = 8.5 Hz, 2H), 6.84 (d, *J* = 8.5 Hz, 2H), 3.79 (s, 3H), 3.50 – 3.47 (m, 1H), 3.00 – 2.94 (m, 1H), 2.92 – 2.81 (m, 3H), 2.36 (d, *J* = 2.3 Hz, 1H), 1.77 – 1.67 (m, 2H), 1.57 – 1.45 (m, 2H), 1.36 – 1.25 (m, 4H), 0.90 (t, *J* = 7.0 Hz, 3H); **^{13}C-NMR** (176 MHz, $CDCl_3$) δ 158.3, 132.8, 129.6, 114.0, 83.8, 71.7, 55.4, 35.3, 35.2, 34.6, 33.1, 31.4, 27.1, 22.6, 14.1; **IR** (neat): ν 3284, 2923, 2857, 1611, 1511, 1464, 1301, 1245, 1176, 1035, 820, 636 cm^{-1}; **HRMS** (ESI^+, m/z): $[M+Na]^+$ calculated for $[C_{17}H_{24}OSNa]^+$: 299.1440, found: 299.1436.

Racemic standard:

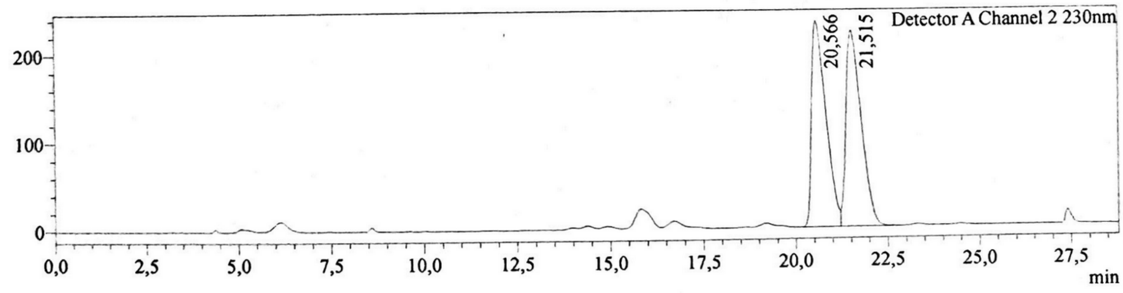

Method Description:
Column:Lux-Cellulose1 (Chiralcel OD-H) 250x4,6mm
Solvent System: n-Heptan+0,1%IPA
Flow:1 ml/min

Detector A Channel 2 230nm

Peak#	Ret. Time	Area	Area%
1	20,566	6342907	49,736
2	21,515	6410291	50,264
Total		12753198	100,000

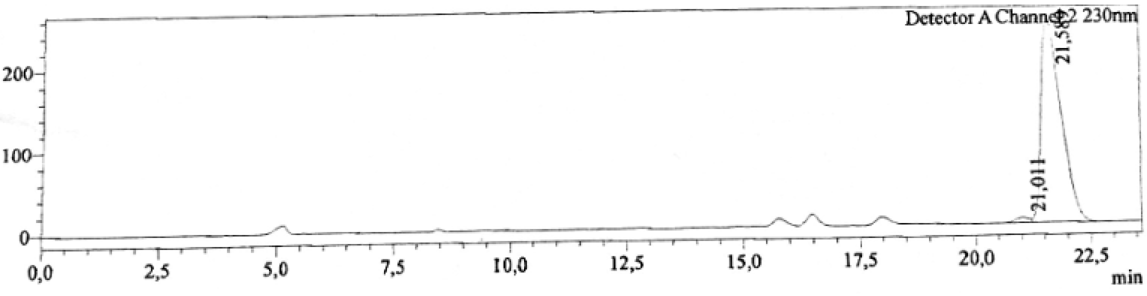

Method Description:
Column:Lux-Cellulose1 (Chiralcel OD-H) 250x4,6mm
Solvent System: n-Heptan+0,1%IPA
Flow:1 ml/min

Detector A Channel 2 230nm

Peak#	Ret. Time	Area	Area%
1	21,011	133056	1,739
2	21,589	7519103	98,261
Total		7652159	100,000

(1*R*,4*R*)-4-(phenethylthio)cyclopent-2-en-1-yl acetate (2g)

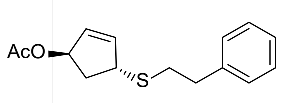

2g

Prepared according to general procedure **C** from (1*R*,4*S*)-4-hydroxycyclopent-2-en-1-yl acetate (43 mg, 0.30 mmol, 1.0 equiv., >99%) and **1b** or **1bb** (1.0 equiv.). Purification on silica gel (heptane/Et_2O = 9:1). Colorless oil (from **1b**: 54 mg, 0.21 mmol, 69% yield, d.r. 94:6; from **1bb**: 58 mg, 0.22 mmol, 74% yield, one diastereomer).

^{1}H NMR (700 MHz, $CDCl_3$) δ 7.30 (t, *J* = 7.4 Hz, 2H), 7.22 (t, *J* = 7.4 Hz, 1H), 7.20 (d, *J* = 7.4 Hz, 2H), 6.04 (ddd, *J* = 5.5, 2.1, 0.9 Hz, 1H), 5.91 (dt, *J* = 5.5, 2.0 Hz, 1H), 5.77 – 5.74 (m, 1H), 4.01 – 3.98 (m, 1H), 2.93 – 2.82 (m, 2H), 2.81 – 2.70 (m, 2H), 2.34 – 2.25 (m, 2H), 2.03 (s, 3H); **^{13}C-NMR** (176 MHz, $CDCl_3$) δ 171.0, 140.5, 138.6, 131.0, 128.7, 128.6, 126.6, 79.6, 48.2, 39.1, 36.5, 32.2, 21.3; **IR** (neat): ν 3027, 3920, 1728, 1496, 1357, 1235, 1174, 1017, 982, 722, 696 cm^{-1}; **HRMS** (ESI^+, m/z): $[M+Na]^+$ calculated for $[C_{15}H_{18}O_2SNa]^+$: 285.0920, found: 285.0917.

(*R*)-(1-(2-bromophenyl)ethyl)(ethyl)sulfane (2h)

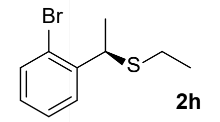

2h

Prepared according to general procedure **C** from (*S*)-1-(2-bromophenyl)ethan-1-ol (40 mg, 0.20 mmol, 1.0 equiv., >98%) and **1cc** (1.0 equiv.). Purification on silica gel (pentane). Yellowish oil (33 mg, 0.14 mmol, 67% yield, >98% ee).

^{1}H NMR (600 MHz, $CDCl_3$) δ 7.63 (dd, J = 7.8, 1.7 Hz, 1H), 7.52 (dd, J = 8.0, 1.2 Hz, 1H), 7.35 – 7.29 (m, 1H), 7.10 – 7.05 (m, 1H), 4.57 (q, J = 7.0 Hz, 1H), 2.51 – 2.32 (m, 2H), 1.51 (d, J = 7.0 Hz, 3H), 1.19 (t, J = 7.4 Hz, 3H); **^{13}C-NMR** (151 MHz, $CDCl_3$) δ 143.5, 132.7, 128.8, 128.4, 128.1, 124.0, 42.3, 25.5, 22.3, 14.8; **IR** (neat): ν 2967, 2925, 1467, 1020, 753, 726, 658 cm^{-1}; **HRMS** (ESI^+, m/z): $[M+H]^+$ calculated for $[C_{10}H_{14}S(^{79}Br)]^+$: 244.9994, found: 244.9994; calculated for $[C_{10}H_{14}S(^{81}Br)]^+$: 246.9974, found: 246.9973. **$[\alpha]_D^{20}$** = - 57.4 (*c* = 1, $CHCl_3$) [lit. **$[\alpha]_D^{20}$** = - 54.7 (*c* = 1, $CHCl_3$)][1]

Racemic standard:

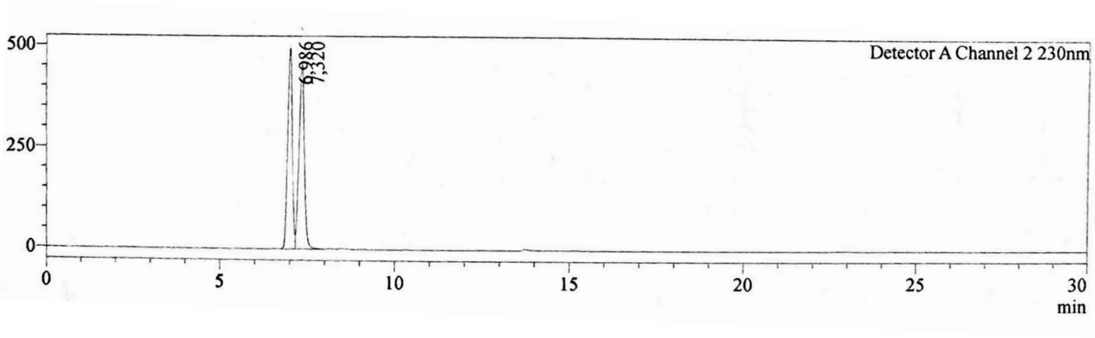

Method Description:
Column:Lux-3 Cellulose-3
250x4,6mm Particle Size 3 micrometer
Solvent System: n-Heptan+0,1%IPA/n-Heptan+2%IPA50:50
Flow=0,7 ml/min T=25°C

Detector A Channel 2 230nm

Peak#	Ret. Time	Area	Area%
1	6,986	4623068	49,243
2	7,320	4765293	50,757
Total		9388361	100,000

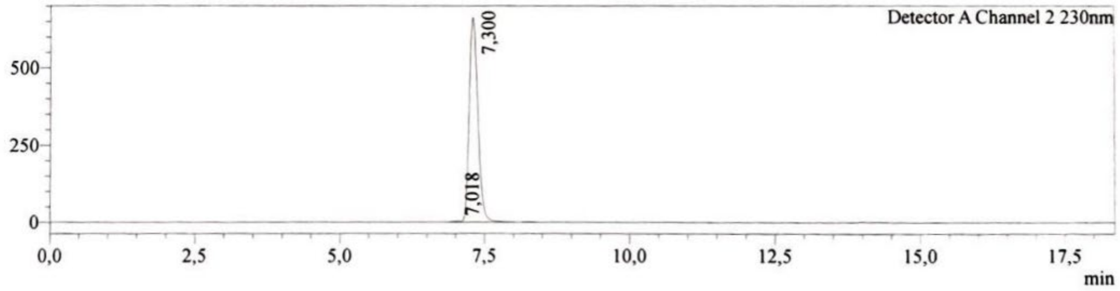

Method Description:
Column:Lux-3 Cellulose-3
250x4,6mm Particle Size 3 micrometer
Solvent System: n-Heptan+0,1%IPA/n-Heptan+2%IPA50:50
Flow=0,7 ml/min T=25°C

Detector A Channel 2 230nm

Peak#	Ret. Time	Area	Area%
1	7,018	23369	0,335
2	7,300	6948940	99,665
Total		6972310	100,000

Methyl 2-(phenethylthio)-2-phenylacetate (2i)

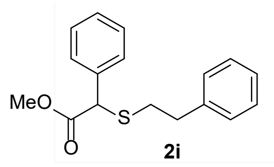

Prepared according to general procedure **C** from methyl L-mandelate (50 mg, 0.30 mmol, 1.0 equiv., >99%) and **1bb** (95 mg, 0.30 mmol, 1.0 equiv.). Purification on silica gel (heptane/EtOAc = 19:1). Colorless oil (61 mg, 0.21 mmol, 71% yield, 0% ee).

^{1}H NMR (600 MHz, $CDCl_3$) δ 7.45 (d, J = 7.5 Hz, 2H), 7.38 – 7.25 (m, 5H), 7.21 (t, J = 7.5 Hz, 1H), 7.14 (d, J = 7.5 Hz, 2H), 4.58 (s, 1H), 3.73 (s, 3H), 2.89 – 2.63 (m, 4H); **^{13}C-NMR** (151 MHz, $CDCl_3$) δ 171.4, 140.3, 136.1, 128.9, 128.67, 128.65 (2xC), 128.4, 126.6, 52.9, 52.4, 35.9, 33.4.; **IR** (neat): ν 3027, 2950, 1735,, 1496, 1453, 1301, 1146, 1006, 729, 695 cm^{-1}; **HRMS** (ESI^+, m/z): $[M+Na]^+$ calculated for $[C_{17}H_{18}O_2SNa]^+$: 309.0920, found: 309.0918.

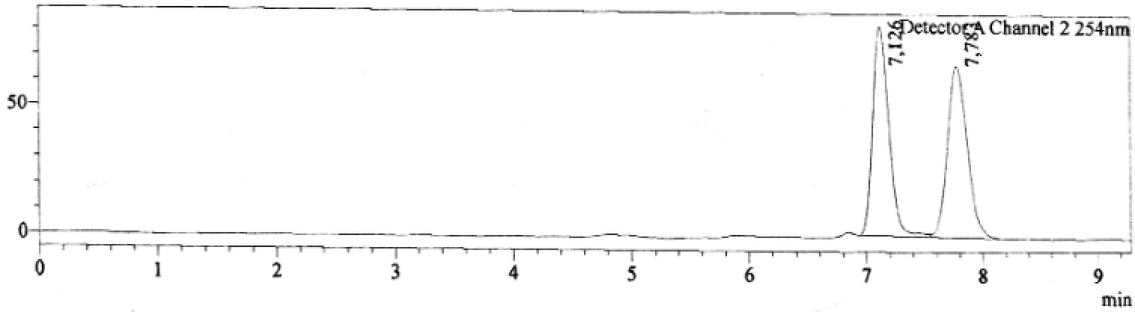

Method Description:
Column:Lux-Cellulose1 (Chiralcel OD-H) 250x4,6mm
Solvent System: n-Heptan+0,1%IPA/EtOH 8:2
Flow:0,7ml/min

Detector A Channel 2 254nm

Peak#	Ret. Time	Area	Area%
1	7,126	783053	49,659
2	7,783	793818	50,341
Total		1576870	100,000

Methyl 2-(phenethylthio)propanoate (2j)

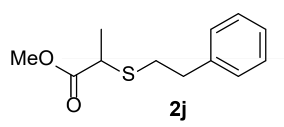

Prepared according to general procedure **C** from methyl L-lactate (31 mg, 0.30 mmol, 1.0 equiv., >97%) and **1bb** (95 mg, 0.30 mmol, 1.0 equiv.). Purification on silica gel (heptane/EtOAc = 19:1). Colorless oil (44 mg, 0.20 mmol, 66% yield, 0% ee).

^{1}H NMR (600 MHz, $CDCl_3$) δ 7.30 (t, J = 7.3 Hz, 2H), 7.24 – 7.18 (m, 3H), 3.74 (s, 3H), 3.43 (q, J = 7.1 Hz, 1H), 2.96 – 2.79 (m, 4H), 1.45 (d, J = 7.1 Hz, 3H); **^{13}C-NMR** (151 MHz, $CDCl_3$) δ 173.7, 140.4, 128.6 (2xC), 126.6, 52.4, 41.2, 36.1, 33.0, 17.3; **IR** (neat): ν 2950, 1711, 1451, 1327, 1260, 1157, 1065, 697 cm^{-1}; **HRMS** (ESI^+, m/z): $[M+Na]^+$ calculated for $[C_{12}H_{16}O_2SNa]^+$: 247.0763, found: 247.0760.

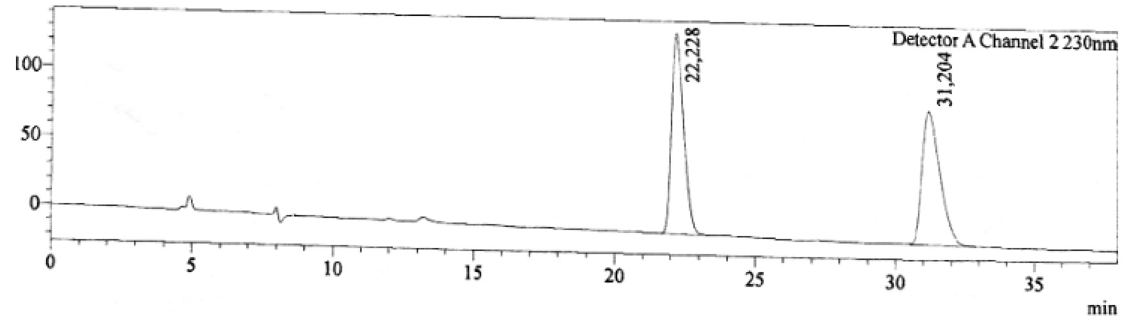

Method Description:
Column:Lux-3 Cellulose-3
250x4,6mm Particle Size 3 micrometer
Solvent System:(n-Heptan+0,1%IPA)/IPA 85:15
Flow=0,7ml/min T=25°C

Detector A Channel 2 230nm

Peak#	Ret. Time	Area	Area%
1	22,228	4248750	49,562
2	31,204	4323896	50,438
Total		8572646	100,000

Methyl 3-(phenethylthio)butanoate (2k)

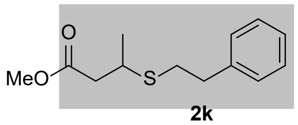

2k

Prepared according to general procedure **C** from methyl *S*-3-hydroxybutyrat (35 mg, 0.30 mmol, 1.0 equiv., 99%) and **1bb** (95 mg, 0.30 mmol, 1.0 equiv.). Purification on silica gel (heptane/EtOAc = 19:1). Colorless oil (30 mg, 0.13 mmol, 42% yield, 0% ee).

^{1}H NMR (700 MHz, $CDCl_3$) δ 7.30 (t, *J* = 7.6 Hz, 2H), 7.24 – 7.19 (m, 3H), 3.69 (s, 3H), 3.30 – 3.17 (m, 1H), 2.88 (dd, *J* = 9.3, 6.4 Hz, 2H), 2.85 – 2.78 (m, 2H), 2.63 (dd, *J* = 15.5, 6.3 Hz, 1H), 2.46 (dd, *J* = 15.5, 8.1 Hz, 1H), 1.33 (d, *J* = 6.8 Hz, 3H); **^{13}C-NMR** (176 MHz, $CDCl_3$) δ 172.1, 140.6, 128.63, 128.60, 126.5, 51.9, 42.3, 36.5, 36.4, 32.4, 21.6; **IR** (neat): ν 2953, 1733, 1497, 1453, 1435, 1221, 1158, 1028, 747, 697 cm^{-1}: **HRMS** (ESI^+, m/z): $[M+Na]^+$ calculated for $[C_{13}H_{18}O_2SNa]^+$: 261.0920, found: 261.0920.

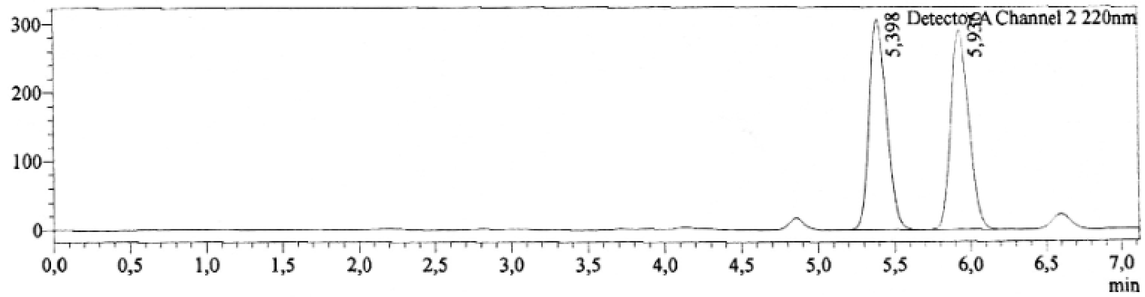

Method Description:
Column:Lux-Cellulose1 (Chiralcel OD-H) 250x4,6mm
Solvent System: n-Heptan+0,1%IPA/IPA 9:1
Flow:1 ml/min

Detector A Channel 2 220nm

Peak#	Ret. Time	Area	Area%
1	5,398	2383393	49,995
2	5,936	2383914	50,005
Total		4767307	100,000

V. Late-stage transformation of biologically relevant molecules

a. General procedure D: attempted thioether synthesis using thio-Mitsunobu reaction conditions

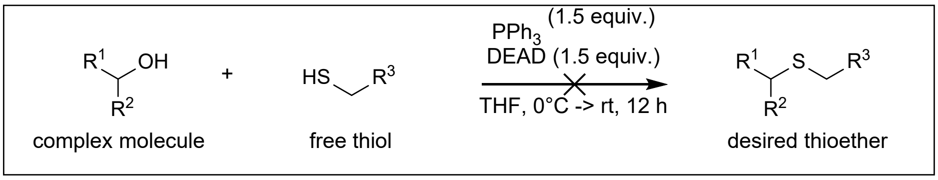

Reaction conditions were adapted from the literature with changes.[3] To a stirred solution of alcohol (0.2 mmol, 1.0 equiv.) and PPh_3 (0.3 mmol, 1.5 equiv.) in dry THF (2 mL), diethyl azodicarboxylate (55 µL, 0.3 mmol, 1.5 equiv.) was added dropwise at 0°C. Stirring continued for 10 min at 0°C, after which thiol (0.2 mmol, 1.0 equiv) was added and the resulting mixture was stirred at rt for 12h. Volatiles were evaporated and ^{1}H NMR spectra of the remaining crude mixture was measured.

b. Thioether characterization

2-methyl-5-nitro-1-(2-(phenethylthio)ethyl)-1*H*-imidazole (3a)

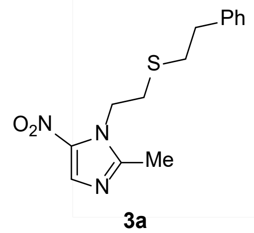

3a

Prepared according to general procedure **C** from metronidazole (51 mg, 0.30 mmol, 1.0 equiv.) and **1b** (95 mg, 0.30 mmol, 1.0 equiv.)**.** Purification on silica gel (heptane/EtOAc = 2:1). Pale yellow oil (55 mg, 0.19 mmol, 63% yield). **^{1}H-NMR** (400 MHz, $CDCl_3$): δ 7.87 (s, 1H), 7.25 – 7.19 (m, 2H), 7.17 – 7.08 (m, 3H), 4.42 – 4.18 (m, 2H), 2.83 – 2.73 (m, 4H), 2.69 (ddd, *J* = 8.1, 6.2, 1.2 Hz, 2H), 2.41 (s, 3H); **^{13}C-NMR** (101 MHz, $CDCl_3$): δ 150.6, 139.9, 138.4, 133.3, 128.7, 128.6, 126.7, 46.4, 36.4, 34.1, 31.9, 14.6; **IR** (neat): ν 2912, 1275, 1221, 1057, 895, 815, 769 cm^{-1}; **HRMS** (ESI$^+$, m/z): [M+Na]$^+$ calculated for $[C_{14}H_{17}N_3O_2SNa]^+$: 314.0934, found: 314.0935.

11-(4-(2-(2-(methylthio)ethoxy)ethyl)piperazin-1-yl)dibenzo[b,f][1,4]thiazepine (3b)

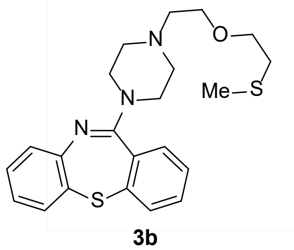

3b

Prepared according to general procedure **C** from quetiapine (115 mg, 0.30 mmol, 1.0 equiv.) and **1f** (82 mg, 0.30 mmol, 1.0 equiv.)**.** Purification on silica gel (heptane/EtOAc = 3:1 to 100% EtOAc). White solid (73 mg, 0.18 mmol, 59% yield).

^{1}H-NMR (400 MHz, $CDCl_3$): δ 7.47 – 7.39 (m, 1H), 7.31 (dd, *J* = 7.7, 1.4 Hz, 1H), 7.27 – 7.18 (m, 3H), 7.09 (ddd, *J* = 8.0, 7.3, 1.5 Hz, 1H), 6.99 (dd, *J* = 8.0, 1.3 Hz, 1H), 6.80 (td, *J* = 7.6, 1.5 Hz, 1H), 3.62 – 3.52 (m, 4H), 3.48 (bs, 4H), 2.66 – 2.41 (m, 8H), 2.06 (s, 3H); **^{13}C-NMR** (101 MHz, $CDCl_3$): δ 160.9, 149.1, 140.1, 134.3, 132.28, 132.25, 130.8, 129.19, 129.12, 128.3, 128.1, 125.4, 122.9, 70.5, 68.8, 58.0, 53.6, 46.9 (bs), 33.7, 16.2; **IR** (neat): ν 2958, 1425, 1235, 789, 755, 668 cm^{-1}; **HRMS** (ESI$^+$, m/z): [M+H]$^+$ calculated for $[C_{22}H_{28}N_3OS_2]^+$: 414.1668, found: 414.1665.

11-(4-(2-(2-(phenethylthio)ethoxy)ethyl)piperazin-1-yl)dibenzo[b,f][1,4]thiazepine (3c)

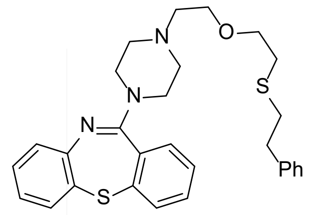

3c

Prepared according to general procedure **C** from quetiapine (115 mg, 0.30 mmol, 1.0 equiv.) and **1b** (95 mg, 0.30 mmol, 1.0 equiv.). Purification on silica gel (heptane/EtOAc = 2:1). White solid (80 mg, 0.16 mmol, 53% yield).

^{1}H-NMR (400 MHz, $CDCl_3$): δ 7.46 – 7.38 (m, 1H), 7.30 (dd, *J* = 7.7, 1.4 Hz, 1H), 7.27 – 7.16 (m, 5H), 7.16 – 7.05 (m, 4H), 6.99 (dd, *J* = 8.0, 1.4 Hz, 1H), 6.79 (td, *J* = 7.5, 1.5 Hz, 1H), 3.62 – 3.34 (m, 8H), 2.84 – 2.78 (m, 2H), 2.77 – 2.70 (m, 2H), 2.64 (td, *J* = 6.9, 2.4 Hz, 2H), 2.59 – 2.41 (m, 6H); **^{13}C-NMR** (101 MHz, $CDCl_3$): δ 160.8, 149.1, 140.6, 140.0, 134.3, 132.26, 132.23, 130.8, 129.17, 129.10, 128.6, 128.3, 128.1, 126.5, 125.4, 122.8, 71.0, 68.8, 58.0, 53.5, 46.8 (bs), 36.5, 34.2, 31.8; **IR** (neat): ν 2959, 2930, 1453, 1264, 755, 748, 698 cm^{-1}; **HRMS** (ESI^+, m/z): $[M+H]^+$ calculated for $[C_{29}H_{34}N_3OS_2]^+$: 504.2138, found: 504.2106.

8-methyl-8-azabicyclo[3.2.1]octan-3-yl 3-(phenethylthio)-2-phenylpropanoate (3d)

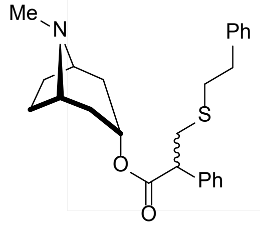

3d

Prepared according to general procedure **C** from atropine (87 mg, 0.30 mmol, 1.0 equiv.) and **1b** (95 mg, 0.30 mmol, 1.0 equiv.). Purification on silica gel (CH_2Cl_2/MeOH = 50:1). Yellow solid (72 mg, 0.18 mmol, 59% yield).

^{1}H-NMR (400 MHz, $CDCl_3$): δ 7.29 – 7.16 (m, 7H), 7.16 – 7.05 (m, 3H), 4.92 (t, *J* = 5.4 Hz, 1H), 3.65 (dd, *J* = 9.2, 6.2 Hz, 1H), 3.18 (dd, *J* = 13.2, 9.2 Hz, 1H), 2.99 – 2.94 (m, 1H), 2.89 – 2.84 (m, 1H), 2.84 – 2.65 (m, 5H), 2.14 (s, 3H), 2.06 – 1.90 (m, 2H), 1.86 – 1.66 (m, 3H), 1.61 (d, *J* = 14.7 Hz, 1H), 1.42 (d, *J* = 14.8 Hz, 1H), 1.37 – 1.28 (m, 1H); **^{13}C-NMR** (101 MHz, $CDCl_3$): δ 171.8, 140.4, 137.9, 128.9, 128.6, 127.85, 127.82, 126.5, 68.4, 59.8, 59.7, 52.9, 40.4, 36.6, 36.4, 36.3, 35.1, 34.3, 25.6, 25.3; **IR** (neat): ν 1726, 1452, 1265, 1208, 1155, 1078, 1026, 749, 728, 698 cm^{-1}; **HRMS** (ESI^+, m/z): $[M+H]^+$ calculated for $[C_{25}H_{32}NO_2S]^+$: 410.2148, found: 410.2153.

5-(4'-((2-butyl-4-chloro-5-((phenethylthio)methyl)-1H-imidazol-1-yl)methyl)-[1,1'-biphenyl]-2-yl)-1-trityl-1*H*-tetrazole (3e)

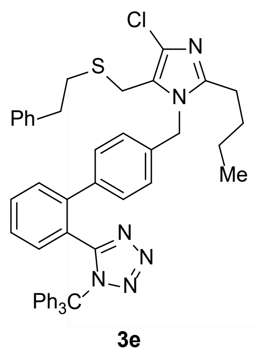

3e

Prepared according to general procedure **C** from trityl losartan (obtained based on literature procedure)[4] (200 mg, 0.30 mmol, 1.0 equiv.) and **1b** (95 mg, 0.30 mmol, 1.0 equiv.). Purification on silica gel (heptane/acetone = 2:8). White foam (187 mg, 0.24 mmol, 79% yield).

^{1}H-NMR (700 MHz, $CDCl_3$): δ 7.96 (dd, *J* = 7.0, 1.4 Hz, 1H), 7.49 (td, *J* = 7.0, 1.4 Hz, 1H), 7.46 (td, *J* = 7.0, 1.4 Hz, 1H), 7.35 – 7.33 (m, 3H), 7.28 – 7.19 (m, 13H), 7.14 (d, *J* = 7.0 Hz, 1H), 7.10 (d, *J* = 8.4 Hz, 2H), 6.90 (d, *J* = 7.0 Hz, 4H), 6.70 (d, *J* = 8.4 Hz, 2H), 5.09 (s, 2H), 3.35 (s, 2H), 2.80 (t, *J* = 7.7 Hz, 2H), 2.64 (t, *J* = 7.7 Hz, 2H), 2.50 (t, *J* = 7.7 Hz, 2H), 1.66 (q, *J* = 7.7 Hz, 2H), 1.29 (sextet, *J* = 7.7 Hz, 2H), 0.86 (t, *J* = 7.7 Hz, 3H). **^{13}C-NMR** (175 MHz, $CDCl_3$): δ 164.0, 148.5, 141.4, 141.2, 140.4, 134.5, 130.9, 130.3, 130.1 (2 carbons), 128.7, 128.6, 128.5, 127.9, 127.8, 126.5, 126.4, 125.2, 121.4, 47.0, 36.3, 32.4, 29.9, 27.0, 24.0, 22.6, 13.9; **IR** (neat) ν 2958, 2930, 2866, 2704, 1604, 1568, 1456, 1413, 1348, 1256, 1151, 1067, 1028, 989, 906, 822, 775, 757, 726 cm^{-1}; **HRMS** (ESI^+, m/z): $[M+H]^+$ calculated for $[C_{49}H_{46}ClN_6S]^+$: 785.3188, found: 785.3185.

(8*S*,9*S*,10*R*,13*S*,14*S*,17*R*)-17-hydroxy-10,13-dimethyl-17-(2-(phenethylthio)acetyl)-7,8,9,10,12,13,14,15,16,17-decahydro-3H-cyclopenta[a]phenanthrene-3,11(6H)-dione (3f)

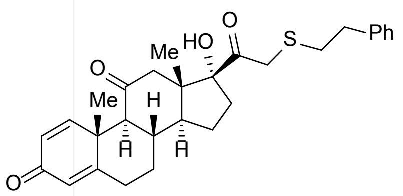

Prepared according to general procedure **C** from prednisone (108 mg, 0.30 mmol, 1.0 equiv.) and **1b** (95 mg, 0.30 mmol, 1.0 equiv.). Purification on silica gel (heptane/acetone = 1:1). White flaky solid (124 mg, 0.26 mmol, 86% yield).

^{1}H-NMR (400 MHz, $CDCl_3$): δ 7.66 (d, *J* = 10.2 Hz, 1H), 7.30 – 7.26 (m, 2H), 7.22 – 7.16 (m, 3H), 6.19 (d, *J* = 10.2 Hz, 1H), 6.07 (br s, 1H), 3.52 – 3.48 (m, 1H), 3.24 – 3.20 (m, 1H), 2.89 – 2.70 (m, 8H), 2.50 (td, *J* = 12.8, 4.4 Hz, 1H), 2.43 – 2.35 (m, 2H), 2.16 – 1.88 (m, 4H), 1.75 – 1.68 (m, 1H), 1.51 – 1.45 (m, 1H), 1.42 (s, 3H), 1.33 – 1.18 (m, 1H), 0.70 (s, 3H); **^{13}C-NMR** (151 MHz, $CDCl_3$): δ 209.3, 205.8, 186.5, 166.6, 155.3, 140.0, 128.7, 128.6, 127.8, 126.6, 124.8, 88.9, 60.2, 51.9, 50.0, 49.6, 38.7, 37.4, 36.3, 35.7, 33.8, 33.4. 32.4, 23.7, 18.9, 16.3; **IR** (neat) ν 3310, 2939, 1703, 1658, 1617, 1512, 1452, 1381, 1329, 1307, 1265, 1239, 1201, 1180, 1149, 1120, 1088, 1065, 1047, 1029, 976, 935, 615, 889, 817, 780, 732 cm^{-1}; **HRMS** (ESI^+, m/z): $[M+H]^+$ calculated for $[C_{29}H_{35}O_4S]^+$: 479.2251 found: 479.2243.

(8*S*,9*S*,10*R*,11*S*,13*S*,14*S*,17*S*)-11-hydroxy-10,13-dimethyl-17-(2-(phenethylthio)acetyl)-1,2,6,7,8,9,10,11,12,13,14,15,16,17-tetradecahydro-3H-cyclopenta[a]phenanthren-3-one (3g)

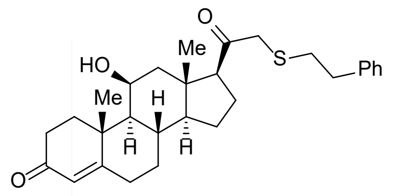

Prepared according to general procedure **C** from corticosterone (104 mg, 0.30 mmol, 1.0 equiv.) and **1b** (95 mg, 0.30 mmol, 1.0 equiv.). Purification on silica gel (heptane/acetone = 1:1). White falky solid (136 mg, 0.29 mmol, 97% yield).

^{1}H-NMR (400 MHz, $CDCl_3$): δ 7.31 – 7.28 (m, 2H), 7.23 – 7.19 (m, 3H), 5.68 (s, 1H), 4.41 – 4.37 (m, 1H), 3.24 – 3.15 (m, 2H), 2.92 – 2.82 (m, 2H), 2.81 – 2.72 (m, 3H), 2.53 – 2.44 (m, 2H), 2.35 (dt, *J* = 16.8, 4.0 Hz, 1H), 2.27 – 2.25 (m, 1H), 2.25 – 2.15 (m, 2H), 2.12 – 2.07 (m, 1H), 2.04 – 1.94 (m, 2H), 1.89 – 1.81 (m, 1H), 1.80 – 1.69 (m, 2H), 1.63 (dd, *J* = 13.6, 3.2 Hz, 1H), 1.44 (s, 3H), 1.41 – 1.34 (m, 1H), 1.16 – 1.08 (m, 2H), 1.06 – 0.98 (m, 2H), 0.93 (s, 3H); **^{13}C-NMR** (101 MHz, $CDCl_3$): δ 205.6, 199.5, 171.9, 128.7 (2xC), 126.6, 122.6, 68.3, 60.5, 57.6, 56.5, 48.3, 41.9, 35.8, 35.2, 34.0, 33.6, 32.7, 32.2, 31.5, 24.6, 23.6, 21.1, 16.3; **IR** (neat) ν 3439, 3409, 2929, 2855, 1701, 1659, 1618, 1497, 1452, 1414, 1389, 1347, 1270, 1232, 1186, 1152, 1119, 1092, 1046, 949, 914, 890, 870, 731 cm^{-1}; **HRMS** (ESI^+, m/z): $[M+Na]^+$ calculated for $[C_{29}H_{38}O_3SNa]^+$: 489.2434, found: 489.2431.

(3*R*,6*R*,8a*S*,9*R*,10*R*,12a*R*)-3,6,9-trimethyl-10-(phenethylthio)decahydro-12*H*-3,12-epoxy[1,2]dioxepino[4,3-i]isochromene (3h)

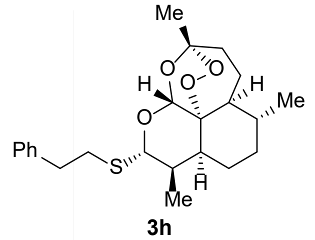

Prepared according to general procedure **C** from dihydroartemisinin (85 mg, 0.30 mmol, 1.0 equiv.) and **1b** (95 mg, 0.30 mmol, 1.0 equiv.). Purification on silica gel (heptane/acetone = 2:8). Colorless oil (75 mg, 0.19 mmol, 62% yield).

Preparation of **3h** was also attempted following general procedure **D** using dihydroartemisinin (58 mg, 0.20 mmol, 1.0 equiv.) and phenethyl mercaptan (27 μL, 0.20 mmol, 1.0 equiv.). ^{1}H NMR of the reaction mixture showed no formation of the desired product.

^{1}H-NMR (700 MHz, $CDCl_3$): δ 7.30 – 7.28 (m, 2H), 7.27 – 7.26 (m, 2H), 7.21 – 7.19 (m, 1H), 5.25 (s, 1H), 4.46 (d, *J* = 10.5 Hz, 1H), 3.10 – 3.04 (m, 2H), 2.99 – 2.94 (m, 1H), 2.89 – 2.85 (m, 1H), 2.65 – 2.60 (m, 1H), 2.37 (td, *J* = 14.7, 4.2 Hz, 1H), 2.03 – 2.00 (m, 1H), 1.90 – 1.86 (m, 1H), 1.72 – 1.67 (m, 2H), 1.60 – 1.57 (m, 1H), 1.51 – 1.44 (m, 1H), 1.42 (s, 3H), 1.35 – 1.22 (m, 3H), 1.05 – 0.99 (m, 1H), 0.95 (d, *J* = 6.3 Hz, 3H), 0.89 (d, *J* = 7.0 Hz, 3H); **^{13}C-NMR** (175 MHz, $CDCl_3$): δ 141.2, 128.9, 128.5, 126.3, 104.4, 92.4, 80.7, 80.6, 51.9, 46.2, 37.5, 37.0, 36.4, 34.2, 31.7, 29.9, 26.1, 24.9, 21.4, 20.4, 15.1; **IR** (neat) ν 2924, 2870, 1452, 1377, 1278, 1229, 1195, 1181, 1151, 1127, 1094, 1085, 1069, 1034, 978, 961, 940, 927, 879, 854, 828, 730 cm^{-1}; **HRMS** (ESI^+, m/z): $[M+Na]^+$ calculated for $[C_{23}H_{32}O_4SNa]^+$: 427.1914, found: 427.1904.

VI. Thioetherification of primary and secondary alcohols

a. Thioether characterization

(4-methoxyphenethyl)(phenethyl)sulfane (4a)

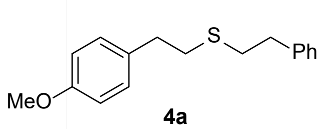

4a

Prepared according to general procedure **C** from 2-(4-methoxyphenyl)ethan-1-ol (46 mg, 0.30 mmol, 1.0 equiv.) and **1b** (95 mg, 0.30 mmol, 1.0 equiv.)**.** Purification on silica gel (heptane/EtOAc = 40:1). Colorless oil (61 mg, 0.22 mmol, 74% yield).

Alternative: The same product can be obtained according to general procedure **C** from 2-phenylethan-1-ol (37 mg, 0.30 mmol, 1.0 equiv.) and **1a** (104 mg, 0.30 mmol, 1.0 equiv.) in a slightly decreased yield (56 mg, 0.22 mmol, 68%).

^{1}H-NMR (400 MHz, $CDCl_3$): δ 7.26 – 7.19 (m, 2H), 7.16 – 7.09 (m, 3H), 7.05 – 7.00 (m, 2H), 6.79 – 6.71 (m, 2H), 3.71 (s, 3H), 2.84 – 2.63 (m, 8H); **^{13}C-NMR** (101 MHz, $CDCl_3$): δ 158.3, 140.7, 132.8, 129.6, 128.6, 128.6, 126.5, 114.0, 55.4, 36.5, 35.6, 34.2, 33.9; **IR** (neat): ν 1511, 1496, 1452, 1243, 1176, 1033, 819, 747, 729, 697 cm^{-1}; **HRMS-**(ESI$^+$, m/z): $[M+Na]^+$ calculated for $[C_{17}H_{20}OSNa]^+$: 295.1127, found: 295.1127.

(4-fluorophenethyl)(phenethyl)sulfane (4b)

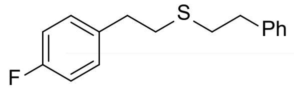

4d

Prepared according to general procedure **C** from 2-(4-fluorophenyl)ethan-1-ol (42 mg, 0.30 mmol, 1.0 equiv.) and **1b** (95 mg, 0.30 mmol, 1.0 equiv.)**.** Purification on silica gel (heptane/EtOAc = 50:1). Colorless oil (53 mg, 0.22 mmol, 67% yield).

Alternative: The same product can be obtained according to general procedure **C** from 2-phenylethan-1-ol (37 mg, 0.30 mmol, 1.0 equiv.) and **1d** (101 mg, 0.30 mmol, 1.0 equiv.) in a lower yield (36 mg, 0.14 mmol, 46%).

^{1}H-NMR (700 MHz, $CDCl_3$) δ 7.31 (t, J = 7.5 Hz, 2H), 7.23 (t, J = 7.4 Hz, 1H), 7.20 (d, J = 7.4 Hz, 2H), 7.14 (dd, J = 8.2, 5.5 Hz, 2H), 6.98 (t, J = 8.6 Hz, 2H), 2.96 – 2.82 (m, 4H), 2.82 – 2.71 (m, 4H); **^{13}C-NMR** (101 MHz, $CDCl_3$): δ 161.7 (d, J = 244.4 Hz), 140.6, 136.3 (d, J = 2.6 Hz), 130.0 (d, J = 7.9 Hz), 128.6 (d, J = 2.5 Hz), 126.5, 115.4 (d, J = 21.1 Hz), 36.5, 35.6, 34.1, 34.0; **^{19}F-NMR** (376 MHz, $CDCl_3$): δ -116.8; **IR** (neat): ν 1509, 1264, 1221, 1158, 823, 733, 700 cm^{-1}, **HRMS** (ESI$^+$, m/z): $[M+H]^+$ calculated for $[C_{16}H_{18}FS]^+$: 261.1108, found: 261.1105.

(*R*)-(3,7-dimethyloct-6-en-1-yl)(phenethyl)sulfane (4c)

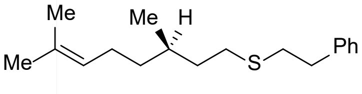

4c

Prepared according to general procedure **C** from (*R*)-*β*-citronellol (47 mg, 0.30 mmol, 1.0 equiv.) and **1b** (95 mg, 0.30 mmol, 1.0 equiv.)**.** Purification on silica gel (heptane/EtOAc = 100:1). Pale yellow oil (62 mg, 0.23 mmol, 75% yield).

^{1}H-NMR (400 MHz, $CDCl_3$): δ 7.32 – 7.27 (m, 2H), 7.24 – 7.17 (m, 3H), 5.14 – 5.05 (m, 1H), 2.89 (dd, J = 9.1, 6.0 Hz, 2H), 2.77 (ddd, J = 8.4, 6.3, 1.1 Hz, 2H), 2.63 – 2.43 (m, 2H), 2.07 – 1.88 (m, 2H), 1.68 (d, J = 1.0 Hz, 3H), 1.66 – 1.62 (m, 1H), 1.60 (s, 3H), 1.60 – 1.54 (m, 1H), 1.46 – 1.28 (m, 2H), 1.16 (dddd, J = 13.5, 9.4, 7.6, 6.1 Hz, 1H), 0.89 (d, J = 6.5 Hz, 3H); **^{13}C-NMR** (101 MHz, $CDCl_3$): δ 140.9, 131.4, 128.62, 128.61, 126.5, 124.9, 36.98, 36.96, 36.6, 33.8, 32.1, 30.2, 25.9, 25.6, 19.4, 17.8; **IR** (neat): 1452, 1264, 748, 698 cm^{-1}; **HRMS** (ESI$^+$, m/z): $[M+H]^+$ calculated for $[C_{18}H_{29}S]^+$: 277.1984, found: 277.1983.

allyl(phenethyl)sulfane (4d)

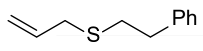

4d

Prepared according to general procedure **C** from prop-2-en-1-ol (17 mg, 0.30 mmol, 1.0 equiv.) and **1b** (95 mg, 0.30 mmol, 1.0 equiv.)**.** Purification on silica gel (heptane/EtOAc = 50:1). Colorless liquid (53 mg, 0.30 mmol, 99% yield).

Alternative: The same product can be obtained according to general procedure **C** from 2-phenylethan-1-ol (37 mg, 0.30 mmol, 1.0 equiv.) and **1e** (76 mg, 0.30 mmol, 1.0 equiv.) in a slightly decreased yield (31 mg, 0.17 mmol, 58%).

^{1}H-NMR (400 MHz, $CDCl_3$): δ 7.36 – 7.28 (m, 2H), 7.26 – 7.19 (m, 3H), 5.82 (ddt, *J* = 16.8, 9.6, 7.2 Hz, 1H), 5.17 – 5.08 (m, 2H), 3.19 – 3.13 (m, 2H), 2.89 (dd, *J* = 9.2, 6.3 Hz, 2H), 2.77 – 2.70 (m, 2H); **^{13}C-NMR** (101 MHz, $CDCl_3$): δ 140.7, 134.6, 128.63, 128.57, 126.4, 117.1, 36.2, 35.0, 32.2; **IR** (neat): v 1263, 764, 749, 703 cm^{-1}; **HRMS** (ESI^+, m/z): $[M+H]^+$ calculated for $[C_{11}H_{15}S]^+$: 179.0889, found: 179.0883.

cinnamyl(phenethyl)sulfane (4e)

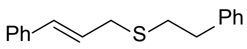

4e

Prepared according to general procedure **C** from (*E*)-3-phenylprop-2-en-1-ol (40 mg, 0.30 mmol, 1.0 equiv.) and **1b** (95 mg, 0.30 mmol, 1.0 equiv.)**.** Purification on silica gel (heptane/EtOAc = 50:1). Colorless liquid (67 mg, 0.26 mmol, 87% yield).

^{1}H-NMR (400 MHz, $CDCl_3$): δ 7.35 – 7.02 (m, 10H), 6.33 (d, *J* = 15.7 Hz, 1H), 6.09 (dt, *J* = 15.6, 7.4 Hz, 1H), 3.21 (dd, *J* = 7.4, 1.1 Hz, 2H), 2.80 (dd, *J* = 9.1, 6.4 Hz, 2H), 2.69 – 2.63 (m, 2H).; **^{13}C-NMR** (101 MHz, $CDCl_3$): δ 140.7, 136.8, 132.3, 128.70, 128.66, 128.57, 127.7, 126.5, 126.4, 126.1, 36.4, 34.6, 32.3; **IR** (neat): v 1263, 749, 732, 699 cm^{-1}; **HRMS** (EI, m/z): $[M]^{\bullet+}$ calculated for $[C_{17}H_{18}S]^{\bullet+}$: 254.1124, found: 254.1122 ($[M]^{\bullet+}$).

(2-methylallyl)(phenethyl)sulfane (4f)

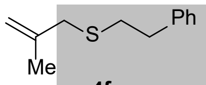

4f

Prepared according to general procedure **C** from 2-methylprop-2-en-1-ol (22 mg, 0.30 mmol, 1.0 equiv.) and **1b** (95 mg, 0.30 mmol, 1.0 equiv.)**.** Purification on silica gel (heptane/EtOAc = 50:1). Colorless liquid (48 mg, 0.26 mmol, 87% yield).

^{1}H-NMR (600 MHz, $CDCl_3$): δ 7.35 – 7.29 (m, 2H), 7.25 – 7.19 (m, 3H), 4.90 – 4.87 (m, 1H), 4.87 – 4.84 (m, 1H), 3.15 (d, *J* = 0.7 Hz, 2H), 2.88 (dd, *J* = 9.2, 6.7 Hz, 2H), 2.70 – 2.66 (m, 2H), 1.86 – 1.81 (m, 3H); **^{13}C-NMR** (150 MHz, $CDCl_3$): δ 141.4, 140.8, 128.62, 128.54, 126.4, 113.7, 39.5, 36.0, 32.3, 20.7; **IR** (neat): v 1276, 1261, 764, 750 cm^{-1}; **HRMS** (ESI^+, m/z): $[M+Na]^+$ calculated for $[C_{12}H_{16}SNa]^+$: 215.0865, found: 215.0871.

phenethyl(prop-2-yn-1-yl)sulfane (4g)

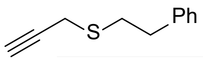

4g

Prepared according to general procedure **C** from prop-2-yn-1-ol (17 mg, 0.30 mmol, 1.0 equiv.) and **1b** (95 mg, 0.30 mmol, 1.0 equiv.)**.** Purification on silica gel (heptane/EtOAc = 50:1). Colorless liquid (48 mg, 0.27 mmol, 91% yield).

^{1}H-NMR (400 MHz, $CDCl_3$): δ 7.26 – 7.19 (m, 2H), 7.17 – 7.10 (m, 3H), 3.17 (d, *J* = 2.6 Hz, 2H), 2.90 – 2.83 (m, 4H), 2.17 (t, *J* = 2.6 Hz, 1H); **^{13}C-NMR** (101 MHz, $CDCl_3$): δ 140.4, 128.7, 128.6, 126.6, 80.0, 71.2, 35.8, 33.1, 19.4; **IR** (neat): v 904, 724, 647 cm^{-1}; **HRMS** (EI, m/z): $[M]^{\bullet+}$ calculated for $[C_{11}H_{12}S]^{\bullet+}$: 176.0654, found: 176.0657.

benzyl(phenethyl)sulfane (4h)

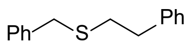

4h

Prepared according to general procedure **C** from benzyl alcohol (32 mg, 0.30 mmol, 1.0 equiv.) and **1b** (95 mg, 0.30 mmol, 1.0 equiv.). Purification on silica gel (heptane/EtOAc = 99:1). Colorless liquid (64 mg, 0.28 mmol, 93% yield).

^{1}H-NMR (400 MHz, $CDCl_3$): δ 7.31 – 7.05 (m, 10H), 3.67 (s, 2H), 2.79 (t, *J* = 2.8 Hz, 2H), 2.61 (t, *J* = 2.8 Hz, 2H). According to literature data.[5]

2-((phenethylthio)methyl)furan (4i)

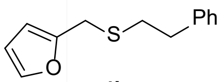

4i

Prepared according to general procedure **C** from furfuryl alcohol (29 mg, 0.30 mmol, 1.0 equiv.) and **1b** (95 mg, 0.30 mmol, 1.0 equiv.). Purification on silica gel (heptane/EtOAc = 50:1). Colorless liquid (49 mg, 0.23 mmol, 75% yield).

^{1}H-NMR (400 MHz, $CDCl_3$) δ 7.39 (dd, *J* = 1.9, 0.8 Hz, 1H), 7.34 – 7.29 (m, 2H), 7.27 – 7.18 (m, 3H), 6.34 (dd, *J* = 3.2, 1.9 Hz, 1H), 6.22 – 6.18 (m, 1H), 3.74 (s, 2H), 2.90 – 2.84 (m, 2H), 2.80 – 2.75 (m, 2H). According to literature data.[5]

2-((phenethylthio)methyl)thiophene (4j)

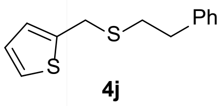

4j

Prepared according to general procedure **C** from thiophen-2-ylmethanol (34 mg, 0.30 mmol, 1.0 equiv.) and **1b** (95 mg, 0.30 mmol, 1.0 equiv.). Purification on silica gel (heptane/EtOAc = 50:1). Colorless liquid (59 mg, 0.25 mmol, 83% yield).

^{1}H-NMR (400 MHz, $CDCl_3$): δ 7.25 – 7.15 (m, 2H), 7.14 – 7.05 (m, 4H), 6.87 – 6.73 (m, 2H), 3.81 (s, 2H), 2.77 (dd, *J* = 9.0, 6.1 Hz, 2H), 2.65 (ddd, *J* = 8.3, 6.4, 1.1 Hz, 2H); **^{13}C-NMR** (101 MHz, $CDCl_3$): δ 142.1, 140.5, 128.62, 128.57, 126.8, 126.5, 126.2, 125.0, 36.1, 33.0, 30.8; **IR** (neat): ν 1263, 1095, 836, 764, 748, 736, 698 cm^{-1}, **HRMS** (ESI^+, m/z): $[M+Na]^+$ calculated for $[C_{13}H_{14}S_2Na]^+$: 257.0429, found: 257.0422.

2-((phenethylthio)methyl)phenol (4k)

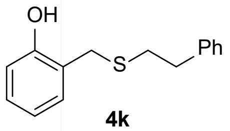

4k

Prepared according to a modified general procedure **C** from 2-(hydroxymethyl)phenol (37 mg, 0.30 mmol, 1.0 equiv.), **1b** (95 mg, 0.30 mmol, 1.0 equiv.) and BTMG (136 µL, 0.66 mmol, 2.2 equiv.) Purification on silica gel (heptane/EtOAc = 10:1). Yellow oil (36 mg, 0.15 mmol, 50% yield).

^{1}H-NMR (400 MHz, $CDCl_3$): δ 7.31 – 7.27 (m, 2H), 7.24 – 7.17 (m, 2H), 7.14 – 7.09 (m, 2H), 7.06 (dd, *J* = 7.4, 1.4 Hz, 1H), 6.92 – 6.84 (m, 2H), 6.51 (s, 1H), 3.79 (s, 2H), 2.84 (dd, *J* = 8.9, 6.6 Hz, 2H), 2.66 (dd, *J* = 8.8, 6.5 Hz, 2H); **^{13}C-NMR** (101 MHz, $CDCl_3$): δ 155.6, 140.2, 130.7, 129.4, 128.7, 126.6, 122.5, 120.8, 117.4, 36.0, 33.0, 32.3; **IR** (neat): ν 1263, 749, 703 cm^{-1}; **HRMS** (ESI^-, m/z): $[M-H]^-$ calculated for $[C_{15}H_{15}OS]^-$: 243.0849, found: 243.0849.

(3-azidopropyl)(phenethyl)sulfane (4l)

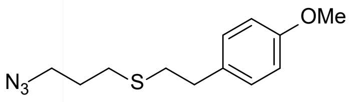

4l

Prepared according to general procedure **C** from 3-azidopropan-1-ol (30 mg, 0.30 mmol, 1.0 equiv.) and **1a** (95 mg, 0.30 mmol, 1.0 equiv.). Purification on silica gel (heptane/EtOAc = 50:1). Pale yellow oil (35 mg, 0.16 mmol, 53% yield).

Preparation of **4l** was also attempted following general procedure **D** using 3-azidopropan-1-ol (20 mg, 0.20 mmol, 1.0 equiv.) and 2-(4-methoxyphenyl)ethanethiol (34 mg, 0.20 mmol, 1.0 equiv.). ^{1}H NMR of the reaction mixture showed no formation of the desired product.

^{1}H-NMR (700 MHz, $CDCl_3$) δ 7.12 (d, J = 8.2 Hz, 2H), 6.85 (d, J = 8.2 Hz, 2H), 3.79 (s, 3H), 3.40 (t, J = 6.6 Hz, 2H), 2.83 (t, J = 7.7 Hz, 2H), 2.75 (t, J = 7.7 Hz, 2H), 2.60 (t, J = 7.1 Hz, 2H), 1.85 (p, J = 6.8 Hz, 2H); **^{13}C-NMR** (176 MHz, $CDCl_3$): δ 158.3, 132.6, 129.6, 114.0, 55.4, 50.2, 35.5, 34.2, 29.3, 28.9; **IR** (neat): ν 2932, 2092, 1510, 1243, 1176, 1033, 819 cm^{-1}; **HRMS** (ESI^+, m/z): $[M+Na]^+$ calculated for $[C_{12}H_{17}N_3SONa]^+$: 274.0985, found: 274.0981.

***tert*-butyldimethyl(4-(phenethylthio)butoxy)silane (4m)**

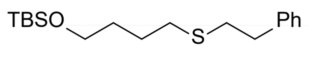

4m

Prepared according to general procedure **C** from 4-((*tert*-butyldimethylsilyl)oxy)butan-1-ol (obtained based on literature procedure)[6] (61 mg, 0.30 mmol, 1.0 equiv.) and **1b** (95 mg, 0.30 mmol, 1.0 equiv.). Purification on silica gel (heptane/EtOAc = 50:1). Colorless liquid (70.5 mg, 0.22 mmol, 72% yield).

^{1}H-NMR (400 MHz, $CDCl_3$) δ 7.34 – 7.27 (m, 2H), 7.25 – 7.14 (m, 3H), 3.62 (dd, *J* = 7.1, 5.0 Hz, 2H), 2.89 (dd, *J* = 8.8, 6.2 Hz, 2H), 2.82 – 2.73 (m, 2H), 2.60 – 2.50 (m, 2H), 1.72 – 1.55 (m, 4H), 0.90 (s, 9H), 0.05 (s, 6H); **^{13}C-NMR** (101 MHz, $CDCl_3$): δ 140.9, 128.62, 128.60, 126.5, 62.8, 36.6, 33.7, 32.3, 32.2, 26.2, 26.1, 18.5; **IR** (neat): ν 1263, 1030, 750, 698 cm^{-1}; **HRMS** (ESI^+, m/z): $[M+Na]^+$ calculated for $[C_{18}H_{32}OSiSNa]^+$: 347.1835, found: 347.1834.

phenethyl(1-phenylethyl)sulfane (4n)

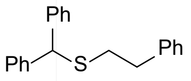

4n

Prepared according to general procedure **C** from diphenylmethanol (55 mg, 0.30 mmol, 1.0 equiv.) and **1b** (95 mg, 0.30 mmol, 1.0 equiv.). Purification on silica gel (heptane/EtOAc = 50:1). Colorless oil (64 mg, 0.22 mmol, 74% yield).

^{1}H-NMR (400 MHz, $CDCl_3$): δ 7.32 (d, *J* = 7.2 Hz, 4H), 7.27 – 7.09 (m, 9H), 7.03 (d, *J* = 7.1 Hz, 2H), 5.03 (s, 1H), 2.79 – 2.73 (m, 2H), 2.59 – 2.52 (m, 2H); **^{13}C-NMR** (101 MHz, $CDCl_3$): δ = 141.5, 140.6, 128.68, 128.68, 128.55, 128.47, 127.3, 126.4, 54.4, 36.1, 33.9; **IR** (neat): ν 1291, 1155, 802, 784, 733 cm^{-1}; **HRMS** (ESI^+, m/z): $[M+Na]^+$ calculated for $[C_{21}H_{20}SNa]^+$: 327.1178, found: 327.1179.

(4-methoxyphenethyl)(4-methyl-1-phenylpent-1-yn-3-yl)sulfane (4o)

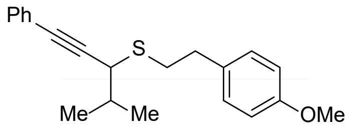

4o

Prepared according to general procedure **C** from 4-methyl-1-phenylpent-1-yn-3-ol (35 mg, 0.20 mmol, 1.0 equiv.) and **1a** (95 mg, 0.20 mmol, 1.0 equiv.). Purification on silica gel (heptane/EtOAc = 50:1). Pale yellow oil (48 mg, 0.15 mmol, 74% yield).

^{1}H-NMR (400 MHz, $CDCl_3$): δ 7.48 – 7.40 (m, 2H), 7.34 – 7.27 (m, 3H), 7.15 (d, *J* = 8.6 Hz, 2H), 6.87 – 6.81 (m, 2H), 3.79 (s, 3H), 3.69 (d, *J* = 5.4 Hz, 1H), 3.09 – 2.84 (m, 4H), 2.04 (dq, *J* = 13.3, 6.7 Hz, 1H), 1.15 (dd, *J* = 8.8, 6.7 Hz, 6H).; **^{13}C-NMR** (101 MHz, $CDCl_3$): δ 158.3, 132.9, 131.8, 129.6, 128.4, 128.1, 123.4, 114.0, 87.9, 85.1, 55.4, 43.1, 35.6, 33.6, 33.1, 21.2, 19.4; **IR** (neat): ν 2957, 1611, 1511, 1442, 1300, 1245, 1176, 1034, 819, 755, 690 cm^{-1}; **HRMS** (ESI^+, m/z): $[M+Na]^+$ calculated for $[C_{21}H_{24}OSNa]^+$: 347.1440, found: 347.1423.

2-(3-((4-phenylbutan-2-yl)thio)propyl)isoindoline-1,3-dione (4p)

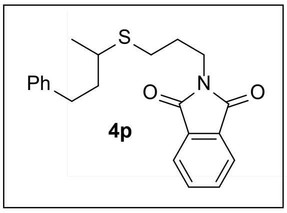

Prepared according to general procedure **C** from 4-phenylbutan-2-ol (30 mg, 0.20 mmol, 1.0 equiv.) and **1j** (80 mg, 0.20 mmol, 1.0 equiv.). Purification on silica gel (heptane/EtOAc = 25:2). Pale yellow oil (23.5 mg, 0.7 mmol, 33% yield). **^{1}H-NMR** (600 MHz, $CDCl_3$): δ 7.87 – 7.83 (m, 2H), 7.74 – 7.69 (m, 2H), 7.28 – 7.24 (m, 2H), 7.19 – 7.14 (m, 3H), 3.79 (t, *J* = 7.1 Hz, 2H), 2.78 – 2.69 (m, 3H), 2.56 (t, *J* = 7.4 Hz, 2H), 1.98 – 1.92 (m, 2H), 1.89 – 1.74 (m, 2H), 1.30 (d, *J* = 6.8 Hz, 3H); **^{13}C-NMR** (151 MHz, $CDCl_3$): δ 168.5, 142.0, 134.1, 132.3, 128.6, 128.5, 126.0, 123.4, 39.6, 38.7, 37.5, 33.3, 28.9, 27.7, 21.7; **IR** (neat): v 3001, 1704, 1644, 1611, 1538, 1413, 1296, 1277, 844, 780, 681 cm^{-1}; **HRMS** (ESI^+, m/z): $[M+Na]^+$ calculated for $[C_{21}H_{23}NO_2SNa]^+$: 376.1342, found: 376.1340.

methyl(phenethyl)sulfane (4q)

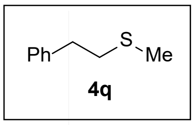

Prepared according to general procedure **C** from 2-phenylethyl alcohol (37 mg, 0.30 mmol, 1.0 equiv.) and **1f** (82 mg, 0.30 mmol, 1.0 equiv.). Purification on silica gel (heptane/EtOAc = 50:1). Colorless oil (64 mg, 0.22 mmol, 73% yield).

^{1}H NMR (400 MHz, $CDCl_3$) δ 7.34 – 7.28 (m, 2H), 7.22 (dd, *J* = 7.1, 4.9 Hz, 2H), 2.91 (dd, *J* = 9.1, 6.4 Hz, 2H), 2.78 – 2.73 (m, 2H), 2.13 (s, 3H). According to literature data.[7]

isopropyl(phenethyl)sulfane (4r)

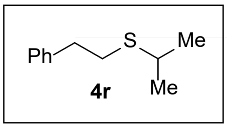

Prepared according to general procedure **C** from 2-phenylethyl alcohol (37 mg, 0.30 mmol, 1.0 equiv.) and **1g** (77 mg, 0.30 mmol, 1.0 equiv.). Purification on silica gel (heptane/EtOAc = 50:1). Colorless oil (40 mg, 0.22 mmol, 74% yield).

^{1}H NMR (400 MHz, $CDCl_3$) δ 7.34 – 7.28 (m, 2H), 7.25 – 7.19 (m, 2H), 2.97 (hept, *J* = 6.7 Hz, 1H), 2.90 – 2.85 (m, 2H), 2.84 – 2.77 (m, 2H), 1.29 (d, *J* = 6.7 Hz, 6 H). According to literature data.[8]

but-2-yn-1-yl(phenethyl)sulfane (4s)

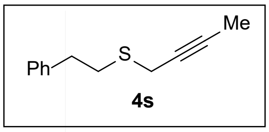

Prepared according to general procedure **C** from 2-phenylethyl alcohol (37 mg, 0.30 mmol, 1.0 equiv.) and **1h** (80 mg, 0.30 mmol, 1.0 equiv.). Purification on silica gel (heptane/EtOAc = 50:1). Pale yellow oil (42 mg, 0.22 mmol, 73% yield). **^{1}H-NMR** (400 MHz, $CDCl_3$): δ 7.26 – 7.20 (m, 2H), 7.18 – 7.10 (m, 3H), 3.16 (q, *J* = 2.5 Hz, 2H), 2.85 (s, 4H), 1.76 (dd, *J* = 3.3, 1.7 Hz, 3H). According to literature data.[9]

cyclohex-2-en-1-yl(4-methoxyphenethyl)sulfane (4t)

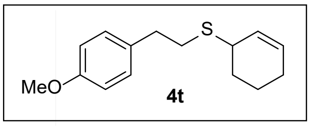

Prepared according to general procedure **C** from 2-(4-methoxyphenyl)ethan-1-ol (46 mg, 0.30 mmol, 1.0 equiv.) and **1i** (88 mg, 0.30 mmol, 1.0 equiv.). Purification on silica gel (heptane/CH_2Cl_2 = 70:30). Colorless liquid (69 mg, 0.24 mmol, 80% yield).

^{1}H NMR (400 MHz, $CDCl_3$) δ 7.17 – 7.10 (m, 2H), 6.93 – 6.78 (m, 2H), 5.84 – 5.74 (m, 1H), 5.73 – 5.67 (m, 1H), 3.79 (s, 2H), 3.44 – 3.31 (m, 1H), 2.87 – 2.74 (m, 4H), 2.03 – 1.91 (m, 3H), 1.91 – 1.80 (m, 1H), 1.79 – 1.70 (m, 1H), 1.64 – 1.54 (m, 1H); **^{13}C NMR** (101 MHz, $CDCl_3$) δ 158.3, 133.0, 129.8, 129.5, 128.0, 114.0, 55.4, 41.0, 35.9, 32.9, 29.7, 25.0, 20.1; **IR** (neat) v 3024, 2927, 2833, 1610, 1586, 1510, 1441, 1300, 1243, 1176, 1102, 1034, 871, 819, 751, 722, 634 cm^{-1}; **HRMS** (ESI^+, m/z): $[M+Na]^+$calculated for $[C_{15}H_{20}OSNa]^+$: 271.1127, found: 271.1130.

but-2-yn-1-yl(cyclohex-2-en-1-yl)sulfane (4u)

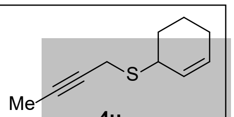

4u

Prepared according to general procedure **C** from 2-butyn-1-ol (21 mg, 0.30 mmol, 1.0 equiv.) and **1i** (88 mg, 0.30 mmol, 1.0 equiv.). Purification on silica gel (heptane/EtOAc = 50:1). Yellow liquid (44 mg, 0.27 mmol, 89% yield).

^{1}H-NMR (400 MHz, $CDCl_3$) δ 5.85 – 5.77 (m, 1H), 5.71 (ddt, *J* = 9.9, 3.7, 1.9 Hz, 1H), 3.63 – 3.54 (m, 1H), 3.24 (qq, *J* = 16.5, 2.5 Hz, 2H), 2.05 – 1.91 (m, 3H), 1.86 (ddd, *J* = 12.5, 6.3, 3.1 Hz, 1H), 1.82 (t, *J* = 2.5 Hz, 3H), 1.81 – 1.75 (m, 1H), 1.67 – 1.54 (m, 1H); **^{13}C-NMR** (101 MHz, $CDCl_3$): δ 130.3, 127.2, 78.8, 75.4, 40.5, 28.9, 25.1, 19.8, 19.2, 3.8; **IR** (neat): v 1264, 733, 698 $cm^{-1;}$ **HRMS** (EI, m/z): $[M]^{\bullet+}$ calculated for $[C_{10}H_{14}S]^{\bullet+}$: 166.0811, found: 166.0812.

allyl(cinnamyl)sulfane (4v)

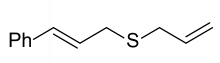

4v

Prepared according to general procedure **C** from (*E*)-3-phenylprop-2-en-1-ol (40 mg, 0.30 mmol, 1.0 equiv.) and **1e** (76 mg, 0.30 mmol, 1.0 equiv.). Purification on silica gel (heptane/EtOAc = 50:1). Colorless liquid (40 mg, 0.21 mmol, 70% yield).

^{1}H-NMR (400 MHz, $CDCl_3$) δ 7.38 (dd, *J* = 5.3, 3.4 Hz, 2H), 7.35 – 7.29 (m, 2H), 7.27 – 7.19 (m, 1H), 6.43 (d, *J* = 15.7 Hz, 1H), 6.17 (dt, *J* = 15.7, 7.3 Hz, 1H), 5.88 – 5.73 (m, 1H), 5.15 (s, 1H), 5.13 – 5.10 (m, 1H), 3.28 (dd, *J* = 7.3, 1.2 Hz, 2H), 3.14 (dt, *J* = 7.1, 1.0 Hz, 2H); **^{13}C-NMR** (101 MHz, $CDCl_3$): δ 136.9, 134.5, 132.6, 128.7, 127.7, 126.4, 126.0, 117.4, 33.7, 33.2; **IR** (neat): v 1276, 1261, 764, 751 cm^{-1}; **HRMS** (EI, m/z): $[M]^{\bullet+}$ calculated for $[C_{12}H_{14}S]^{\bullet+}$: 190.0811, found: 190.0815.

ethyl 3,4-bis(phenethylthio)butanoate (4w)

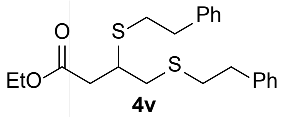

4v

Prepared according to general procedure **C** from ethyl (E)-4-hydroxybut-2-enoate (obtained based on literature procedure)[10] (**5c**, 39 mg, 0.30 mmol, 1.0 equiv.) and **1b** (95 mg, 0.30 mmol, 1.0 equiv.). Purification on silica gel (heptane/EtOAc = 97:3). Colorless liquid (41 mg, 0.11 mmol, 70% yield).

^{1}H-NMR (600 MHz, $CDCl_3$) δ 7.29 (t, J = 7.5 Hz, 4H), 7.24 – 7.18 (m, 6H), 4.16 (q, J = 7.1 Hz, 2H), 3.27 – 3.18 (m, 1H), 2.94 – 2.86 (m, 6H), 2.86 – 2.77 (m, 4H), 2.74 – 2.65 (m, 1H), 2.51 (dd, J = 16.0, 8.1 Hz, 1H), 1.26 (t, J = 7.1 Hz, 3H); **^{13}C-NMR** (151 MHz, $CDCl_3$): δ 171.6, 140.45, 140.43, 128.65 (6C), 128.63 (2C), 126.58 (2C), 60.9, 41.9, 39.3, 38.2, 36.50, 36.45, 34.5, 33.1, 14.4; **IR** (neat): v 2914, 1729, 1204, 1136, 1030, 731, 696 cm^{-1}; **HRMS** (EI, m/z): $[M+Na]^+$ calculated for $[C_{22}H_{28}O_2S_2Na]^+$: 411.1423, found: 411.1422.

1,6-bis(phenethylthio)hexane (6)

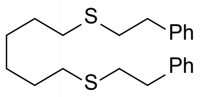

6

Prepared following general procedure **C** from 6-bromohexan-1-ol (36 mg, 0.2 mmol, 1.0 equiv.) and **1b** (64 mg, 0.2 mmol, 1.0 equiv). Purification on silica gel (heptane/EtOAc = 50:1). Colorless oil (11 mg, 0.03 mmol, 32% yield).

^{1}H-NMR (400 MHz, $CDCl_3$) δ 7.33 – 7.27 (m, 4H), 7.21 (t, *J* = 6.6 Hz, 6H), 2.88 (dd, *J* = 9.2, 6.2 Hz, 4H), 2.81 – 2.71 (m, 4H), 2.58 – 2.46 (m, 4H), 1.64 – 1.55 (m, 4H), 1.44 – 1.33 (m, 4H); **^{13}C-NMR** (101 MHz, $CDCl_3$): δ 140.8, 128.6(4C), 126.5, 36.6, 33.8, 32.4, 29.7, 28.6; **IR** (neat): v 3085, 3062, 3026, 3001, 2925, 2854, 1603, 1496, 1453, 1305, 1266, 1253, 1223, 1030, 739, 698 cm^{-1}; **HRMS** (EI, m/z): $[M+Na]^+$ calculated for $[C_{22}H_{30}S_2Na]^+$: 381.1681, found: 381.1675.

b. Synthesis of 4a under open-to-air conditions.

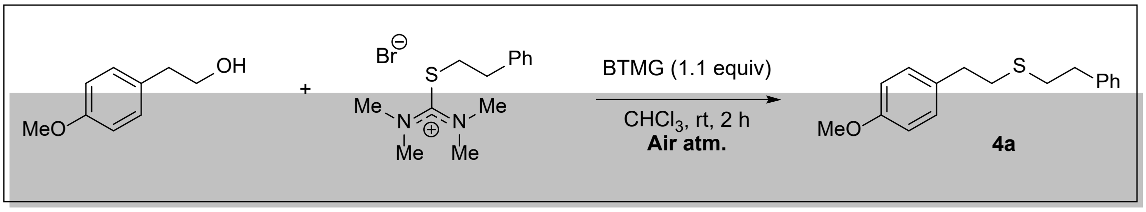

BTMG (2-t*ert*-butyl-1,1,3,3-tetramethylguanidine, Barton's base) (68 μL, 0.33 mmol, 1.1 equiv.) was added dropwise to a solution of 2-(4-methoxyphenyl)ethan-1-ol (46 mg, 0.30 mmol, 1.0 equiv.) and 1,1,3,3-tetramethyl-2-phenethylisothiouronium bromide (95 mg, 0.30 mmol, 1.0 equiv.) in dry $CHCl_3$ (2.2 mL) under air atmosphere (balloon filled with air on the septum cap). The reaction mixture was then stirred at rt for 2 h, before excess base was quenched by addition of a saturated solution of NH_4Cl. The mixture was then extracted with CH_2Cl_2 (3x), the combined organic phases were dried over $MgSO_4$, filtered and concentrated under reduced pressure. The residue was purified by column chromatography (heptane/EtOAc = 40:1) to provide **4a** as a colorless oil (61 mg, 0.22 mmol, 74% yield).

c. Gram-scale synthesis of 4a.

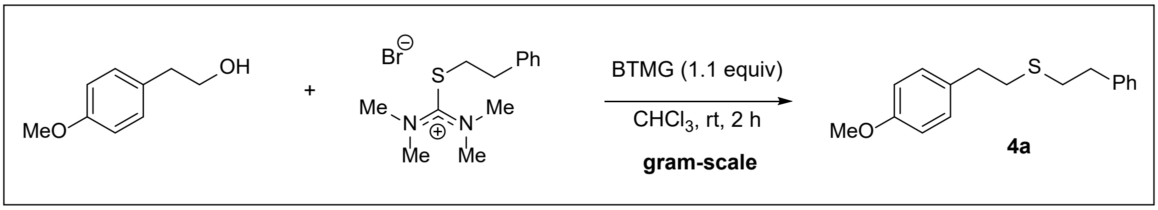

BTMG (2-*tert*-butyl-1,1,3,3-tetramethylguanidine, Barton's base) (1.5 mL, 7.2 mmol, 1.1 equiv.) was added dropwise to a solution of 2-(4-methoxyphenyl)ethan-1-ol (1.0 g, 6.6 mmol, 1.0 equiv.) and 1,1,3,3-tetramethyl-2-phenethylisothiouronium bromide (2.1 g, 6.6 mmol, 1.0 equiv.) in dry $CHCl_3$ (44 mL) under an Ar atmosphere. The reaction mixture was then stirred at rt for 2 h, before excess base was quenched by addition of a saturated solution of NH_4Cl (75 mL). The mixture was then extracted with CH_2Cl_2 (3x), the combined organic phases were dried over $MgSO_4$, filtered and concentrated under reduced pressure. The residue was purified by column chromatography (heptane/EtOAc = 40:1) to provide **4a** as a colorless oil (1.37 g, 5.0 mmol, 74% yield).

VII. References

[1] S. Tong, M. Zhang, S. Wang, R. Yin, R. Yu, S. Wan, T. Jiang and L. Zhang, *Eur. J. Med. Chem.*, 2016, **123**, 849.

[2] S. S. Khokhar and T. Wirth, *Eur. J. Org. Chem.*, 2004, 4567.

[3] Q. Yao and Y. Zhang, *J. Am. Chem. Soc.*, 2004, **126**, 74.

[4] T. Hadizad, J. Collins, R. E. Antoun, R. S. Beanlands and J. N. DaSilva, *J. Label Compd. Radiopharm.,* 2011, **54**, 754.

[5] D. Limnios and C. G. Kokotos, *Adv. Synth. Catal.*, 2017, **359**, 323.

[6] A. Throup, L. H. Patterson and H. M. Sheldrake, Org. Biomol. Chem., 2016, **14**, 9554.

[7] S. K. Kristensen, S. L. R. Laursen, E. Taarning and T. Skrydstrup, *Angew. Chem. Int. Ed.*, 2018, **57**, 13887.

[8] R. S. Glass, J. L. Boecker and M. E. Jatcko, *Tetrahedron*, 1989, **45**, 1263.

[9] C. S. Holzwarth, I. Alt and B. Pletker, *Angew. Chem. Int. Ed.*, 2012, **51**, 5351.

[10] L. Hutchings-Goetz, C. Yang and T. N. Snaddon, ACS Catal., 2018, **8**, 10537.

VIII. NMR spectra

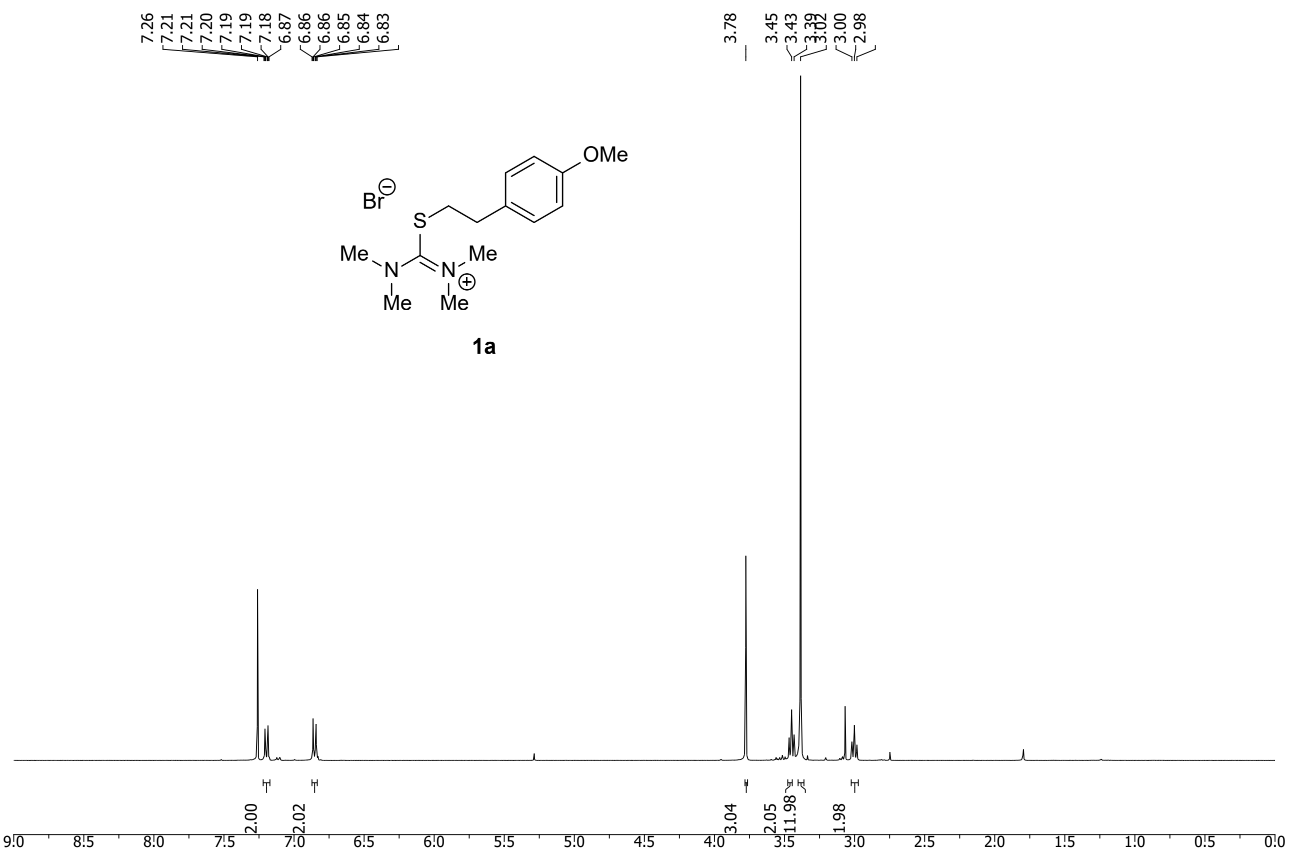

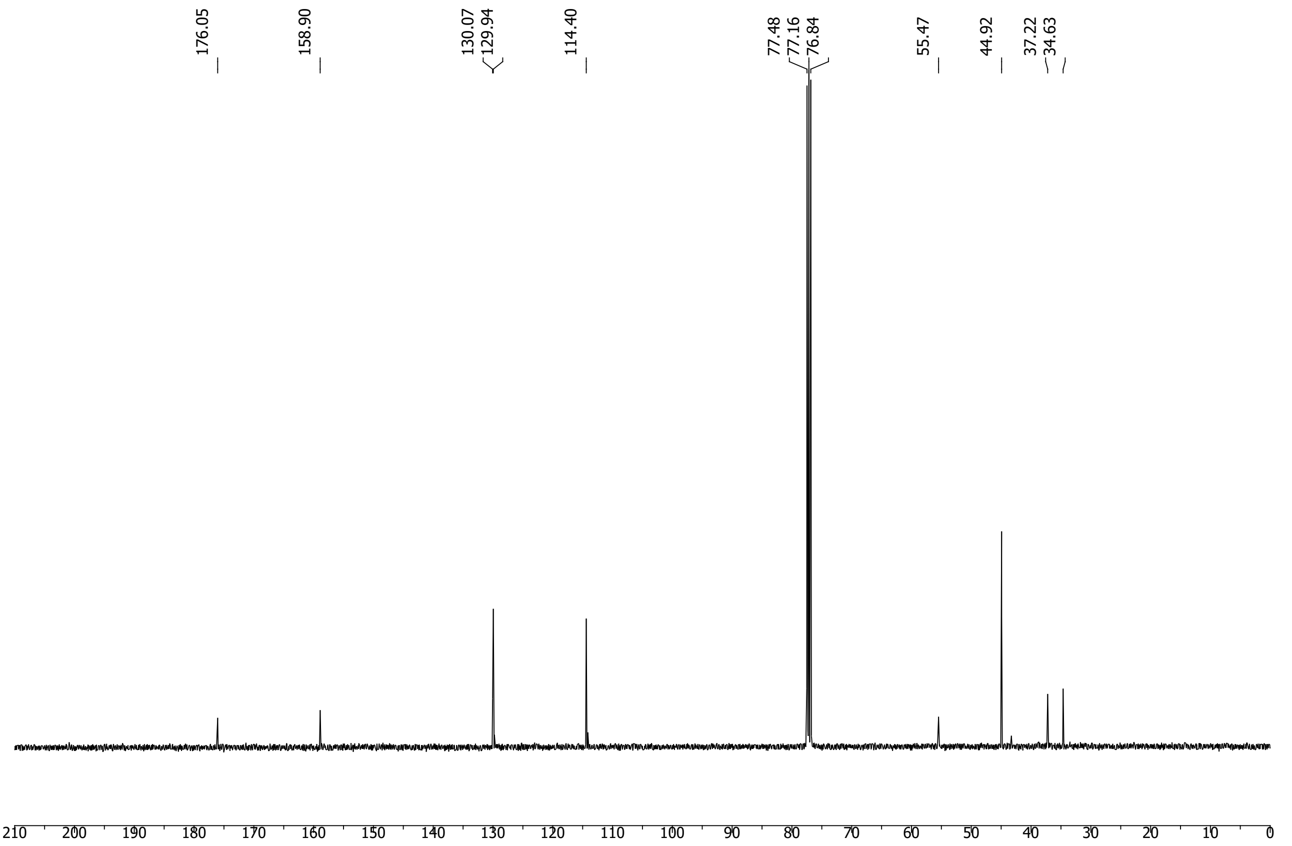

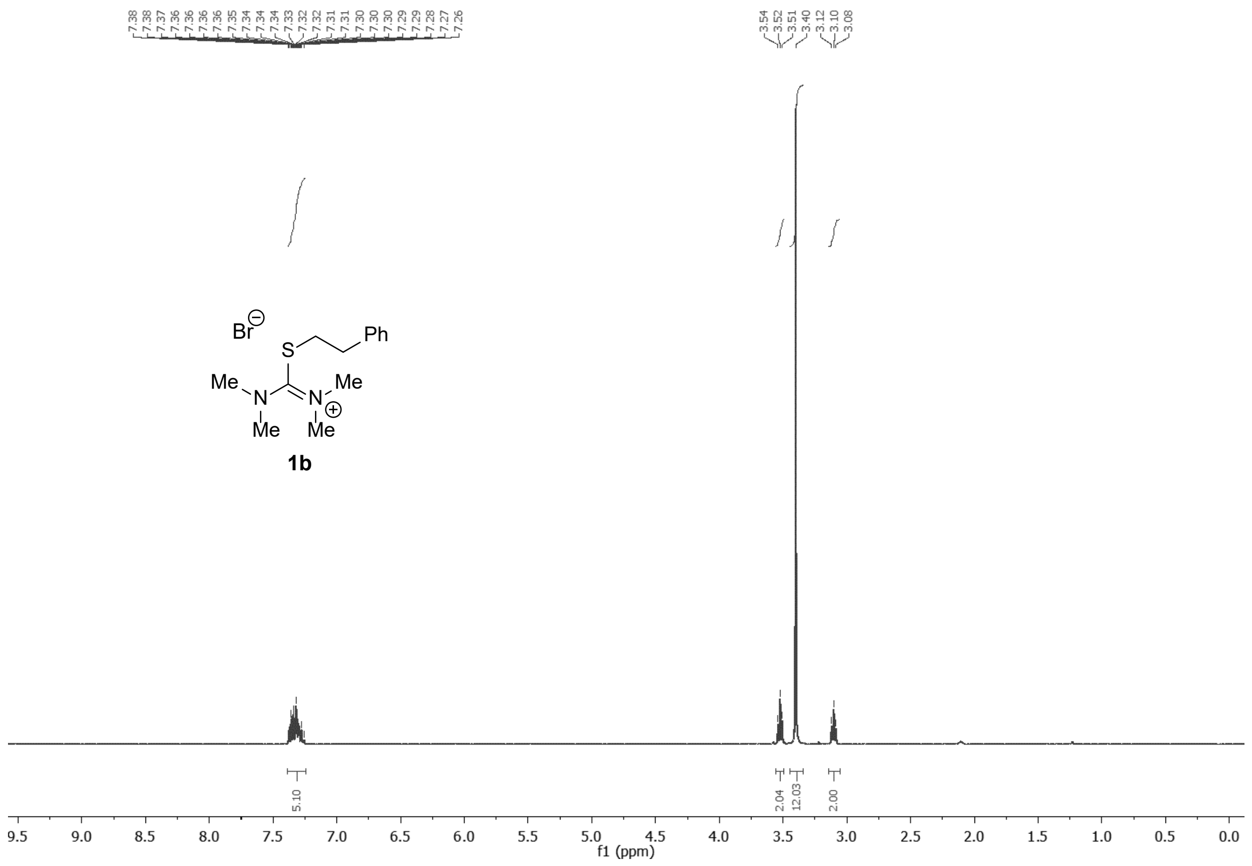
7.38 7.38 7.37 7.36 7.36 7.36 7.35 7.34 7.34 7.34 7.33 7.32 7.32 7.31 7.31 7.30 7.30 7.30 7.29 7.29 7.28 7.27 7.26
3.54 3.52 3.51 3.40 3.12 3.10 3.08
Br
S
Ph
Me
N
N
Me
Me
Me
1b
5.10
2.04
12.03
2.00
9.5 9.0 8.5 8.0 7.5 7.0 6.5 6.0 5.5 5.0 4.5 4.0 3.5 3.0 2.5 2.0 1.5 1.0 0.5 0.0
f1 (ppm)

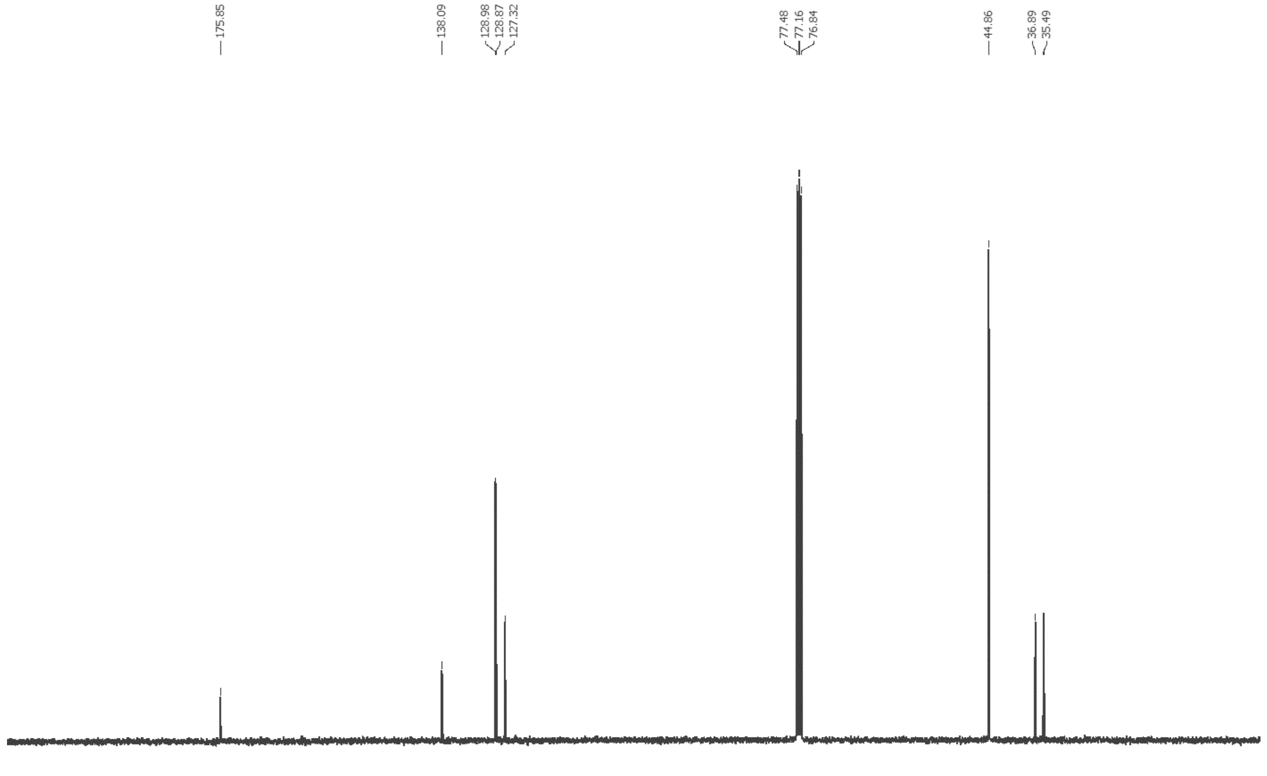
175.85
138.09
128.98 128.87 127.32
77.48 77.16 76.84
44.86
36.89 35.49
210 200 190 180 170 160 150 140 130 120 110 100 90 80 70 60 50 40 30 20 10 0
f1 (ppm)

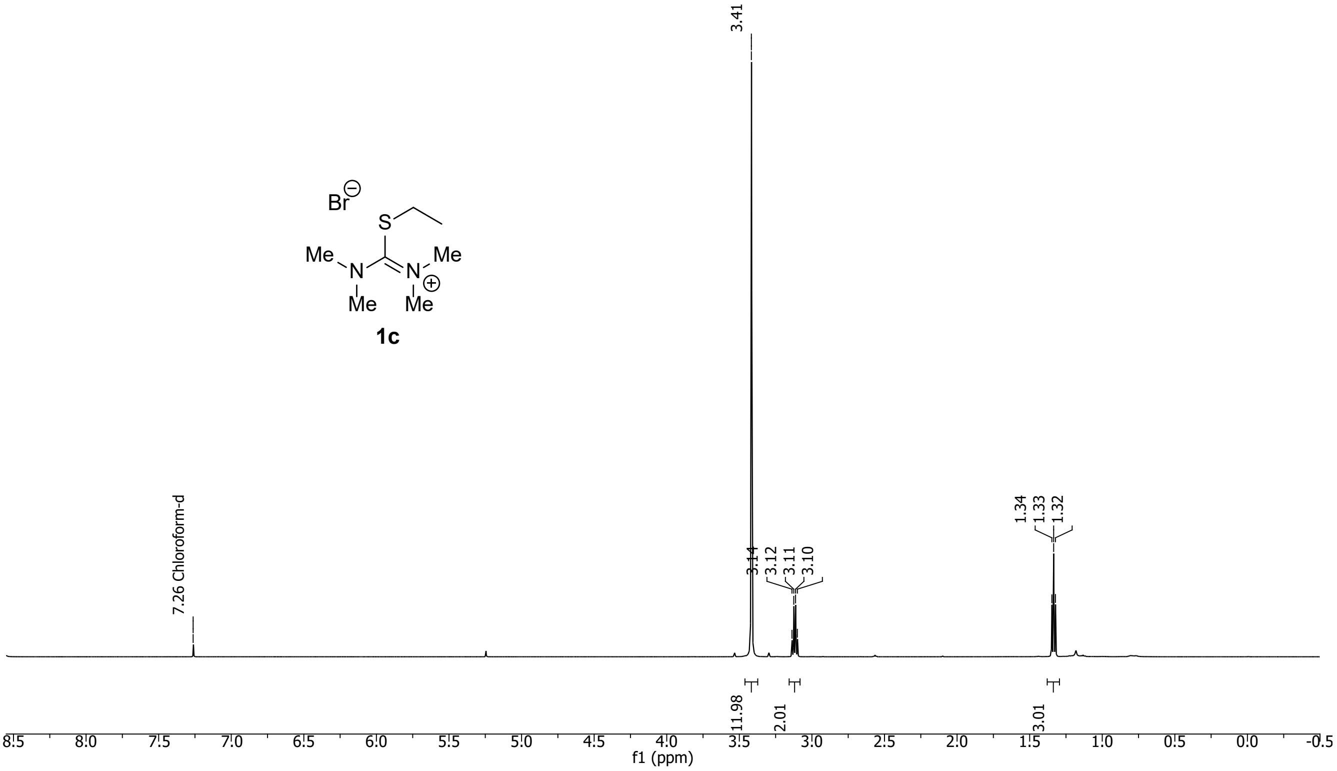
3.41
7.26 Chloroform-d
3.14
3.12
3.11
3.10
1.34
1.33
1.32
11.98
2.01
3.01
8.5 8.0 7.5 7.0 6.5 6.0 5.5 5.0 4.5 4.0 3.5 3.0 2.5 2.0 1.5 1.0 0.5 0.0 -0.5
f1 (ppm)
Br
S
Me
N
N
Me
Me
Me
1c

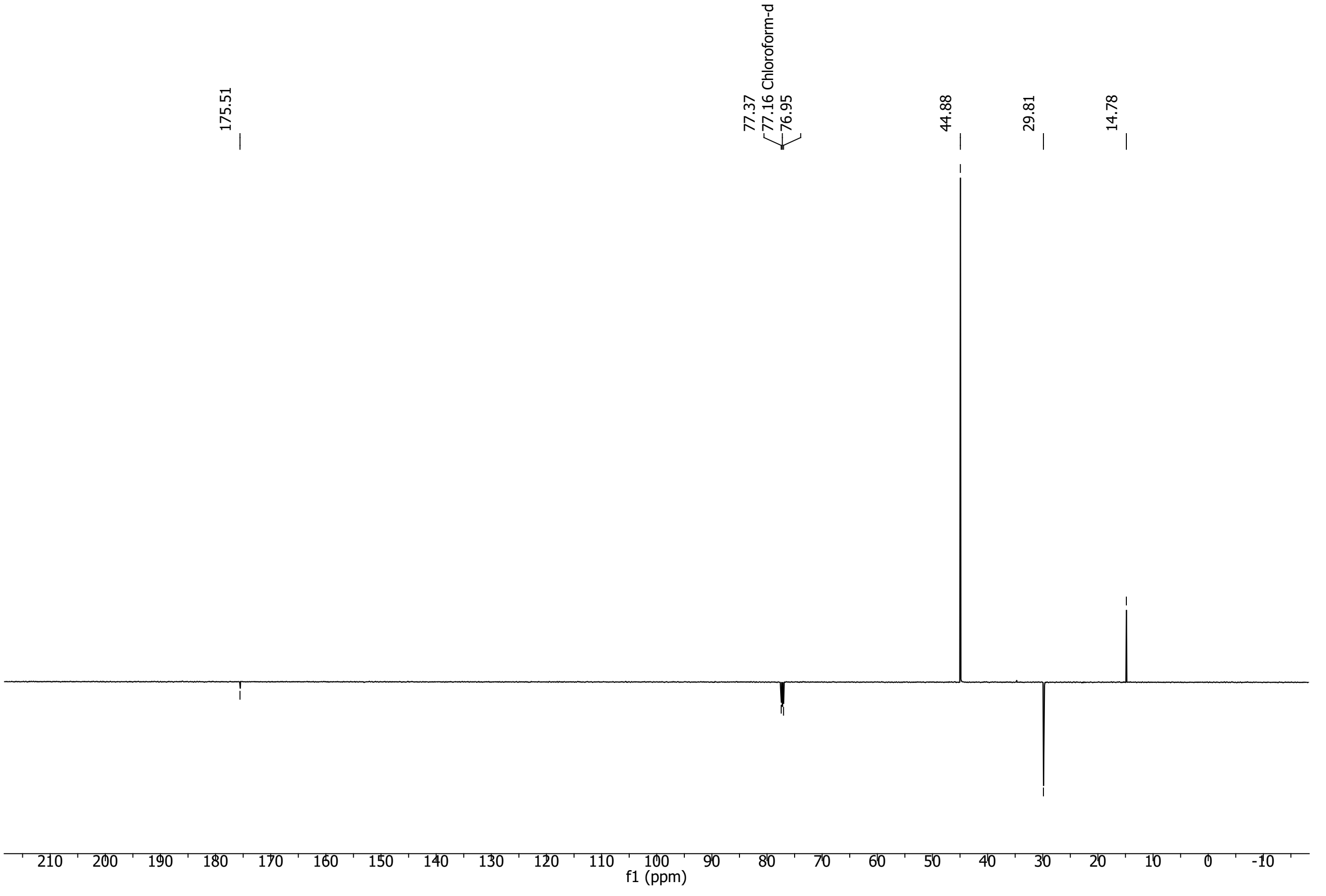
175.51
77.37
77.16 Chloroform-d
76.95
44.88
29.81
14.78
210 200 190 180 170 160 150 140 130 120 110 100 90 80 70 60 50 40 30 20 10 0 -10
f1 (ppm)

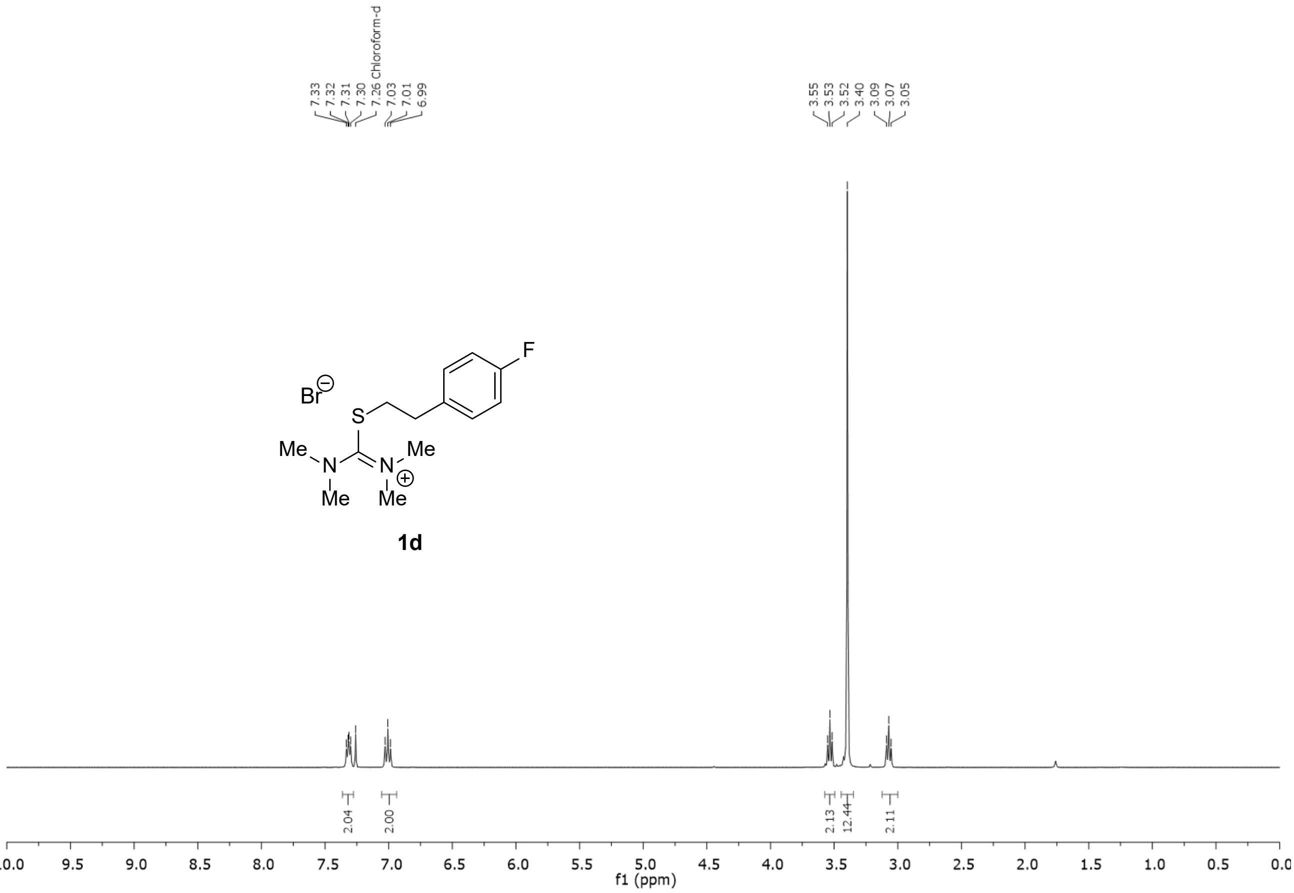
7.33
7.32
7.31
7.30
7.26 Chloroform-d
7.03
7.01
6.99
3.55
3.53
3.52
3.40
3.09
3.07
3.05
F
Br⊖
S
Me
N
N⊕
Me
Me
Me
1d
2.04
2.00
2.13
12.44
2.11
10.0 9.5 9.0 8.5 8.0 7.5 7.0 6.5 6.0 5.5 5.0 4.5 4.0 3.5 3.0 2.5 2.0 1.5 1.0 0.5 0.0
f1 (ppm)

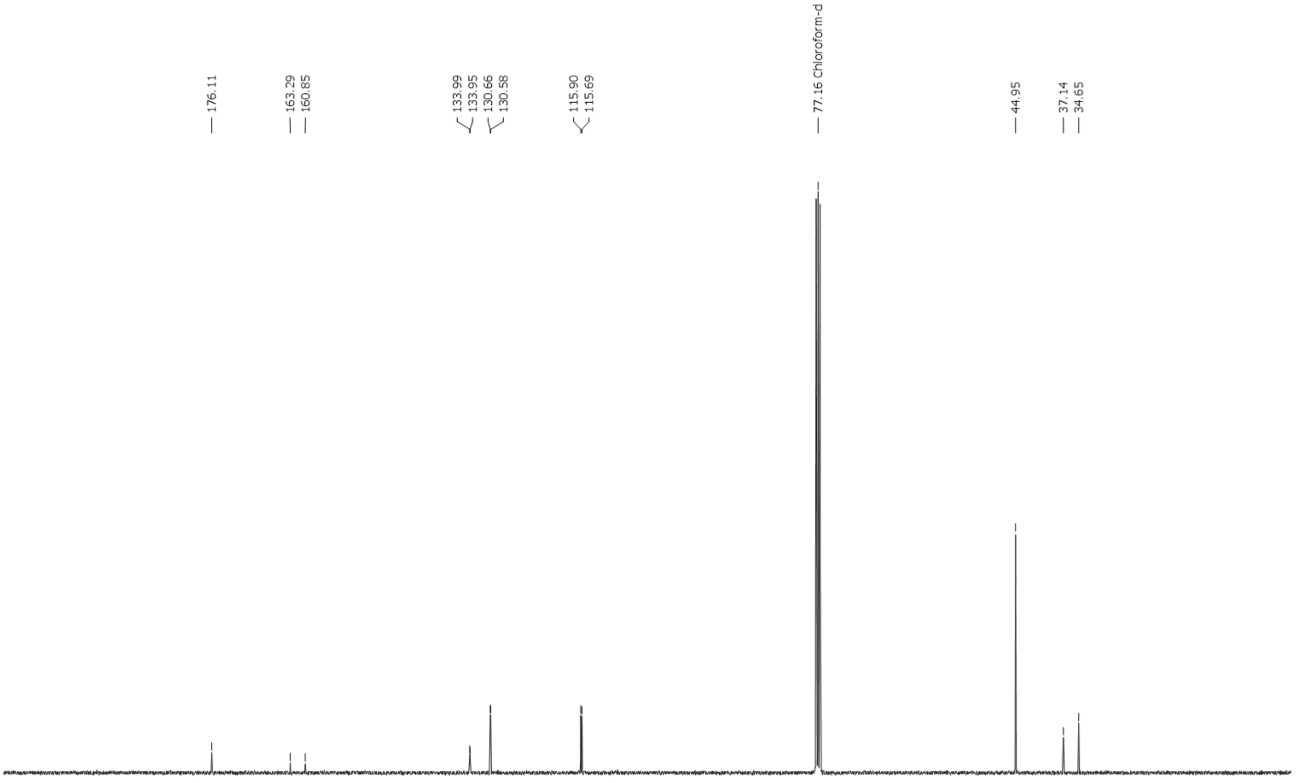
176.11
163.29
160.85
133.99
133.95
130.66
130.58
115.90
115.69
77.16 Chloroform-d
44.95
37.14
34.65
210 200 190 180 170 160 150 140 130 120 110 100 90 80 70 60 50 40 30 20 10 0
f1 (ppm)

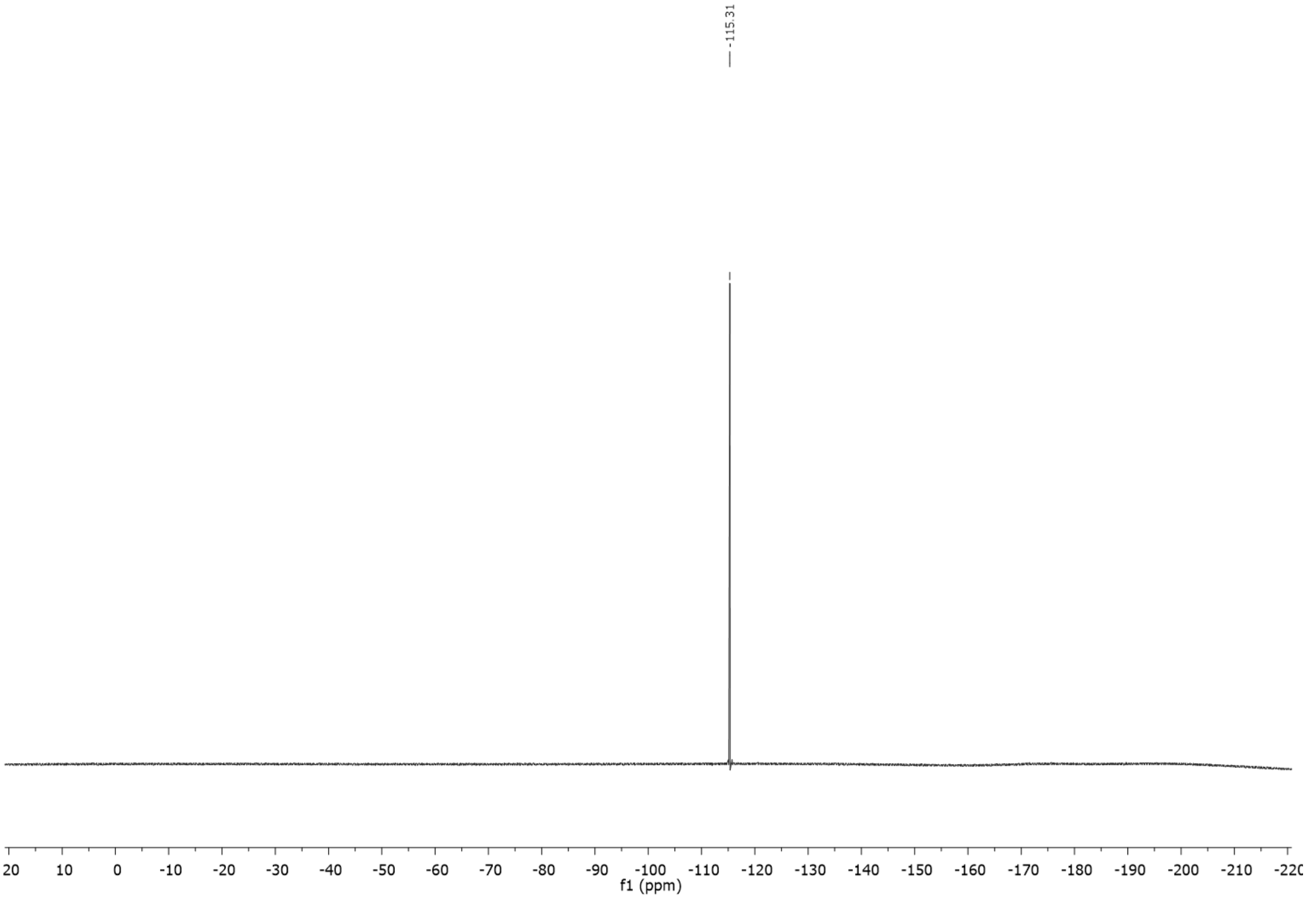
-115.31
20 10 0 -10 -20 -30 -40 -50 -60 -70 -80 -90 -100 -110 -120 -130 -140 -150 -160 -170 -180 -190 -200 -210 -220
f1 (ppm)

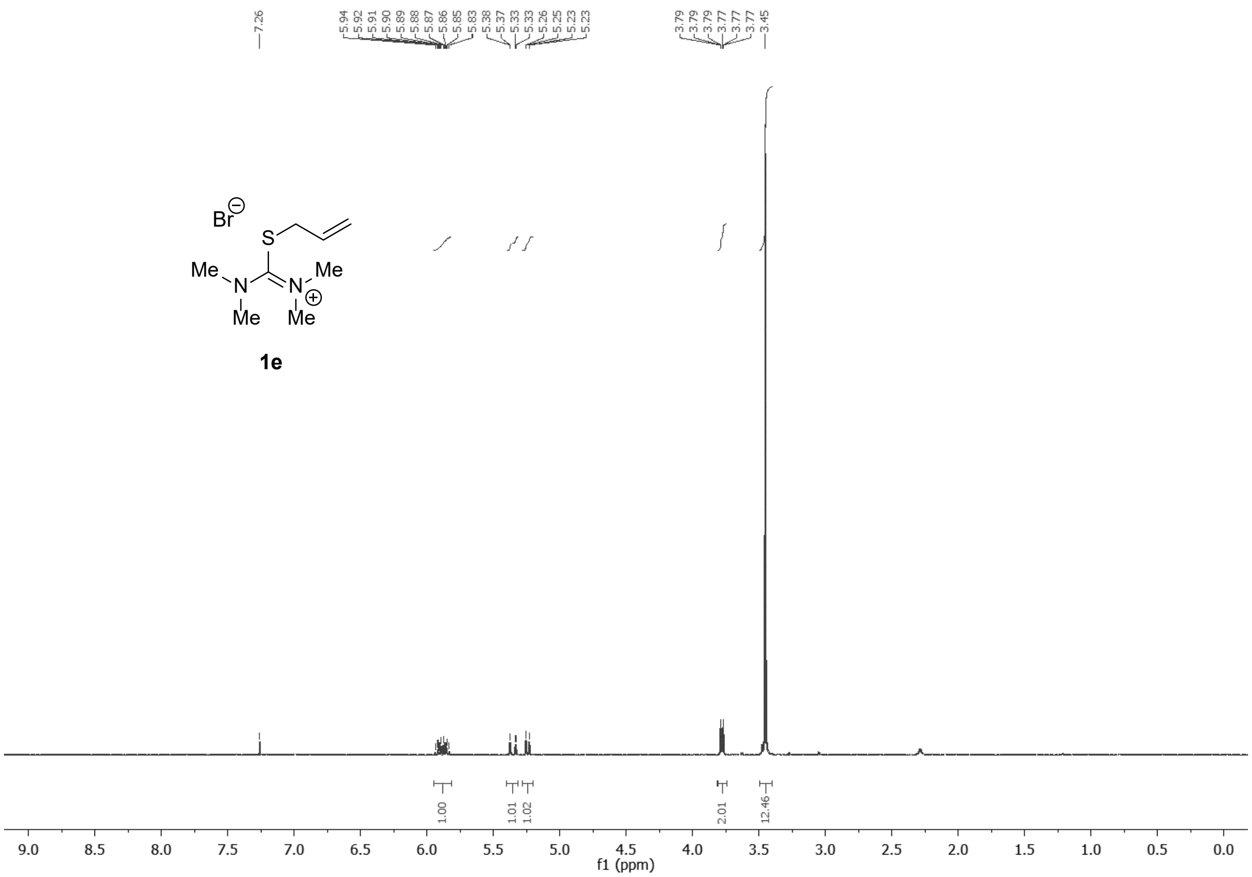

7.26
5.94 5.92 5.91 5.90 5.89 5.88 5.87 5.86 5.85 5.83 5.38 5.37 5.33 5.33 5.33 5.26 5.25 5.23 5.23
3.79 3.79 3.79 3.77 3.77 3.77 3.45
Br⊖
S
Me N N Me
Me Me ⊕
1e
1.00
1.01
1.02
2.01
12.46
9.0 8.5 8.0 7.5 7.0 6.5 6.0 5.5 5.0 4.5 4.0 3.5 3.0 2.5 2.0 1.5 1.0 0.5 0.0
f1 (ppm)

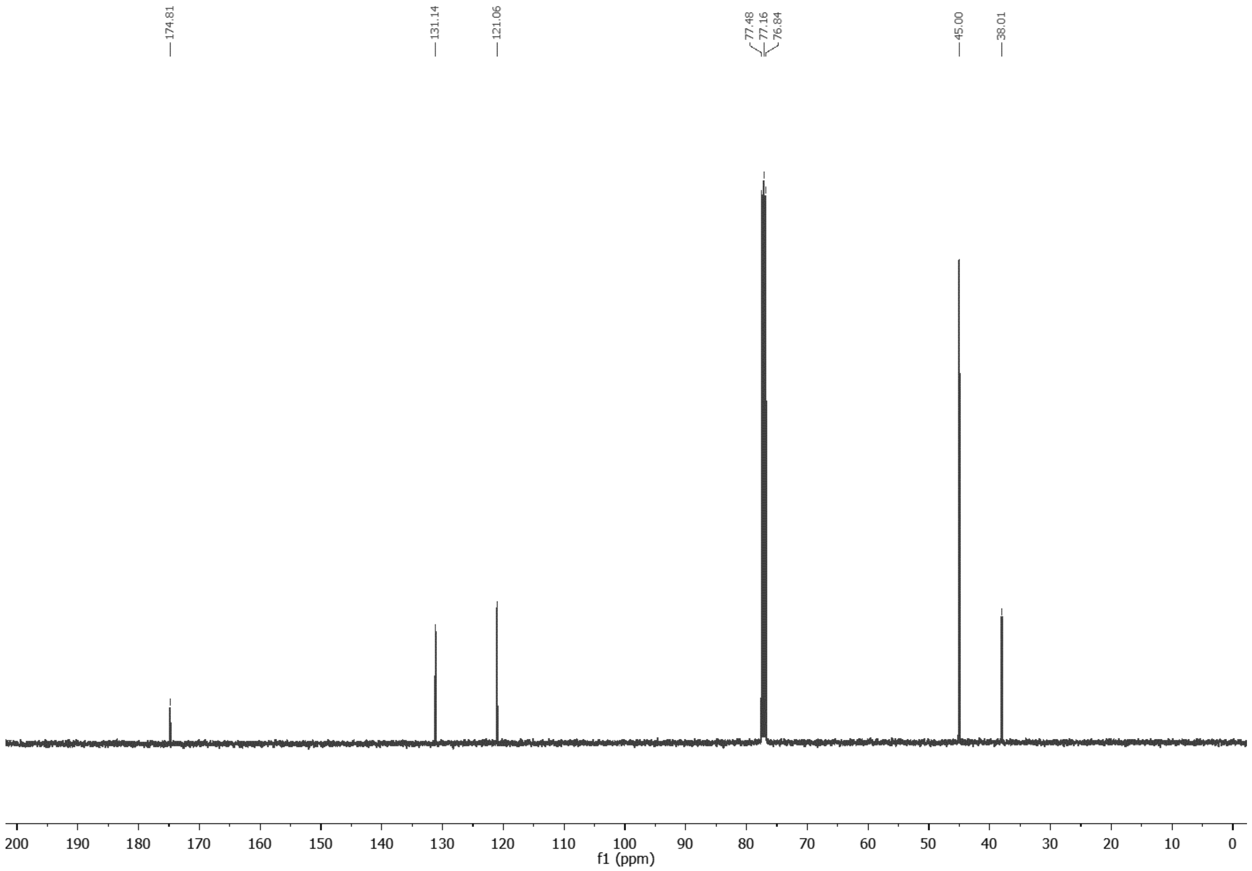

174.81
131.14
121.06
77.48 77.16 76.84
45.00
38.01
200 190 180 170 160 150 140 130 120 110 100 90 80 70 60 50 40 30 20 10 0
f1 (ppm)

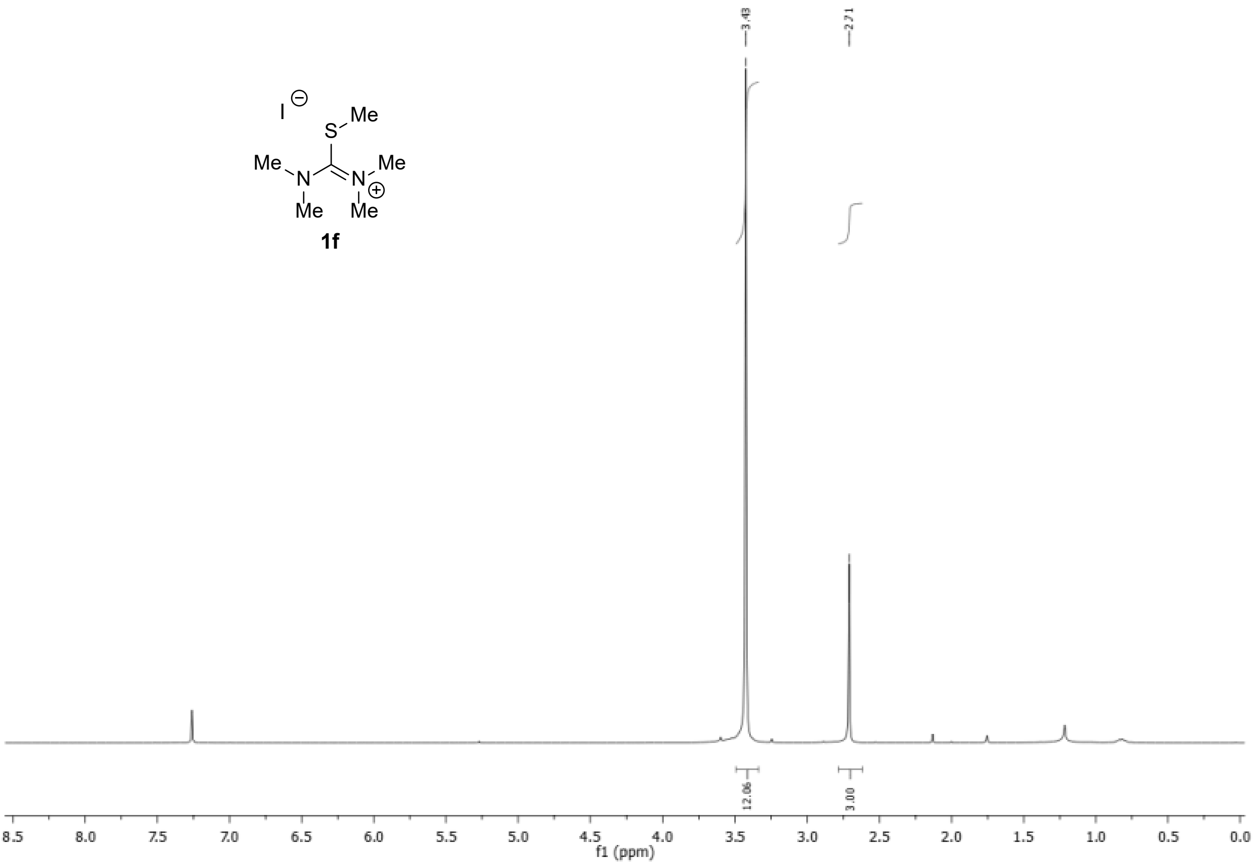

I
S
Me
Me
N
N
Me
Me
Me
1f
3.43
2.71
12.06
3.00
8.5 8.0 7.5 7.0 6.5 6.0 5.5 5.0 4.5 4.0 3.5 3.0 2.5 2.0 1.5 1.0 0.5 0.0
f1 (ppm)

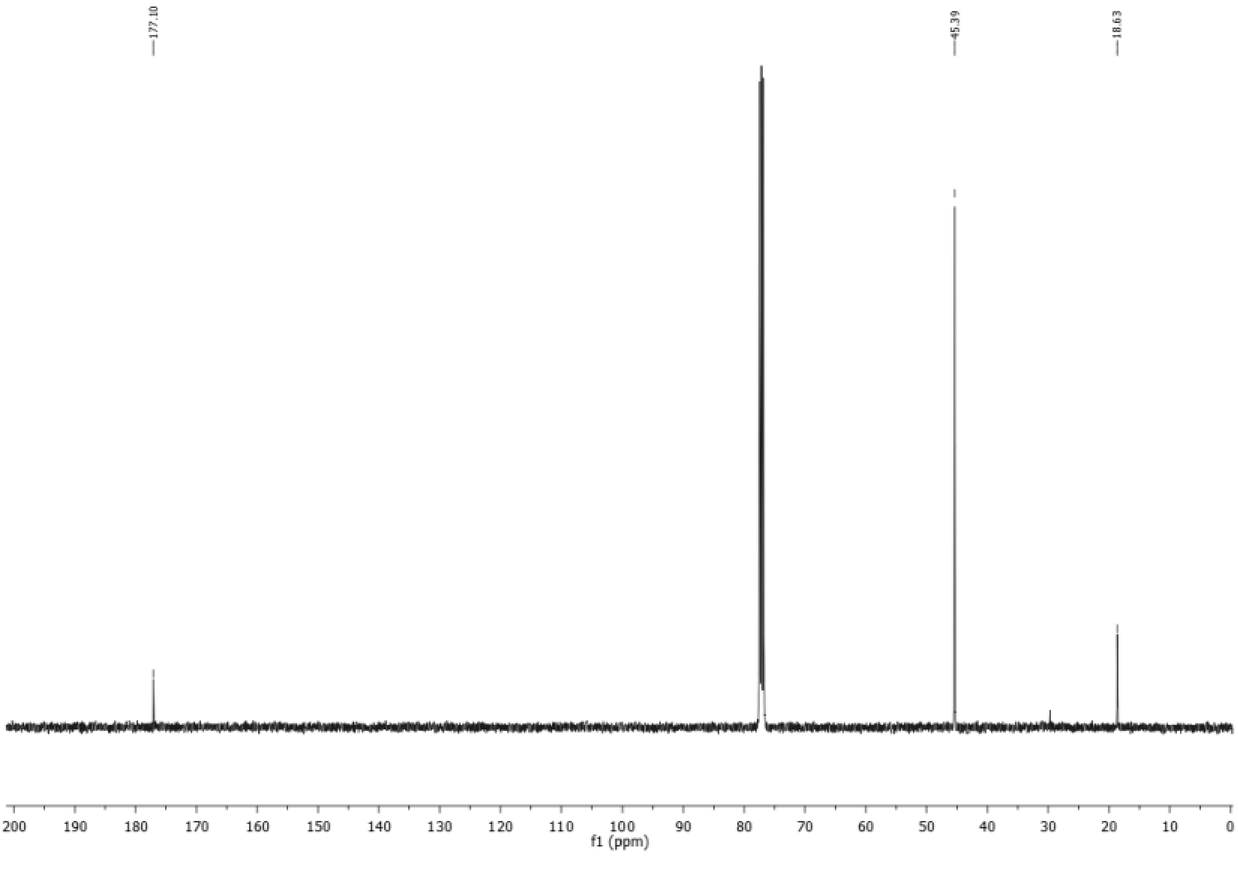

177.10
45.39
18.63
200 190 180 170 160 150 140 130 120 110 100 90 80 70 60 50 40 30 20 10 0
f1 (ppm)

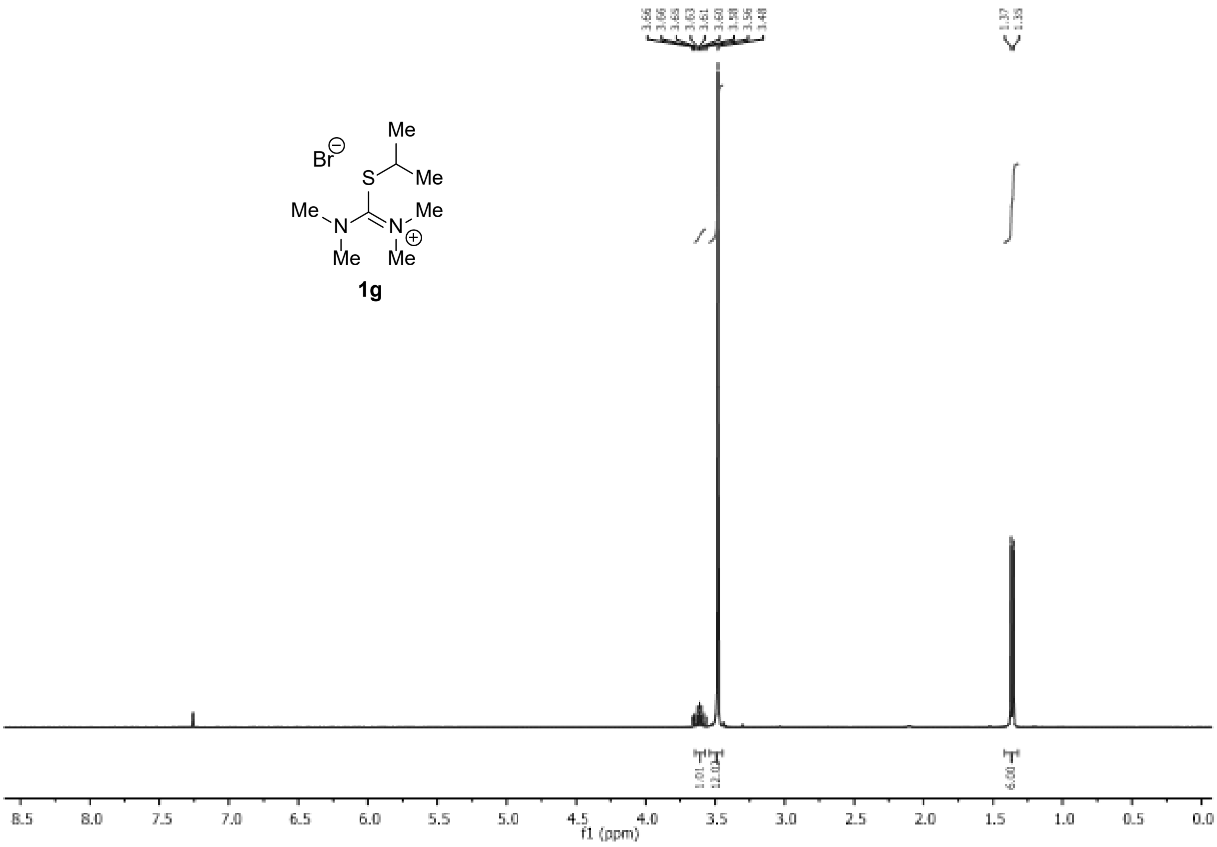
3.66 3.66 3.65 3.63 3.61 3.60 3.58 3.56 3.48
1.37 1.35
Br
Me
S
Me
Me
N
N
Me
Me
Me
1g
1.01
12.03
6.00
8.5 8.0 7.5 7.0 6.5 6.0 5.5 5.0 4.5 4.0 3.5 3.0 2.5 2.0 1.5 1.0 0.5 0.0
f1 (ppm)

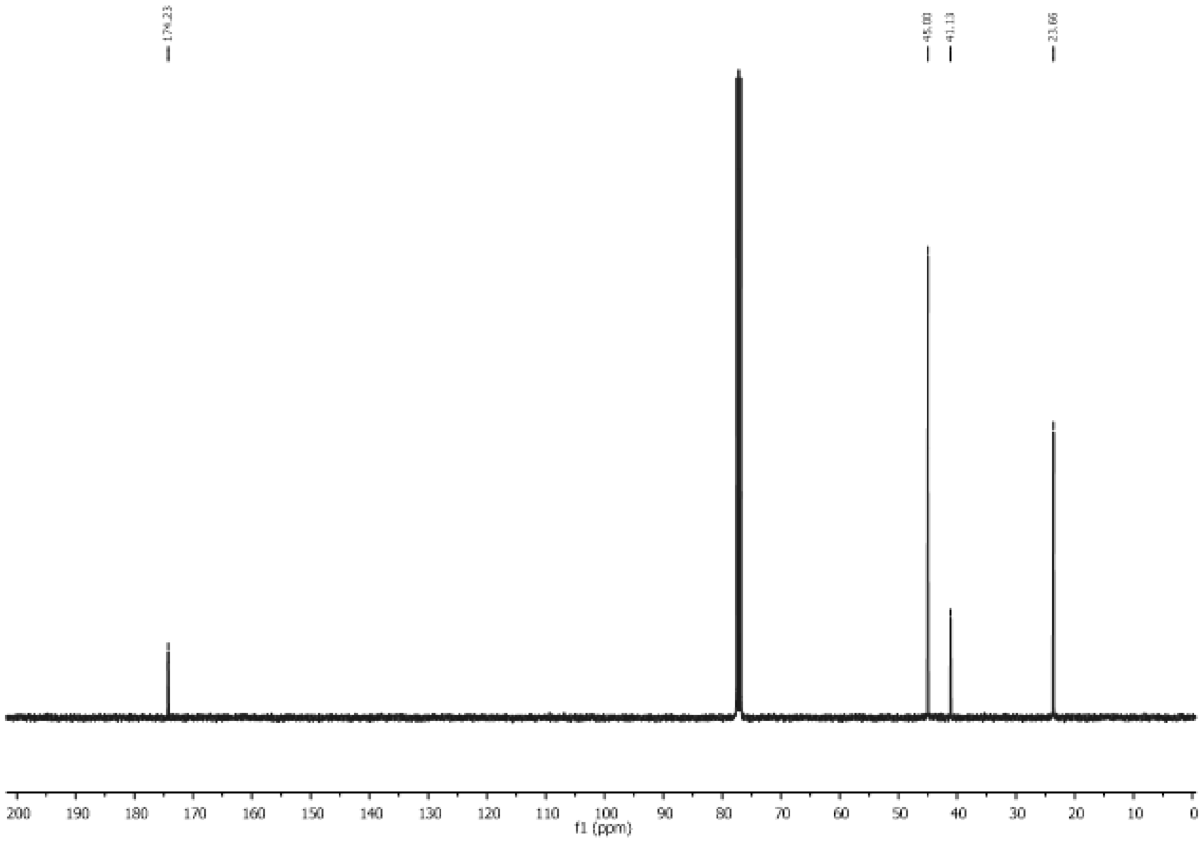
174.23
45.00
41.13
23.66
200 190 180 170 160 150 140 130 120 110 100 90 80 70 60 50 40 30 20 10 0
f1 (ppm)

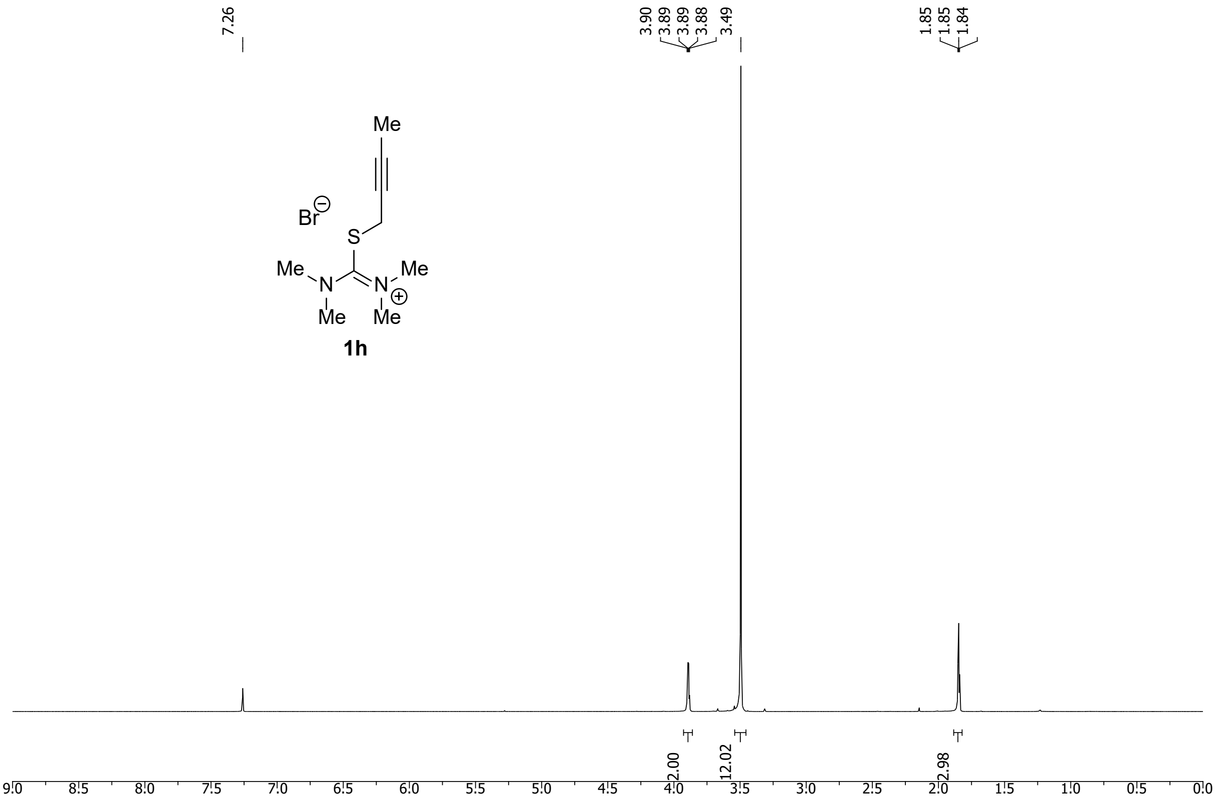
7.26
3.90
3.89
3.89
3.88
3.49
1.85
1.85
1.84
Me
Br
S
Me
N
Me
Me
Me
1h
2.00
12.02
2.98
9.0 8.5 8.0 7.5 7.0 6.5 6.0 5.5 5.0 4.5 4.0 3.5 3.0 2.5 2.0 1.5 1.0 0.5 0.0

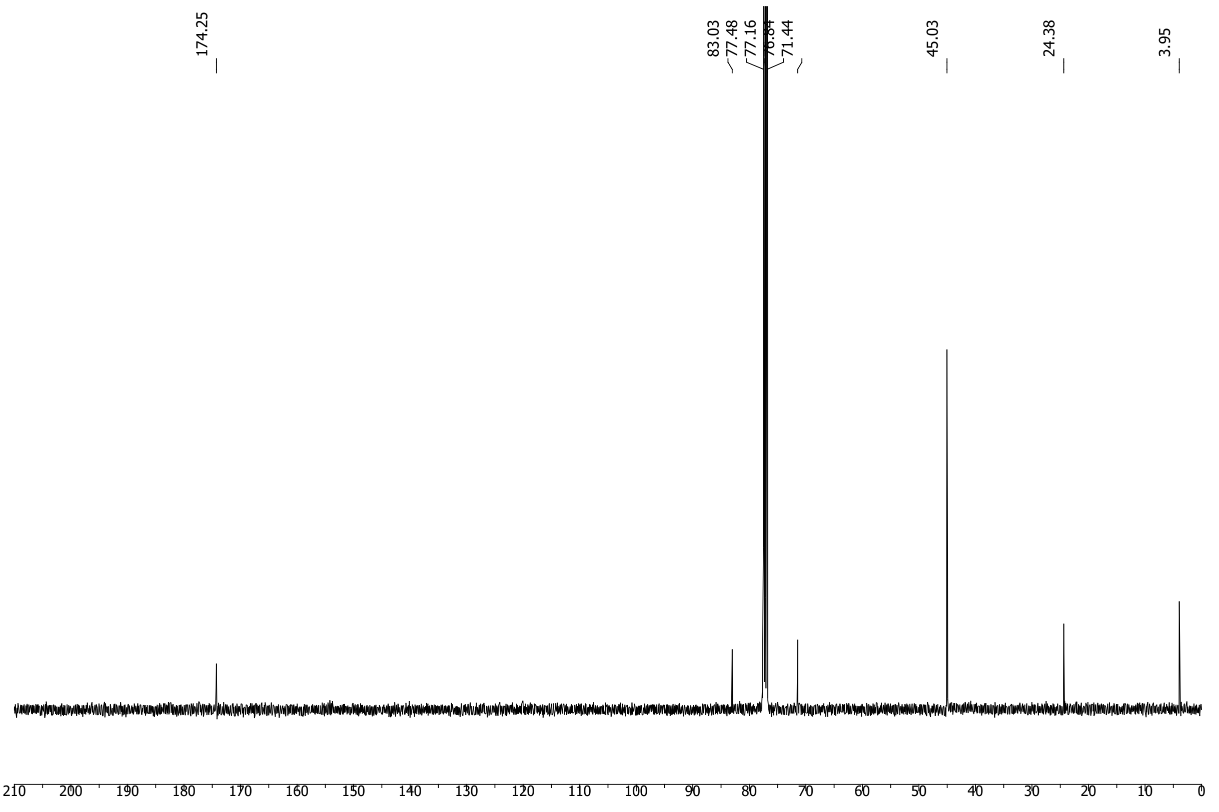
174.25
83.03
77.48
77.16
76.84
71.44
45.03
24.38
3.95
210 200 190 180 170 160 150 140 130 120 110 100 90 80 70 60 50 40 30 20 10 0

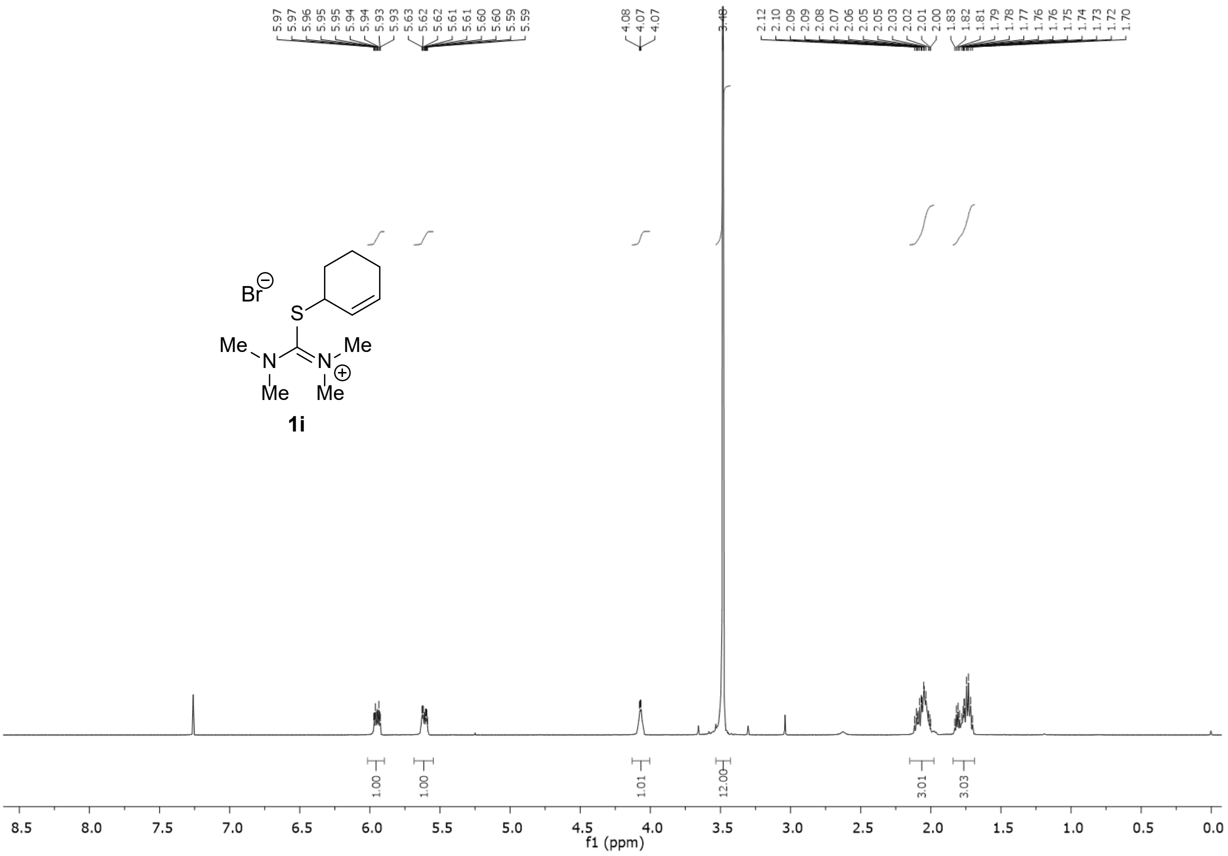
1i
1.00
1.00
1.01
12.00
3.01
3.03
f1 (ppm)

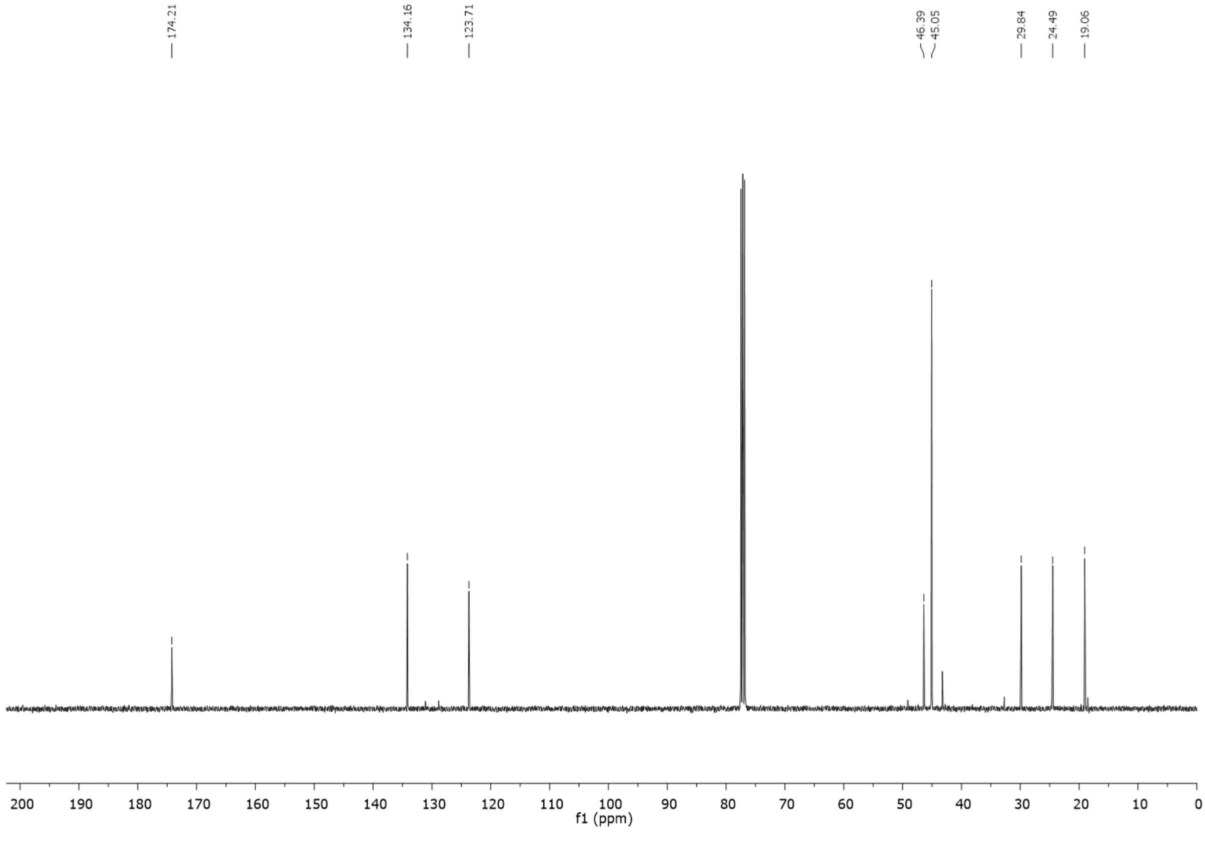
174.21
134.16
123.71
46.39
45.05
29.84
24.49
19.06
f1 (ppm)

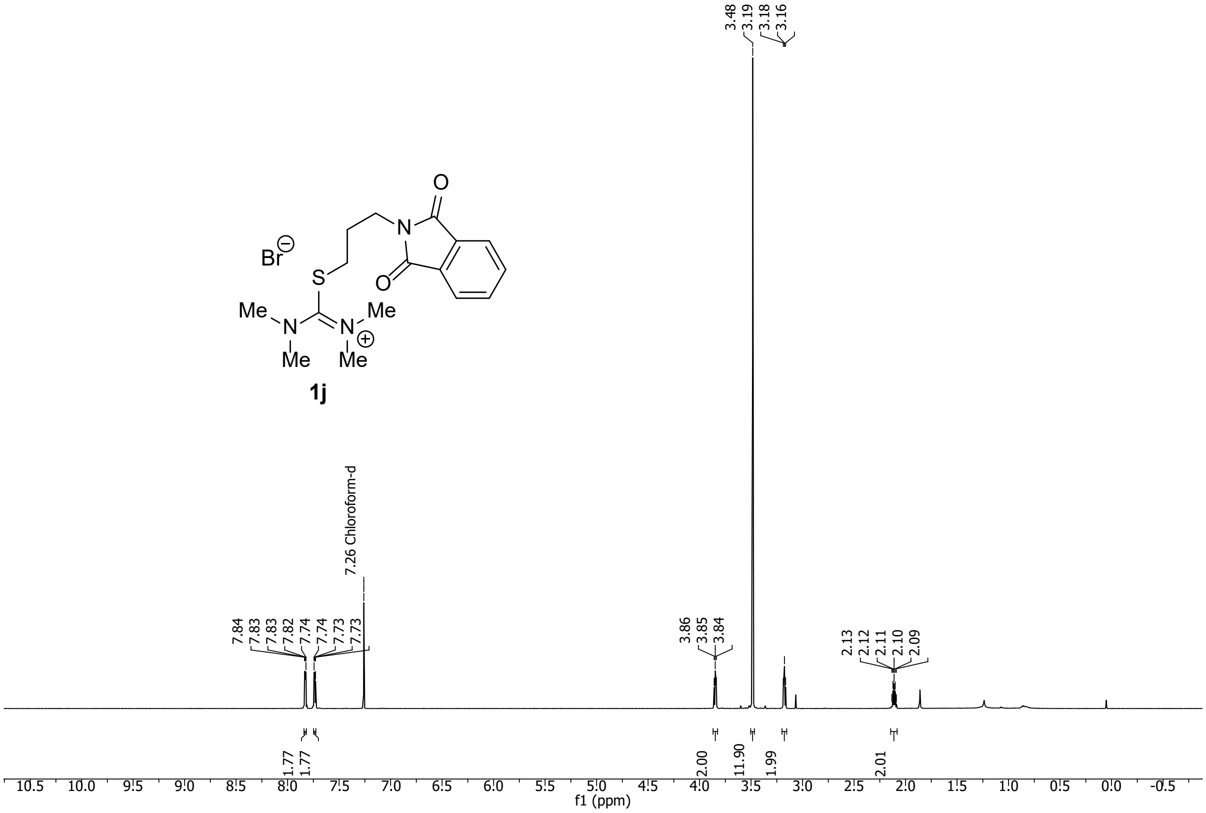
Br
S
O
N
O
Me
N
N
Me
Me
Me
1j
3.48
3.19
3.18
3.16
7.26 Chloroform-d
7.84
7.83
7.83
7.82
7.74
7.74
7.73
7.73
3.86
3.85
3.84
2.13
2.12
2.11
2.10
2.09
1.77
1.77
2.00
11.90
1.99
2.01
f1 (ppm)

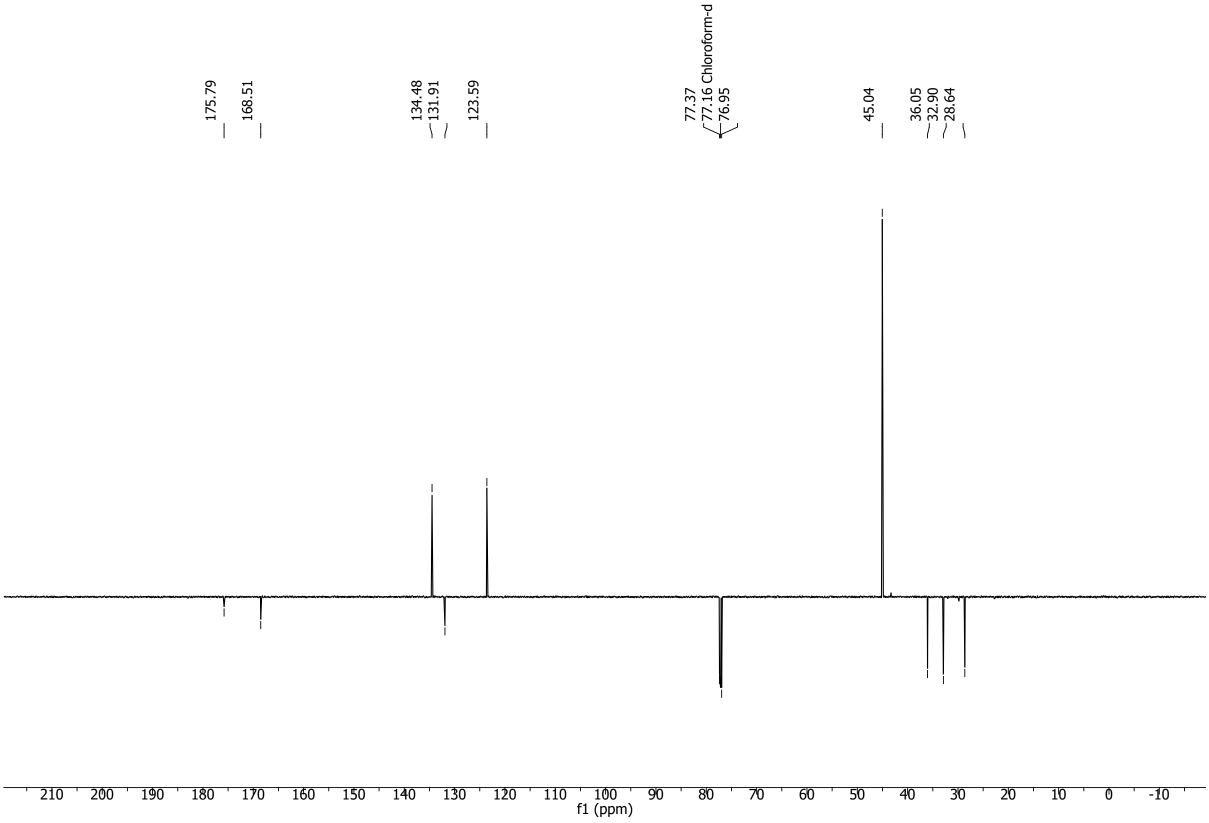
175.79
168.51
134.48
131.91
123.59
77.37
77.16 Chloroform-d
76.95
45.04
36.05
32.90
28.64
f1 (ppm)

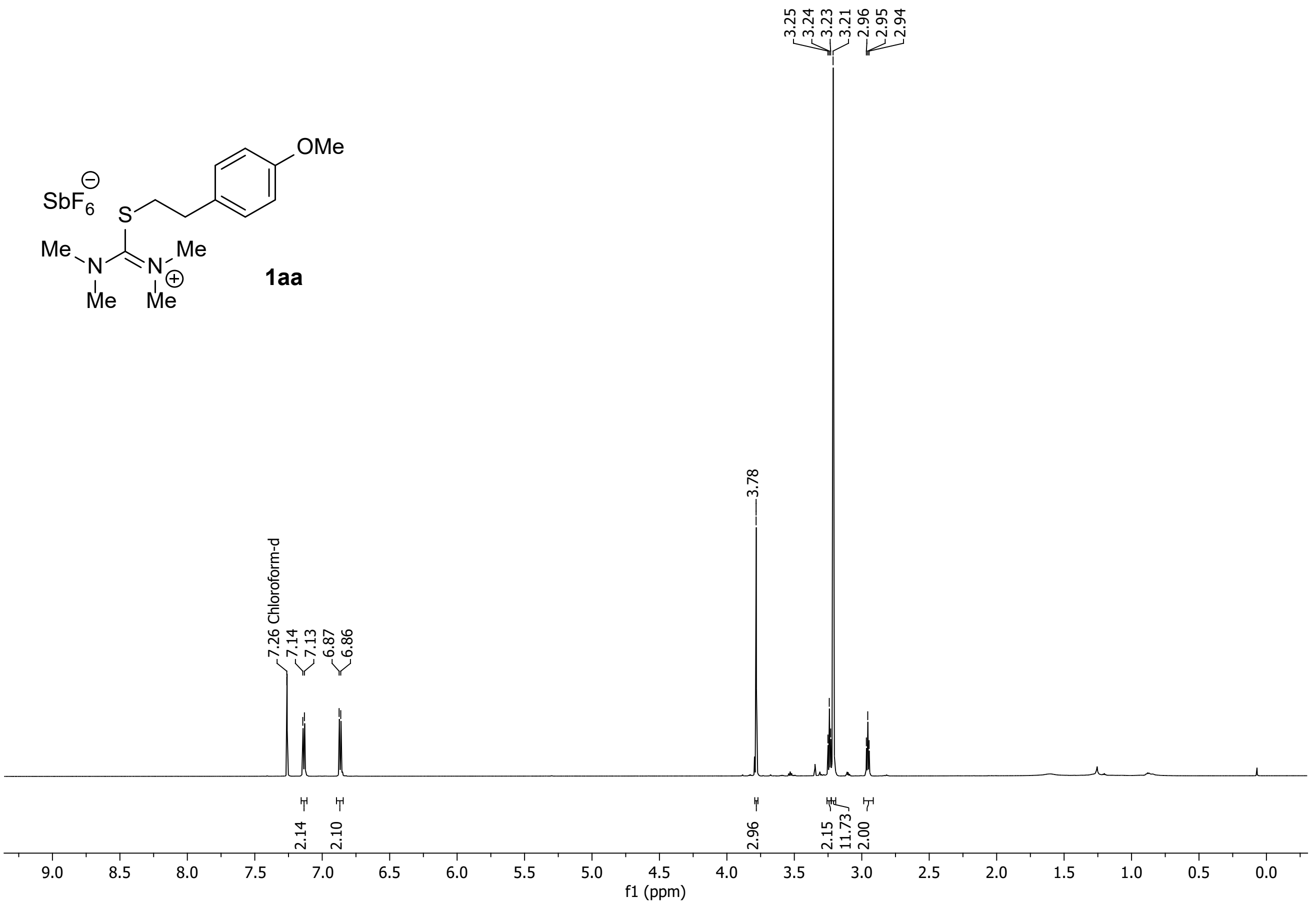
OMe
SbF6
S
Me
N
N
Me
Me
Me
1aa
3.25
3.24
3.23
3.21
2.96
2.95
2.94
3.78
7.26 Chloroform-d
7.14
7.13
6.87
6.86
2.14
2.10
2.96
2.15
11.73
2.00
9.0 8.5 8.0 7.5 7.0 6.5 6.0 5.5 5.0 4.5 4.0 3.5 3.0 2.5 2.0 1.5 1.0 0.5 0.0
f1 (ppm)

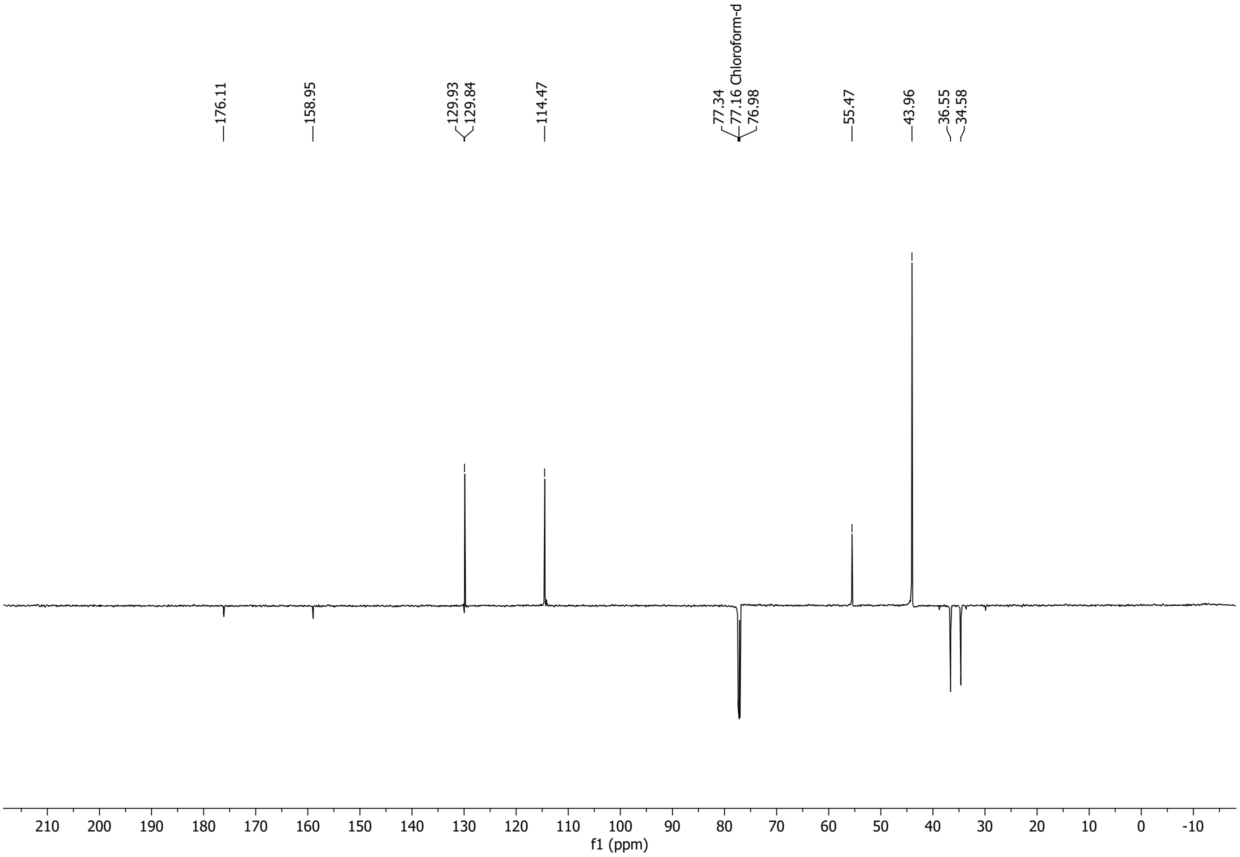
176.11
158.95
129.93
129.84
114.47
77.34
77.16 Chloroform-d
76.98
55.47
43.96
36.55
34.58
210 200 190 180 170 160 150 140 130 120 110 100 90 80 70 60 50 40 30 20 10 0 -10
f1 (ppm)

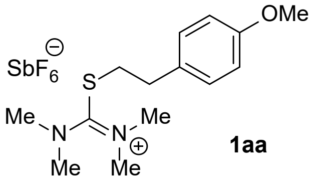
OMe
SbF6
S
Me
N
Me
N
Me
Me

1aa

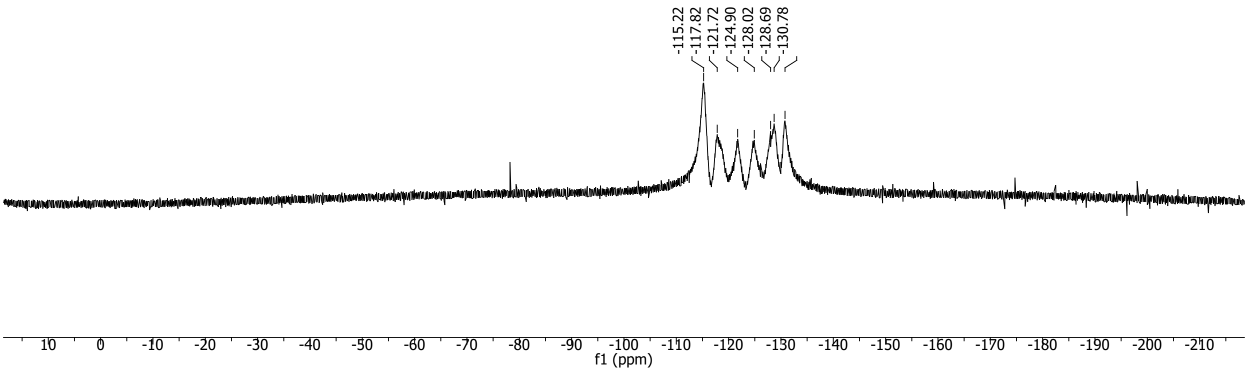
-115.22
-117.82
-121.72
-124.90
-128.02
-128.69
-130.78
10
0
-10
-20
-30
-40
-50
-60
-70
-80
-90
-100
-110
-120
-130
-140
-150
-160
-170
-180
-190
-200
-210
f1 (ppm)

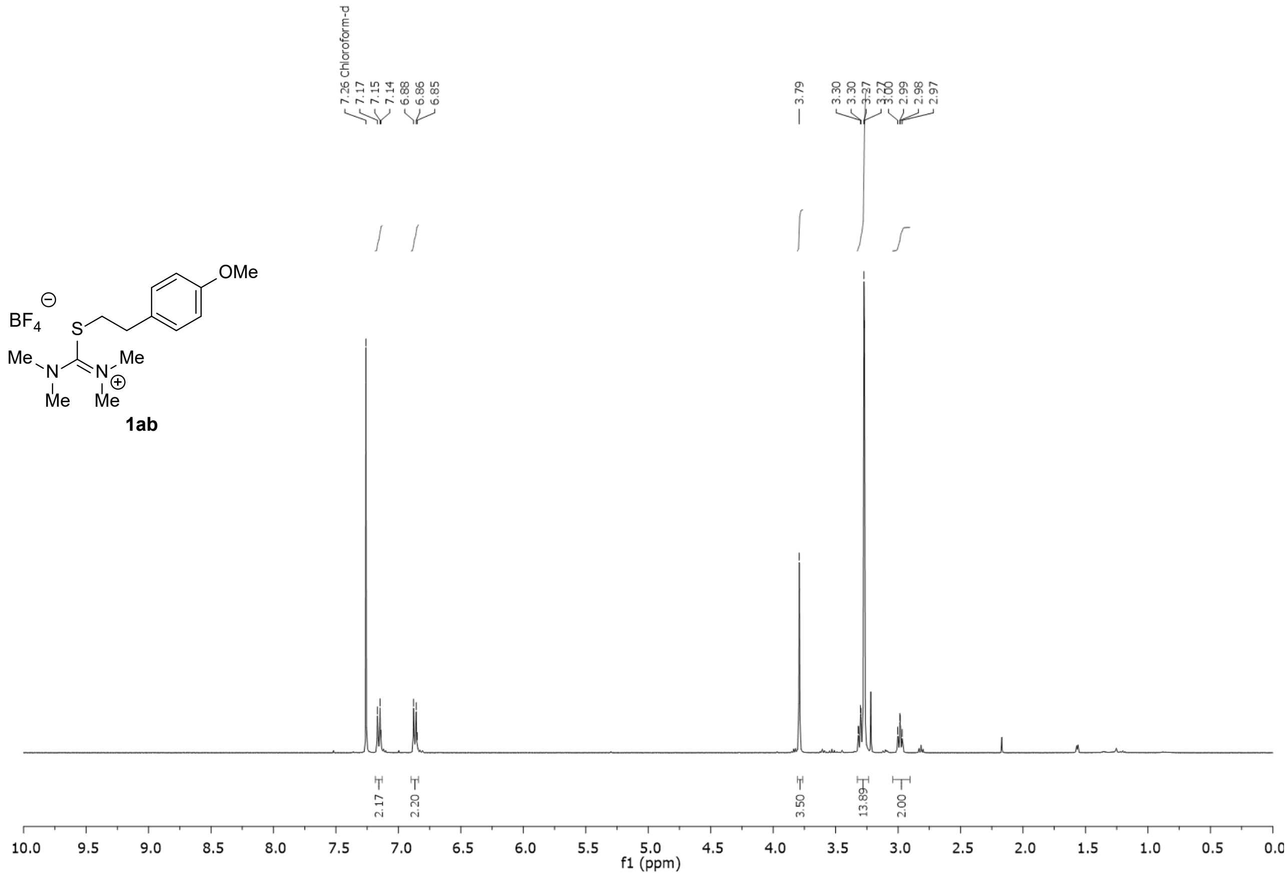
OMe
BF_4^-
S
Me
N
N
Me
Me
Me
7.26 Chloroform-d
7.17
7.15
7.14
6.88
6.86
6.85
3.79
3.30
3.30
3.27
3.27
3.00
2.99
2.98
2.97
2.17
2.20
3.50
13.89
2.00
10.0 9.5 9.0 8.5 8.0 7.5 7.0 6.5 6.0 5.5 5.0 4.5 4.0 3.5 3.0 2.5 2.0 1.5 1.0 0.5 0.0
f1 (ppm)

1ab

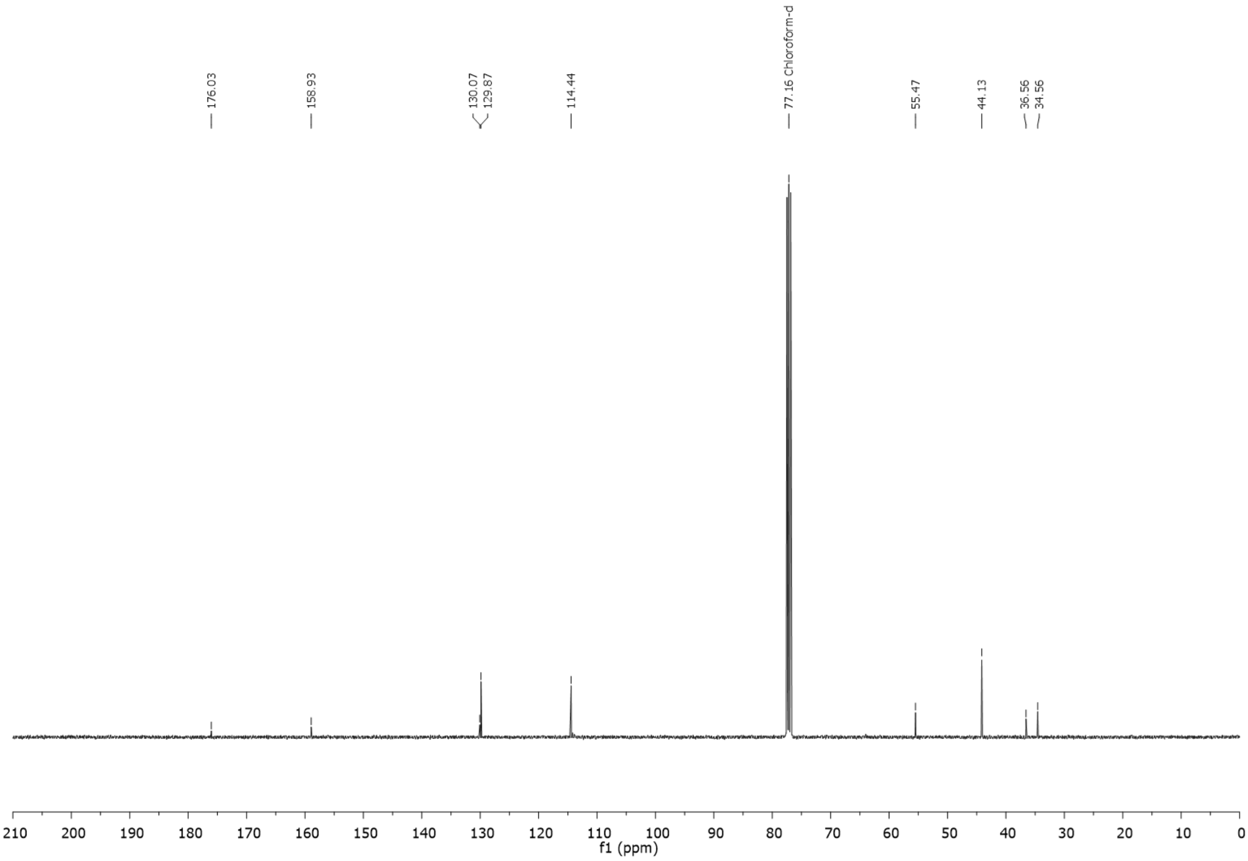
176.03
158.93
130.07
129.87
114.44
77.16 Chloroform-d
55.47
44.13
36.56
34.56
210 200 190 180 170 160 150 140 130 120 110 100 90 80 70 60 50 40 30 20 10 0
f1 (ppm)

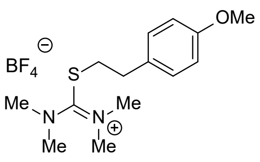
OMe
BF4
S
Me
N
Me
Me
N
Me

1ab

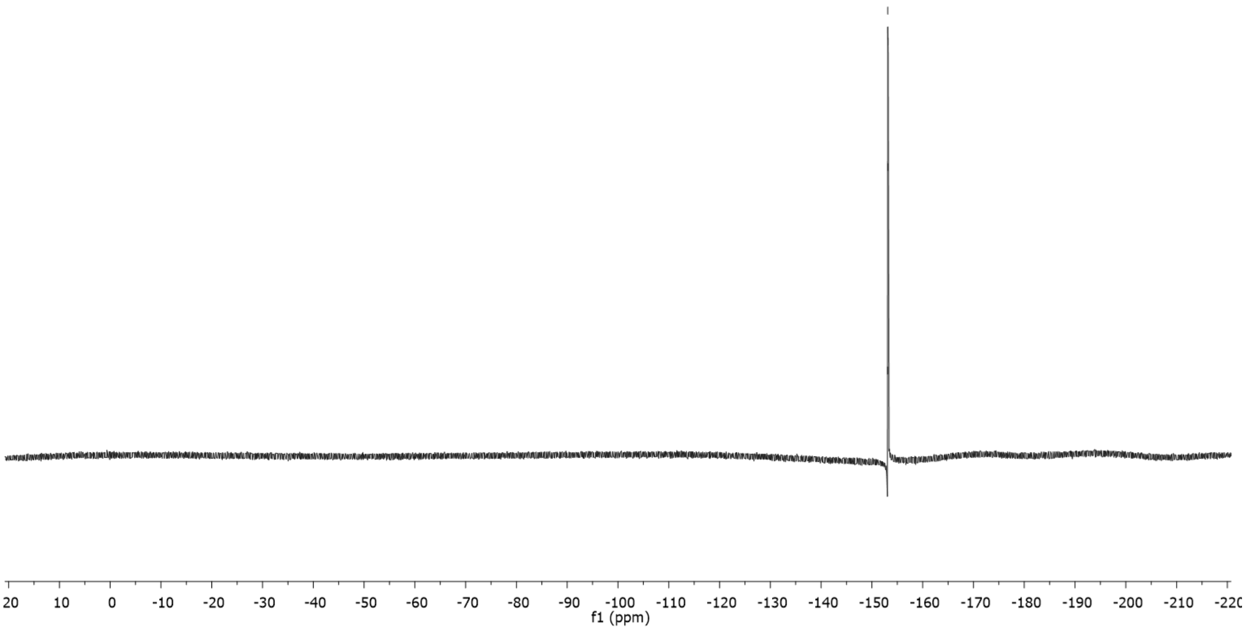
-153.08
-153.13
-153.16
-153.21
20 10 0 -10 -20 -30 -40 -50 -60 -70 -80 -90 -100 -110 -120 -130 -140 -150 -160 -170 -180 -190 -200 -210 -220
f1 (ppm)

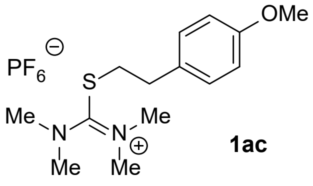

1ac

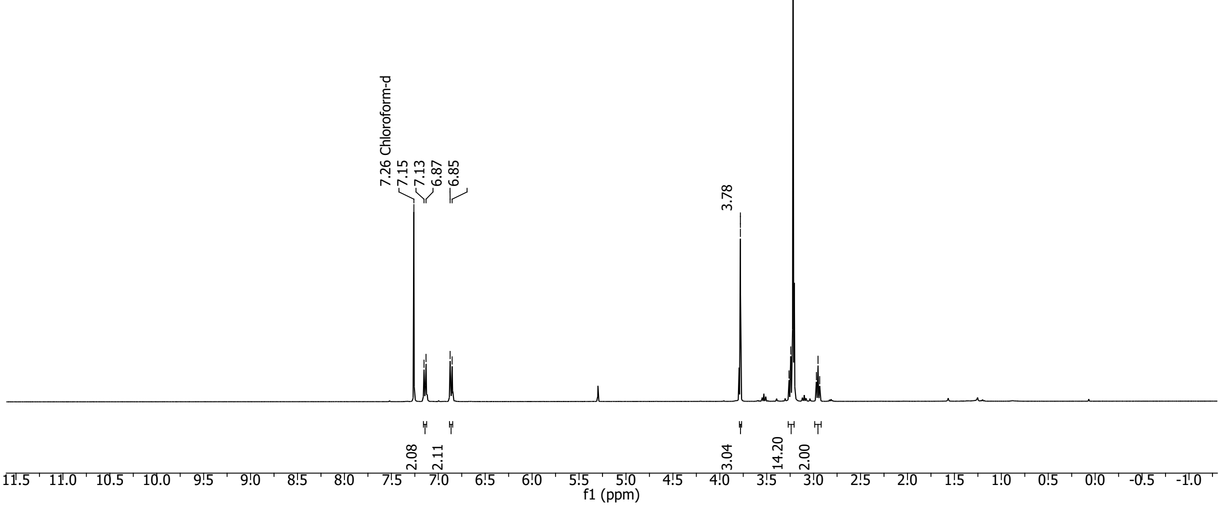

7.26 Chloroform-d
7.15
7.13
6.87
6.85
3.78
3.26
3.24
3.22
2.97
2.95
2.94
2.08
2.11
3.04
14.20
2.00
f1 (ppm)

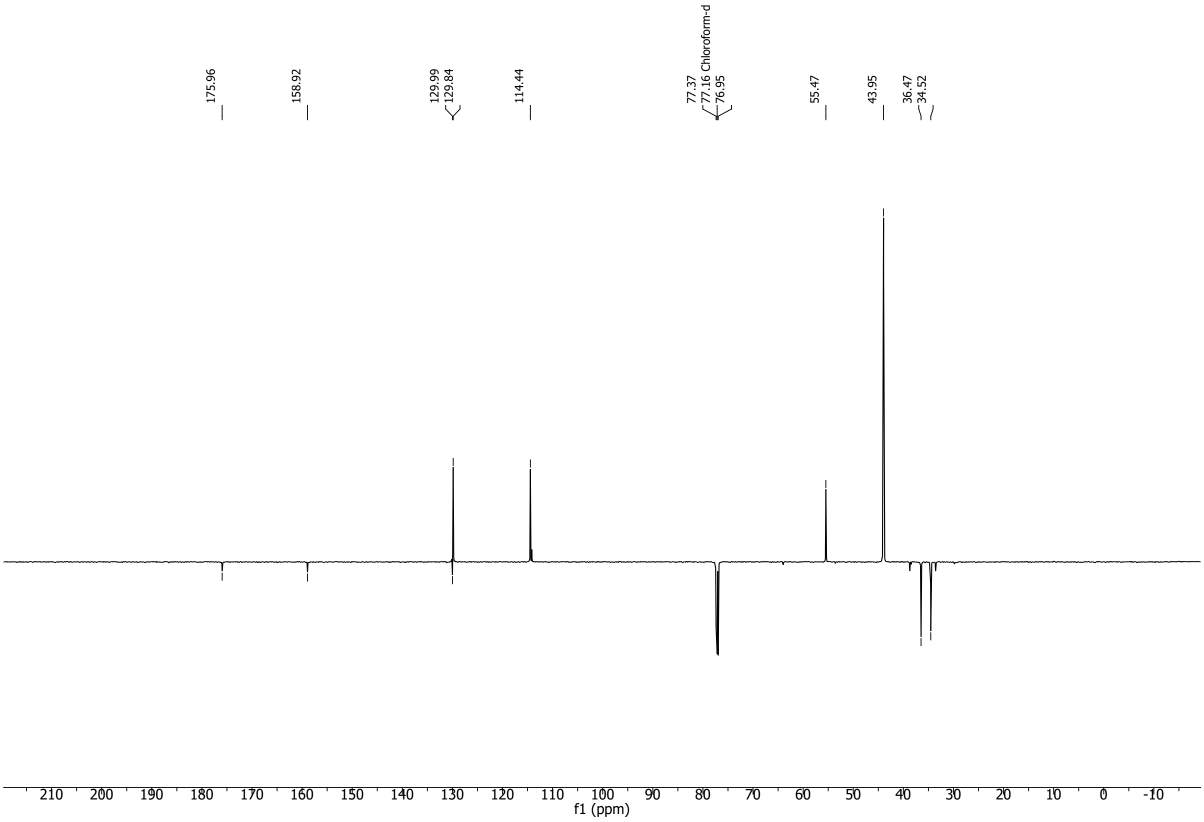

175.96
158.92
129.99
129.84
114.44
77.37
77.16 Chloroform-d
76.95
55.47
43.95
36.47
34.52
f1 (ppm)

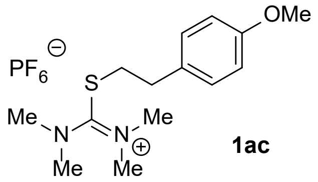
OMe
$PF_6^{\ominus}$
S
Me
N
Me
Me
N
Me

1ac

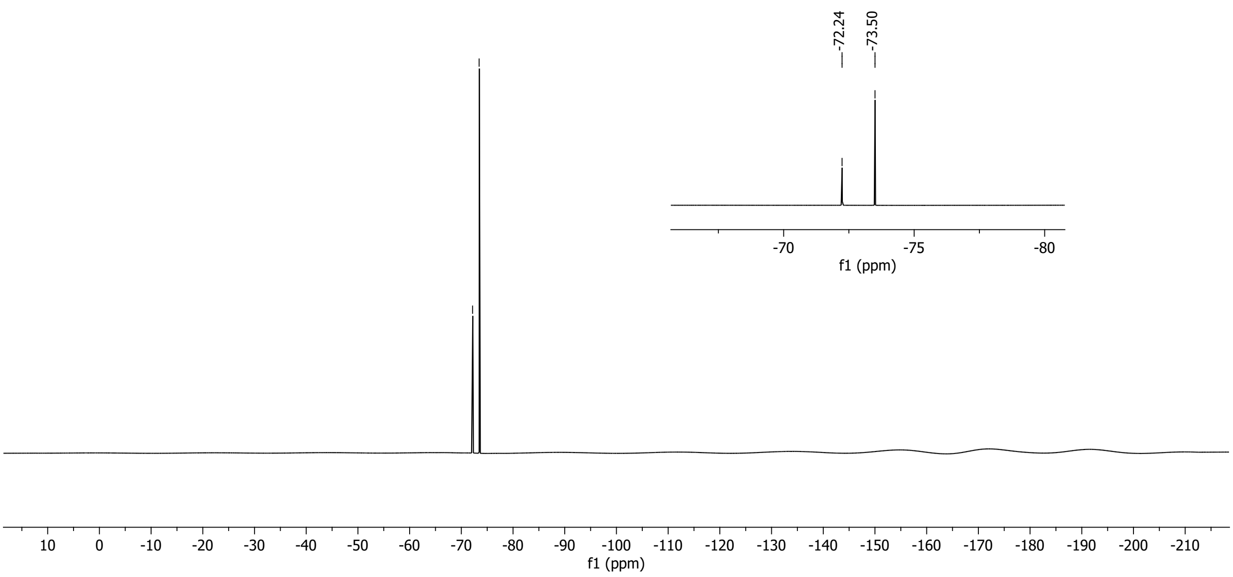
-72.24
-73.50
f1 (ppm)

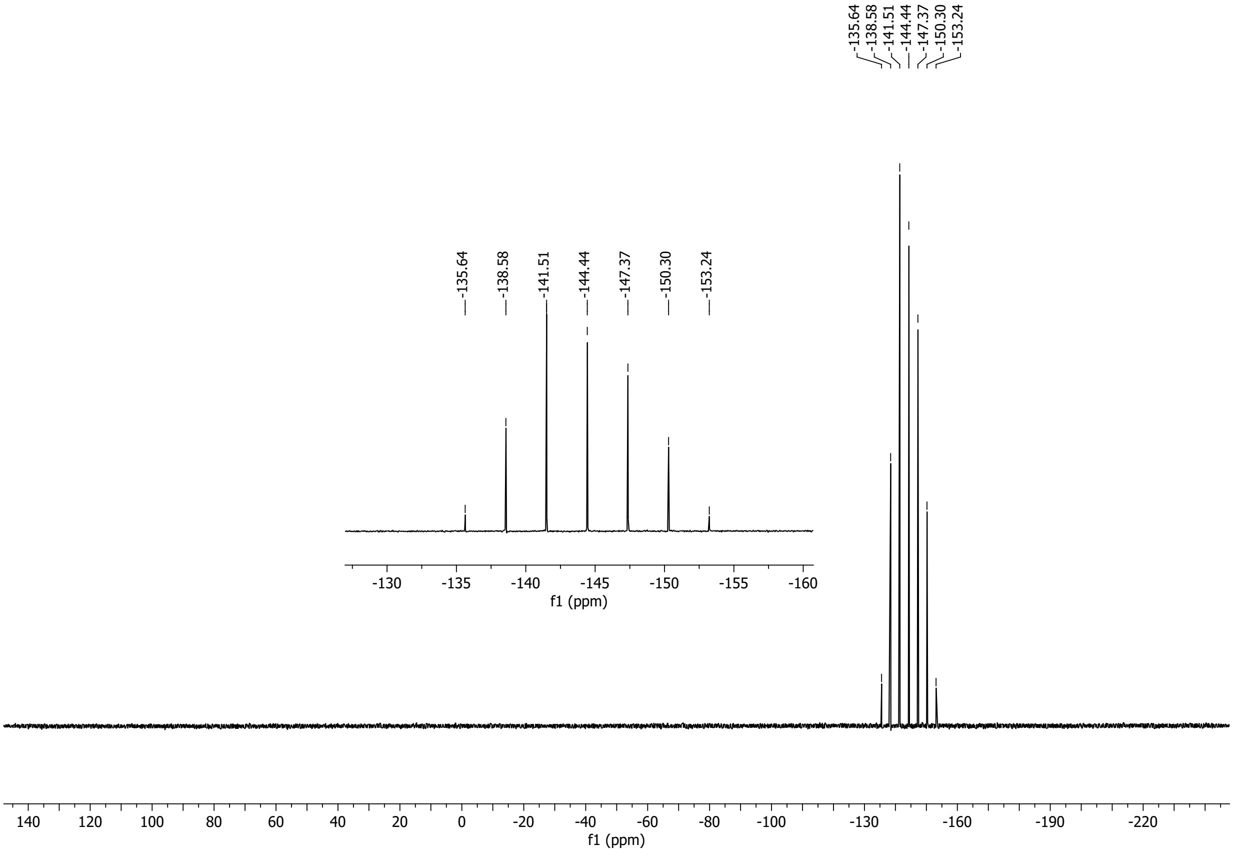
-135.64
-138.58
-141.51
-144.44
-147.37
-150.30
-153.24
f1 (ppm)

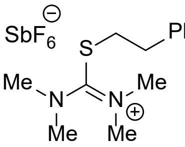
SbF6
S
Ph
Me
N
N
Me
Me
Me

1bb

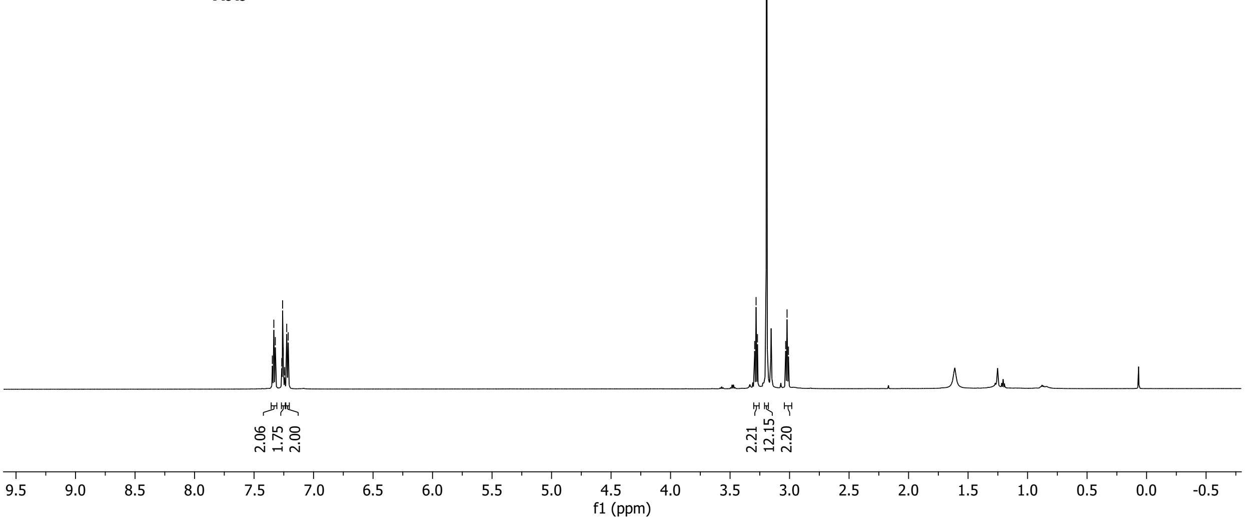
7.35
7.33
7.32
7.27
7.26 Chloroform-d
7.25
7.24
7.23
7.21
3.29
3.28
3.27
3.19
3.03
3.02
3.01
2.06
1.75
2.00
2.21
12.15
2.20
9.5 9.0 8.5 8.0 7.5 7.0 6.5 6.0 5.5 5.0 4.5 4.0 3.5 3.0 2.5 2.0 1.5 1.0 0.5 0.0 -0.5
f1 (ppm)

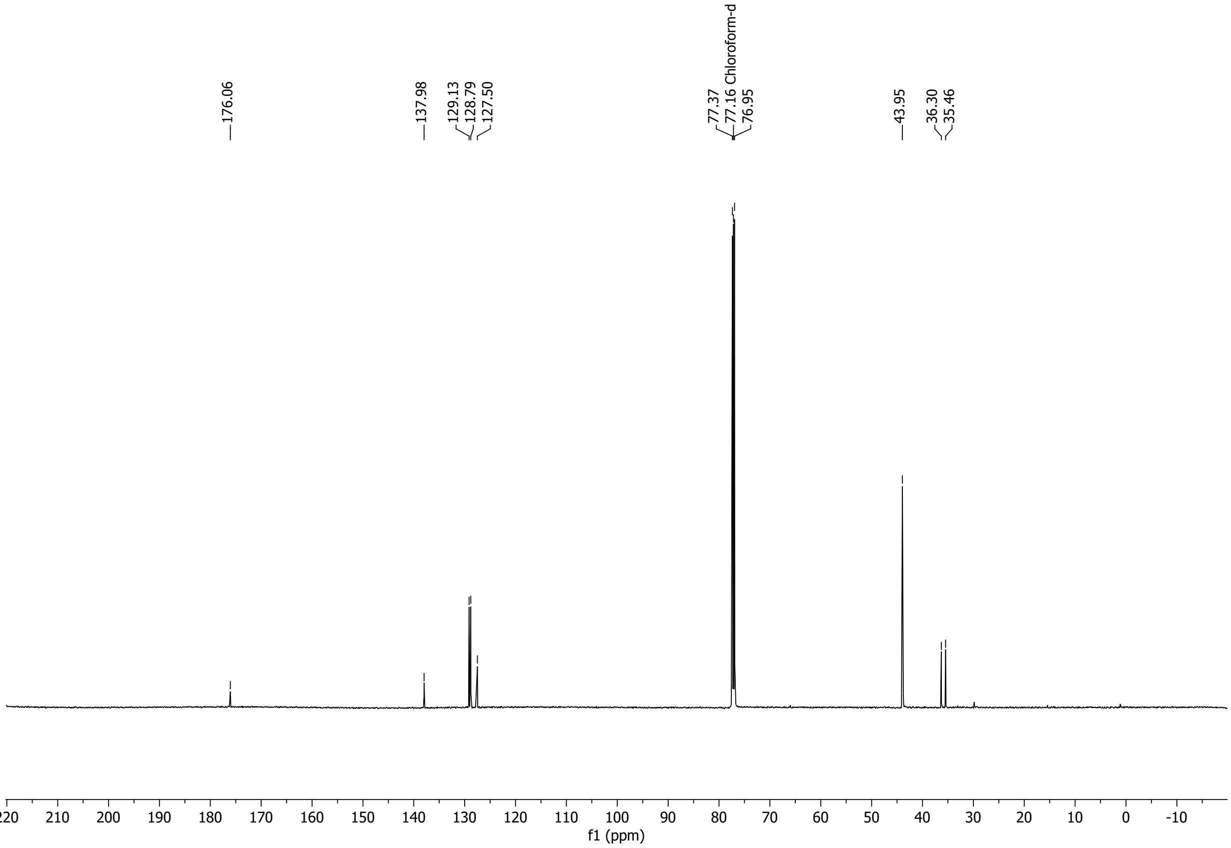
176.06
137.98
129.13
128.79
127.50
77.37
77.16 Chloroform-d
76.95
43.95
36.30
35.46
220 210 200 190 180 170 160 150 140 130 120 110 100 90 80 70 60 50 40 30 20 10 0 -10
f1 (ppm)

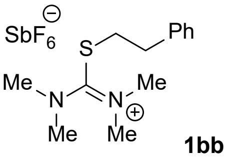
SbF6
S
Ph
Me
N
N
Me
Me
Me

1bb

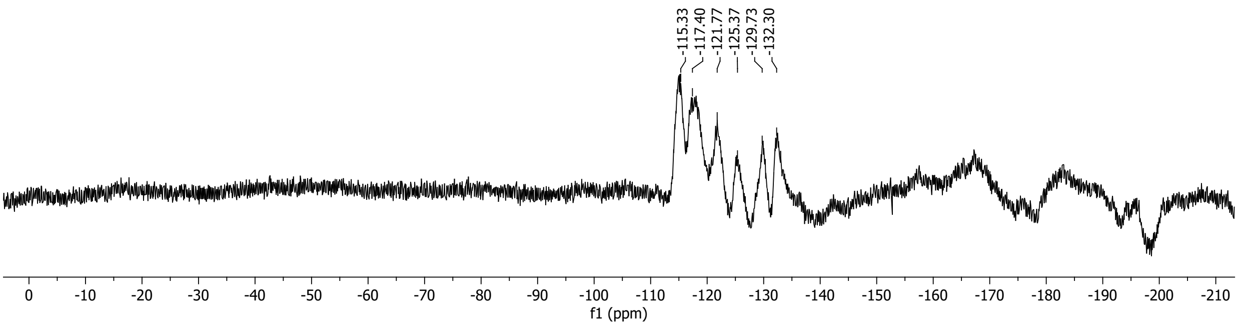
-115.33
-117.40
-121.77
-125.37
-129.73
-132.30
0 -10 -20 -30 -40 -50 -60 -70 -80 -90 -100 -110 -120 -130 -140 -150 -160 -170 -180 -190 -200 -210
f1 (ppm)

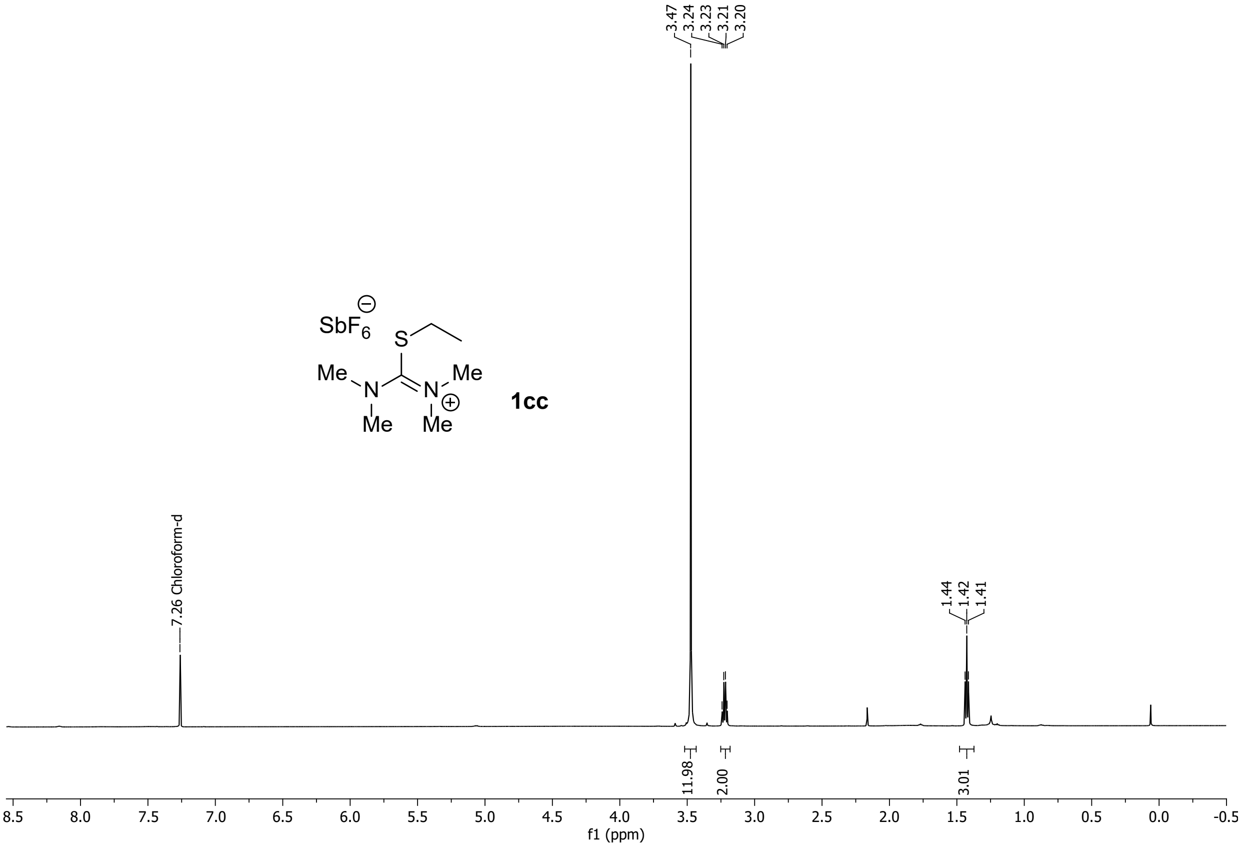
3.47
3.24
3.23
3.21
3.20
SbF6
S
Me
N
N
Me
Me
Me
1cc
7.26 Chloroform-d
1.44
1.42
1.41
11.98
2.00
3.01
f1 (ppm)

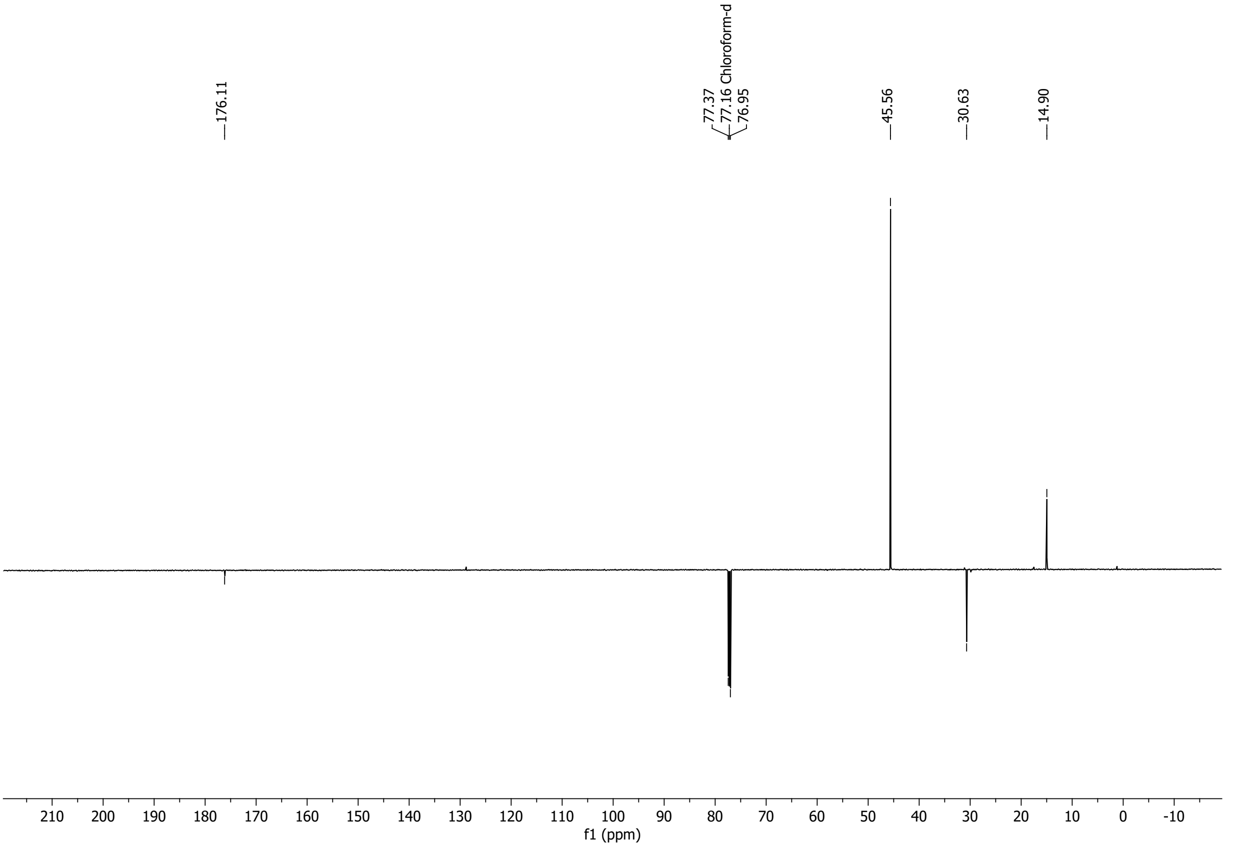
176.11
77.37
77.16 Chloroform-d
76.95
45.56
30.63
14.90
f1 (ppm)

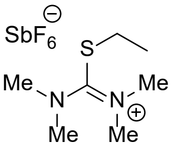

SbF6
S
Me
N
Me
N
Me
Me

1cc

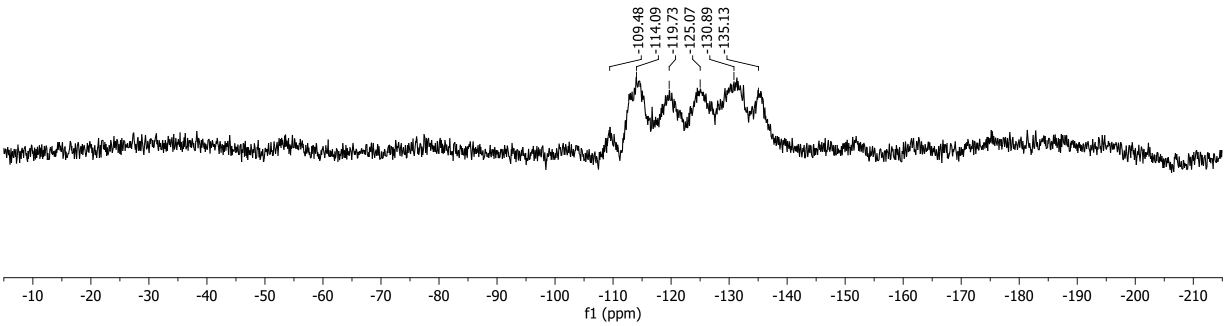

-109.48
-114.09
-119.73
-125.07
-130.89
-135.13
-10 -20 -30 -40 -50 -60 -70 -80 -90 -100 -110 -120 -130 -140 -150 -160 -170 -180 -190 -200 -210
f1 (ppm)

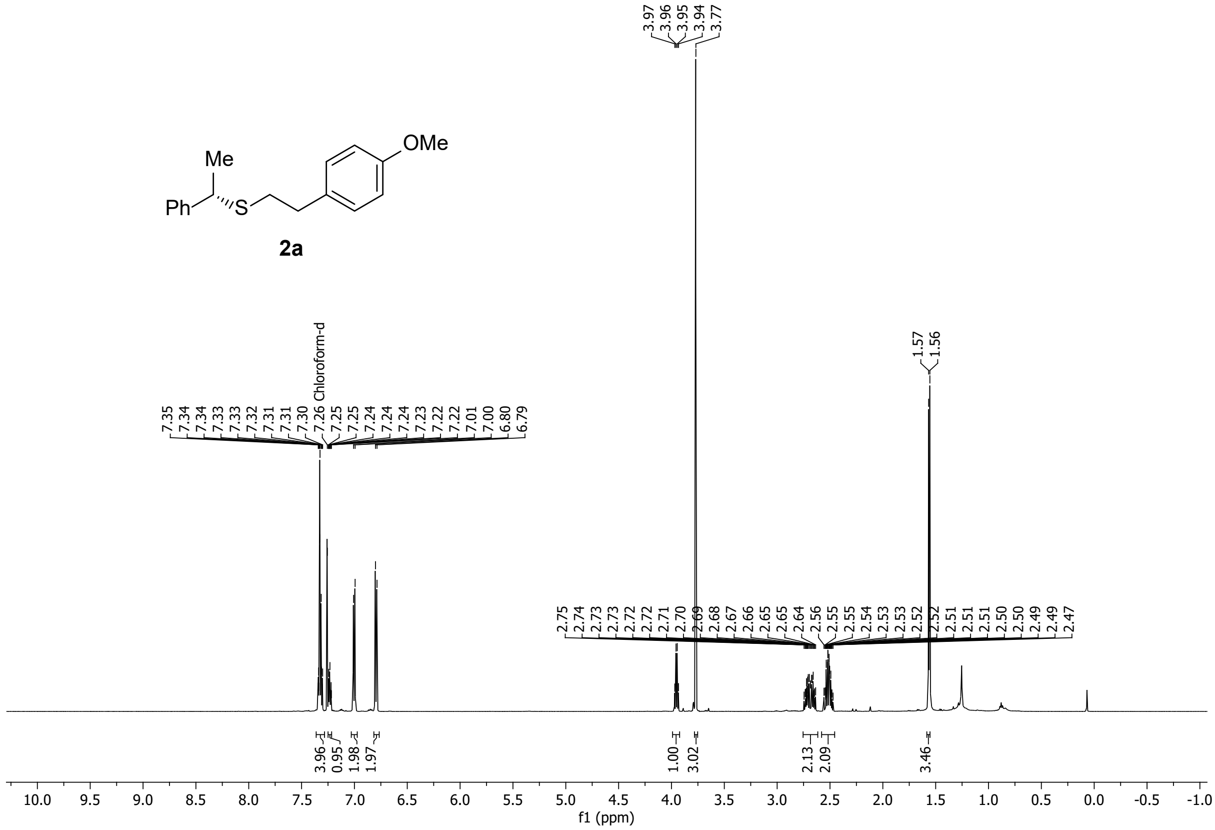
OMe
Me
Ph
S
2a
3.97 3.96 3.95 3.94 3.77
7.35 7.34 7.34 7.33 7.33 7.32 7.31 7.31 7.30 7.26 Chloroform-d 7.25 7.25 7.24 7.24 7.24 7.23 7.22 7.22 7.22 7.01 7.00 6.80 6.79
1.57 1.56
2.75 2.74 2.73 2.73 2.72 2.72 2.71 2.70 2.69 2.68 2.67 2.66 2.65 2.65 2.64 2.56 2.55 2.55 2.54 2.53 2.53 2.52 2.52 2.51 2.51 2.51 2.50 2.50 2.49 2.49 2.47
3.96 0.95 1.98 1.97 1.00 3.02 2.13 2.09 3.46
f1 (ppm)

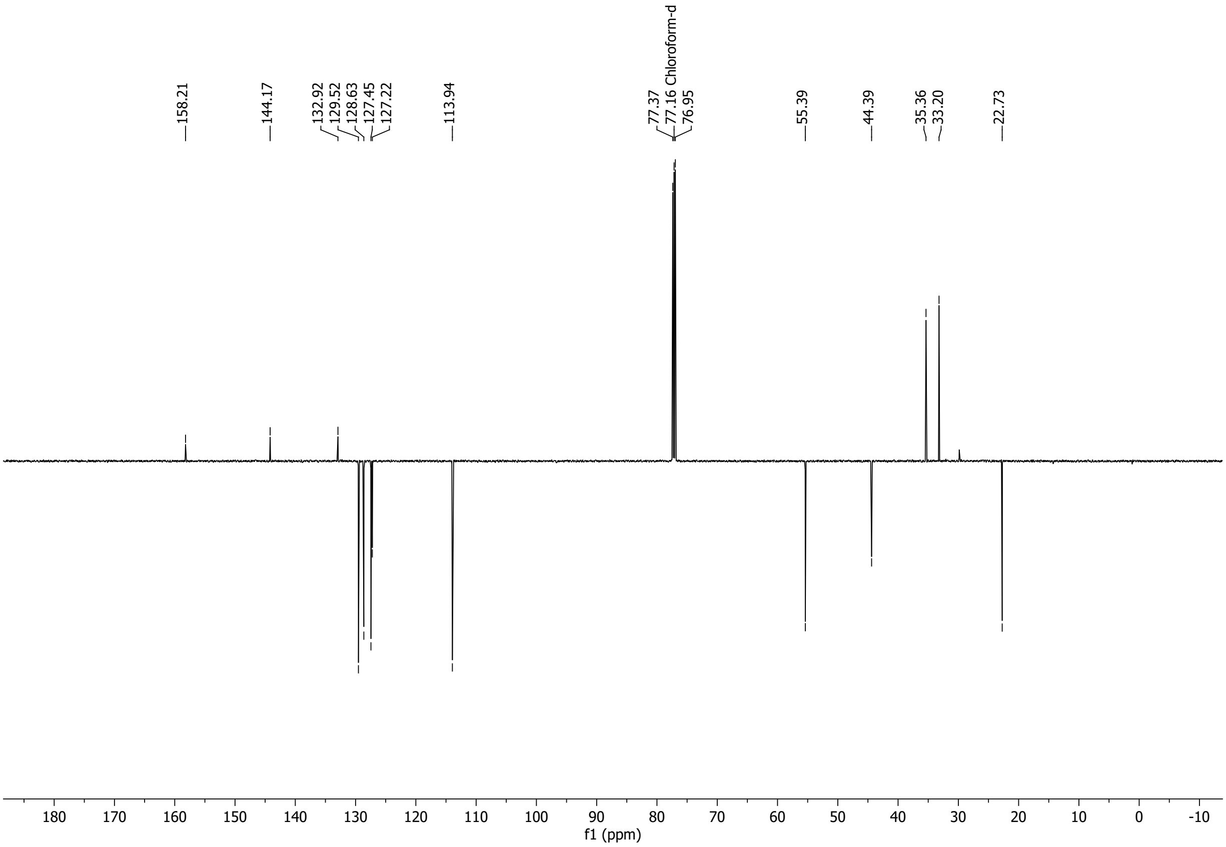
158.21 144.17 132.92 129.52 128.63 127.45 127.22 113.94 77.37 77.16 Chloroform-d 76.95 55.39 44.39 35.36 33.20 22.73
f1 (ppm)

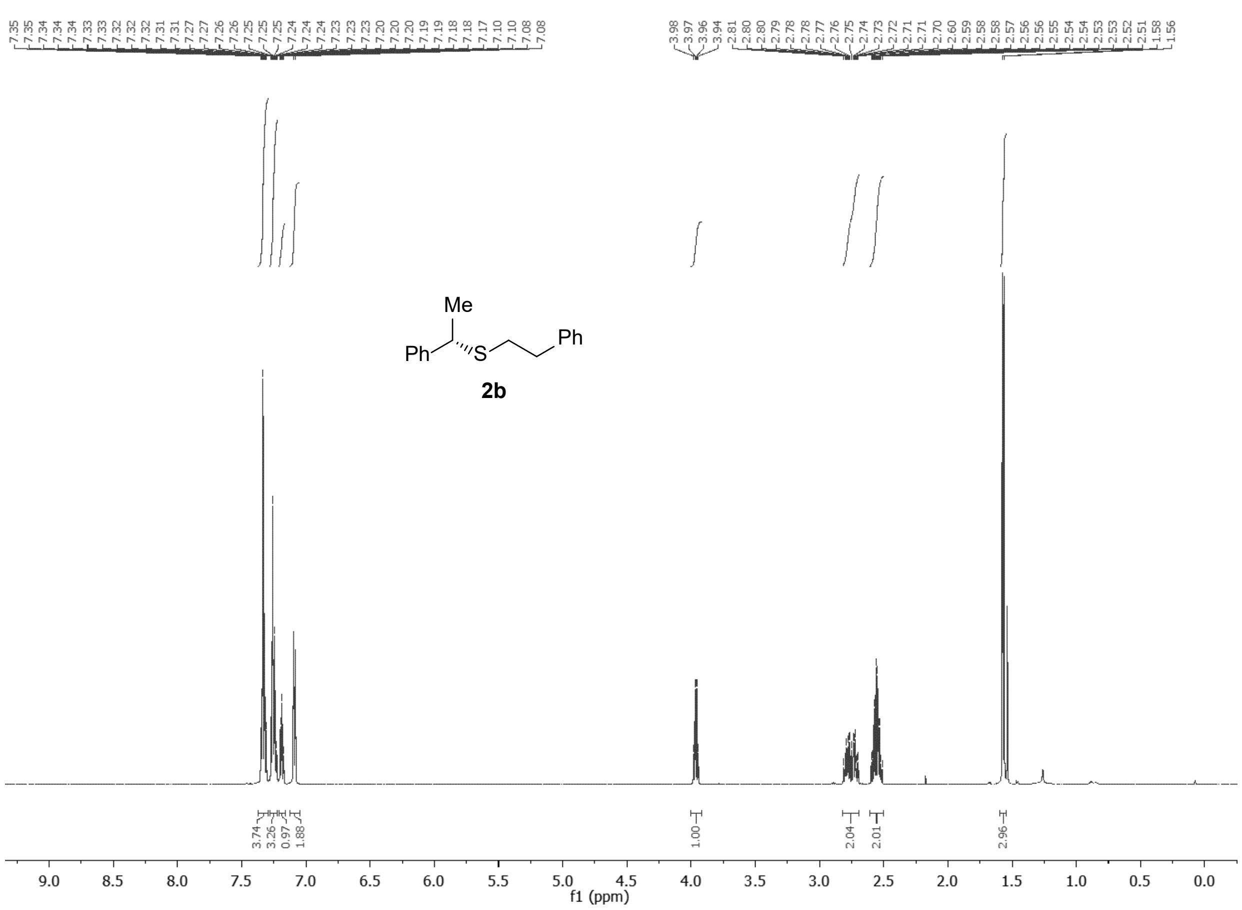
7.35 7.35 7.34 7.34 7.33 7.33 7.32 7.32 7.32 7.31 7.27 7.27 7.26 7.25 7.25 7.25 7.24 7.24 7.24 7.23 7.23 7.23 7.20 7.20 7.20 7.19 7.19 7.18 7.18 7.17 7.10 7.10 7.08 7.08
3.98 3.97 3.96 3.94 2.81 2.80 2.79 2.78 2.78 2.77 2.76 2.75 2.74 2.73 2.72 2.71 2.71 2.70 2.60 2.59 2.58 2.58 2.58 2.57 2.56 2.56 2.55 2.54 2.54 2.53 2.53 2.52 2.51 1.58 1.56
Me
Ph
S
Ph
2b
3.74 3.26 0.97 1.88 1.00 2.04 2.01 2.96
9.0 8.5 8.0 7.5 7.0 6.5 6.0 5.5 5.0 4.5 4.0 3.5 3.0 2.5 2.0 1.5 1.0 0.5 0.0
f1 (ppm)

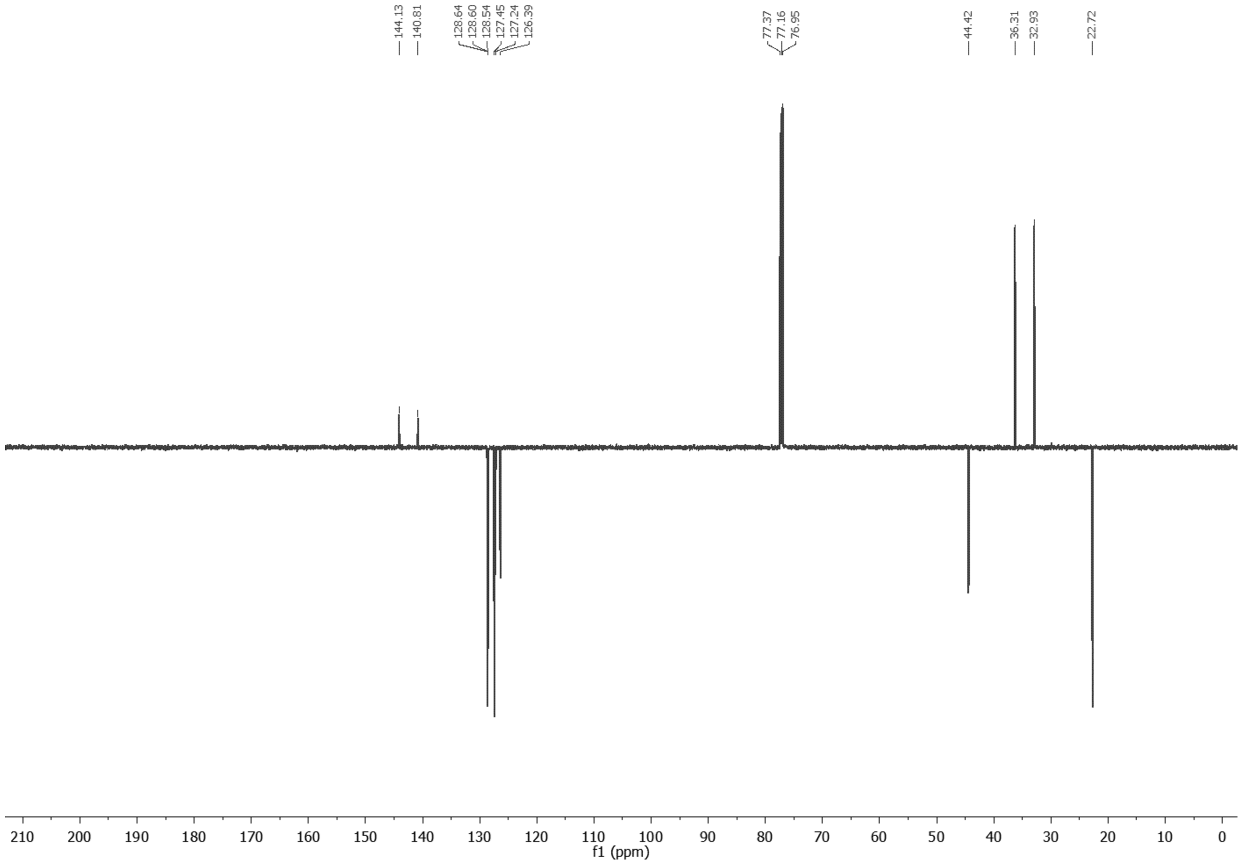
144.13 140.81 128.64 128.60 128.54 127.45 127.24 126.39 77.37 77.16 76.95 44.42 36.31 32.93 22.72
210 200 190 180 170 160 150 140 130 120 110 100 90 80 70 60 50 40 30 20 10 0
f1 (ppm)

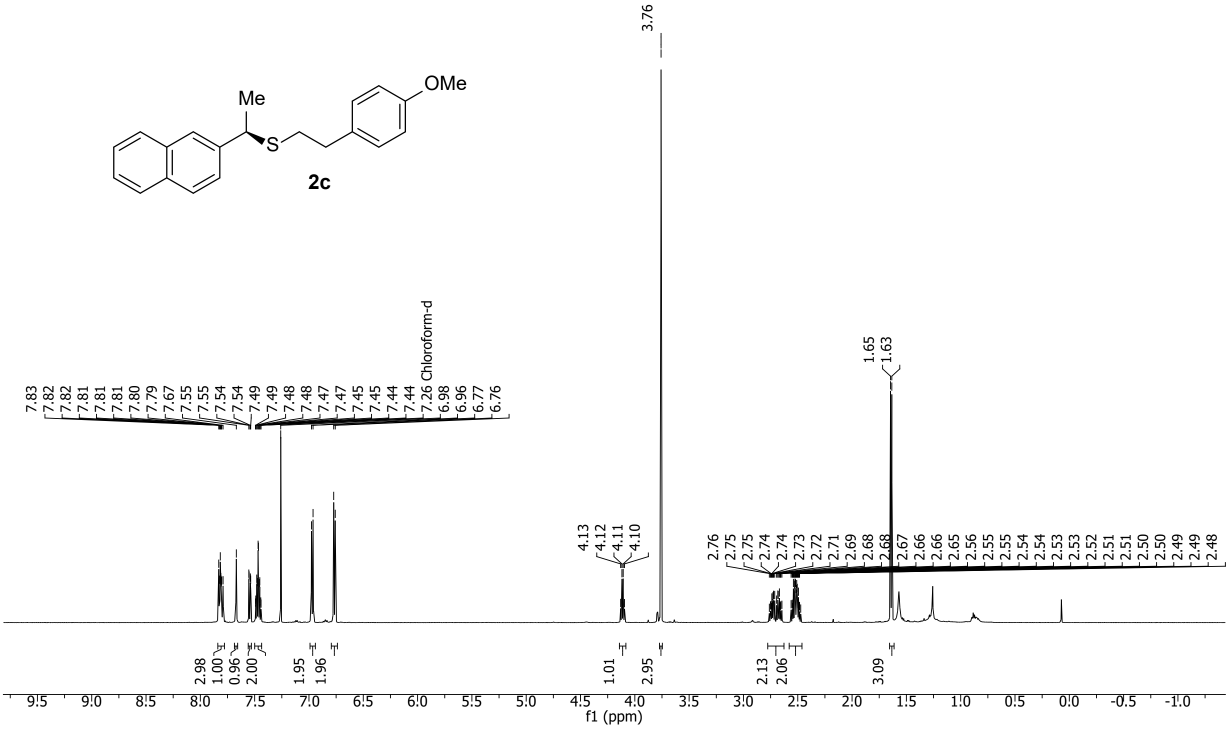

Me
OMe
S
2c
3.76
7.83
7.82
7.82
7.81
7.81
7.81
7.80
7.79
7.67
7.55
7.55
7.54
7.54
7.49
7.48
7.48
7.47
7.47
7.45
7.45
7.44
7.44
7.26 Chloroform-d
6.98
6.96
6.77
6.76
1.65
1.63
4.13
4.12
4.11
4.10
2.76
2.75
2.75
2.74
2.74
2.73
2.72
2.71
2.69
2.68
2.68
2.67
2.66
2.66
2.65
2.56
2.55
2.55
2.54
2.54
2.53
2.53
2.52
2.51
2.51
2.50
2.50
2.49
2.49
2.48
2.98
1.00
0.96
2.00
1.95
1.96
1.01
2.95
2.13
2.06
3.09
9.5 9.0 8.5 8.0 7.5 7.0 6.5 6.0 5.5 5.0 4.5 4.0 3.5 3.0 2.5 2.0 1.5 1.0 0.5 0.0 -0.5 -1.0
f1 (ppm)

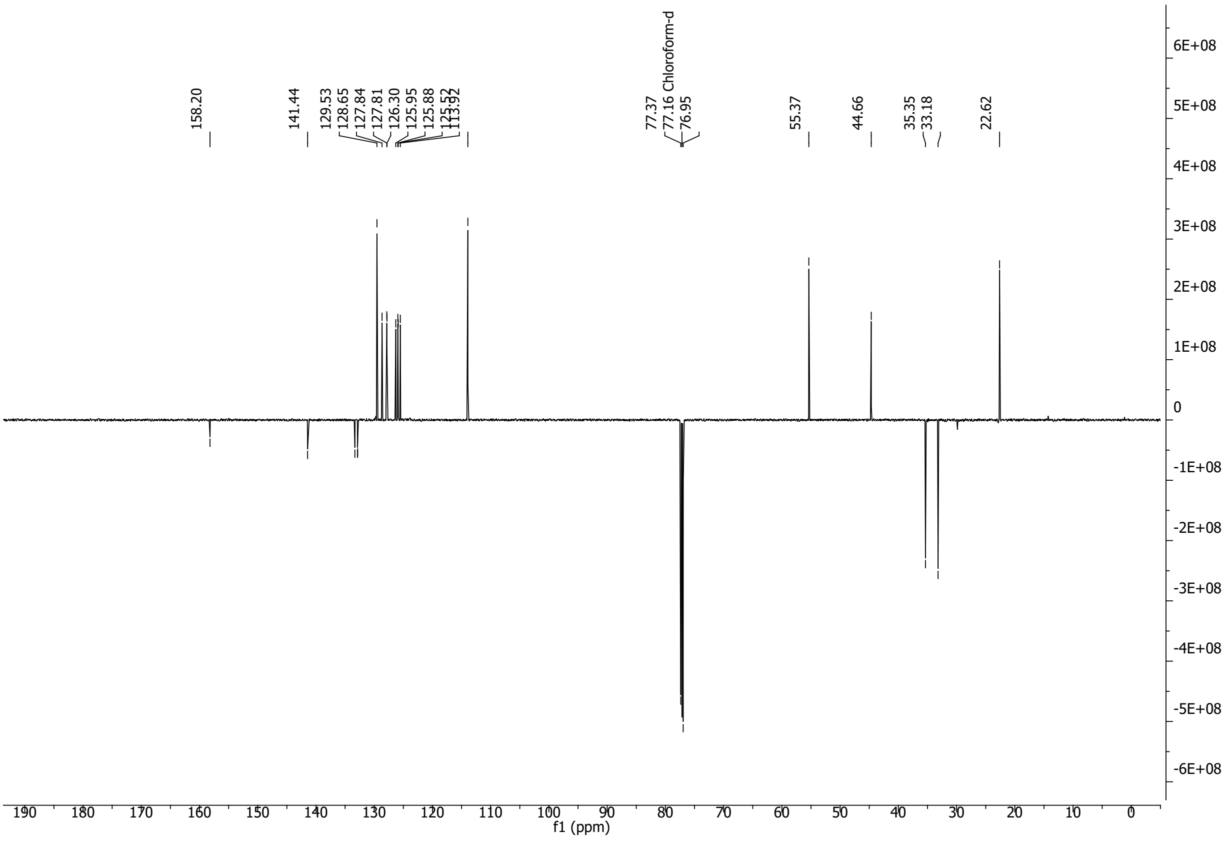

158.20
141.44
129.53
128.65
127.84
127.81
126.30
125.95
125.88
125.52
113.92
77.37
77.16 Chloroform-d
76.95
55.37
44.66
35.35
33.18
22.62
6E+08
5E+08
4E+08
3E+08
2E+08
1E+08
0
-1E+08
-2E+08
-3E+08
-4E+08
-5E+08
-6E+08
190 180 170 160 150 140 130 120 110 100 90 80 70 60 50 40 30 20 10 0
f1 (ppm)

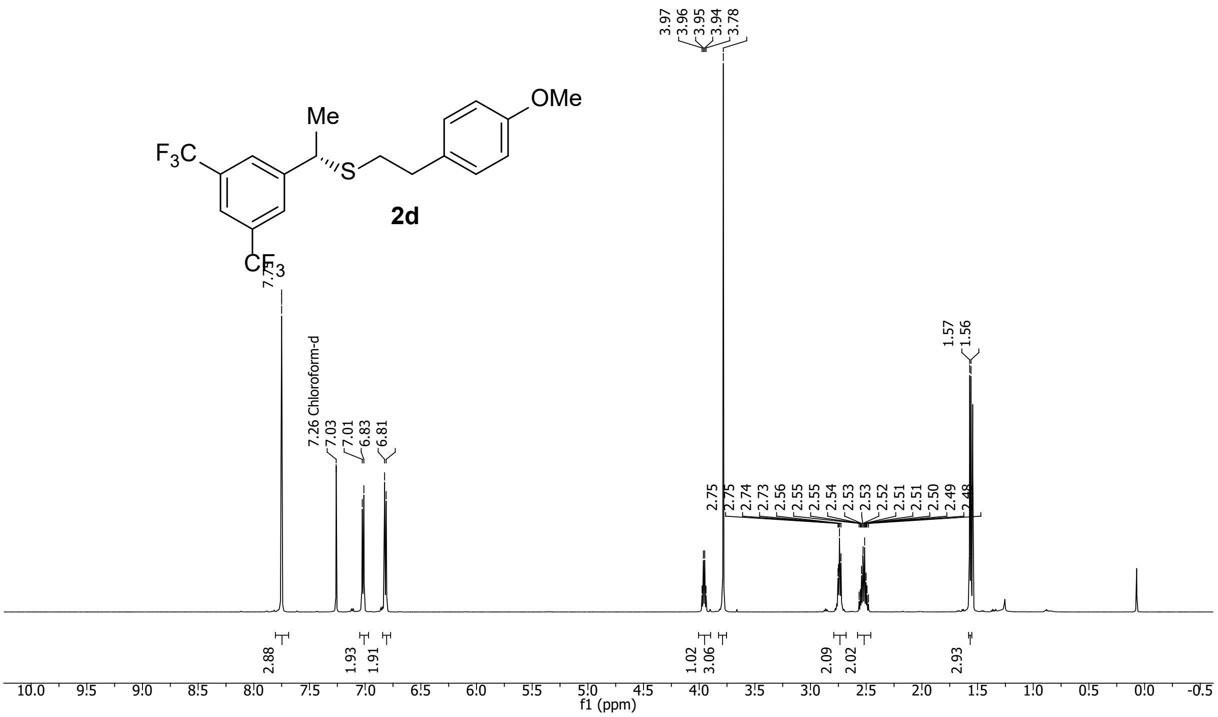

3.97
3.96
3.95
3.94
3.78
OMe
Me
F3C
S
2d
CF3
7.73
7.26 Chloroform-d
7.03
7.01
6.83
6.81
1.57
1.56
2.75
2.75
2.74
2.73
2.56
2.55
2.55
2.54
2.53
2.53
2.52
2.51
2.51
2.50
2.49
2.48
2.88
1.93
1.91
1.02
3.06
2.09
2.02
2.93
10.0 9.5 9.0 8.5 8.0 7.5 7.0 6.5 6.0 5.5 5.0 4.5 4.0 3.5 3.0 2.5 2.0 1.5 1.0 0.5 0.0 -0.5
f1 (ppm)

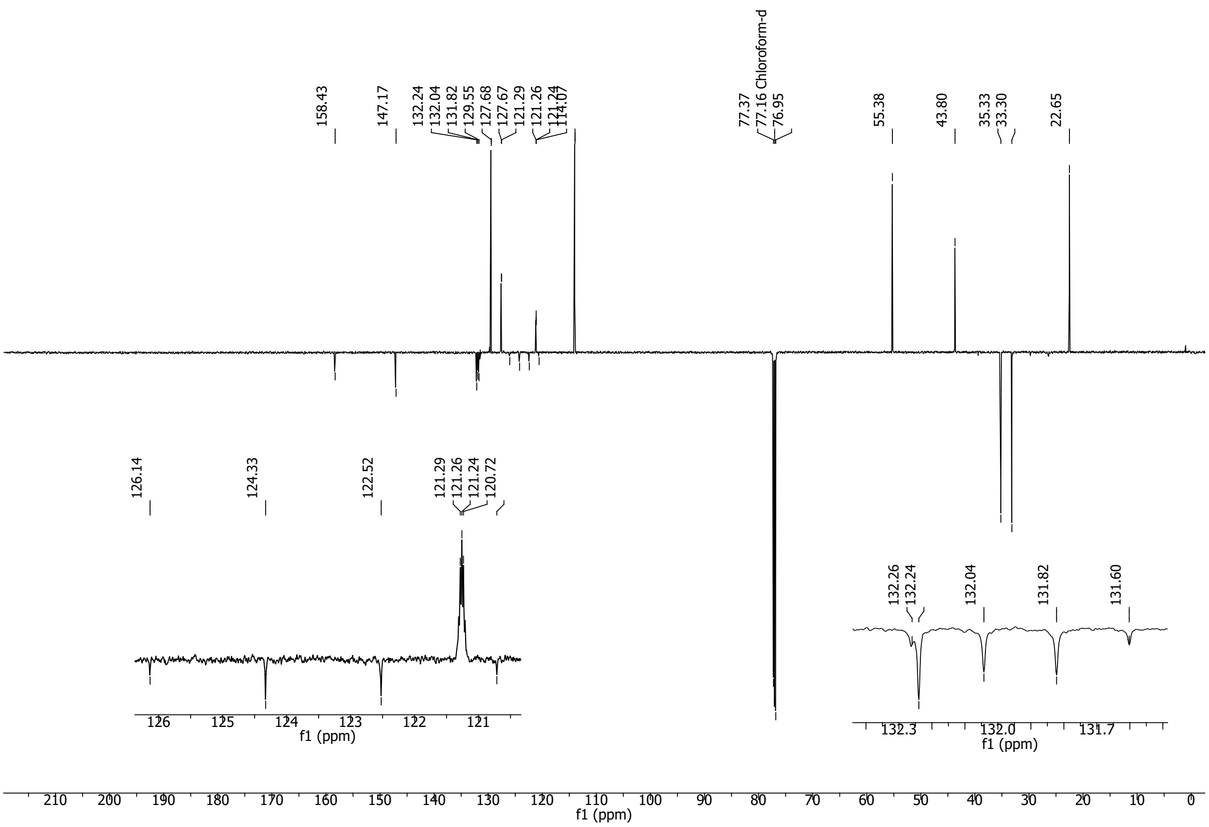

77.37
77.16 Chloroform-d
76.95
158.43
147.17
132.24
132.04
131.82
129.55
127.68
127.67
121.29
121.26
121.24
114.07
55.38
43.80
35.33
33.30
22.65
126.14
124.33
122.52
121.29
121.26
121.24
120.72
126 125 124 123 122 121
f1 (ppm)
132.26
132.24
132.04
131.82
131.60
132.3 132.0 131.7
f1 (ppm)
210 200 190 180 170 160 150 140 130 120 110 100 90 80 70 60 50 40 30 20 10 0
f1 (ppm)

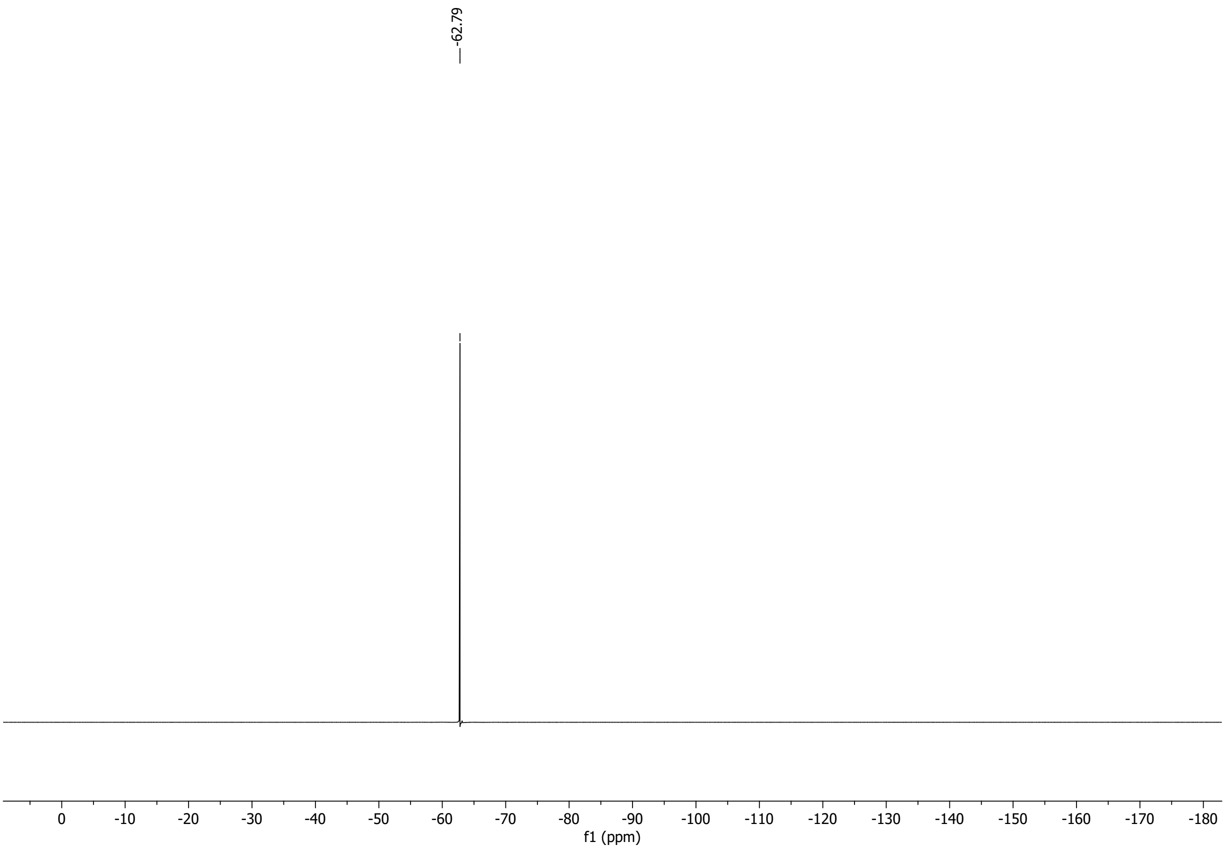
-62.79
0
-10
-20
-30
-40
-50
-60
-70
-80
-90
-100
-110
-120
-130
-140
-150
-160
-170
-180
f1 (ppm)

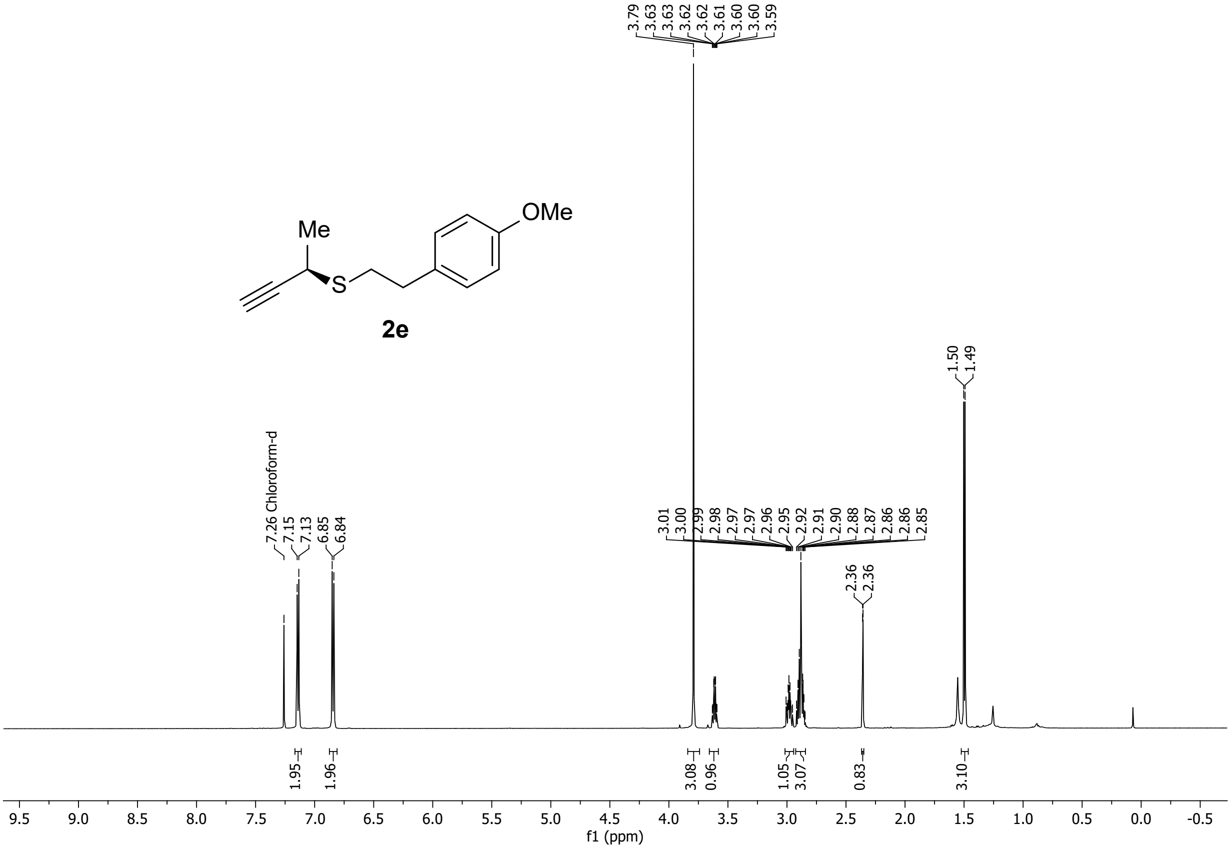
3.79 3.63 3.63 3.62 3.62 3.61 3.60 3.60 3.59
Me
OMe
S
7.26 Chloroform-d 7.15 7.13 6.85 6.84
3.01 3.00 2.99 2.98 2.97 2.97 2.96 2.95 2.92 2.91 2.90 2.88 2.87 2.86 2.86 2.85
2.36 2.36
1.50 1.49
1.95 1.96 3.08 0.96 1.05 3.07 0.83 3.10
9.5 9.0 8.5 8.0 7.5 7.0 6.5 6.0 5.5 5.0 4.5 4.0 3.5 3.0 2.5 2.0 1.5 1.0 0.5 0.0 -0.5
f1 (ppm)

2e

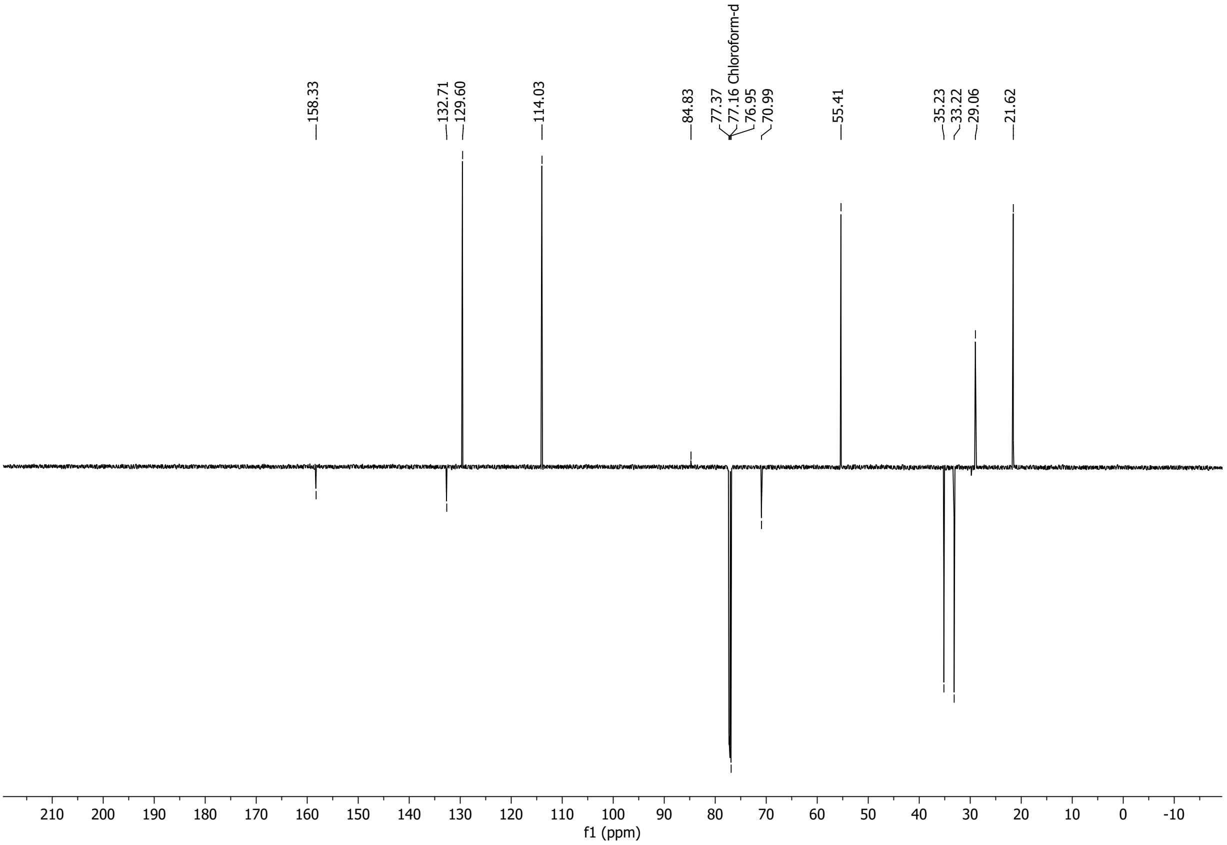
158.33
132.71 129.60
114.03
84.83
77.37 77.16 Chloroform-d 76.95 70.99
55.41
35.23 33.22 29.06
21.62
210 200 190 180 170 160 150 140 130 120 110 100 90 80 70 60 50 40 30 20 10 0 -10
f1 (ppm)

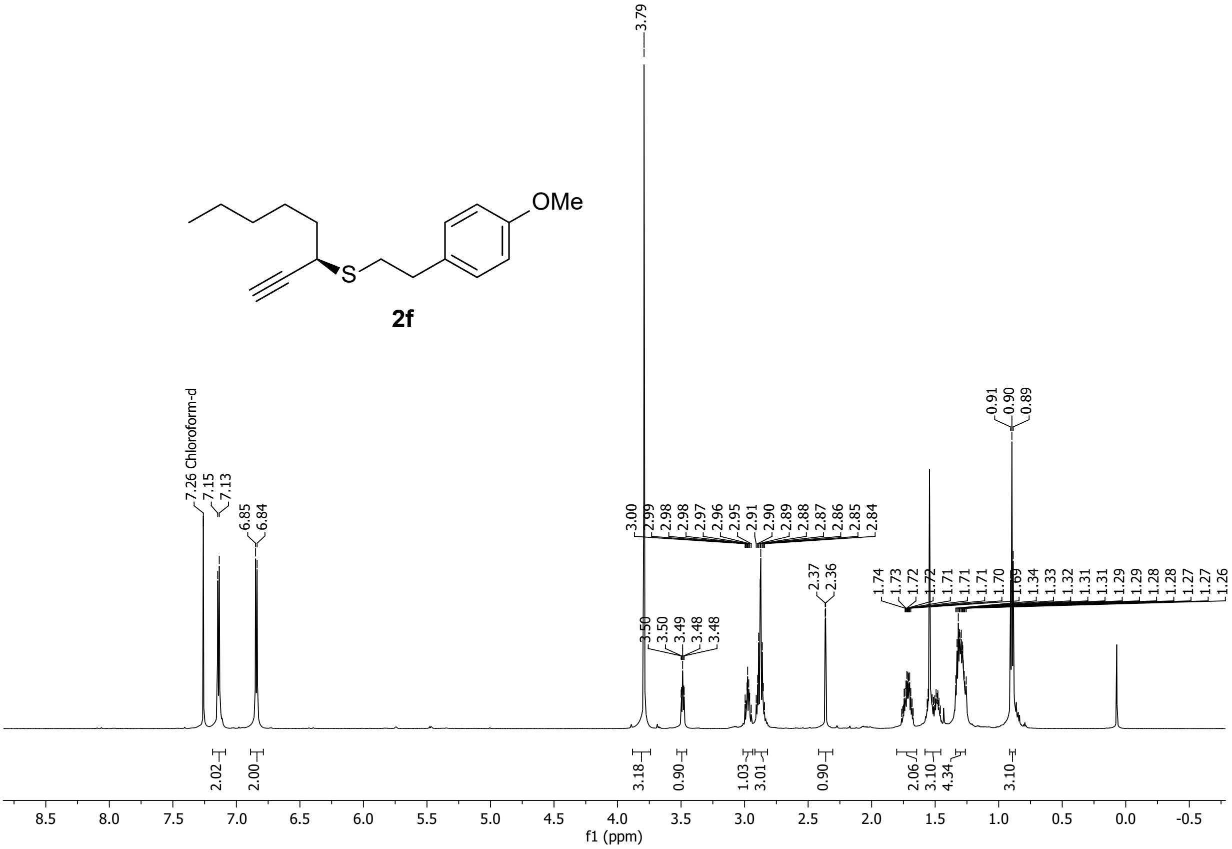
OMe
S
2f
7.26 Chloroform-d
7.15
7.13
6.85
6.84
3.79
3.50
3.50
3.49
3.48
3.48
3.00
2.99
2.98
2.98
2.97
2.96
2.95
2.91
2.90
2.89
2.88
2.87
2.86
2.85
2.84
2.37
2.36
1.74
1.73
1.72
1.72
1.71
1.71
1.71
1.70
1.69
1.34
1.33
1.32
1.31
1.31
1.29
1.29
1.28
1.28
1.27
1.27
1.26
0.91
0.90
0.89
2.02
2.00
3.18
0.90
1.03
3.01
0.90
2.06
3.10
4.34
3.10
8.5
8.0
7.5
7.0
6.5
6.0
5.5
5.0
4.5
4.0
3.5
3.0
2.5
2.0
1.5
1.0
0.5
0.0
-0.5
f1 (ppm)

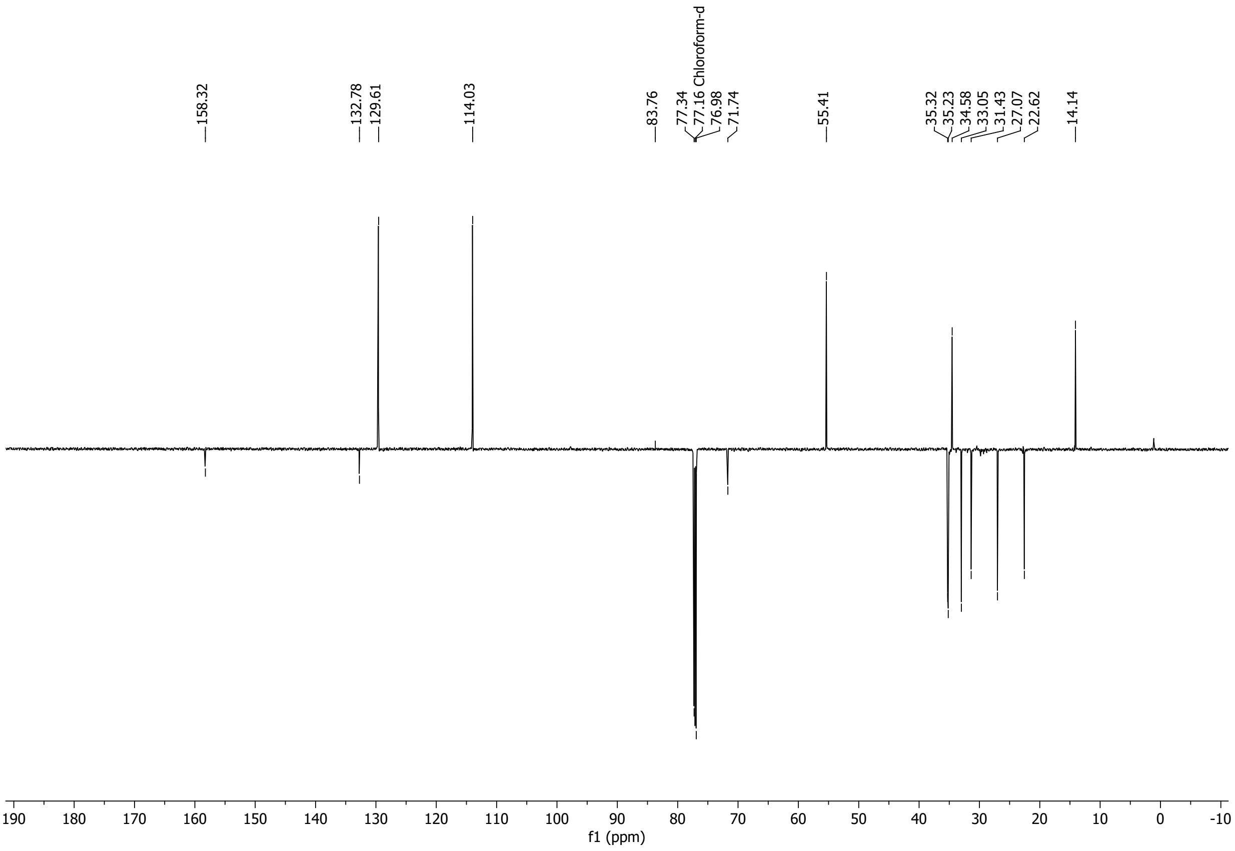
158.32
132.78
129.61
114.03
83.76
77.34
77.16 Chloroform-d
76.98
71.74
55.41
35.32
35.23
34.58
33.05
31.43
27.07
22.62
14.14
190
180
170
160
150
140
130
120
110
100
90
80
70
60
50
40
30
20
10
0
-10
f1 (ppm)

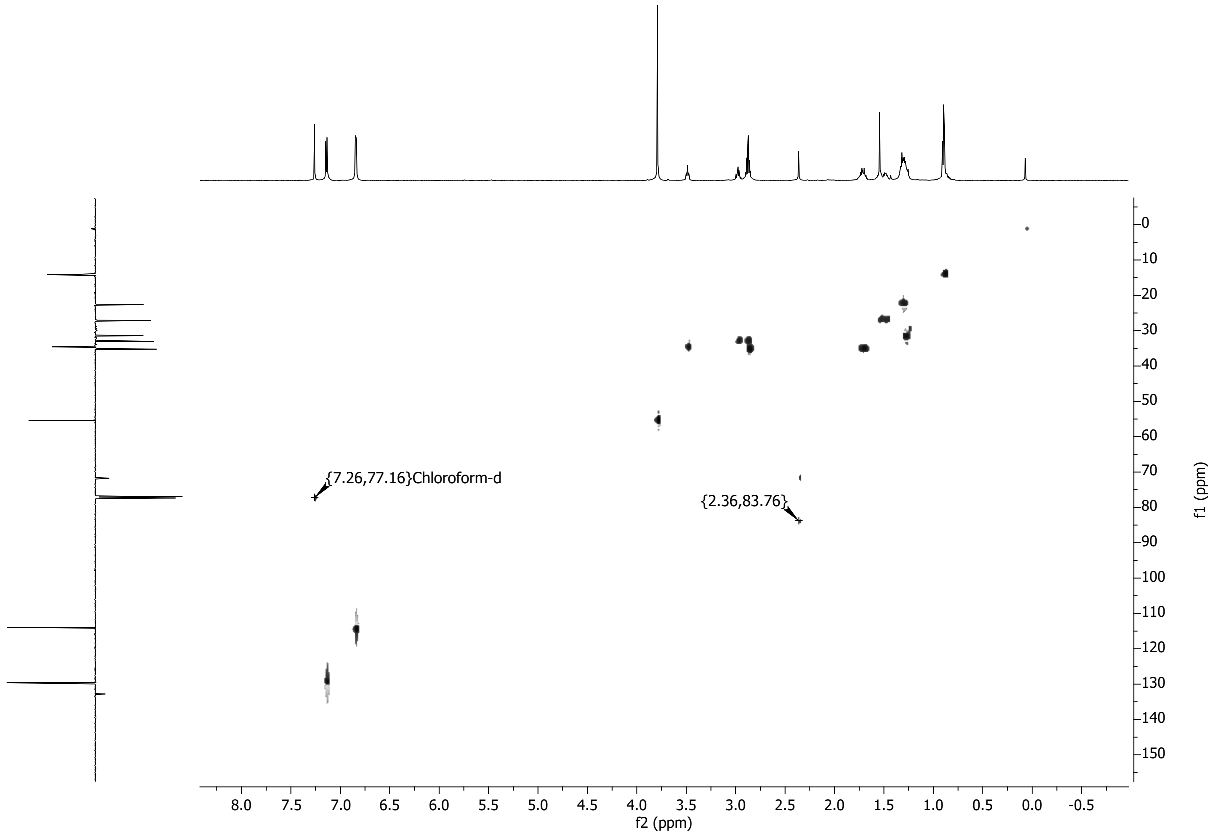
{7.26,77.16}Chloroform-d
{2.36,83.76}
f1 (ppm)
f2 (ppm)
8.0 7.5 7.0 6.5 6.0 5.5 5.0 4.5 4.0 3.5 3.0 2.5 2.0 1.5 1.0 0.5 0.0 -0.5
0 10 20 30 40 50 60 70 80 90 100 110 120 130 140 150

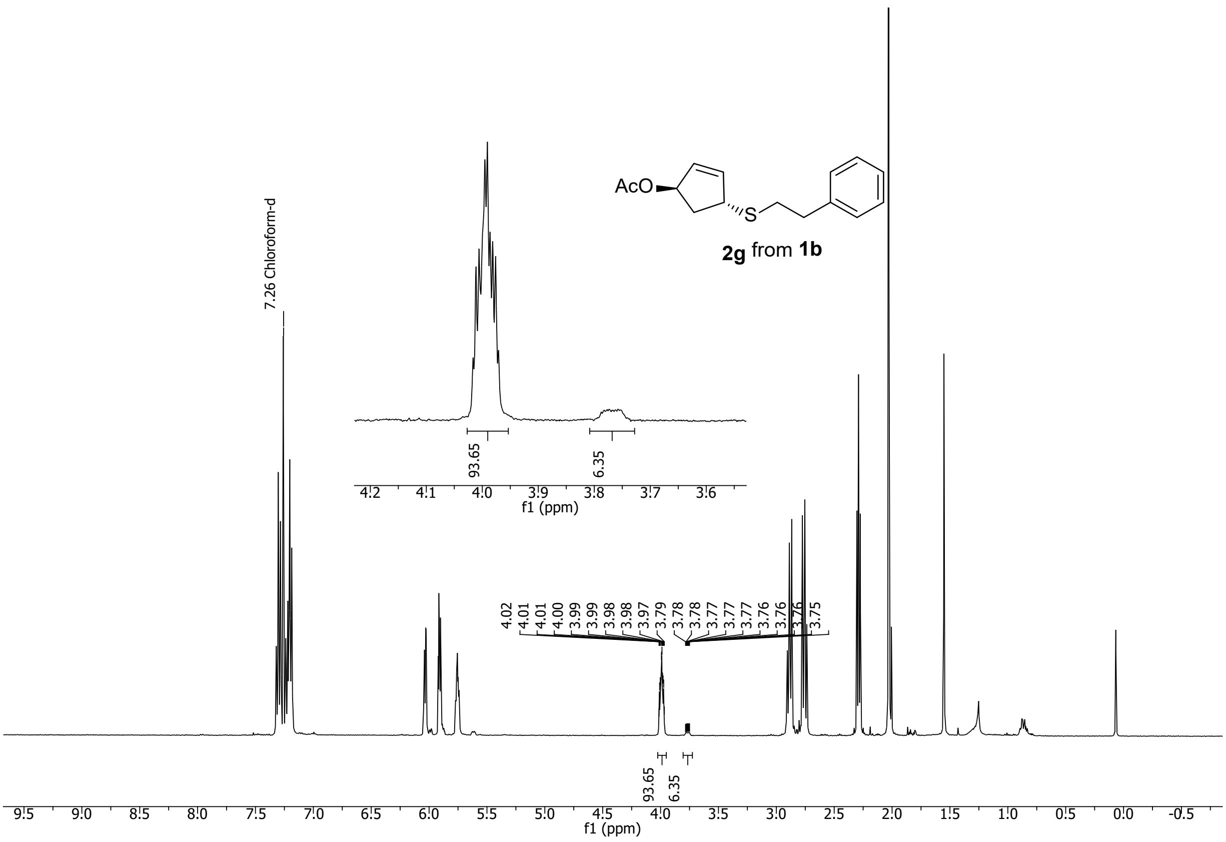
AcO
S
2g from 1b
7.26 Chloroform-d
93.65
6.35
4.02
4.01
4.01
4.00
3.99
3.99
3.98
3.98
3.97
3.79
3.78
3.78
3.77
3.77
3.77
3.76
3.76
3.76
3.75
f1 (ppm)

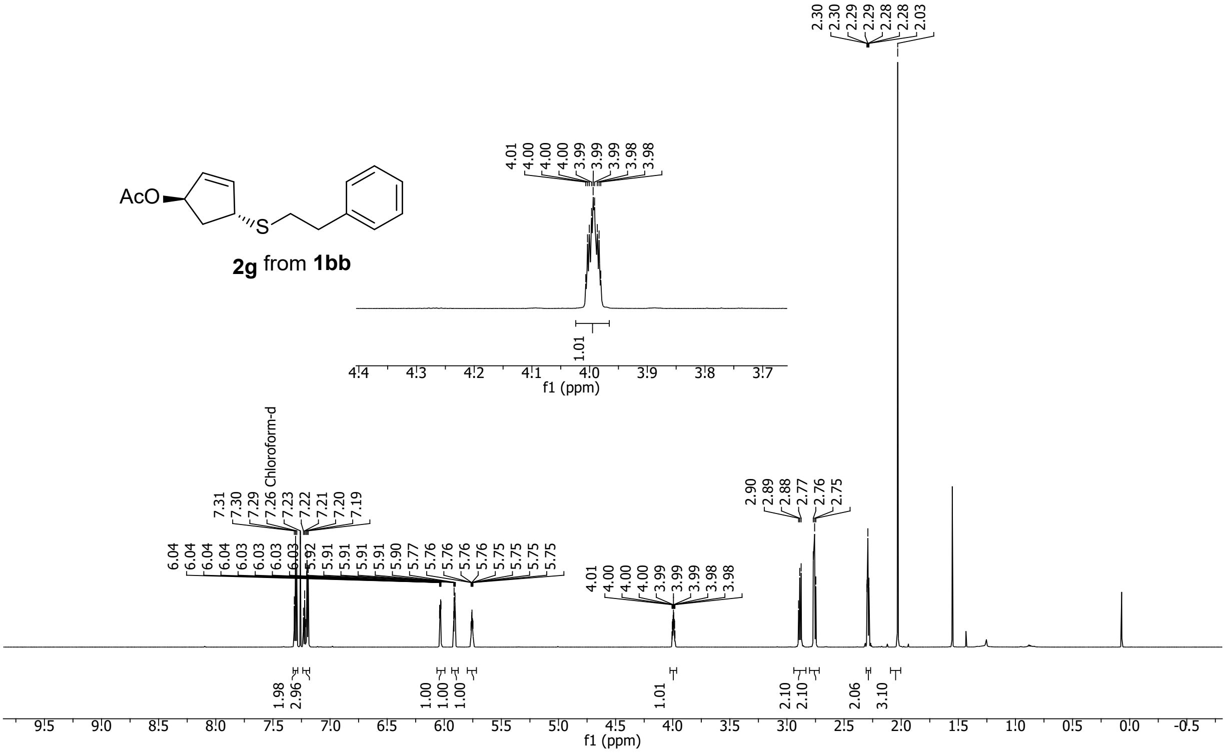
AcO
S
2g from 1bb
2.30
2.30
2.29
2.29
2.28
2.28
2.03
4.01
4.00
4.00
3.99
3.99
3.99
3.98
3.98
1.01
7.31
7.30
7.29
7.26 Chloroform-d
7.23
7.22
7.21
7.20
7.19
6.04
6.04
6.04
6.03
6.03
6.03
6.03
5.92
5.91
5.91
5.91
5.91
5.90
5.77
5.76
5.76
5.76
5.76
5.75
5.75
5.75
5.75
2.90
2.89
2.88
2.77
2.76
2.75
1.98
2.96
1.00
1.00
1.00
2.10
2.10
2.06
3.10
f1 (ppm)

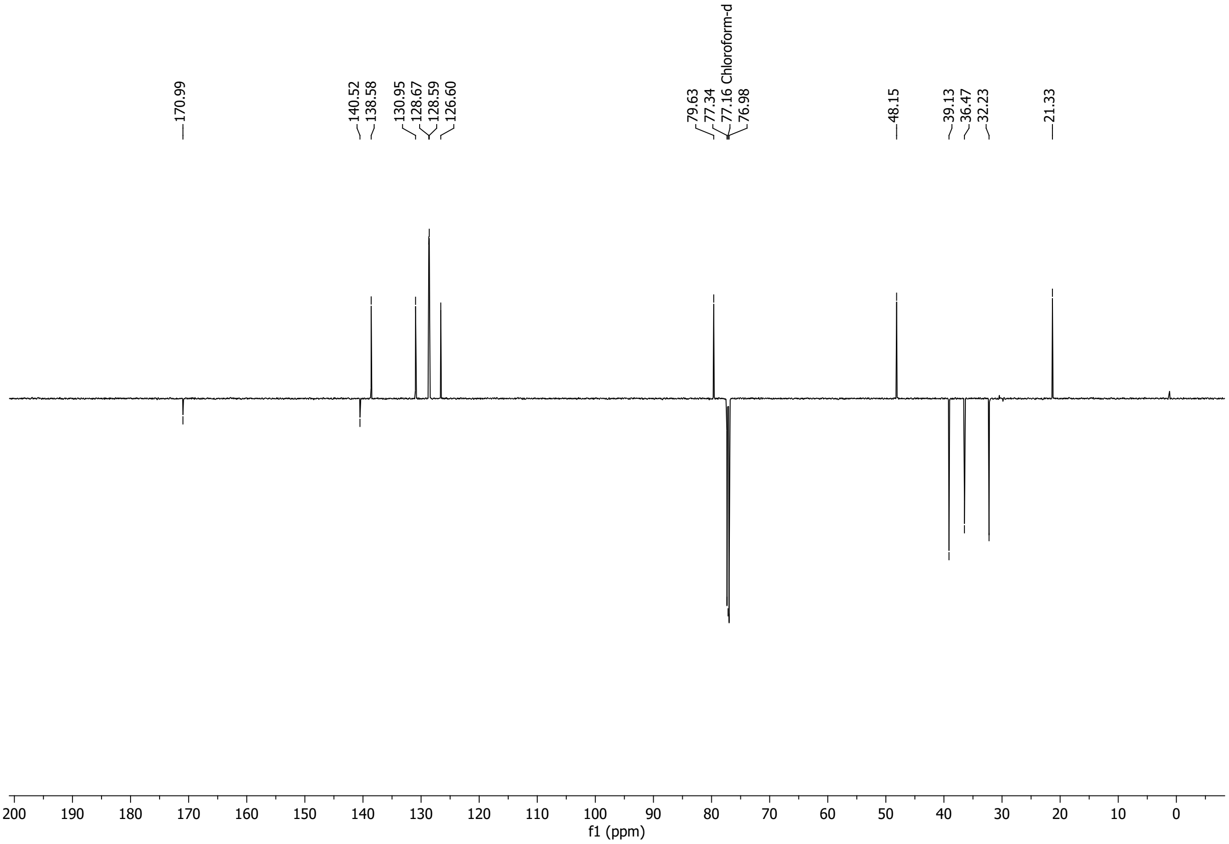
170.99
140.52
138.58
130.95
128.67
128.59
126.60
79.63
77.34
77.16 Chloroform-d
76.98
48.15
39.13
36.47
32.23
21.33
f1 (ppm)

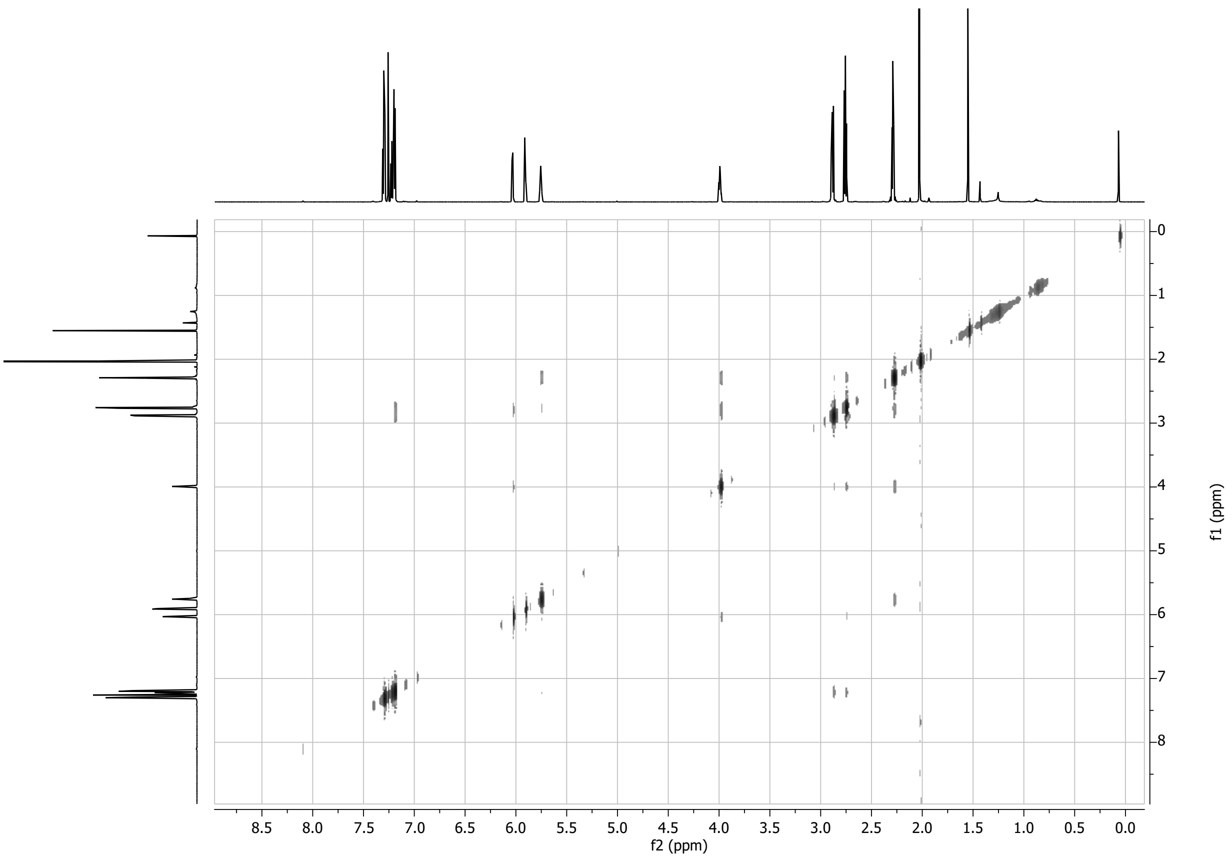
f2 (ppm)
f1 (ppm)

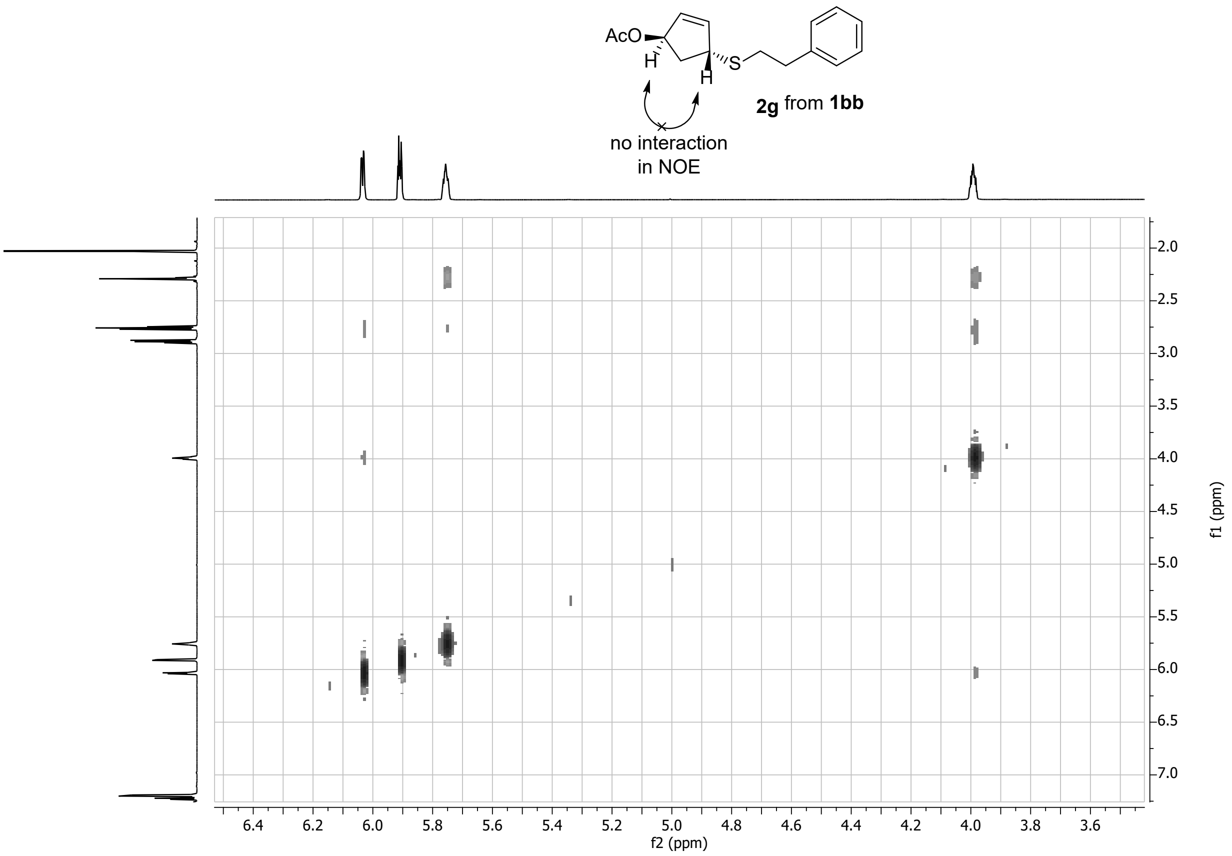

AcO
H
H
S
2g from 1bb
no interaction
in NOE
f2 (ppm)
f1 (ppm)

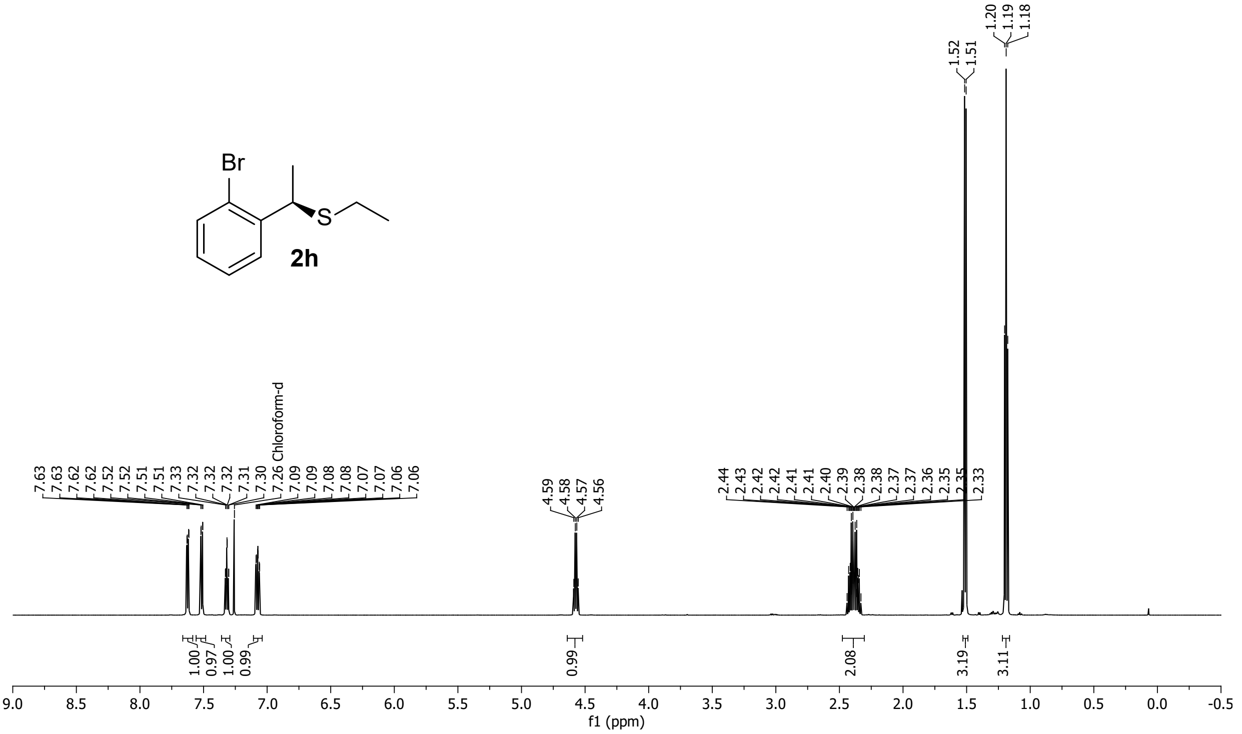
Br
S
2h
7.63
7.63
7.62
7.62
7.52
7.52
7.51
7.51
7.33
7.32
7.32
7.32
7.31
7.30
7.26 Chloroform-d
7.09
7.09
7.08
7.08
7.07
7.07
7.06
7.06
4.59
4.58
4.57
4.56
2.44
2.43
2.42
2.42
2.41
2.41
2.40
2.39
2.38
2.38
2.37
2.37
2.36
2.35
2.35
2.33
1.52
1.51
1.20
1.19
1.18
1.00
0.97
1.00
0.99
0.99
2.08
3.19
3.11
9.0 8.5 8.0 7.5 7.0 6.5 6.0 5.5 5.0 4.5 4.0 3.5 3.0 2.5 2.0 1.5 1.0 0.5 0.0 -0.5
f1 (ppm)

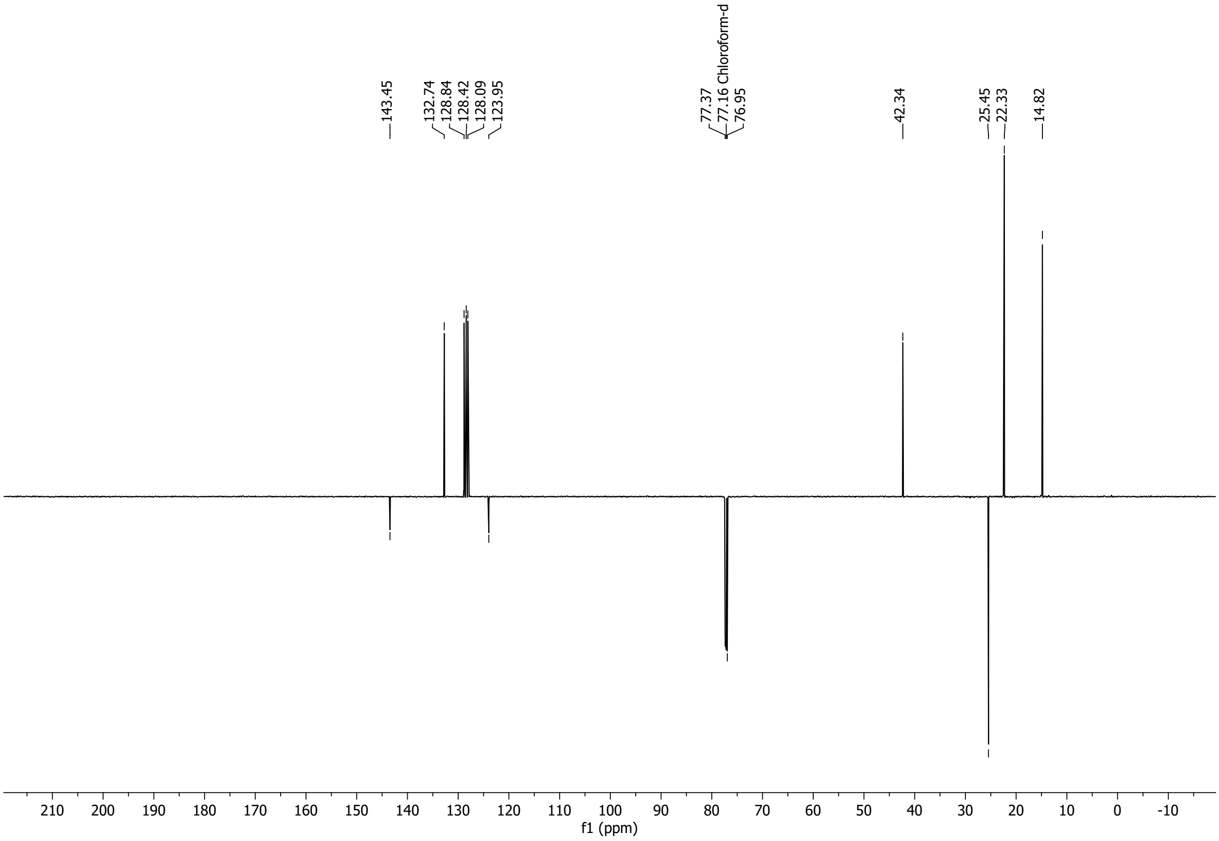
143.45
132.74
128.84
128.42
128.09
123.95
77.37
77.16 Chloroform-d
76.95
42.34
25.45
22.33
14.82
210 200 190 180 170 160 150 140 130 120 110 100 90 80 70 60 50 40 30 20 10 0 -10
f1 (ppm)

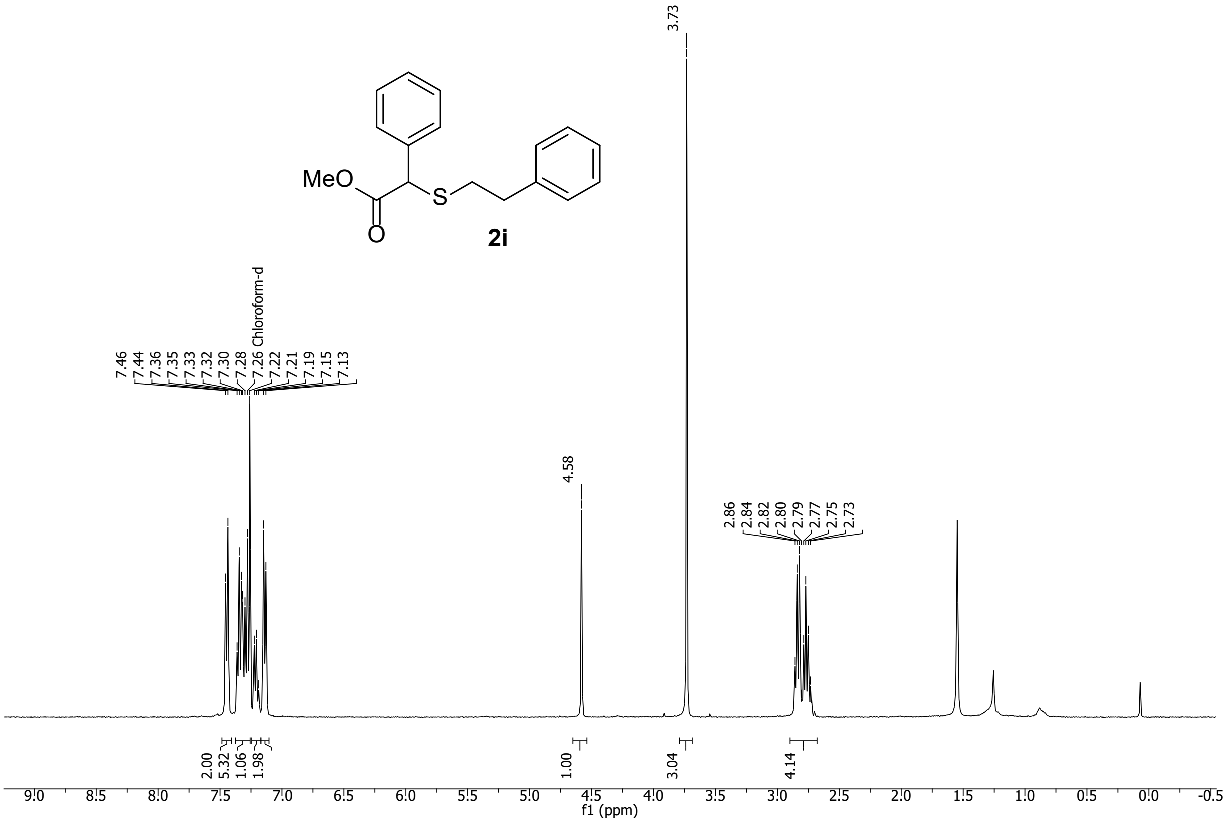
MeO
S
O
2i
3.73
7.46
7.44
7.36
7.35
7.33
7.32
7.30
7.28
7.26 Chloroform-d
7.22
7.21
7.19
7.15
7.13
4.58
2.86
2.84
2.82
2.80
2.79
2.77
2.75
2.73
2.00
5.32
1.06
1.98
1.00
3.04
4.14
9.0 8.5 8.0 7.5 7.0 6.5 6.0 5.5 5.0 4.5 4.0 3.5 3.0 2.5 2.0 1.5 1.0 0.5 0.0 -0.5
f1 (ppm)

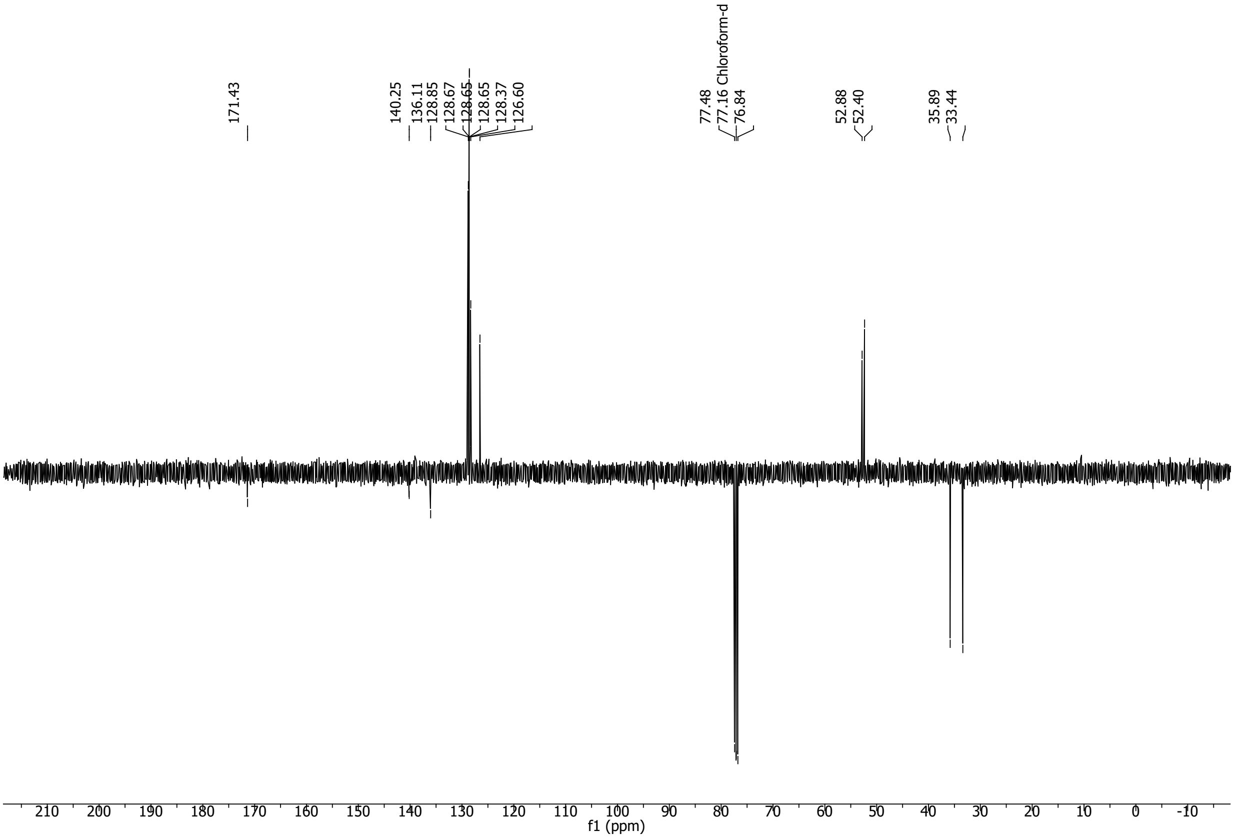
171.43
140.25
136.11
128.85
128.67
128.65
128.65
128.37
126.60
77.48
77.16 Chloroform-d
76.84
52.88
52.40
35.89
33.44
210 200 190 180 170 160 150 140 130 120 110 100 90 80 70 60 50 40 30 20 10 0 -10
f1 (ppm)

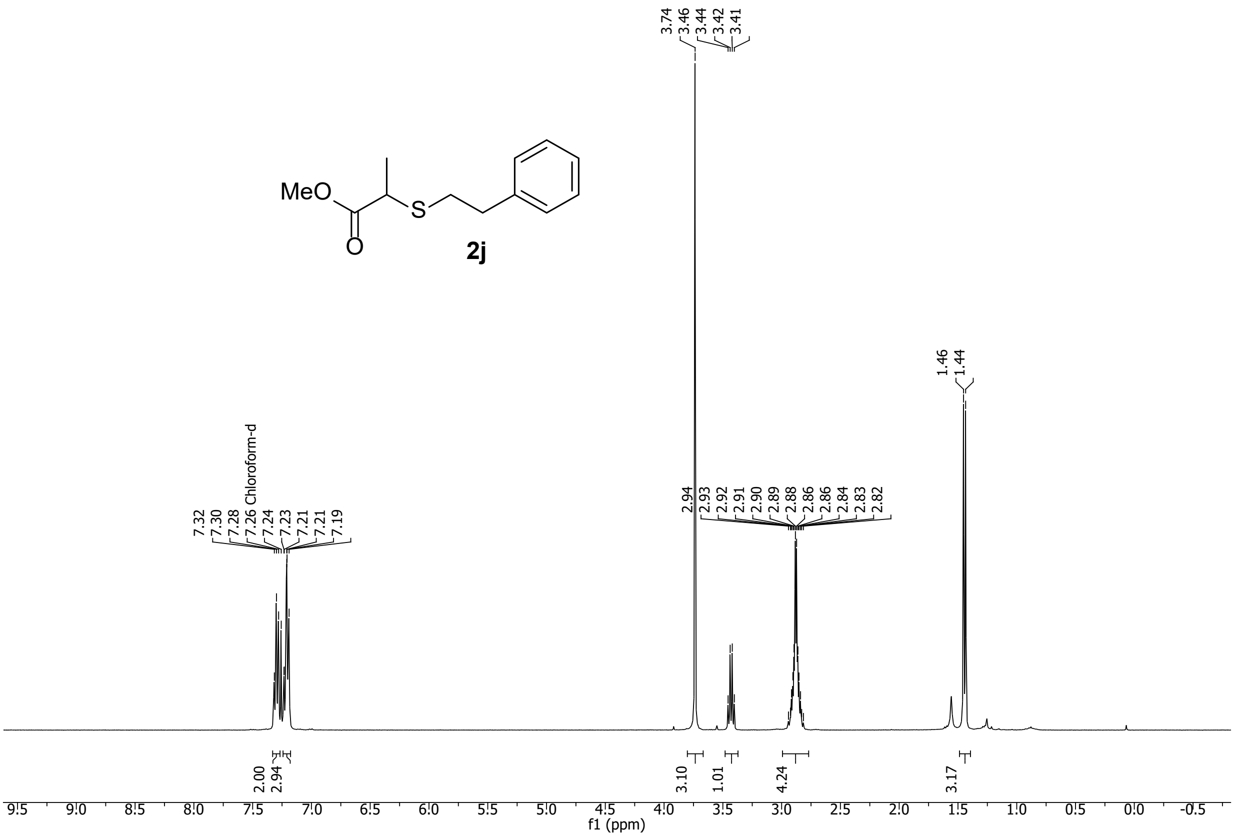
MeO
S
O
2j
3.74
3.46
3.44
3.42
3.41
7.32
7.30
7.28
7.26 Chloroform-d
7.24
7.23
7.21
7.21
7.19
2.94
2.93
2.92
2.91
2.90
2.89
2.88
2.86
2.86
2.84
2.83
2.82
1.46
1.44
2.00
2.94
3.10
1.01
4.24
3.17
f1 (ppm)

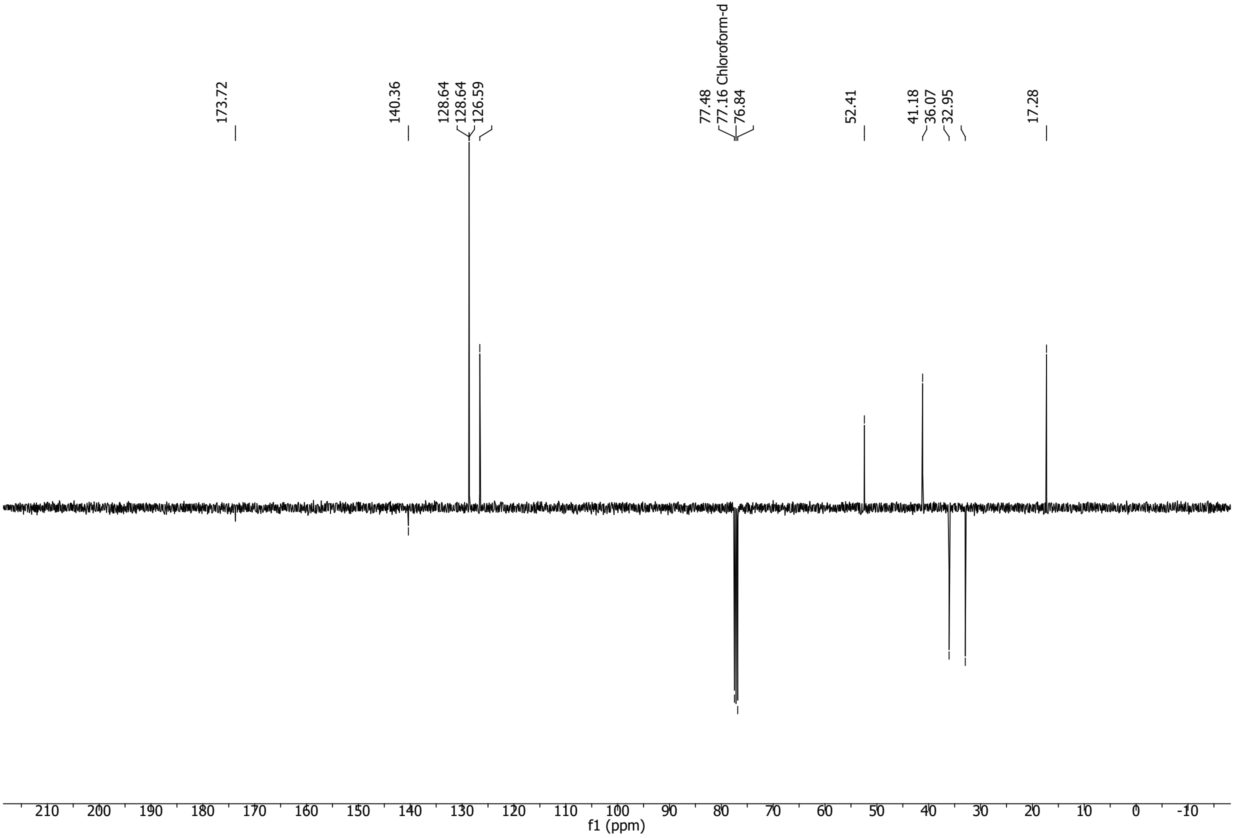
173.72
140.36
128.64
128.64
126.59
77.48
77.16 Chloroform-d
76.84
52.41
41.18
36.07
32.95
17.28
f1 (ppm)

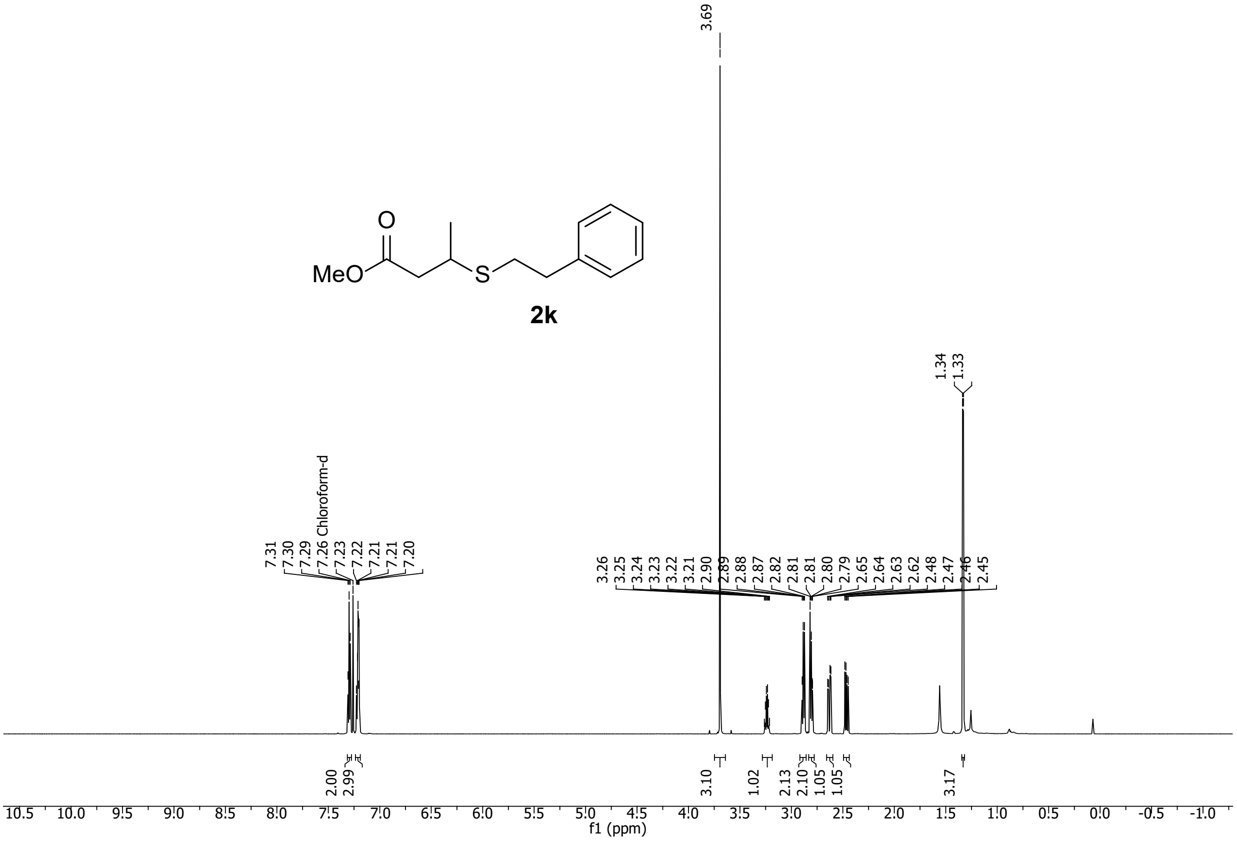
MeO
O
S
2k
3.69
1.34
1.33
7.31
7.30
7.29
7.26 Chloroform-d
7.23
7.22
7.21
7.21
7.20
3.26
3.25
3.24
3.23
3.22
3.21
2.90
2.89
2.88
2.87
2.82
2.81
2.81
2.80
2.79
2.65
2.64
2.63
2.62
2.48
2.47
2.46
2.45
2.00
2.99
3.10
1.02
2.13
2.10
1.05
1.05
3.17
f1 (ppm)

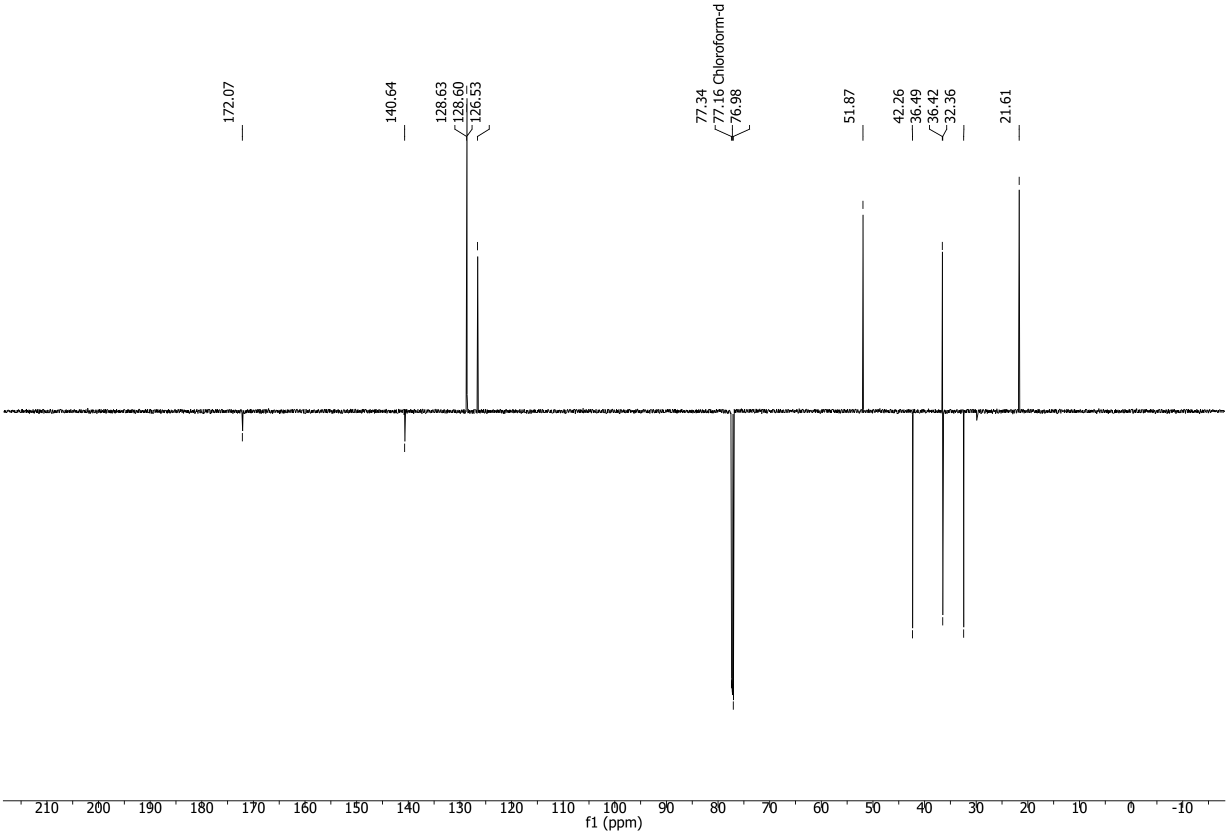
172.07
140.64
128.63
128.60
126.53
77.34
77.16 Chloroform-d
76.98
51.87
42.26
36.49
36.42
32.36
21.61
f1 (ppm)

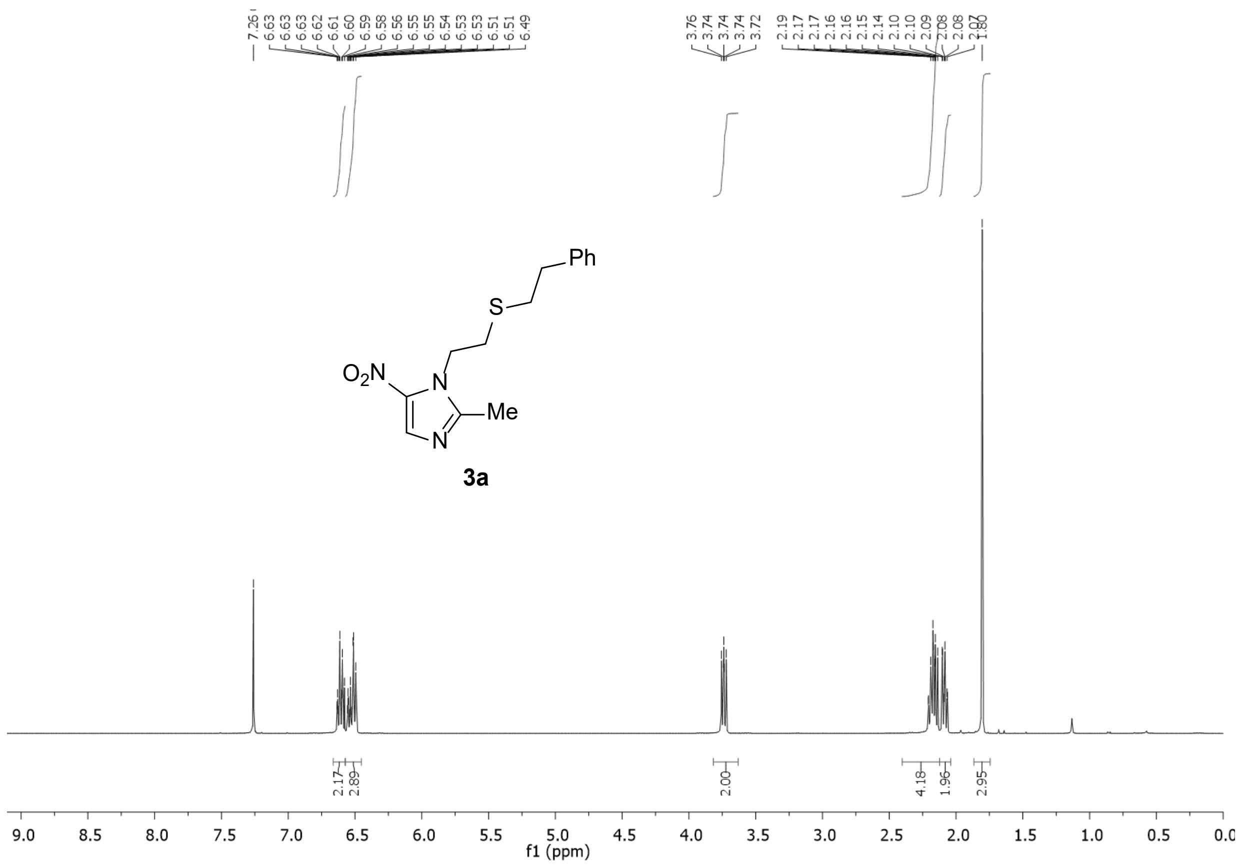
Ph
S
O_2N
N
Me
N
3a
f1 (ppm)

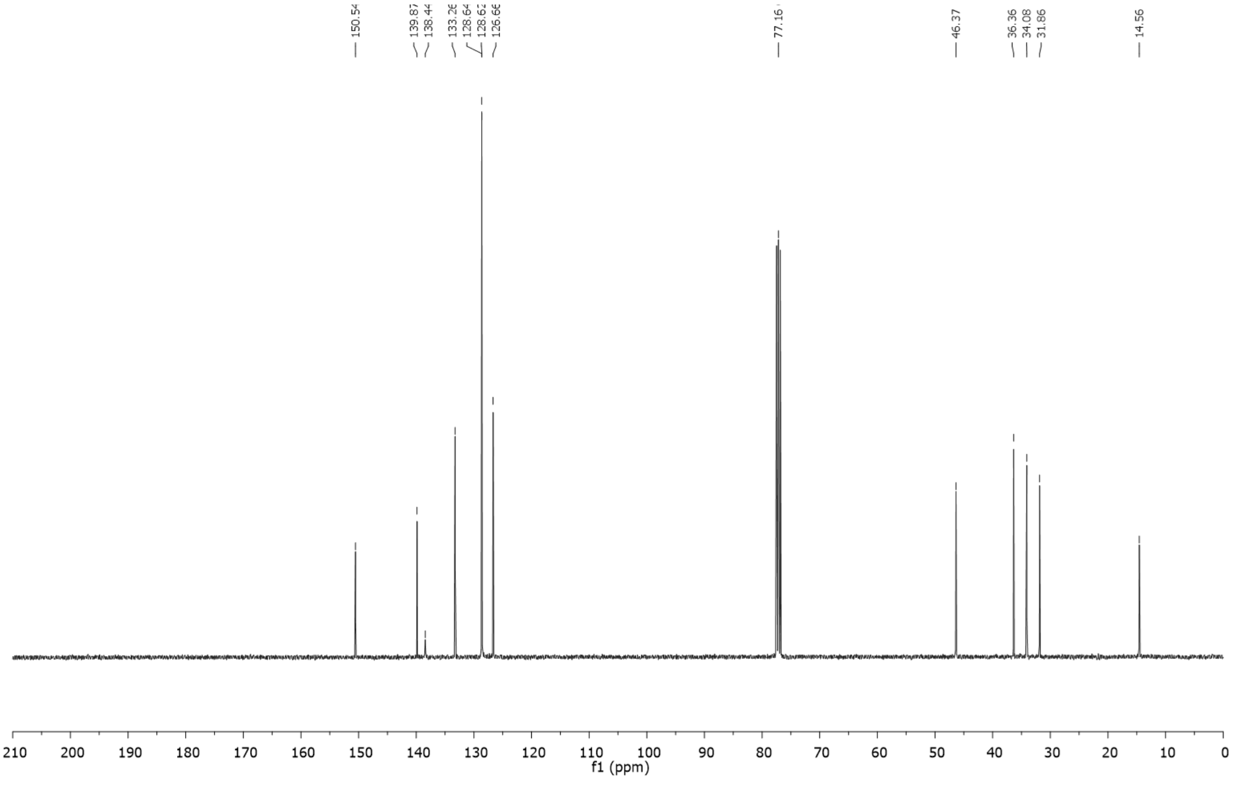
f1 (ppm)

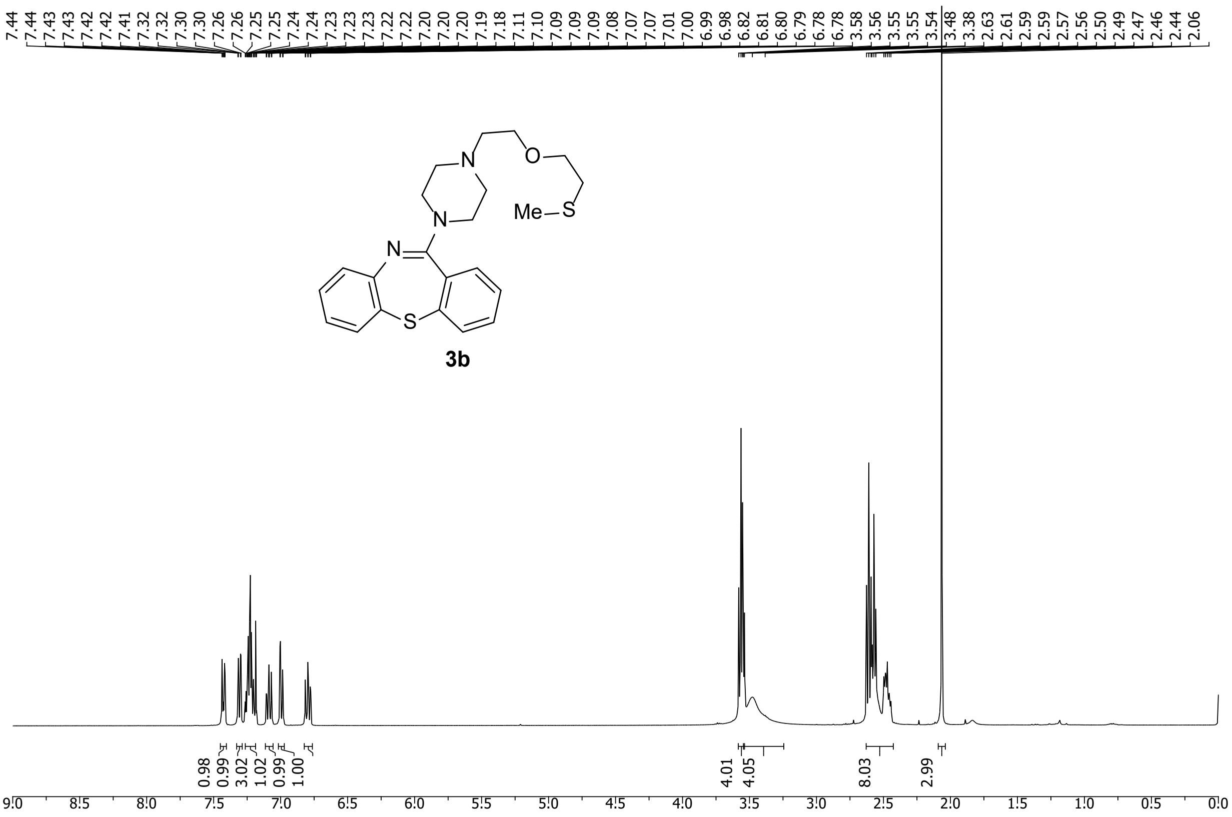
7.44
7.44
7.43
7.43
7.42
7.42
7.41
7.32
7.32
7.30
7.26
7.26
7.25
7.25
7.24
7.24
7.23
7.23
7.23
7.22
7.22
7.20
7.20
7.20
7.19
7.18
7.11
7.10
7.09
7.09
7.09
7.08
7.07
7.07
7.01
7.00
6.99
6.98
6.82
6.81
6.80
6.79
6.78
6.78
3.58
3.56
3.55
3.54
3.48
3.38
2.63
2.61
2.59
2.59
2.57
2.56
2.50
2.49
2.47
2.46
2.44
2.06
N
N
O
Me
S
N
S
3b
0.98
0.99
3.02
1.02
0.99
1.00
4.01
4.05
8.03
2.99
9.0
8.5
8.0
7.5
7.0
6.5
6.0
5.5
5.0
4.5
4.0
3.5
3.0
2.5
2.0
1.5
1.0
0.5
0.0

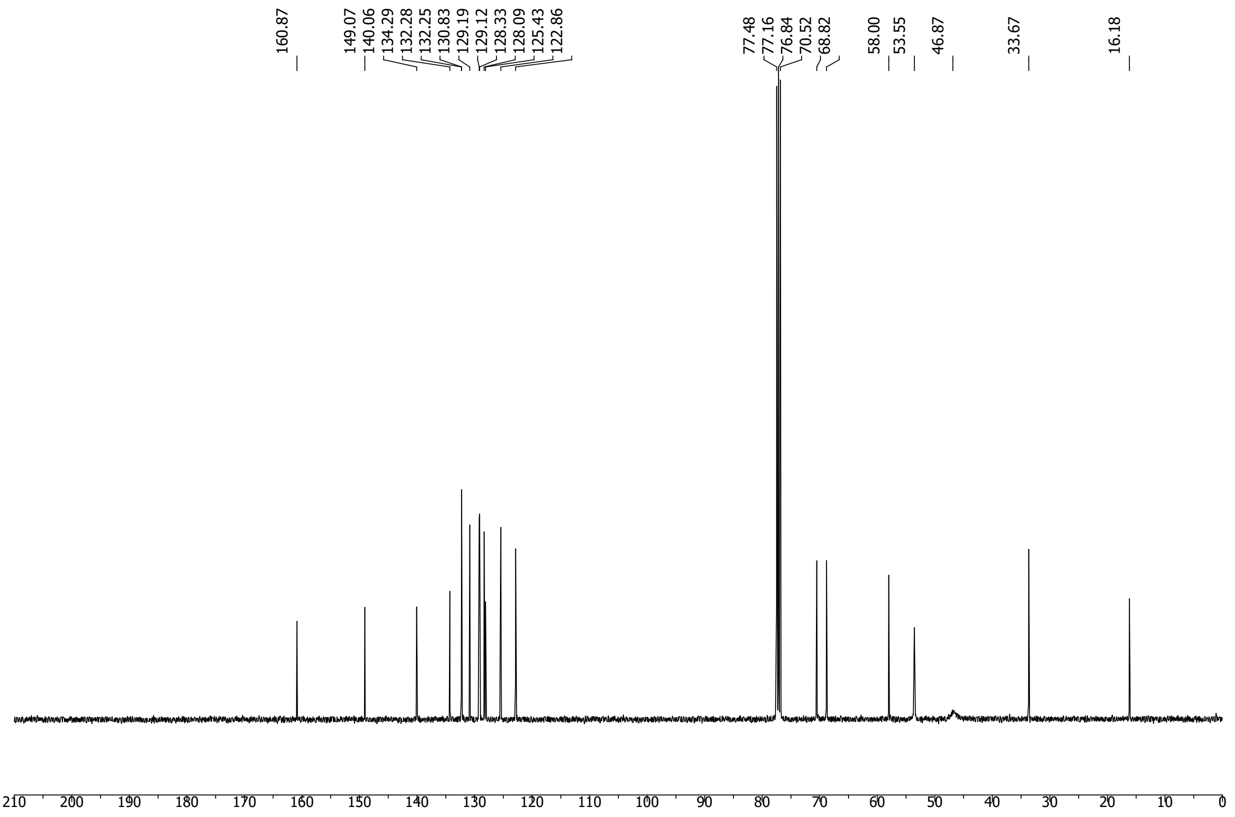
160.87
149.07
140.06
134.29
132.28
132.25
130.83
129.19
129.12
128.33
128.09
125.43
122.86
77.48
77.16
76.84
70.52
68.82
58.00
53.55
46.87
33.67
16.18
210
200
190
180
170
160
150
140
130
120
110
100
90
80
70
60
50
40
30
20
10
0

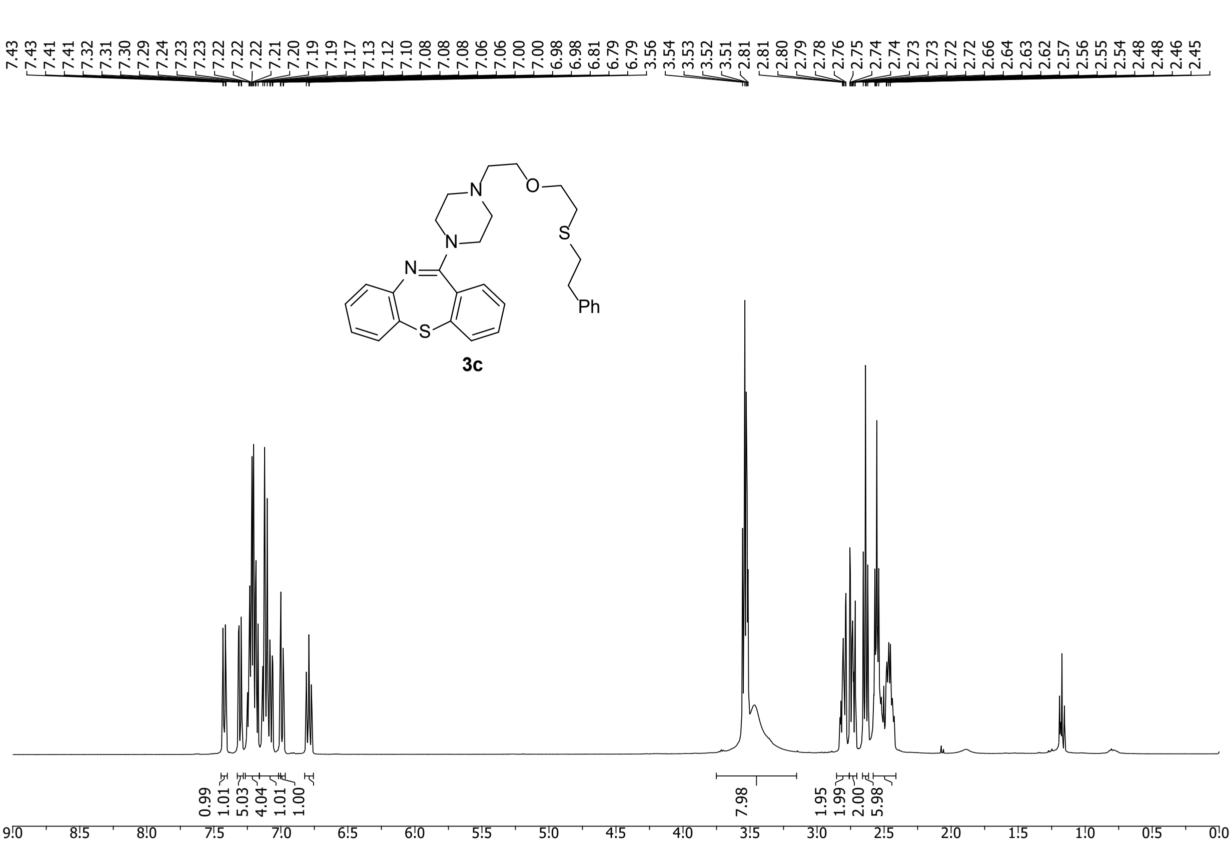
7.43 7.43 7.41 7.41 7.32 7.31 7.30 7.29 7.24 7.23 7.23 7.22 7.22 7.22 7.21 7.20 7.19 7.19 7.17 7.13 7.12 7.10 7.08 7.08 7.08 7.06 7.06 7.00 7.00 6.98 6.81 6.79 3.56 3.54 3.53 3.52 3.51 2.81 2.81 2.80 2.79 2.78 2.76 2.75 2.74 2.74 2.73 2.73 2.72 2.72 2.66 2.64 2.63 2.62 2.57 2.56 2.55 2.54 2.48 2.46 2.45
N
O
S
S
Ph
3c
0.99
1.01
5.03
4.04
1.01
1.00
7.98
1.95
1.99
2.00
5.98
9.0 8.5 8.0 7.5 7.0 6.5 6.0 5.5 5.0 4.5 4.0 3.5 3.0 2.5 2.0 1.5 1.0 0.5 0.0

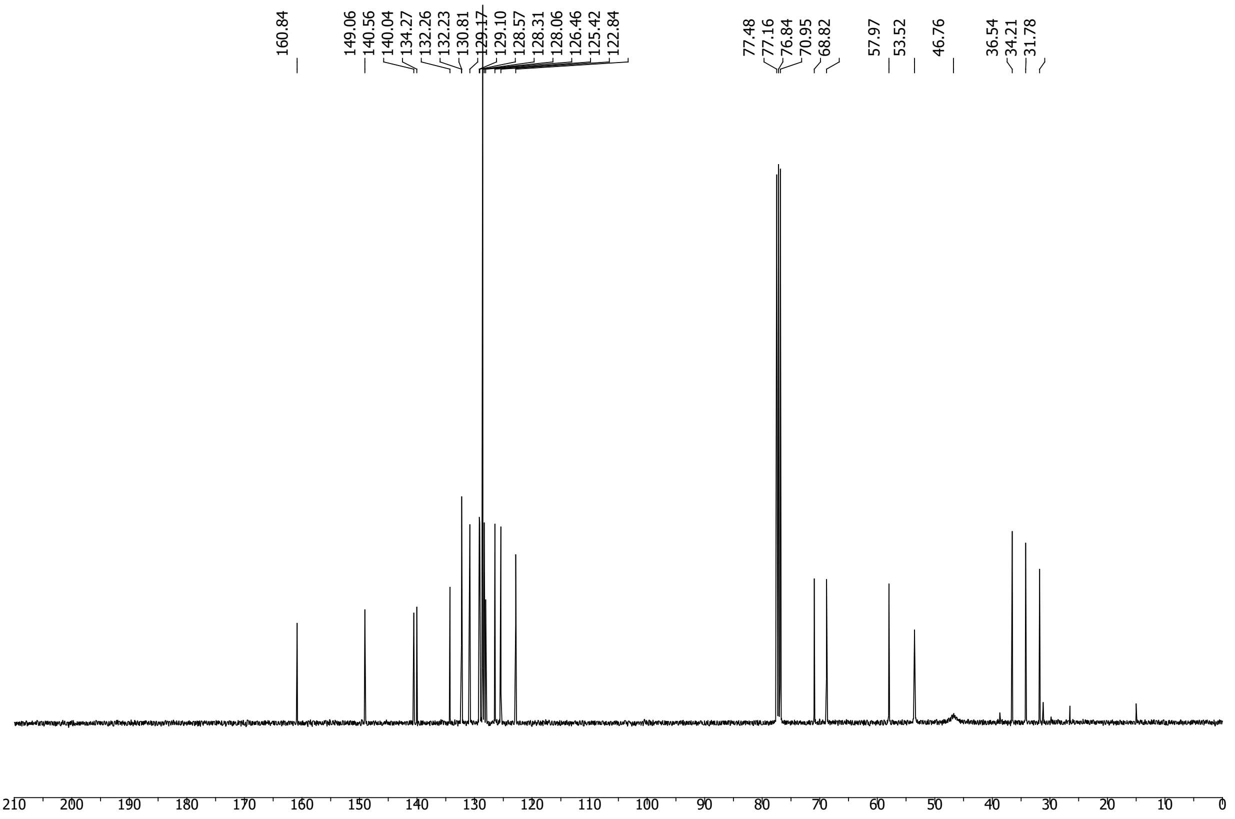
160.84
149.06 140.56 140.04 134.27 132.26 132.23 130.81 129.10 128.57 128.31 128.06 126.46 125.42 122.84
77.48 77.16 76.84 70.95 68.82
57.97 53.52 46.76
36.54 34.21 31.78
210 200 190 180 170 160 150 140 130 120 110 100 90 80 70 60 50 40 30 20 10 0

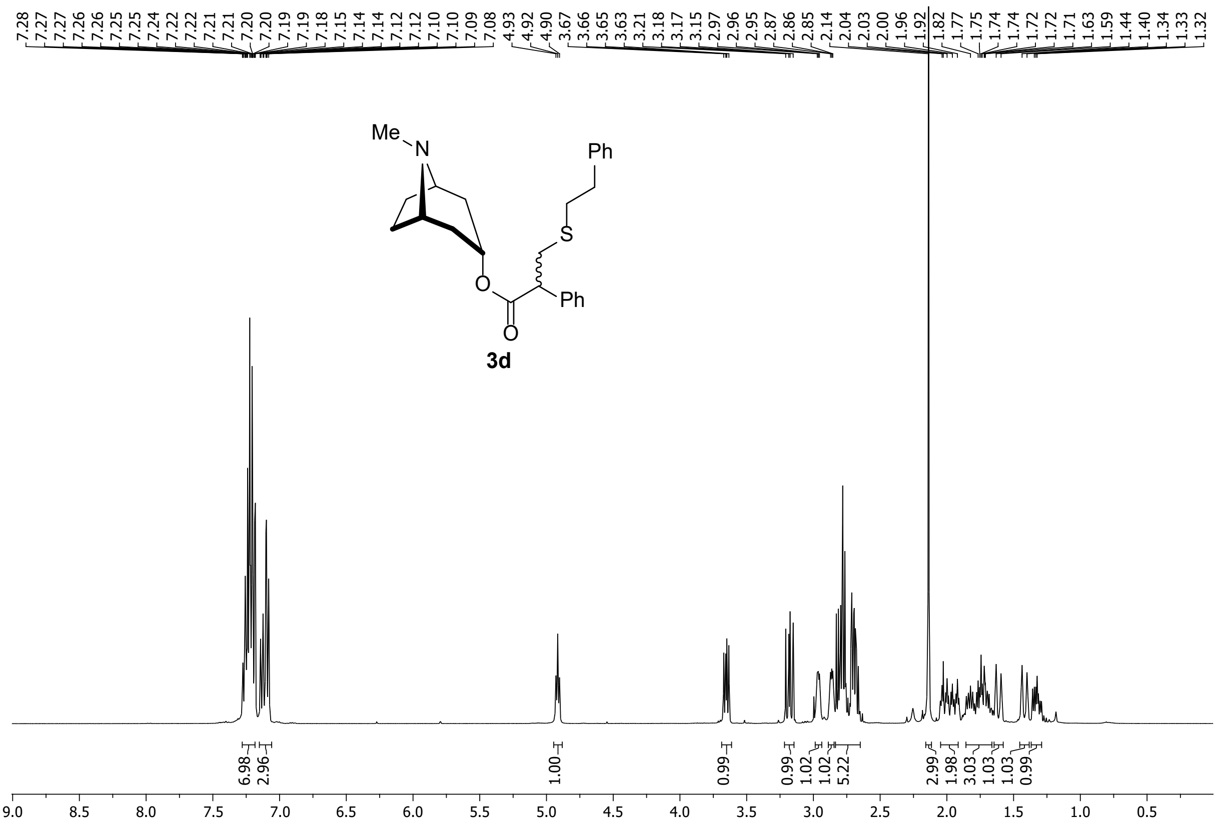
7.28 7.27 7.27 7.26 7.26 7.25 7.25 7.24 7.22 7.22 7.21 7.21 7.20 7.20 7.19 7.19 7.18 7.15 7.14 7.14 7.12 7.12 7.10 7.10 7.09 7.08 4.93 4.92 4.90 3.67 3.66 3.65 3.63 3.21 3.18 3.17 3.15 2.97 2.96 2.95 2.87 2.86 2.85 2.14 2.04 2.03 2.00 1.96 1.92 1.82 1.77 1.75 1.74 1.74 1.72 1.72 1.71 1.63 1.59 1.44 1.40 1.34 1.33 1.32
Me
N
Ph
S
O
O
Ph
3d
6.98 2.96 1.00 0.99 0.99 1.02 1.02 5.22 2.99 1.98 3.03 1.03 1.03 0.99
9.0 8.5 8.0 7.5 7.0 6.5 6.0 5.5 5.0 4.5 4.0 3.5 3.0 2.5 2.0 1.5 1.0 0.5

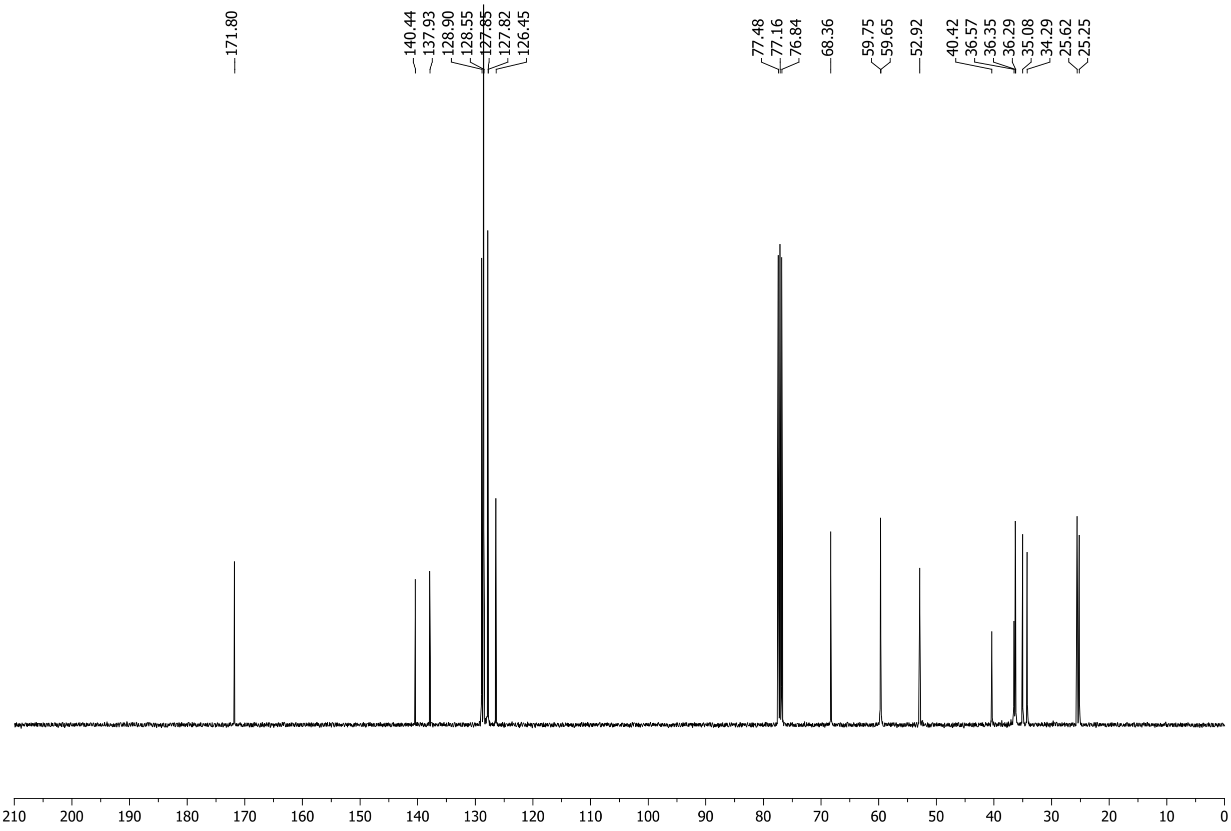
171.80
140.44 137.93 128.90 128.55 127.85 127.82 126.45
77.48 77.16 76.84
68.36
59.75 59.65
52.92
40.42 36.57 36.35 36.29 35.08 34.29 25.62 25.25
210 200 190 180 170 160 150 140 130 120 110 100 90 80 70 60 50 40 30 20 10 0

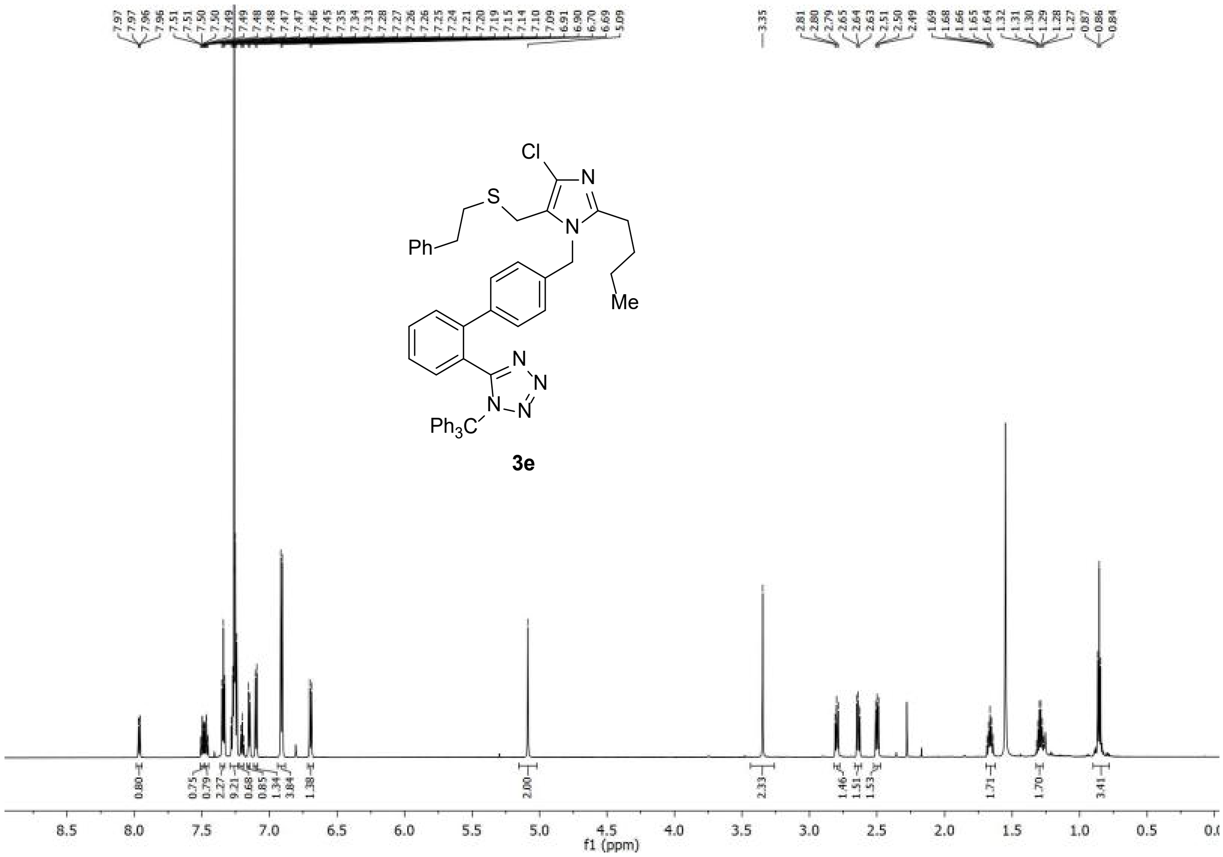
7.97 7.97 7.96 7.51 7.50 7.50 7.49 7.49 7.48 7.48 7.47 7.47 7.46 7.45 7.35 7.34 7.33 7.28 7.27 7.26 7.26 7.25 7.24 7.21 7.20 7.19 7.15 7.14 7.10 7.09 6.91 6.90 6.70 6.69 5.09
3.35
2.81 2.80 2.79 2.65 2.64 2.63 2.51 2.50 2.49 1.69 1.68 1.66 1.65 1.64 1.32 1.31 1.30 1.29 1.28 1.27 0.87 0.86 0.84
Cl
N
S
N
Ph
Me
N
N
N
N
Ph_3C
3e
0.80 0.75 0.79 2.27 9.21 0.68 0.85 1.34 3.84 1.38 2.00 2.33 1.46 1.51 1.53 1.71 1.70 3.41
8.5 8.0 7.5 7.0 6.5 6.0 5.5 5.0 4.5 4.0 3.5 3.0 2.5 2.0 1.5 1.0 0.5
f1 (ppm)

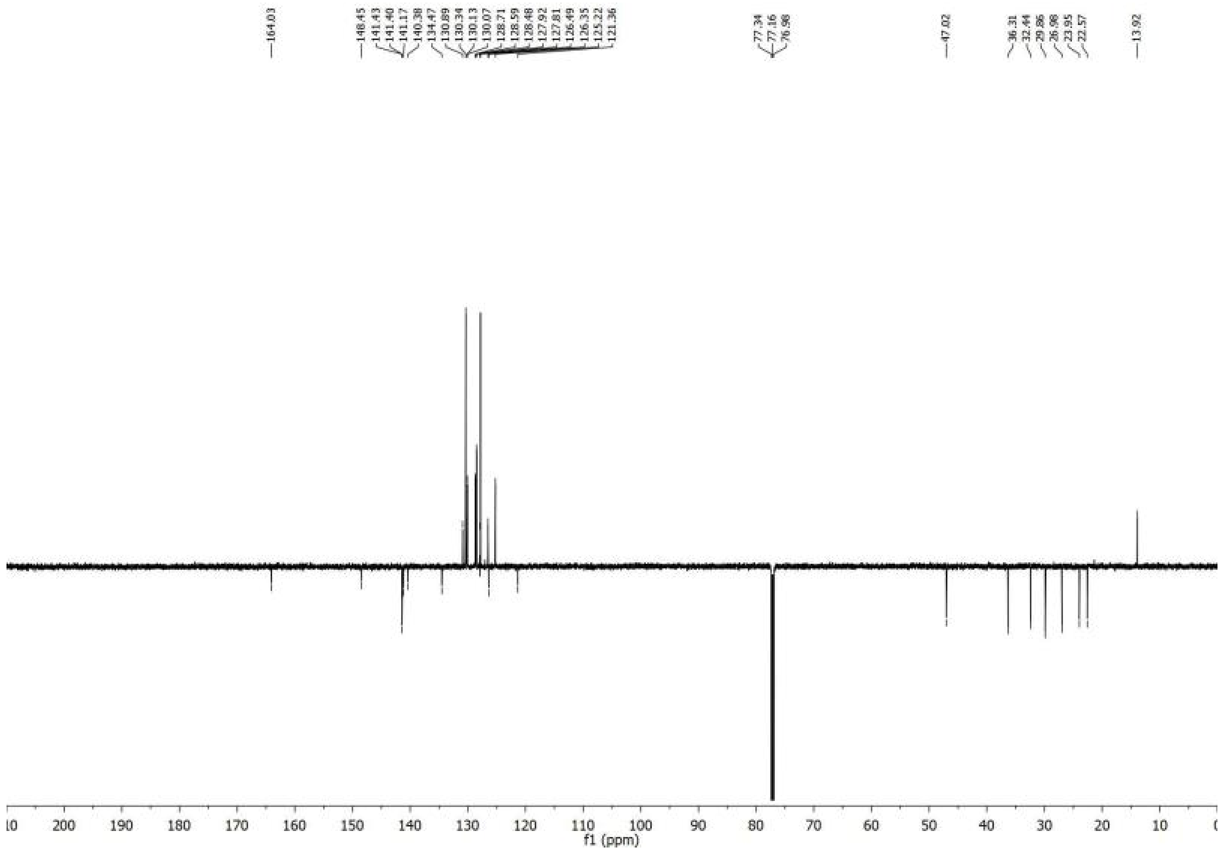
164.03
148.45 141.43 141.40 141.17 140.38 134.47 130.89 130.34 130.13 130.07 128.71 128.59 128.48 127.92 127.81 126.49 126.35 125.22 121.36
77.34 77.16 76.98
47.02
36.31 32.44 29.86 26.98 23.95 22.57
13.92
200 190 180 170 160 150 140 130 120 110 100 90 80 70 60 50 40 30 20 10
f1 (ppm)

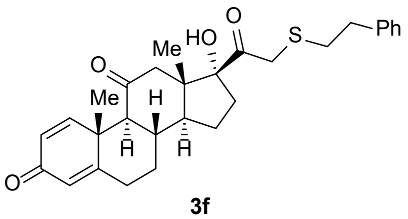
O
Me
HO
S
Ph
O
Me
H
H
H
O

3f

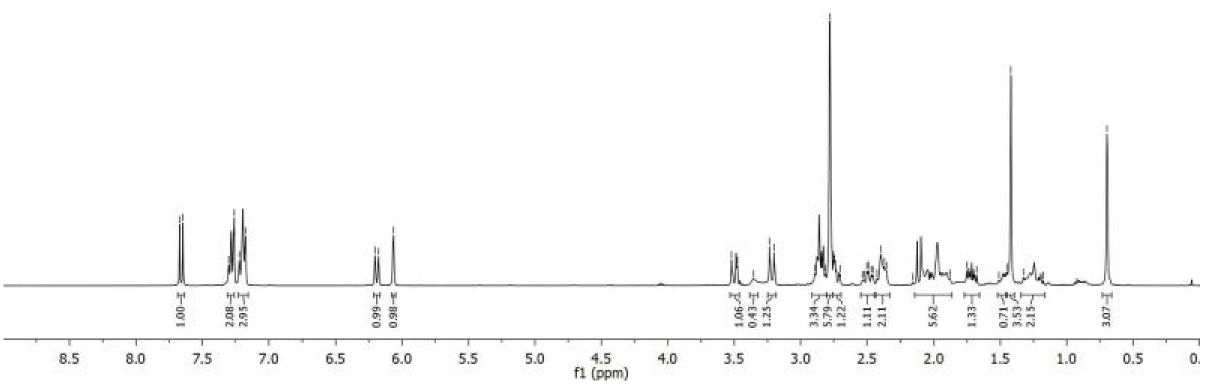
7.67
7.65
7.30
7.26
7.26
7.22
7.18
6.20
6.18
6.07
3.52
3.48
3.35
3.24
3.20
2.89
2.81
2.78
2.75
2.70
2.53
2.52
2.50
2.49
2.47
2.46
2.43
2.40
2.37
2.35
2.16
1.88
1.75
1.68
1.51
1.45
1.42
1.33
1.18
0.70
1.00
2.08
2.95
0.99
0.98
1.06
0.43
1.25
3.34
5.79
1.22
1.11
2.11
5.62
1.33
0.71
3.53
2.15
3.07
f1 (ppm)

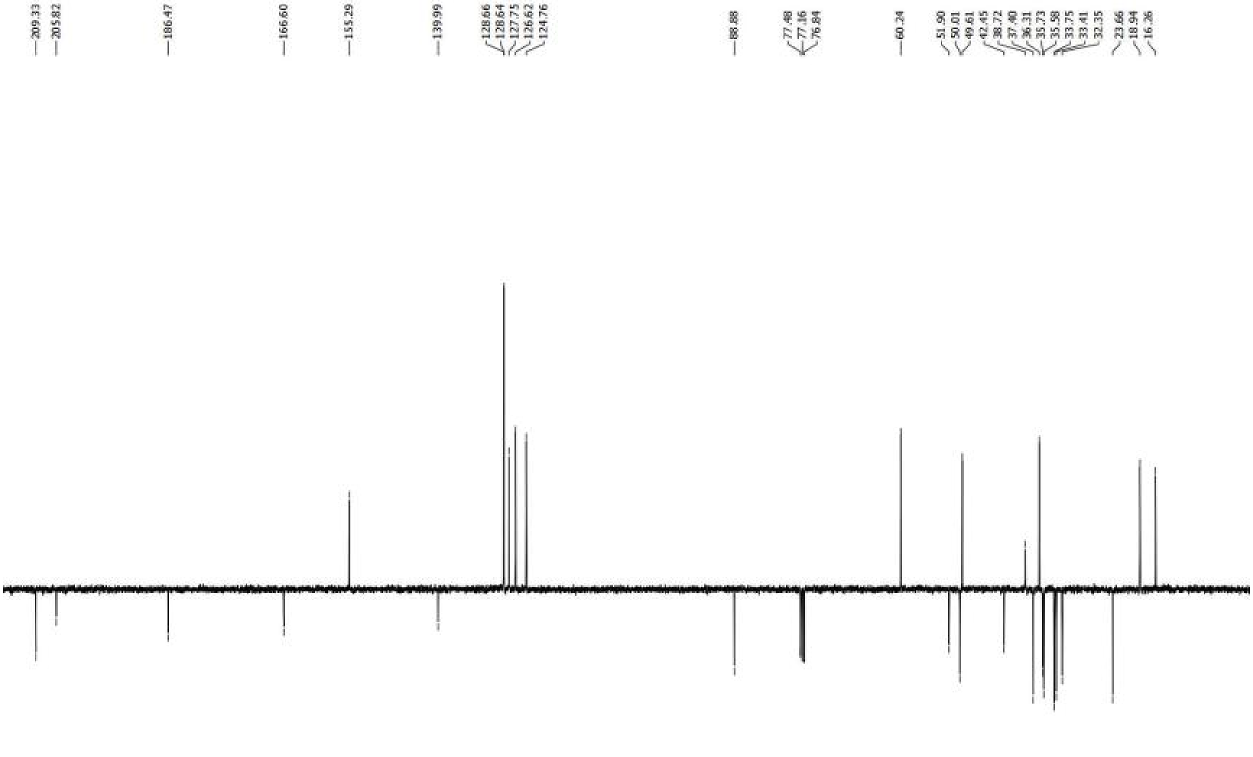
209.33
205.82
186.47
166.60
155.29
139.99
128.66
128.64
127.75
126.62
124.76
88.88
77.48
77.16
76.84
60.24
51.90
50.01
49.61
42.45
38.72
37.40
36.31
35.73
35.58
33.75
33.41
32.35
23.66
18.94
16.26
f1 (ppm)

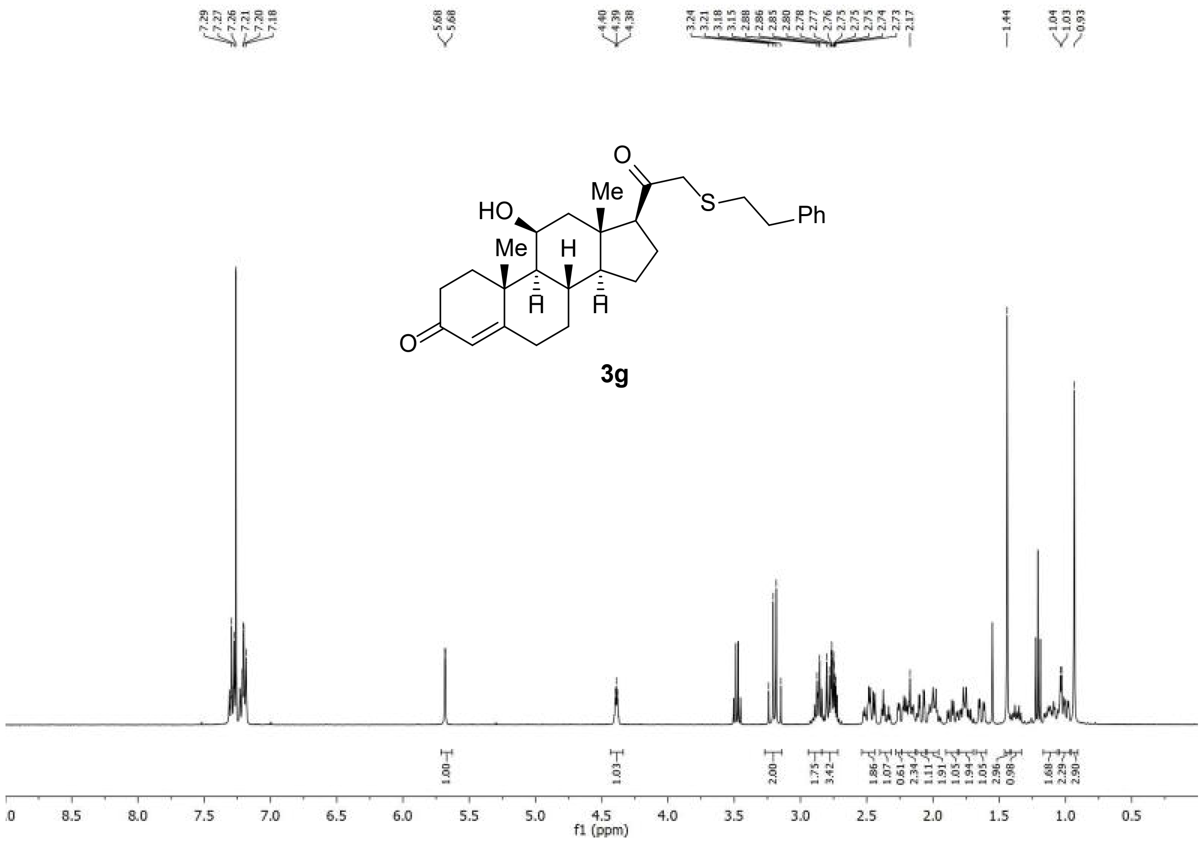
7.29 7.27 7.26 7.21 7.20 7.18
5.68 5.68
4.40 4.39 4.38
3.24 3.21 3.18 3.15 2.88 2.86 2.85 2.80 2.78 2.77 2.76 2.75 2.75 2.74 2.73 2.17
1.44
1.04 1.03 0.93
f1 (ppm)
O
Me
HO
S
Ph
Me
H
O
H
H

3g

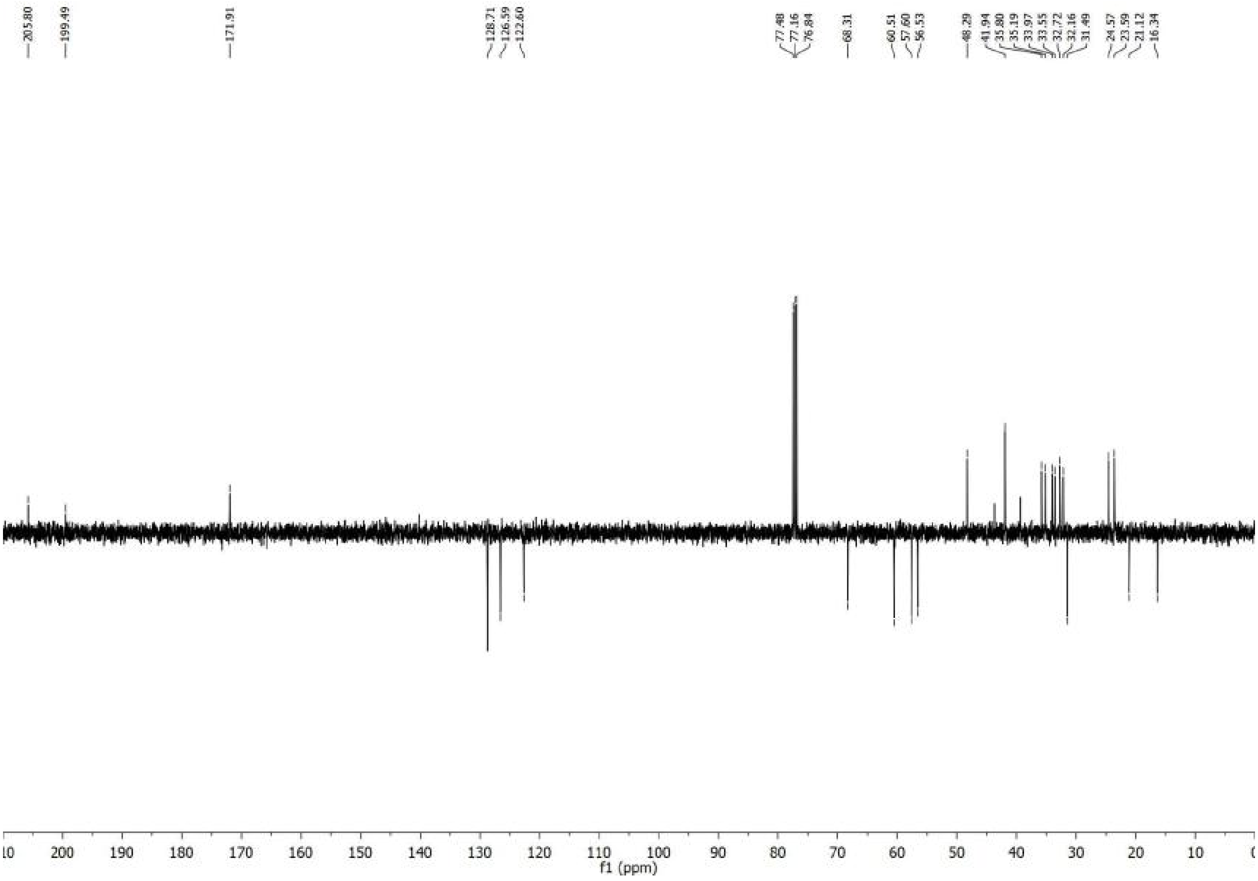
205.80
199.49
171.91
128.71 126.59 122.60
77.48 77.16 76.84
68.31
60.51 57.60 56.53
48.29 41.94 35.80 35.19 33.97 33.55 32.72 32.16 31.49
24.57 23.59 21.12 16.34
f1 (ppm)

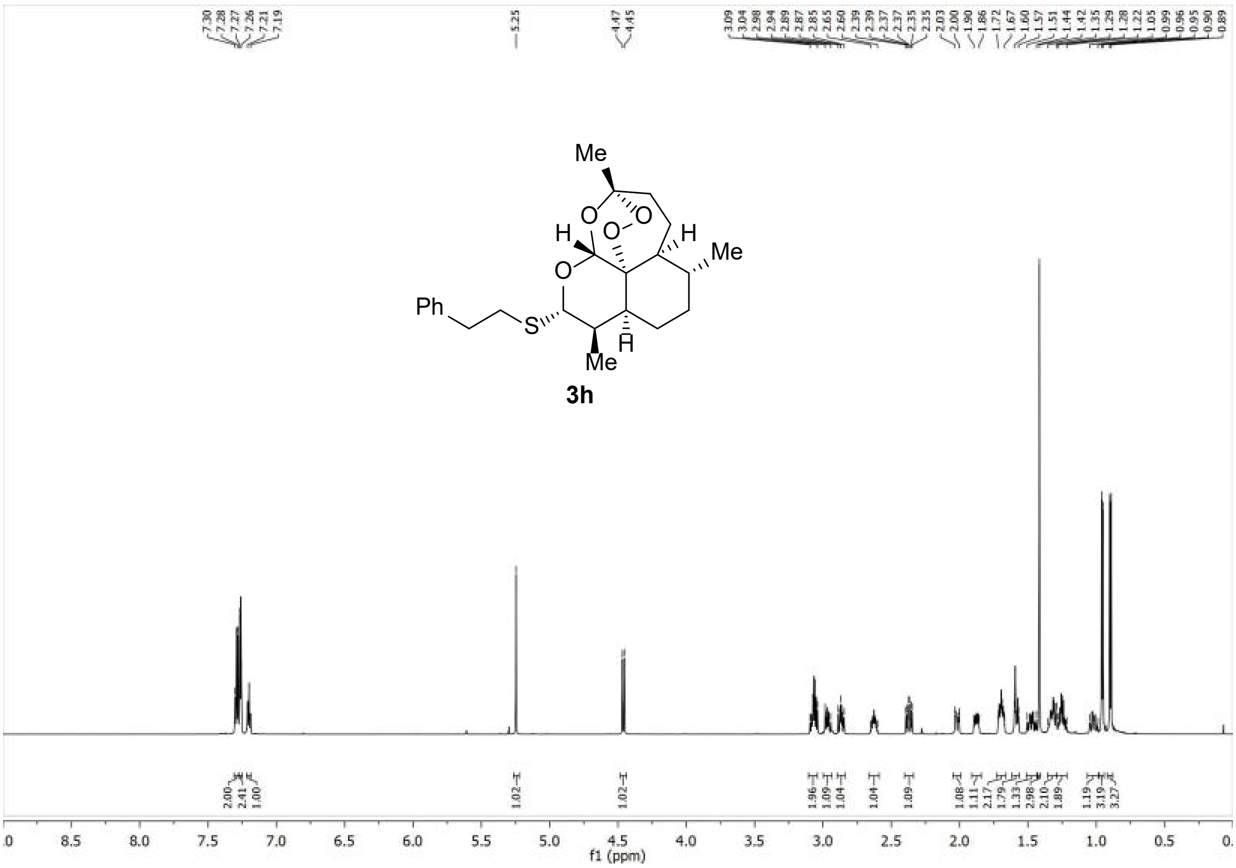
Me
Me
Ph
S
Me
3h
f1 (ppm)

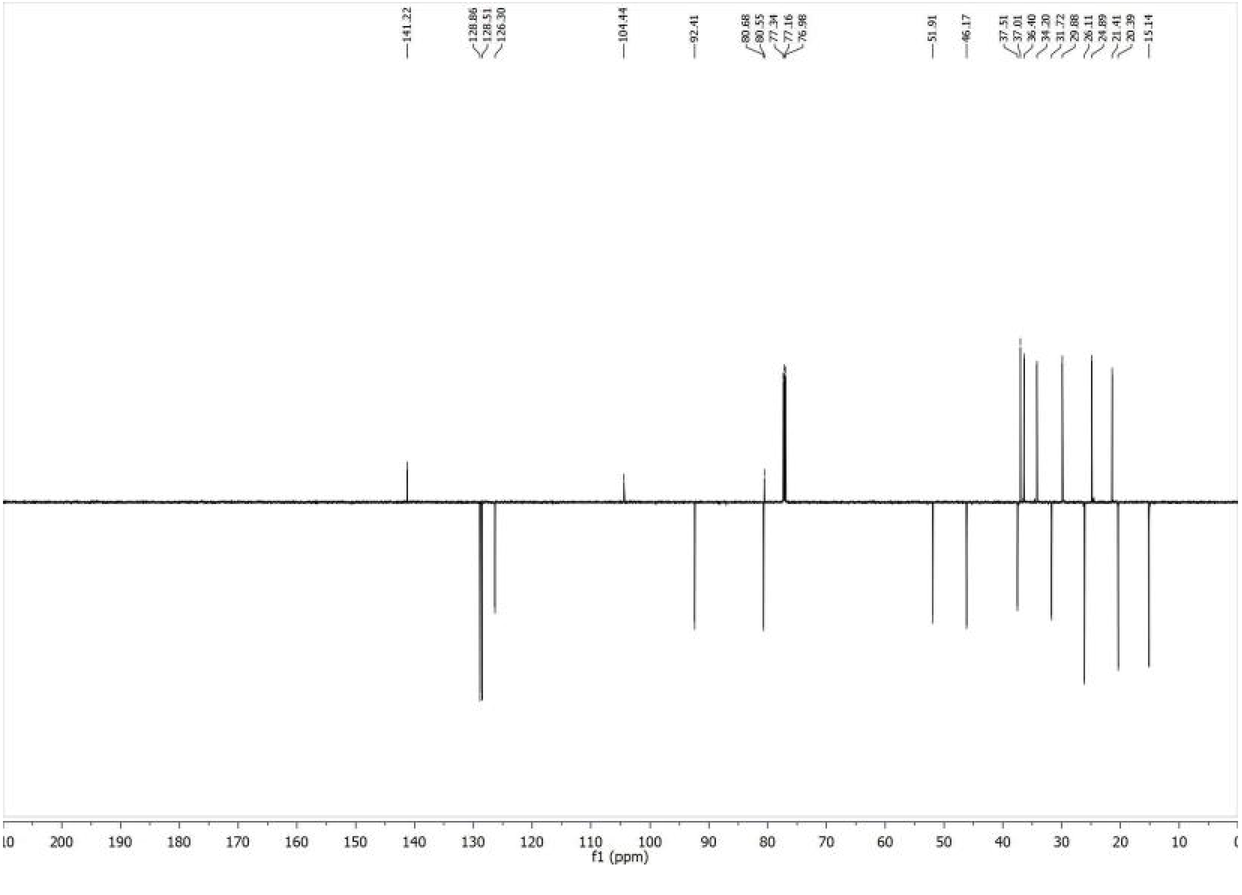
f1 (ppm)

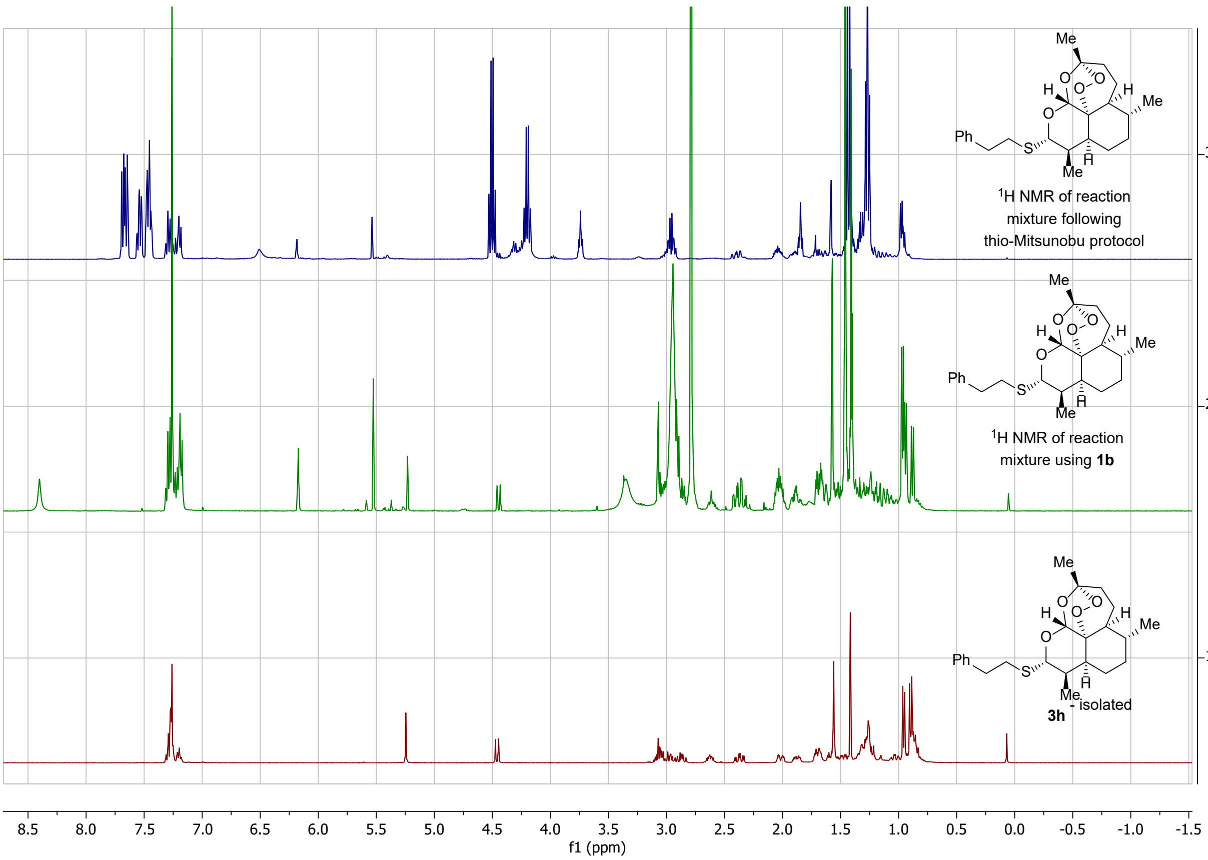
Me
H
O
O
O
O
H
Me
Ph
S
Me
H
^{1}H NMR of reaction mixture following thio-Mitsunobu protocol
^{1}H NMR of reaction mixture using **1b**
3h - isolated
3
2
1
8.5 8.0 7.5 7.0 6.5 6.0 5.5 5.0 4.5 4.0 3.5 3.0 2.5 2.0 1.5 1.0 0.5 0.0 -0.5 -1.0 -1.5
f1 (ppm)

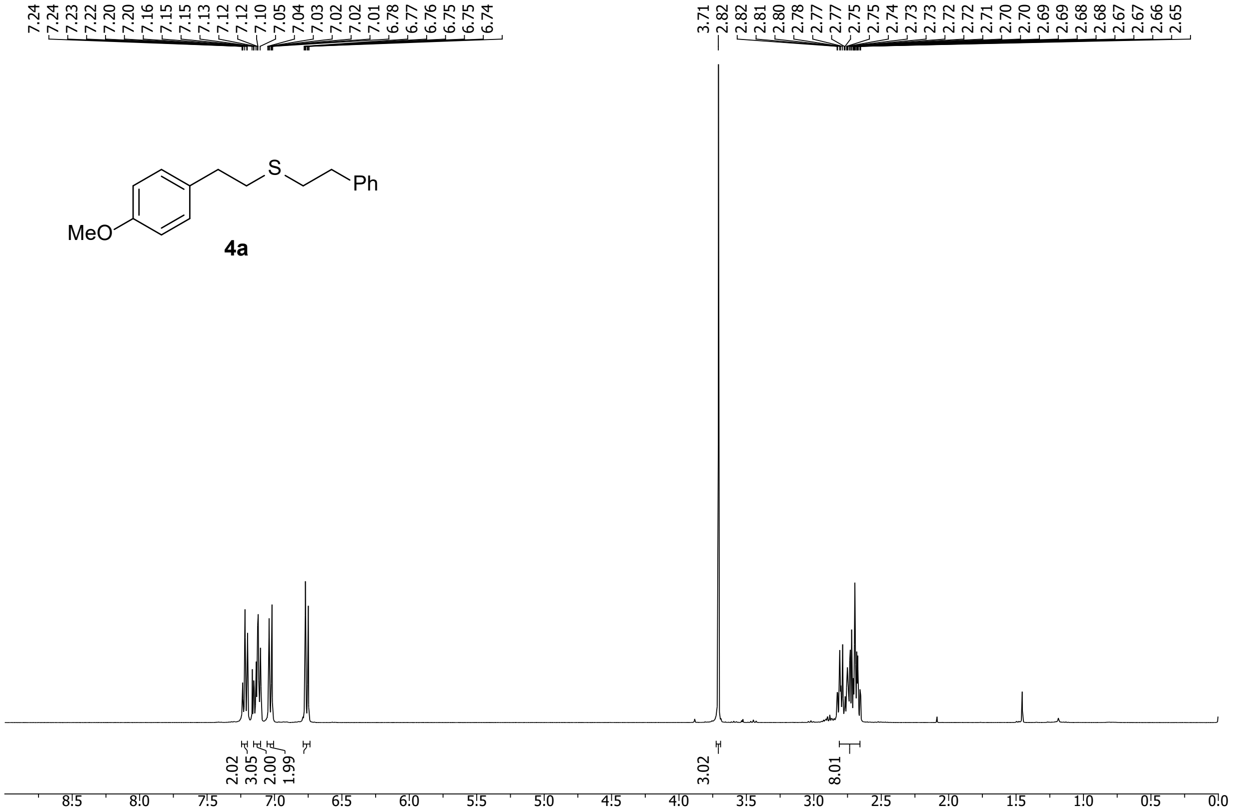
S
Ph
MeO
4a
7.24 7.24 7.23 7.22 7.20 7.20 7.16 7.15 7.15 7.13 7.12 7.12 7.10 7.05 7.04 7.03 7.02 7.02 7.01 6.78 6.77 6.76 6.75 6.74
3.71 2.82 2.82 2.81 2.80 2.78 2.77 2.77 2.75 2.75 2.74 2.73 2.73 2.72 2.72 2.71 2.70 2.70 2.69 2.68 2.68 2.67 2.67 2.66 2.65
2.02 3.05 2.00 1.99
3.02
8.01
8.5 8.0 7.5 7.0 6.5 6.0 5.5 5.0 4.5 4.0 3.5 3.0 2.5 2.0 1.5 1.0 0.5 0.0

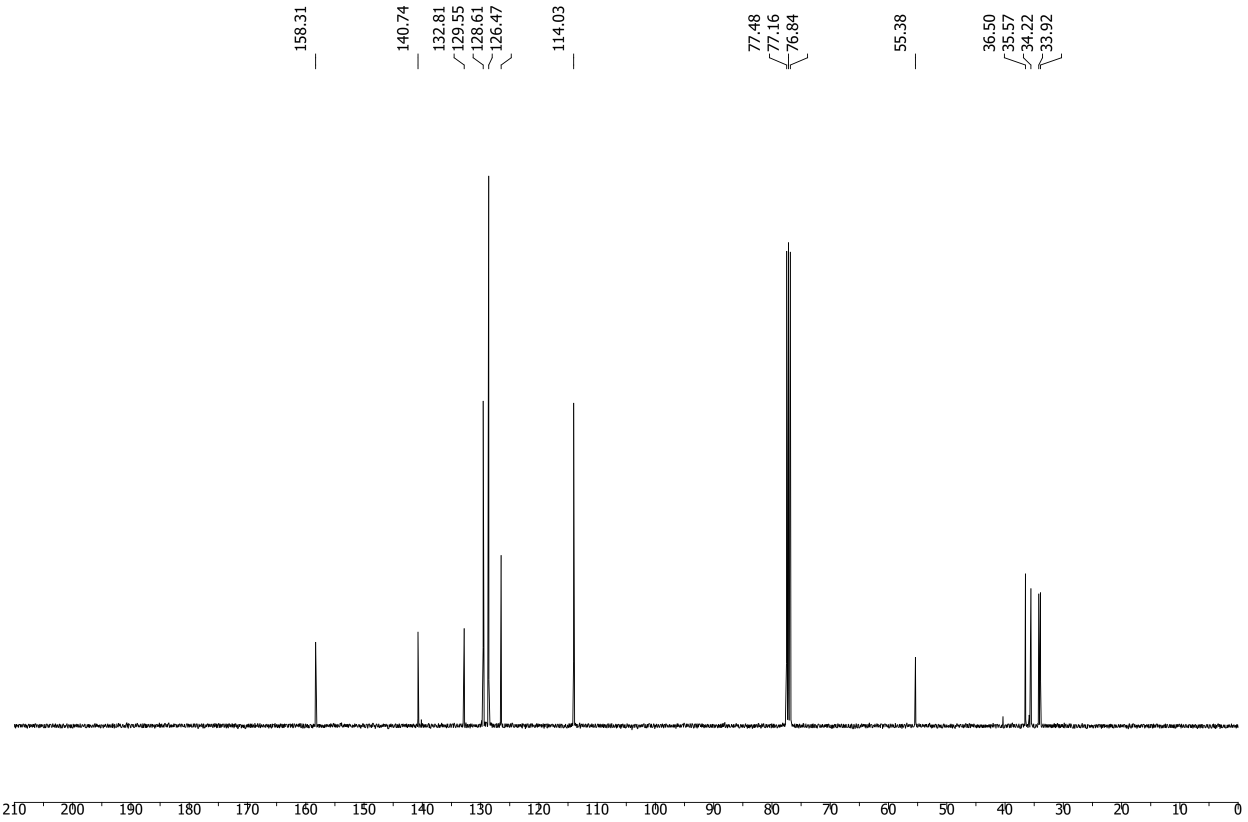
158.31
140.74
132.81 129.55 128.61 126.47
114.03
77.48 77.16 76.84
55.38
36.50 35.57 34.22 33.92
210 200 190 180 170 160 150 140 130 120 110 100 90 80 70 60 50 40 30 20 10 0

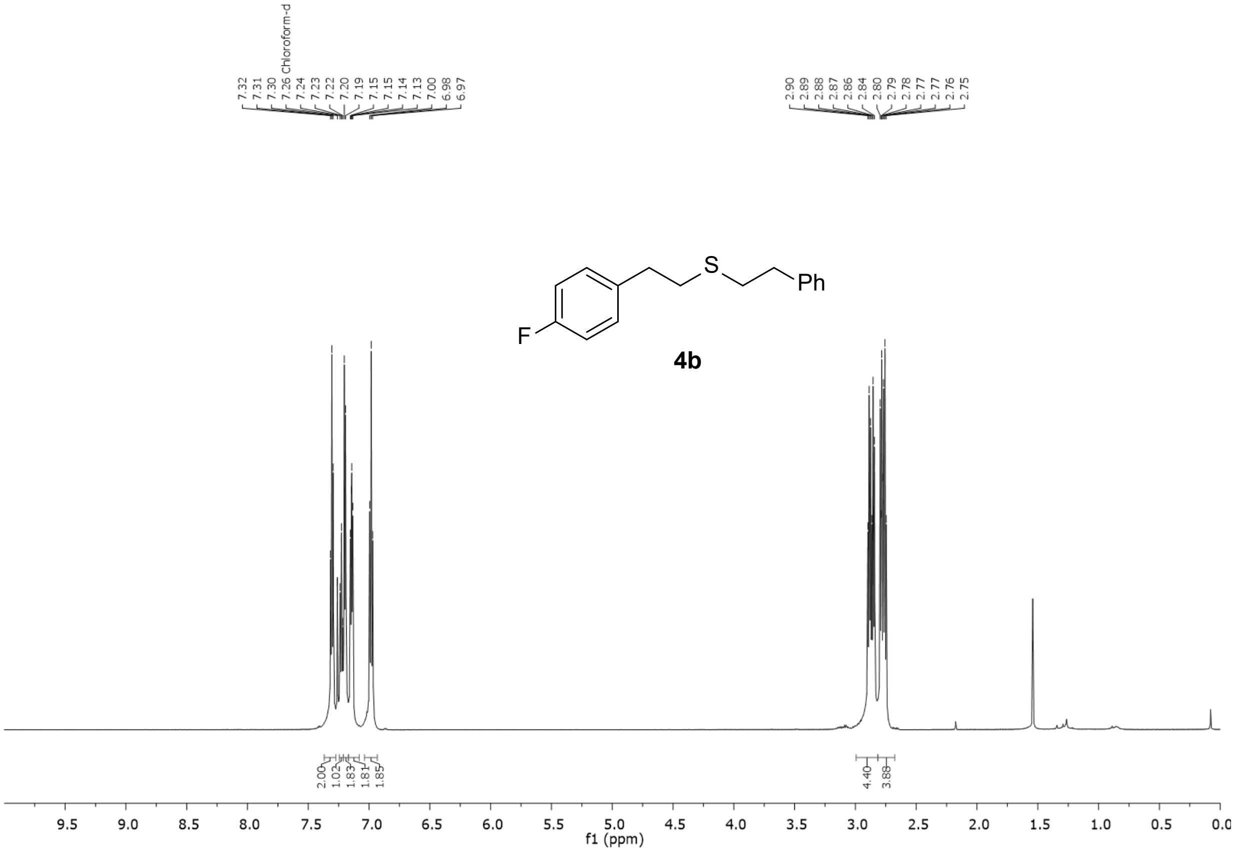
7.32
7.31
7.30
7.26 Chloroform-d
7.24
7.23
7.22
7.20
7.19
7.15
7.15
7.14
7.13
7.00
6.98
6.97
2.90
2.89
2.88
2.87
2.86
2.84
2.80
2.79
2.78
2.77
2.77
2.76
2.75
2.00
1.02
1.83
1.81
1.85
4.40
3.88
F
S
Ph
f1 (ppm)

4b

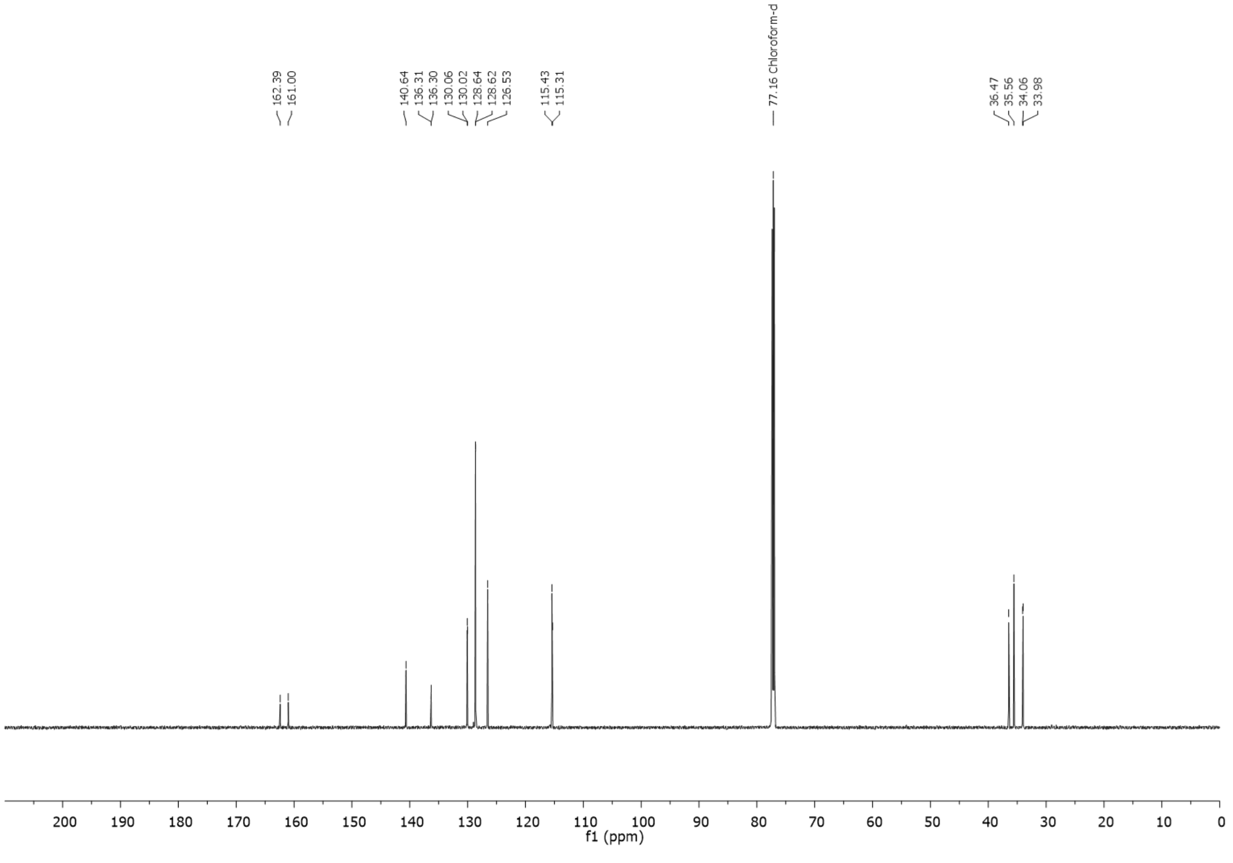
162.39
161.00
140.64
136.31
136.30
130.06
130.02
128.64
128.62
126.53
115.43
115.31
77.16 Chloroform-d
36.47
35.56
34.06
33.98
f1 (ppm)

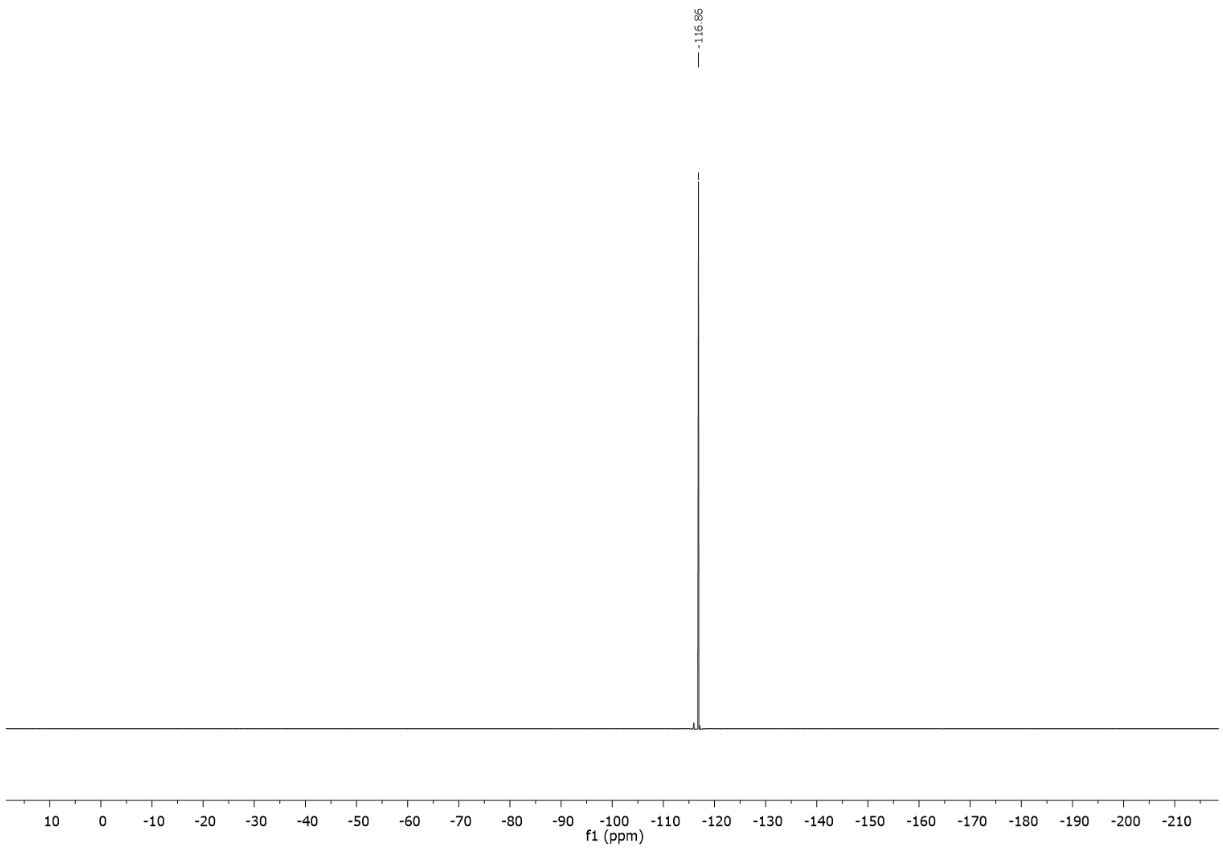
-116.86
10 0 -10 -20 -30 -40 -50 -60 -70 -80 -90 -100 -110 -120 -130 -140 -150 -160 -170 -180 -190 -200 -210
f1 (ppm)

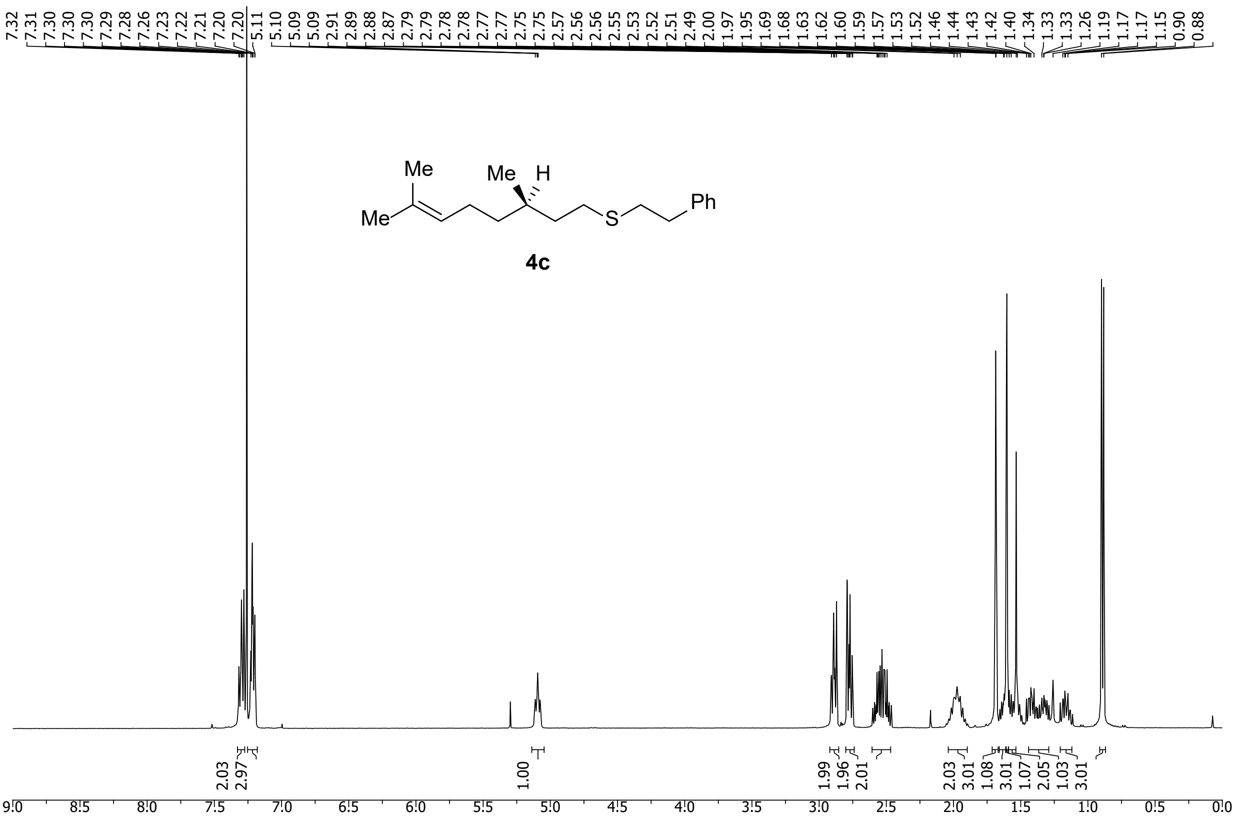
7.32 7.31 7.30 7.30 7.30 7.29 7.28 7.26 7.23 7.22 7.21 7.20 7.20 5.11 5.10 5.09 5.09 2.91 2.89 2.88 2.87 2.79 2.79 2.78 2.78 2.77 2.77 2.75 2.75 2.57 2.56 2.56 2.55 2.53 2.52 2.51 2.49 2.00 1.97 1.95 1.69 1.68 1.63 1.62 1.60 1.59 1.57 1.53 1.52 1.46 1.44 1.43 1.42 1.40 1.34 1.33 1.33 1.26 1.19 1.17 1.17 1.15 0.90 0.88
Me
Me
Me
H
S
Ph
4c
2.03 2.97 1.00 1.99 1.96 2.01 2.03 3.01 1.08 3.01 1.07 2.05 1.03 3.01
9.0 8.5 8.0 7.5 7.0 6.5 6.0 5.5 5.0 4.5 4.0 3.5 3.0 2.5 2.0 1.5 1.0 0.5 0.0

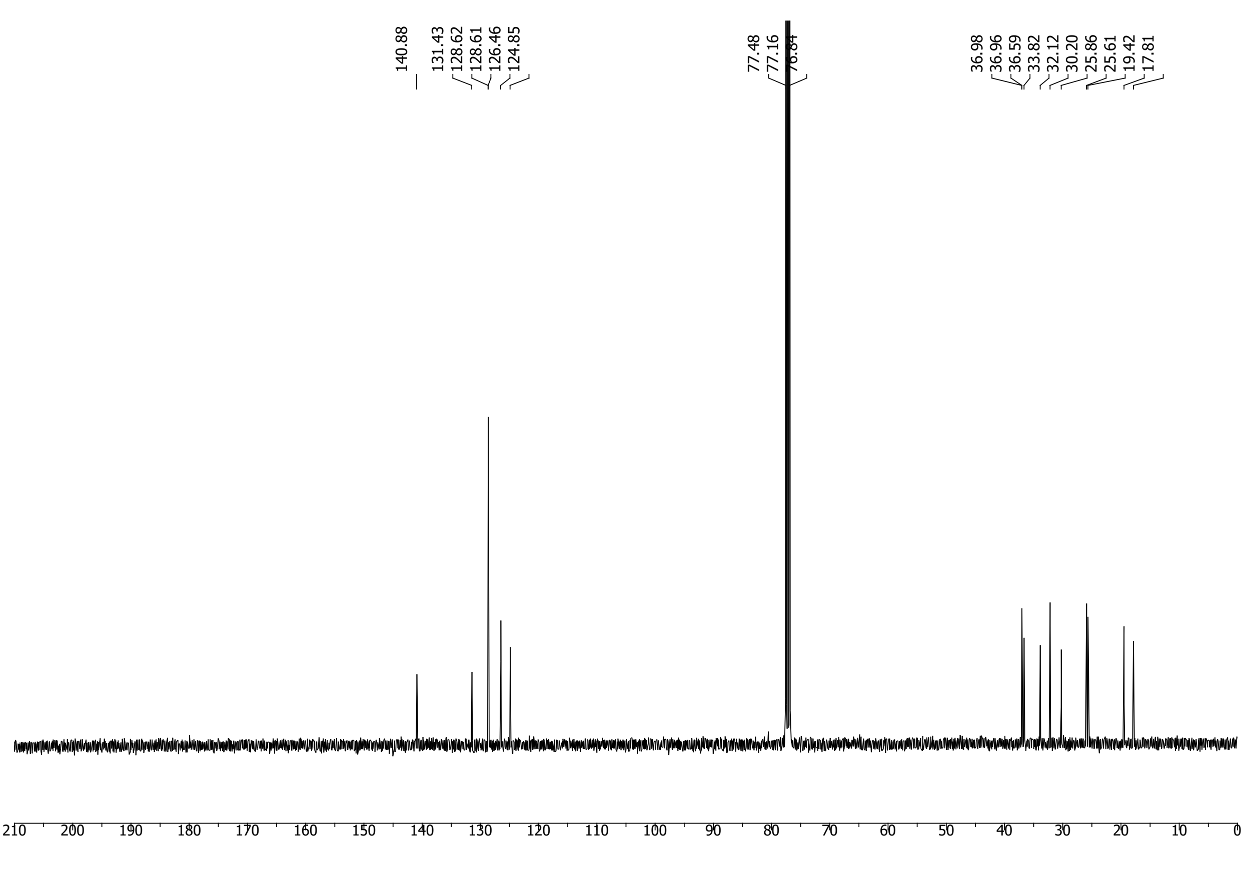
140.88 131.43 128.62 128.61 126.46 124.85 77.48 77.16 76.84 36.98 36.96 36.59 33.82 32.12 30.20 25.86 25.61 19.42 17.81
210 200 190 180 170 160 150 140 130 120 110 100 90 80 70 60 50 40 30 20 10 0

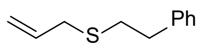
S
Ph

4d

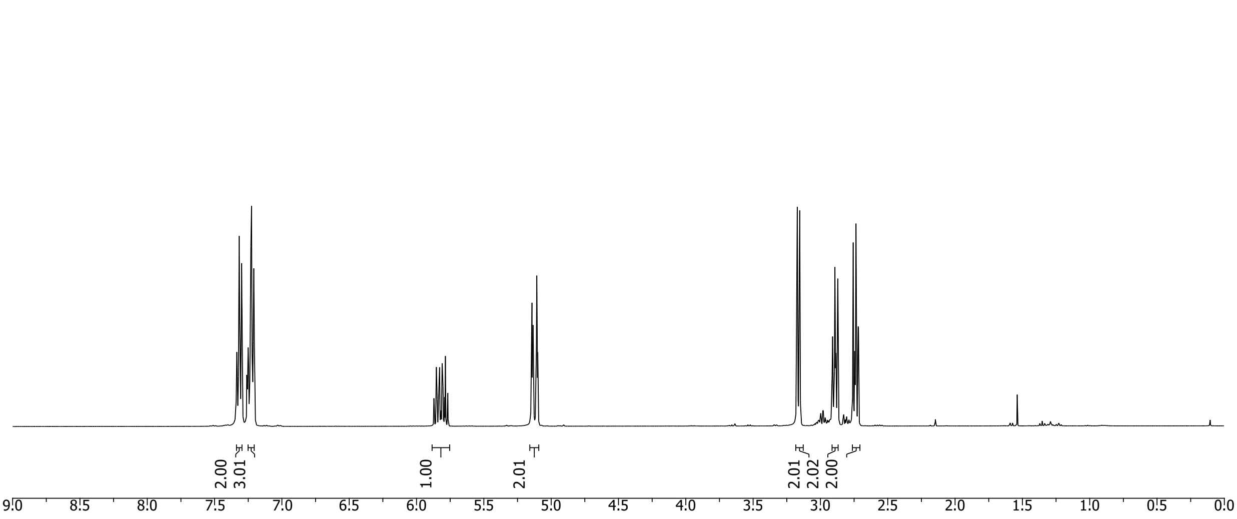
7.34 7.33 7.33 7.32 7.32 7.31 7.30 7.26 7.25 7.25 7.25 7.23 7.23 7.22 7.22 7.21 7.21 7.21 5.87 5.85 5.85 5.83 5.83 5.81 5.80 5.79 5.78 5.77 5.15 5.14 5.14 5.14 5.14 5.13 5.13 5.13 5.13 5.11 5.11 5.11 5.11 5.10 5.10 5.10 3.17 3.17 3.17 3.16 3.15 3.15 2.91 2.89 2.88 2.87 2.76 2.74 2.74 2.74 2.73 2.73 2.72 2.72
2.00
3.01
1.00
2.01
2.01
2.02
2.00
9.0 8.5 8.0 7.5 7.0 6.5 6.0 5.5 5.0 4.5 4.0 3.5 3.0 2.5 2.0 1.5 1.0 0.5 0.0

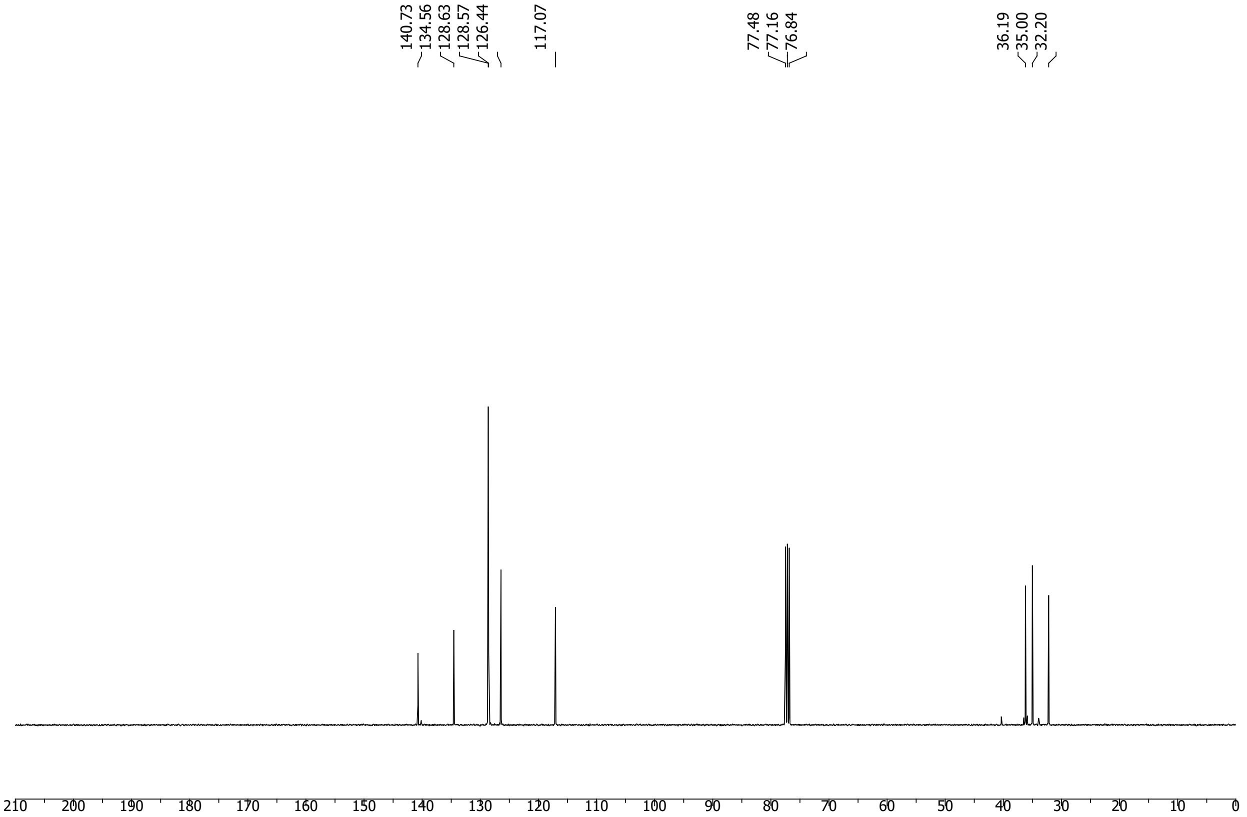
140.73
134.56
128.63
128.57
126.44
117.07
77.48
77.16
76.84
36.19
35.00
32.20
210 200 190 180 170 160 150 140 130 120 110 100 90 80 70 60 50 40 30 20 10 0

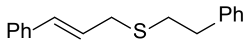

Ph
S
Ph

4e

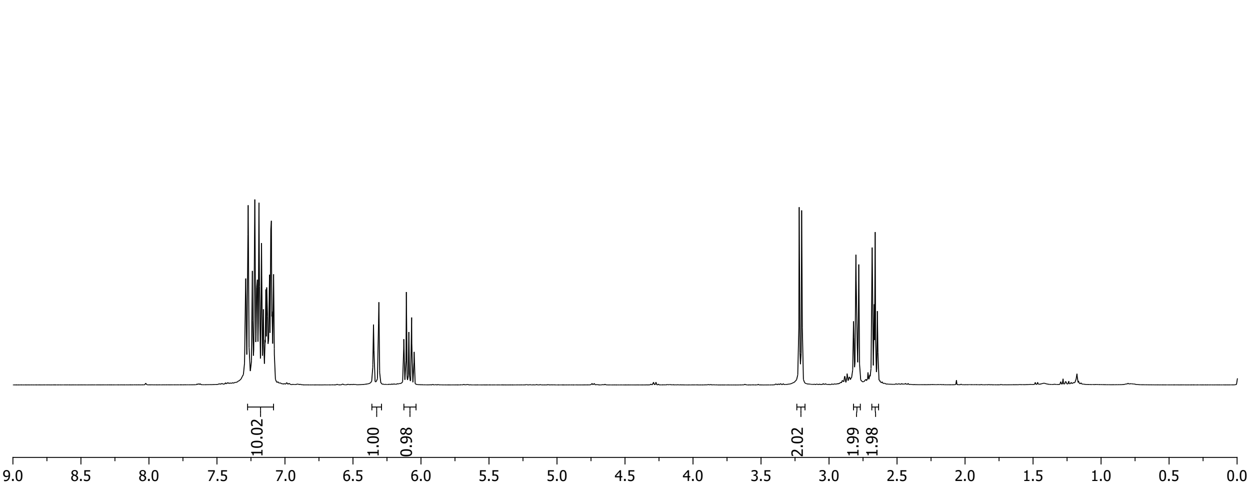

7.29
7.29
7.27
7.24
7.22
7.22
7.21
7.20
7.19
7.17
7.14
7.13
7.11
7.10
7.10
7.08
6.35
6.31
6.13
6.11
6.09
6.09
6.07
6.05
3.22
3.22
3.20
3.20
2.82
2.80
2.80
2.78
2.68
2.68
2.67
2.67
2.66
2.65
2.64
10.02
1.00
0.98
2.02
1.99
1.98
9.0 8.5 8.0 7.5 7.0 6.5 6.0 5.5 5.0 4.5 4.0 3.5 3.0 2.5 2.0 1.5 1.0 0.5 0.0

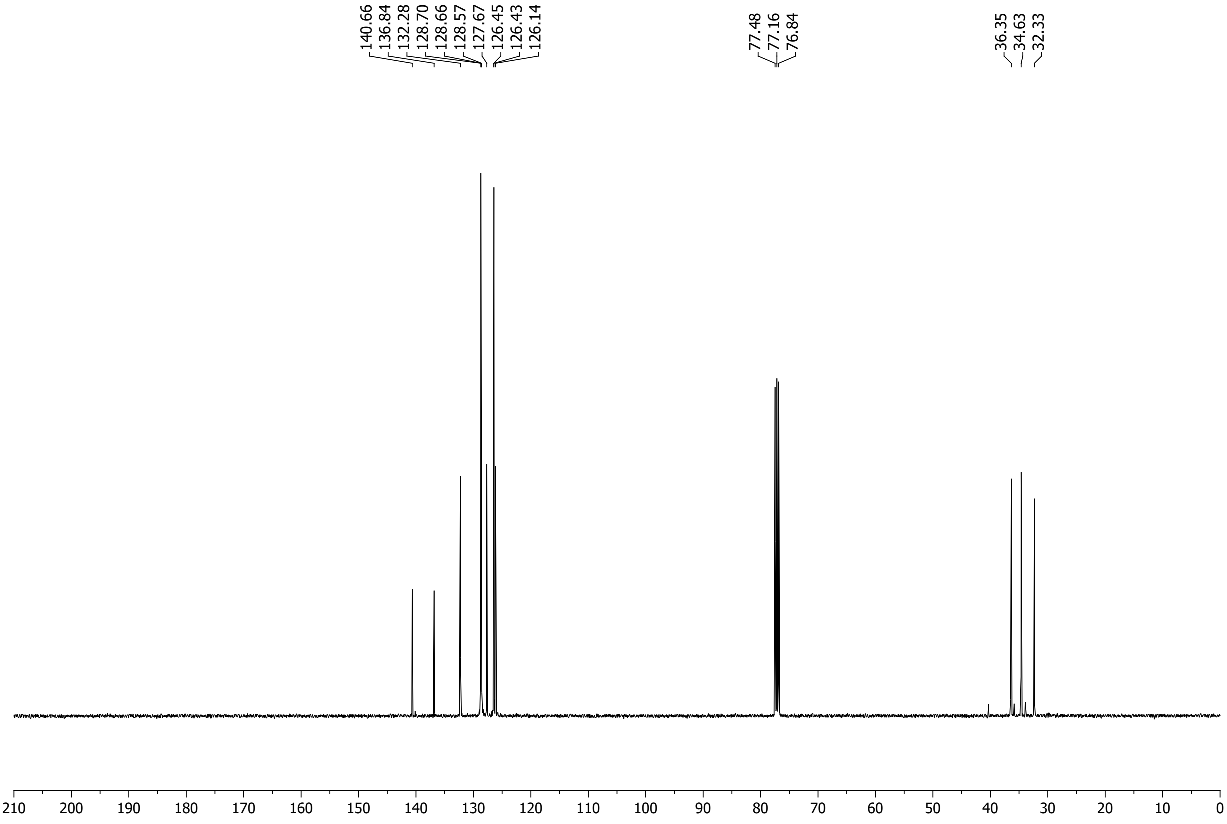

140.66
136.84
132.28
128.70
128.66
128.57
127.67
126.45
126.43
126.14
77.48
77.16
76.84
36.35
34.63
32.33
210 200 190 180 170 160 150 140 130 120 110 100 90 80 70 60 50 40 30 20 10 0

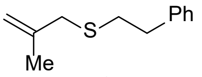
S
Ph
Me

4f

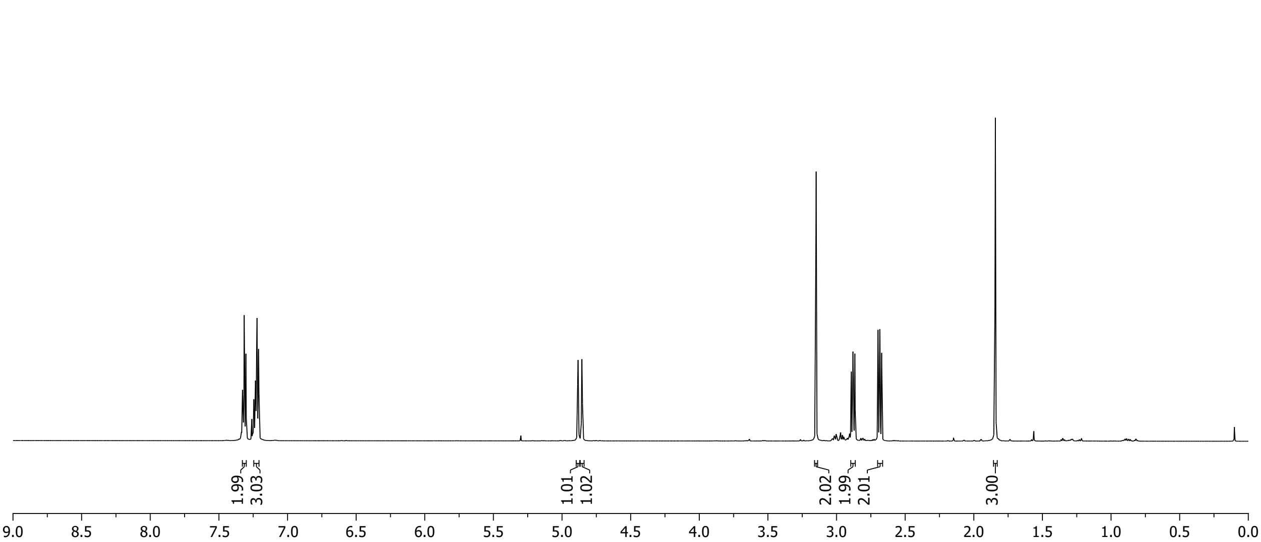
7.34
7.33
7.33
7.32
7.30
7.26
7.25
7.25
7.25
7.24
7.23
7.23
7.22
7.22
7.21
4.89
4.88
4.88
4.86
4.86
4.85
3.15
3.15
2.89
2.88
2.88
2.87
2.70
2.69
2.68
2.68
2.67
1.84
1.84
1.99
3.03
1.01
1.02
2.02
1.99
2.01
3.00
9.0
8.5
8.0
7.5
7.0
6.5
6.0
5.5
5.0
4.5
4.0
3.5
3.0
2.5
2.0
1.5
1.0
0.5
0.0

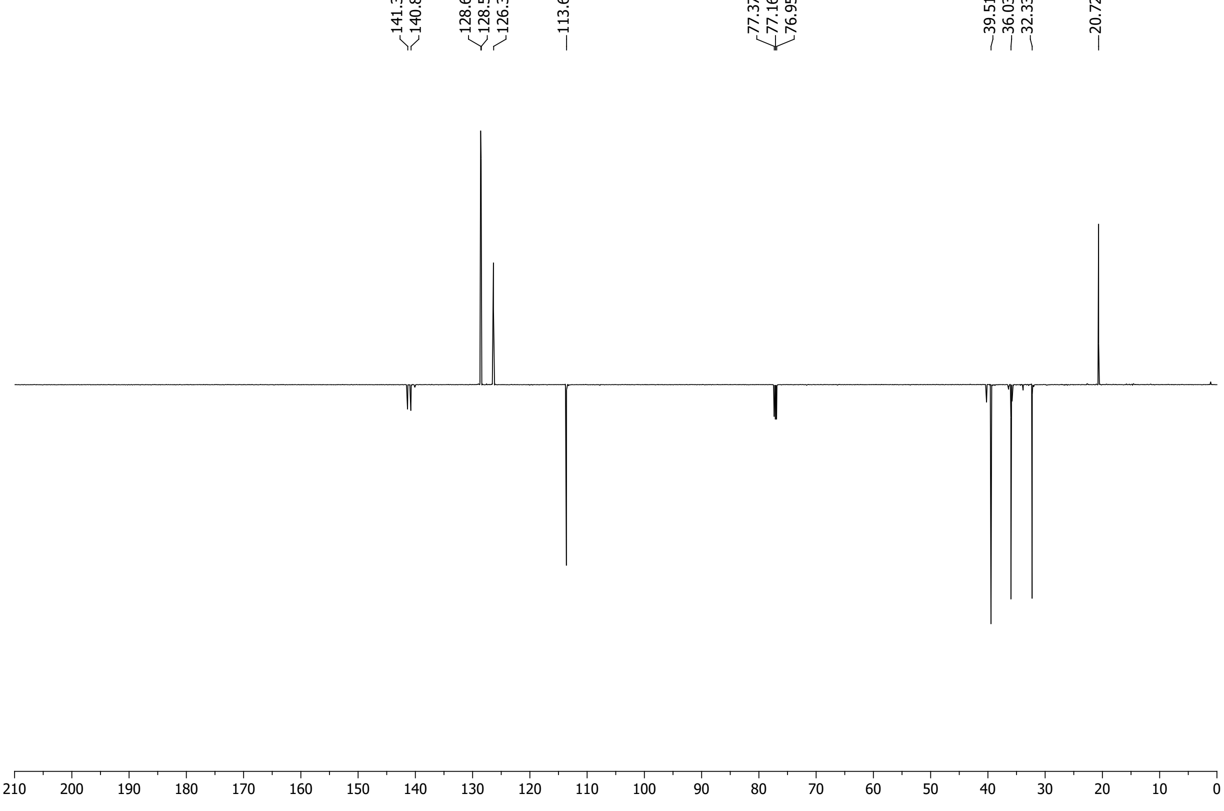
141.39
140.81
128.62
128.54
126.39
113.65
77.37
77.16
76.95
39.51
36.03
32.33
20.72
210
200
190
180
170
160
150
140
130
120
110
100
90
80
70
60
50
40
30
20
10
0

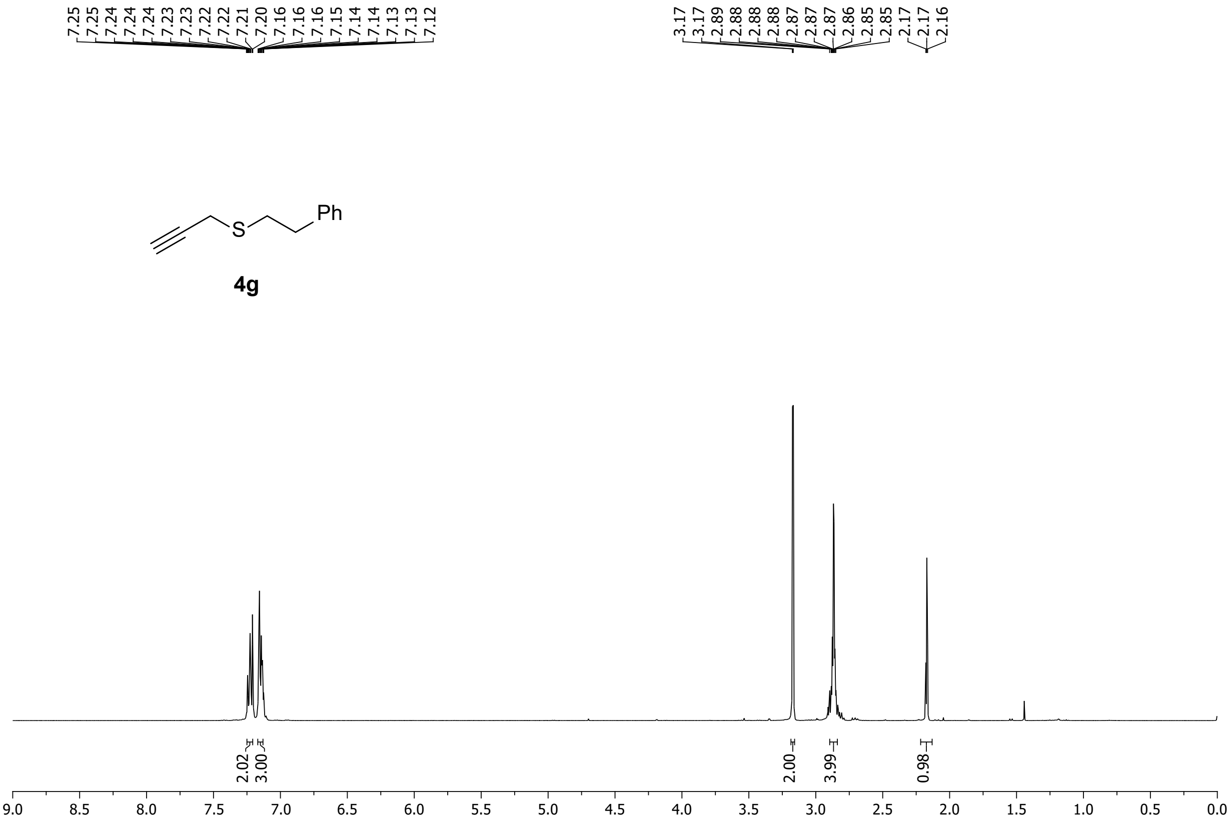
Ph
S
4g
7.25 7.25 7.24 7.24 7.24 7.23 7.23 7.22 7.22 7.21 7.20 7.16 7.16 7.16 7.15 7.14 7.14 7.13 7.13 7.12
3.17 3.17 2.89 2.88 2.88 2.88 2.87 2.87 2.87 2.86 2.85 2.85 2.17 2.17 2.16
2.02 3.00 2.00 3.99 0.98
9.0 8.5 8.0 7.5 7.0 6.5 6.0 5.5 5.0 4.5 4.0 3.5 3.0 2.5 2.0 1.5 1.0 0.5 0.0

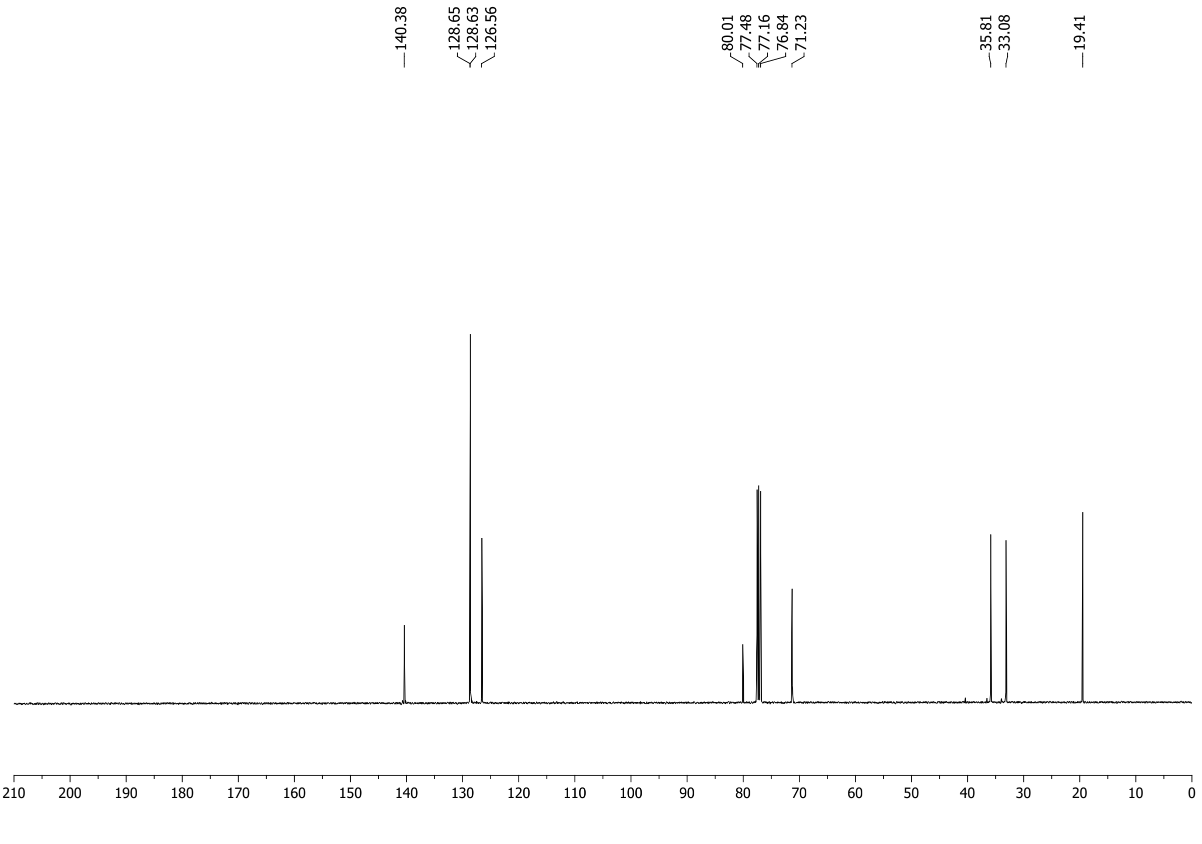
140.38
128.65 128.63 126.56
80.01 77.48 77.16 76.84 71.23
35.81 33.08
19.41
210 200 190 180 170 160 150 140 130 120 110 100 90 80 70 60 50 40 30 20 10 0

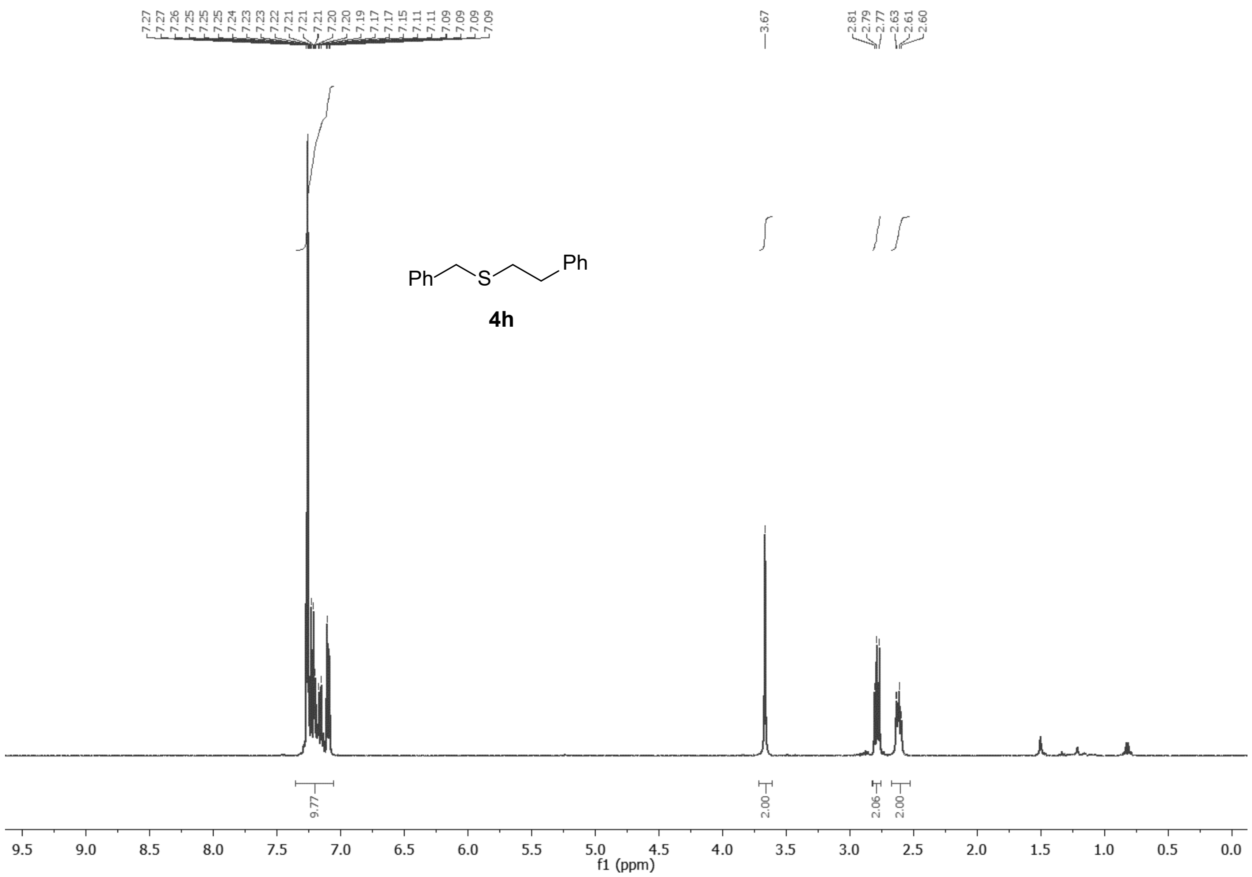
Ph
S
Ph
4h
f1 (ppm)

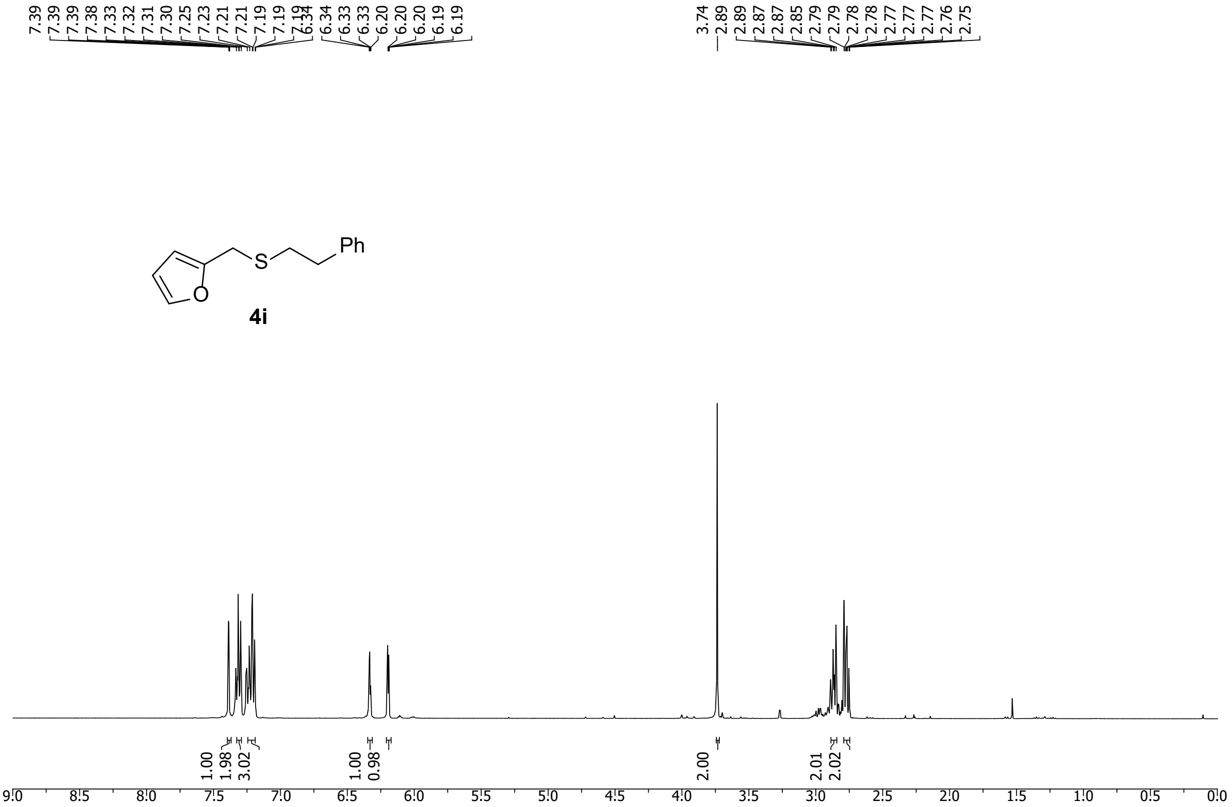
S
Ph
O
4i

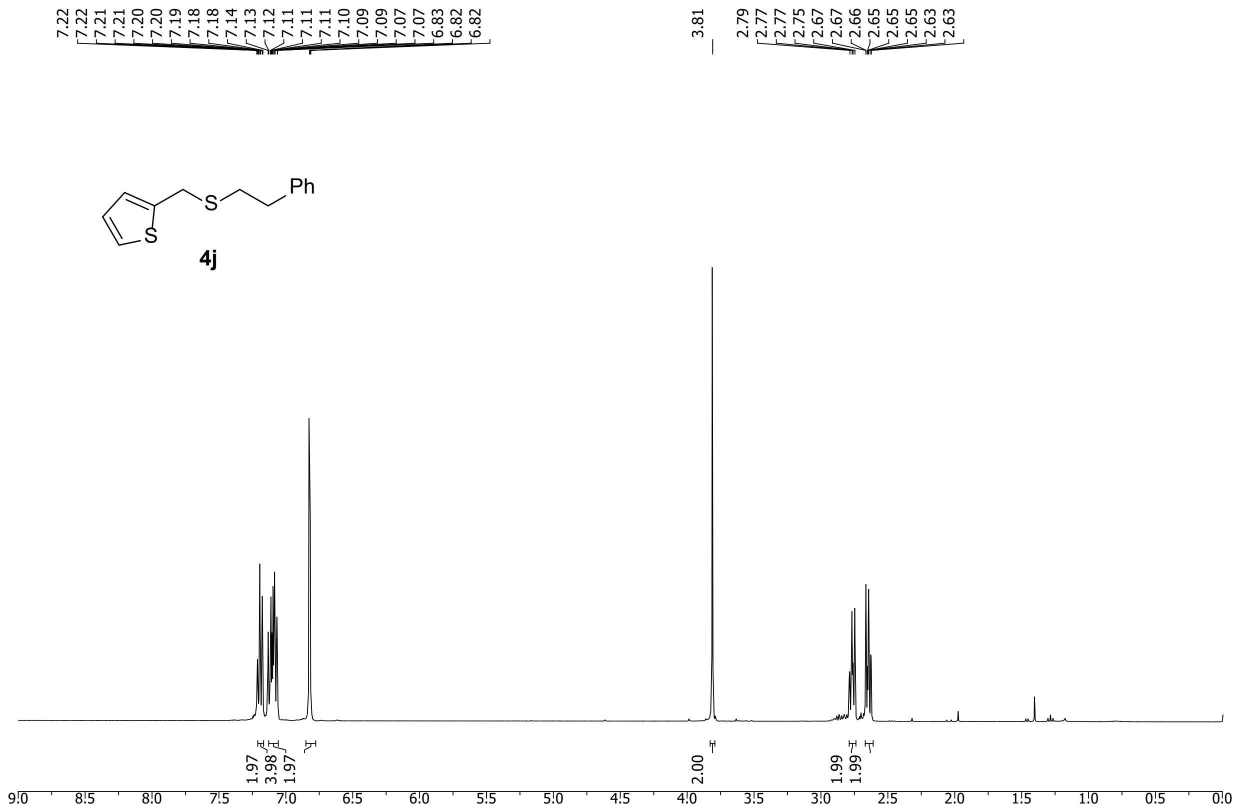
7.22 7.22 7.21 7.21 7.20 7.20 7.19 7.18 7.18 7.14 7.13 7.12 7.11 7.11 7.11 7.10 7.09 7.09 7.07 7.07 6.83 6.82 6.82
3.81
2.79 2.77 2.77 2.75 2.67 2.67 2.66 2.65 2.65 2.65 2.63 2.63
Ph
S
S
4j
1.97 3.98 1.97
2.00
1.99 1.99
9.0 8.5 8.0 7.5 7.0 6.5 6.0 5.5 5.0 4.5 4.0 3.5 3.0 2.5 2.0 1.5 1.0 0.5 0.0

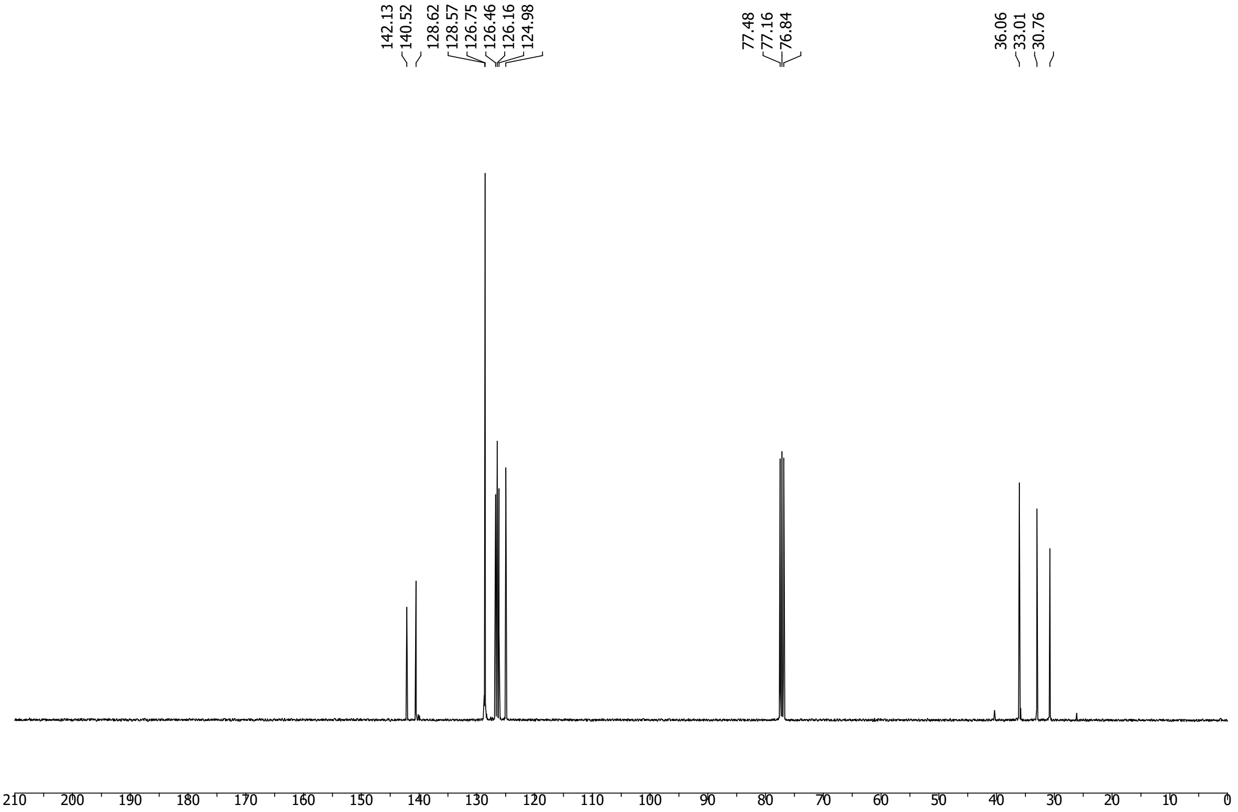
142.13 140.52 128.62 128.57 126.75 126.46 126.16 124.98
77.48 77.16 76.84
36.06 33.01 30.76
210 200 190 180 170 160 150 140 130 120 110 100 90 80 70 60 50 40 30 20 10 0

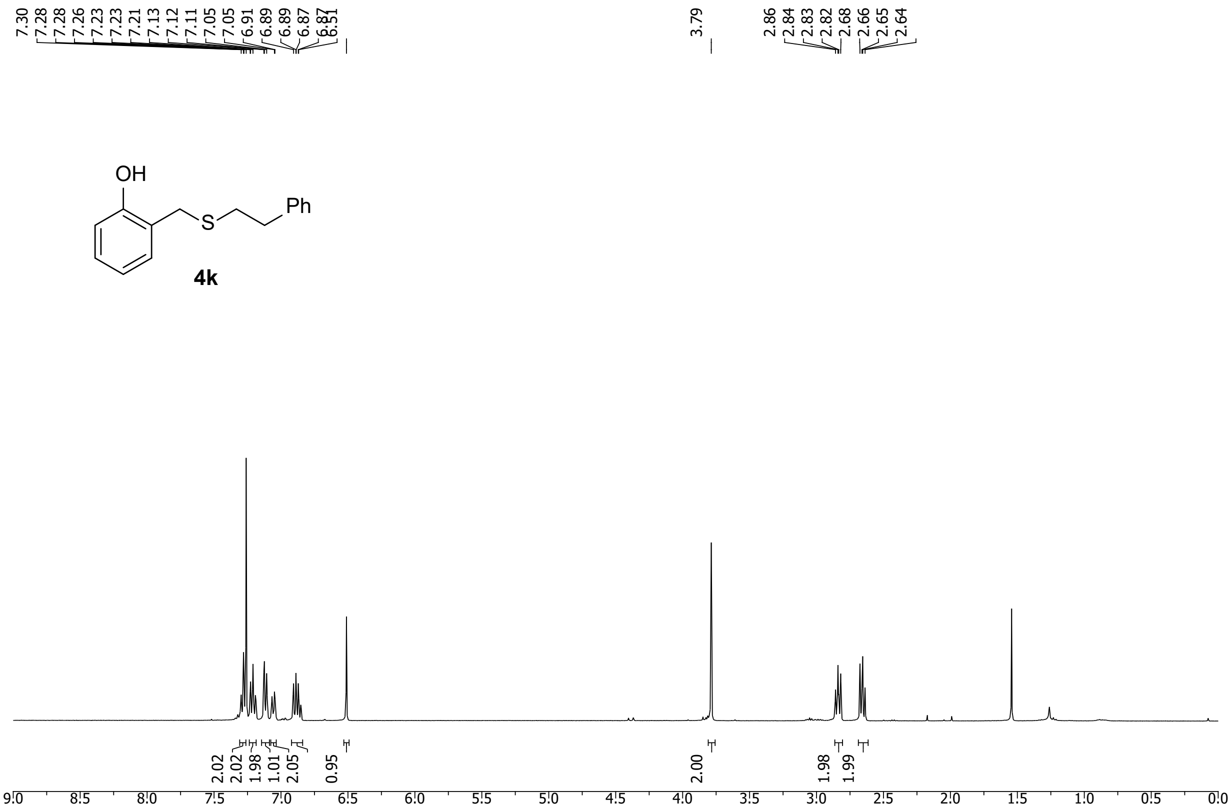
7.30 7.28 7.28 7.26 7.23 7.23 7.21 7.13 7.12 7.11 7.05 7.05 6.91 6.89 6.89 6.87 6.87 6.51
3.79
2.86 2.84 2.83 2.82 2.68 2.66 2.65 2.64
OH
S
Ph
4k
2.02 2.02 1.98 1.01 2.05 0.95 2.00 1.98 1.99
9.0 8.5 8.0 7.5 7.0 6.5 6.0 5.5 5.0 4.5 4.0 3.5 3.0 2.5 2.0 1.5 1.0 0.5 0.0

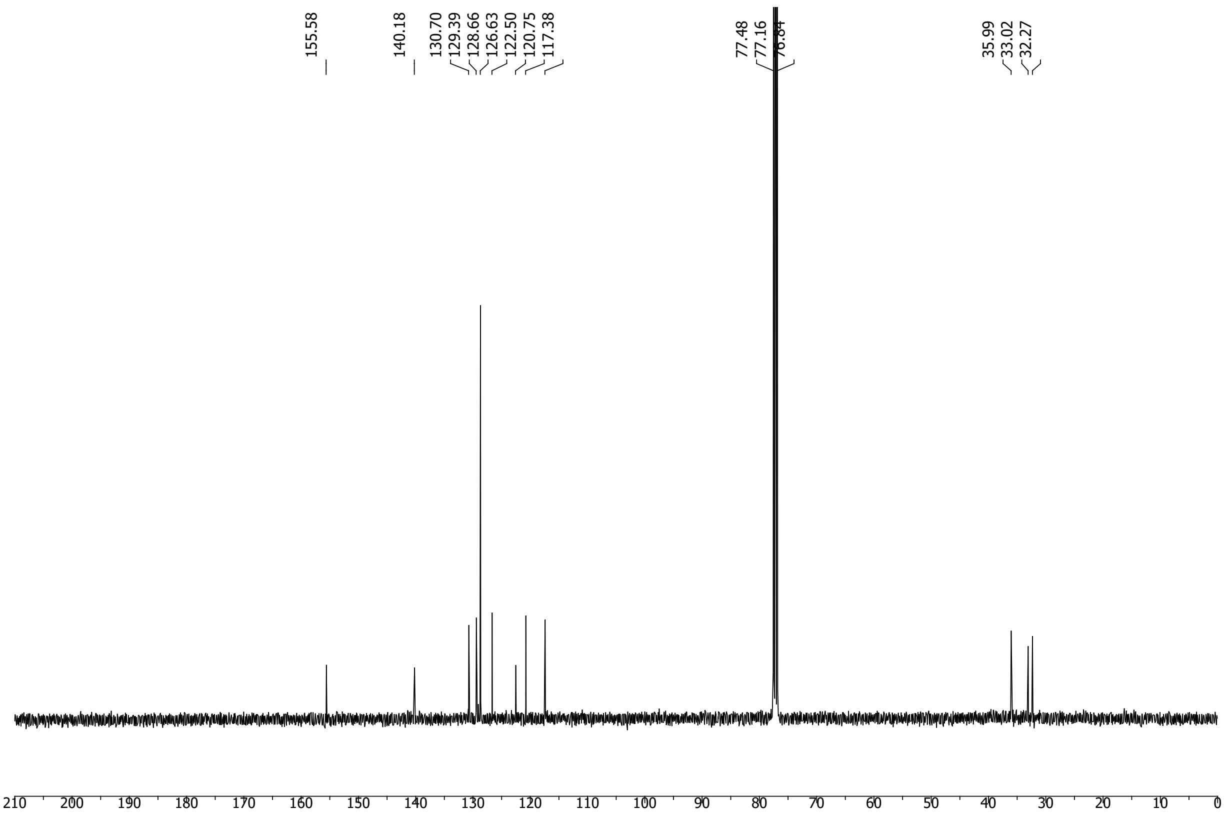
155.58
140.18
130.70 129.39 128.66 126.63 122.50 120.75 117.38
77.48 77.16 76.84
35.99 33.02 32.27
210 200 190 180 170 160 150 140 130 120 110 100 90 80 70 60 50 40 30 20 10 0

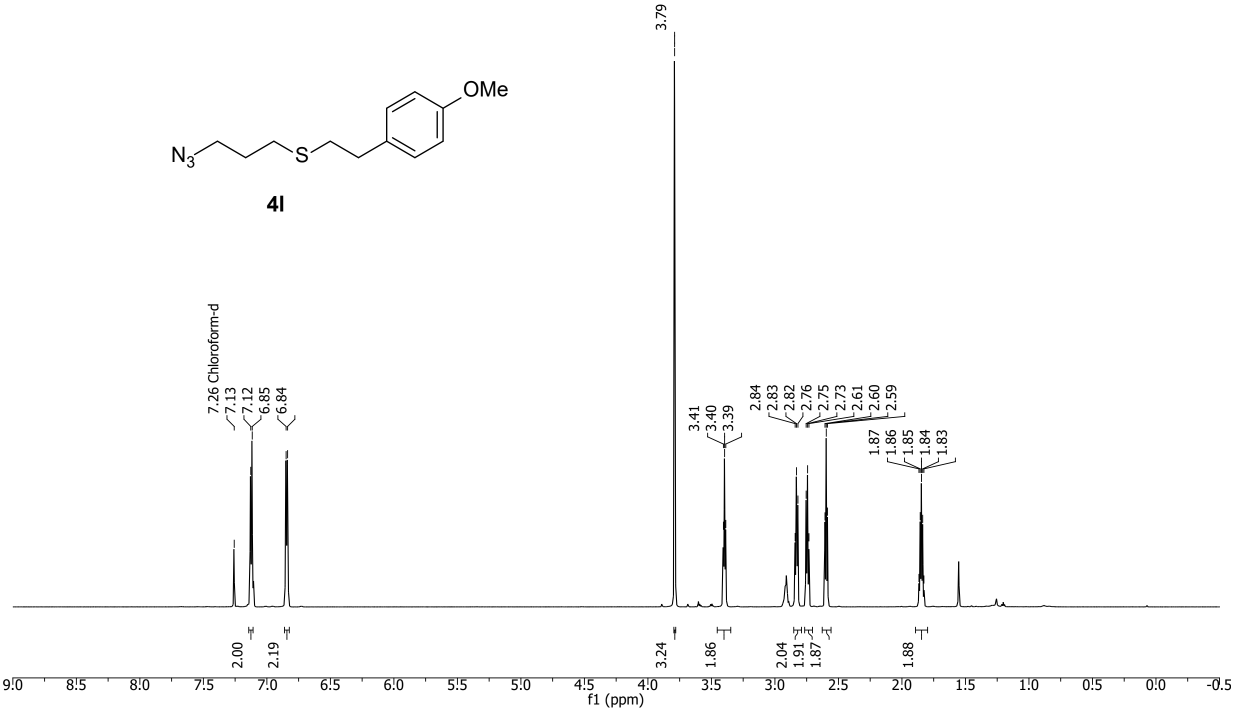
OMe
N3
S
4l
3.79
7.26 Chloroform-d
7.13
7.12
6.85
6.84
3.41
3.40
3.39
2.84
2.83
2.82
2.76
2.75
2.73
2.61
2.60
2.59
1.87
1.86
1.85
1.84
1.83
2.00
2.19
3.24
1.86
2.04
1.91
1.87
1.88
9.0 8.5 8.0 7.5 7.0 6.5 6.0 5.5 5.0 4.5 4.0 3.5 3.0 2.5 2.0 1.5 1.0 0.5 0.0 -0.5
f1 (ppm)

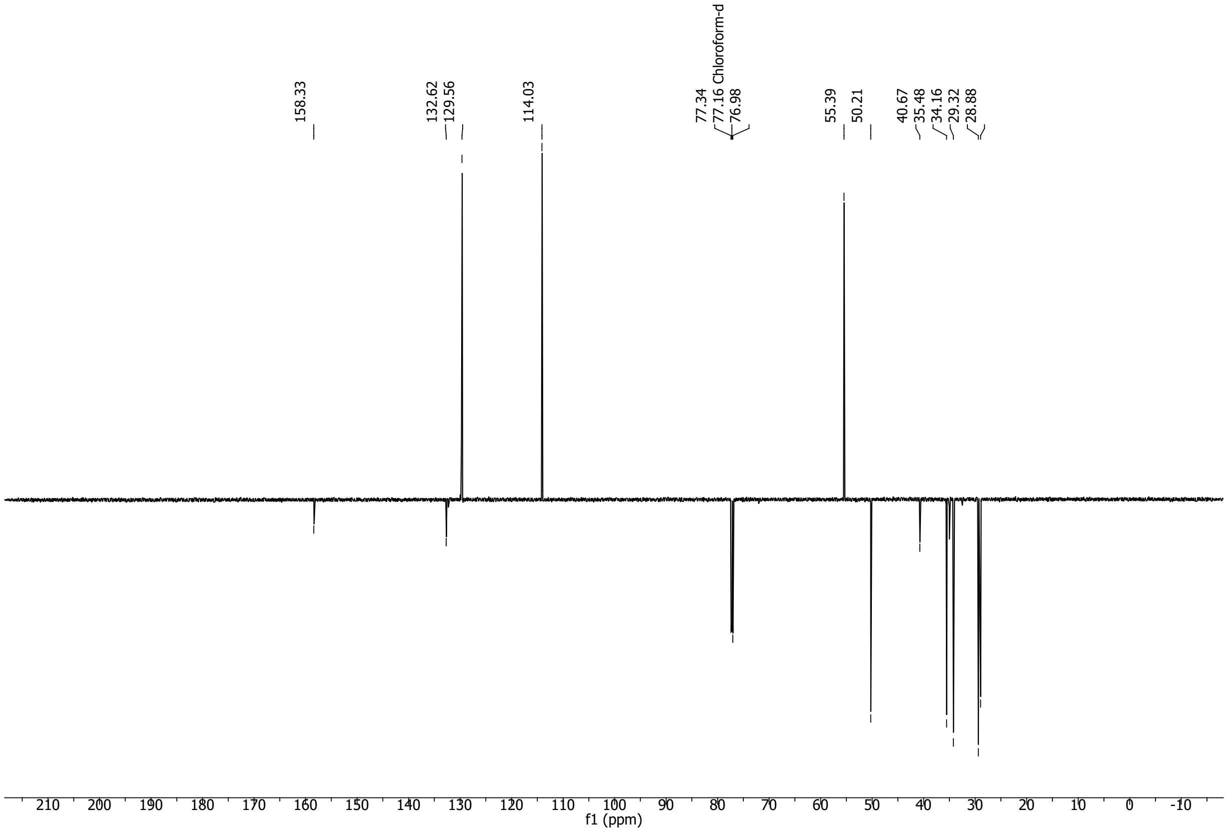
158.33
132.62
129.56
114.03
77.34
77.16 Chloroform-d
76.98
55.39
50.21
40.67
35.48
34.16
29.32
28.88
210 200 190 180 170 160 150 140 130 120 110 100 90 80 70 60 50 40 30 20 10 0 -10
f1 (ppm)

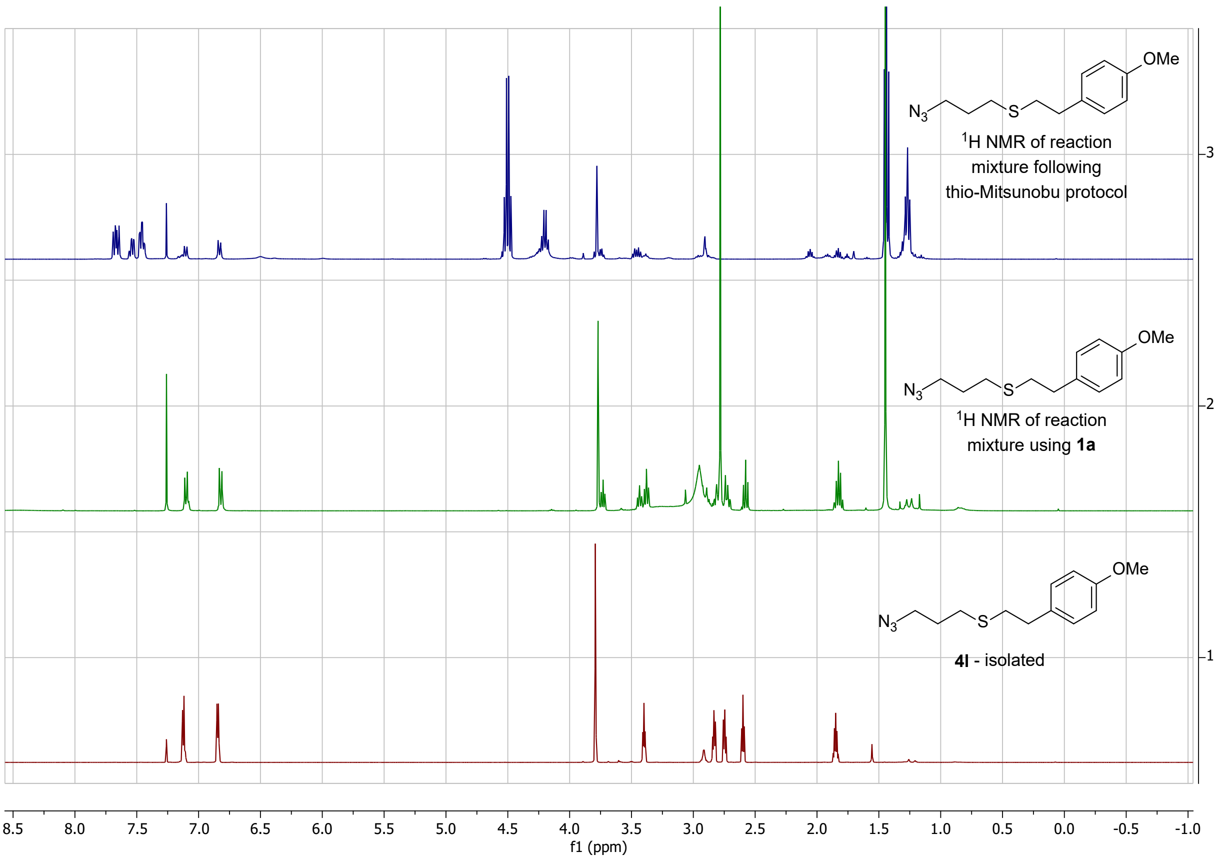

OMe
N3
S
1H NMR of reaction mixture following thio-Mitsunobu protocol
OMe
N3
S
1H NMR of reaction mixture using 1a
OMe
N3
S
4l - isolated
3
2
1
8.5 8.0 7.5 7.0 6.5 6.0 5.5 5.0 4.5 4.0 3.5 3.0 2.5 2.0 1.5 1.0 0.5 0.0 -0.5 -1.0
f1 (ppm)

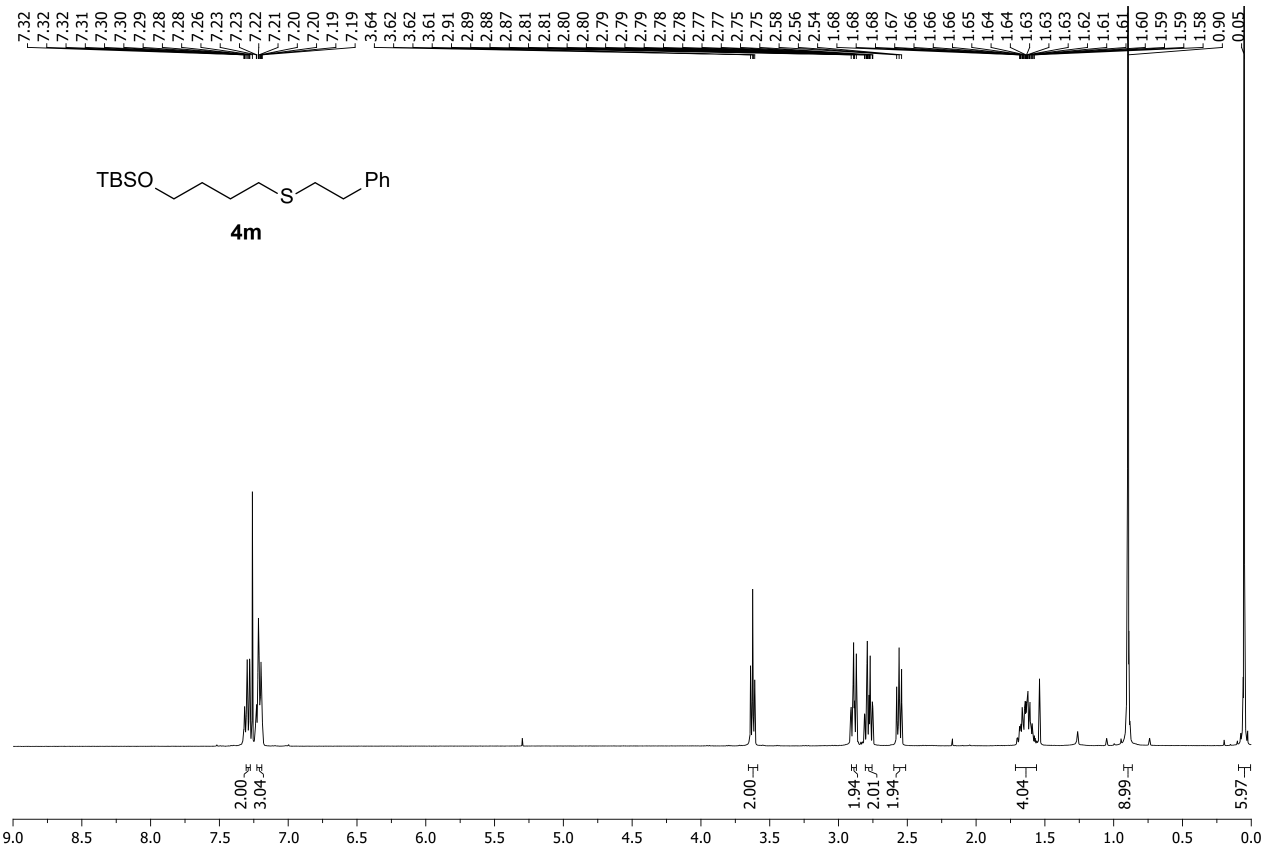
TBSO
S
Ph
4m
7.32 7.32 7.32 7.31 7.30 7.30 7.29 7.28 7.28 7.26 7.23 7.23 7.22 7.21 7.20 7.20 7.19 7.19
3.64 3.62 3.61 2.91 2.89 2.88 2.87 2.81 2.81 2.80 2.80 2.79 2.79 2.79 2.78 2.78 2.77 2.77 2.75 2.75 2.58 2.56 2.54
1.68 1.68 1.68 1.67 1.66 1.66 1.66 1.65 1.64 1.64 1.63 1.63 1.63 1.63 1.62 1.61 1.61 1.60 1.59 1.59 1.58 0.90 0.05
2.00 3.04 2.00 1.94 2.01 1.94 4.04 8.99 5.97
9.0 8.5 8.0 7.5 7.0 6.5 6.0 5.5 5.0 4.5 4.0 3.5 3.0 2.5 2.0 1.5 1.0 0.5 0.0

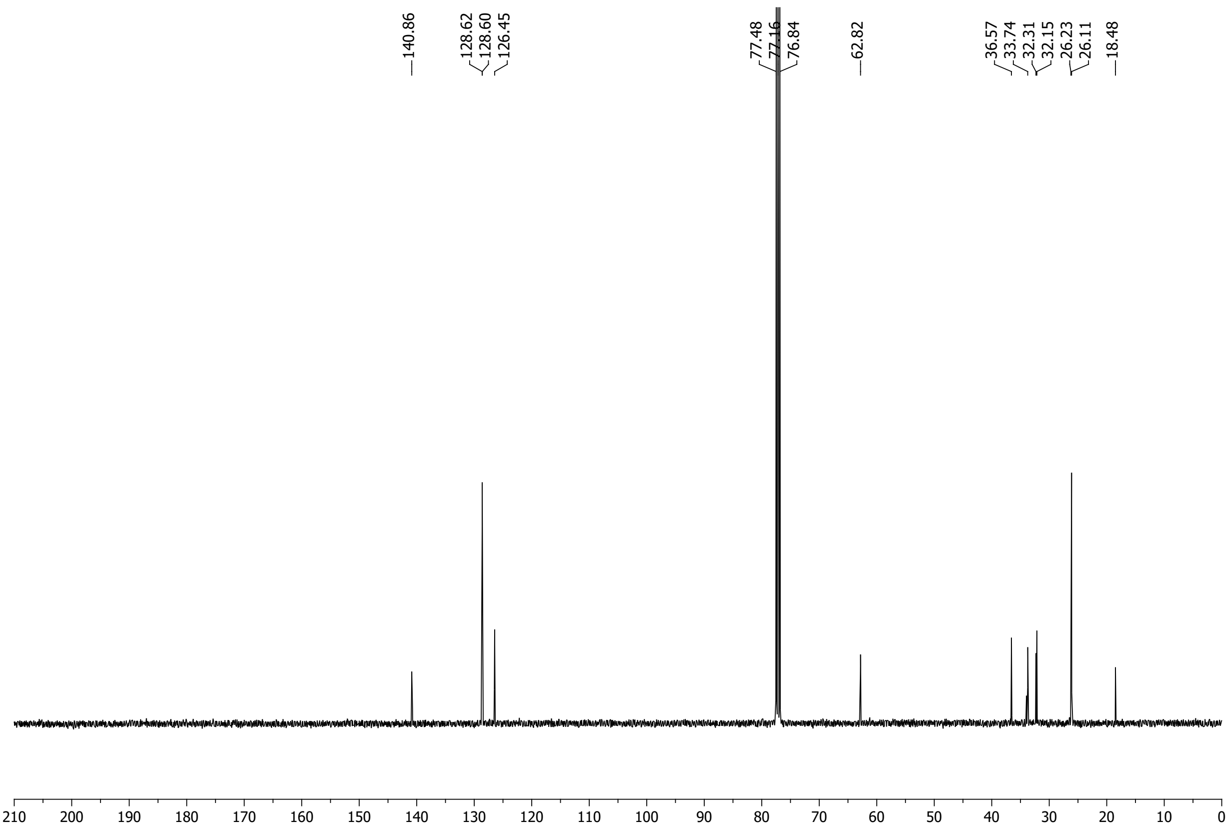
140.86
128.62 128.60 126.45
77.48 77.16 76.84
62.82
36.57 33.74 32.31 32.15 26.23 26.11 18.48
210 200 190 180 170 160 150 140 130 120 110 100 90 80 70 60 50 40 30 20 10 0

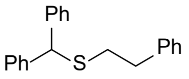
Ph
Ph
S
Ph

4n

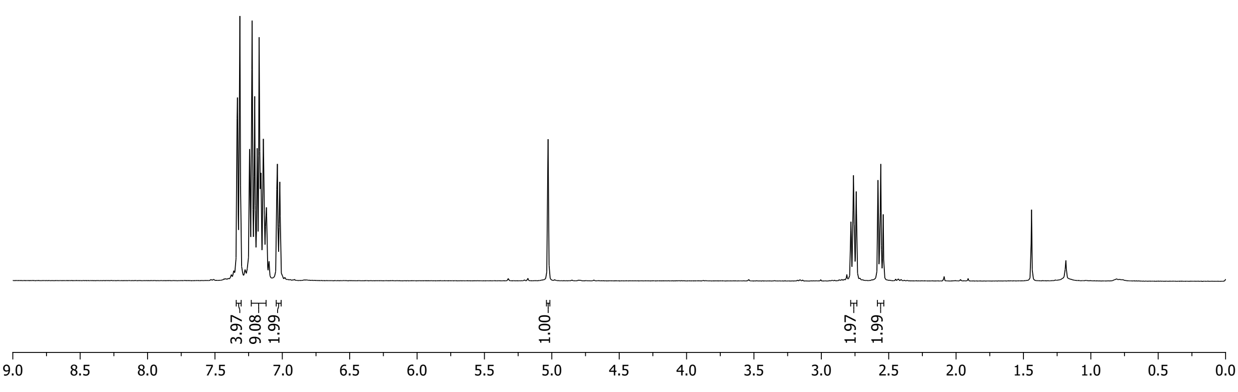
7.33 7.31 7.25 7.24 7.22 7.20 7.19 7.17 7.17 7.16 7.16 7.14 7.14 7.12 7.12 7.04 7.02
5.03
2.78 2.76 2.74 2.58 2.56 2.54
3.97 9.08 1.99
1.00
1.97 1.99
9.0 8.5 8.0 7.5 7.0 6.5 6.0 5.5 5.0 4.5 4.0 3.5 3.0 2.5 2.0 1.5 1.0 0.5 0.0

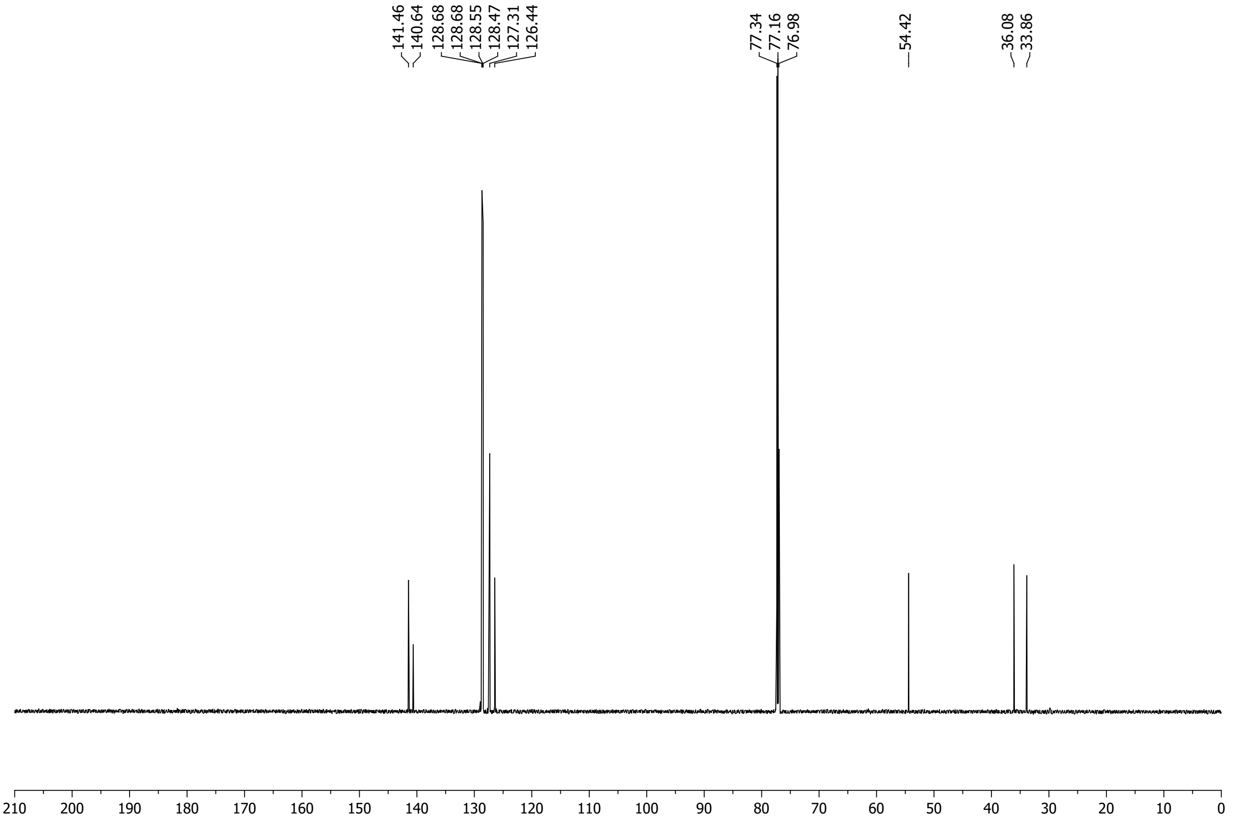
141.46 140.64 128.68 128.68 128.55 128.47 127.31 126.44
77.34 77.16 76.98
54.42
36.08 33.86
210 200 190 180 170 160 150 140 130 120 110 100 90 80 70 60 50 40 30 20 10 0

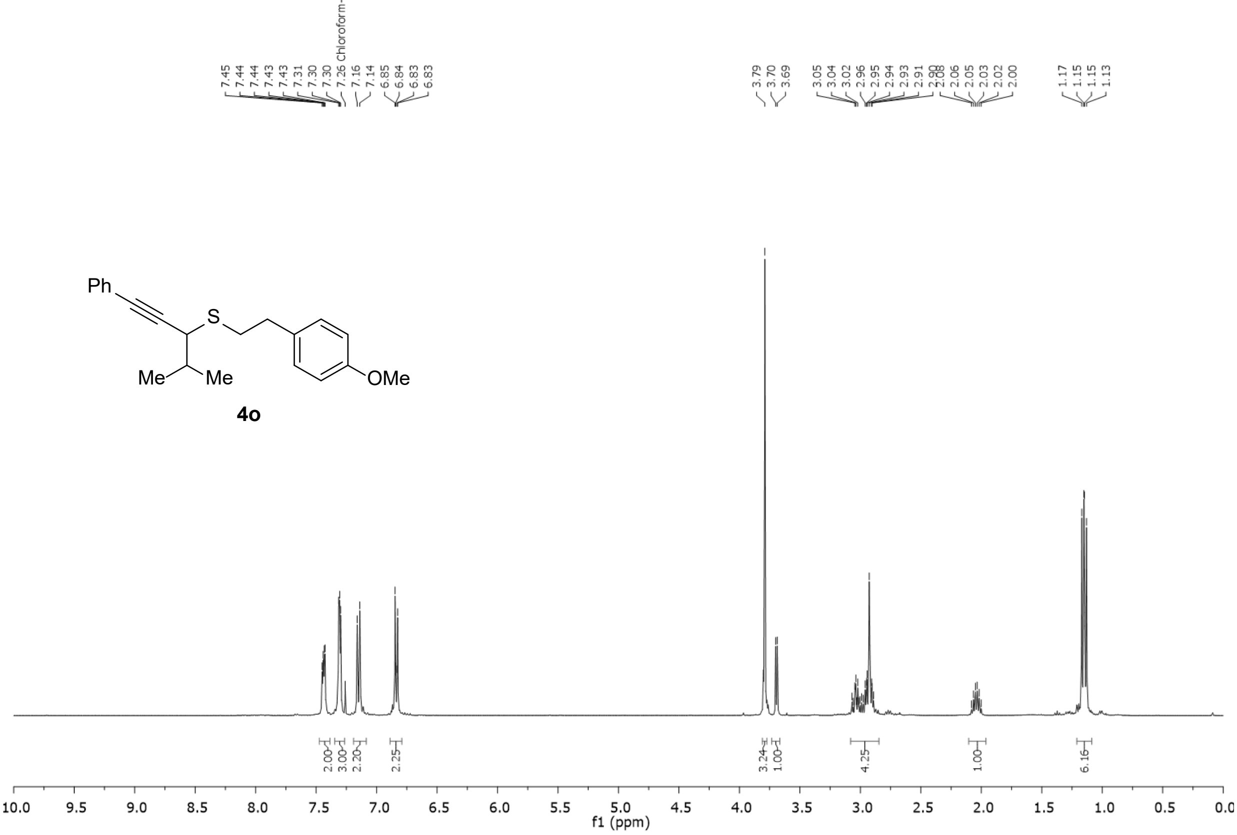
Ph
S
Me
Me
OMe
4o
7.45
7.44
7.44
7.43
7.43
7.31
7.30
7.30
7.26 Chloroform-d
7.16
7.14
6.85
6.84
6.83
6.83
3.79
3.70
3.69
3.05
3.04
3.02
2.96
2.95
2.94
2.93
2.91
2.90
2.08
2.06
2.05
2.03
2.02
2.00
1.17
1.15
1.15
1.13
2.00
3.00
2.20
2.25
3.24
1.00
4.25
1.00
6.16
10.0 9.5 9.0 8.5 8.0 7.5 7.0 6.5 6.0 5.5 5.0 4.5 4.0 3.5 3.0 2.5 2.0 1.5 1.0 0.5 0.0
f1 (ppm)

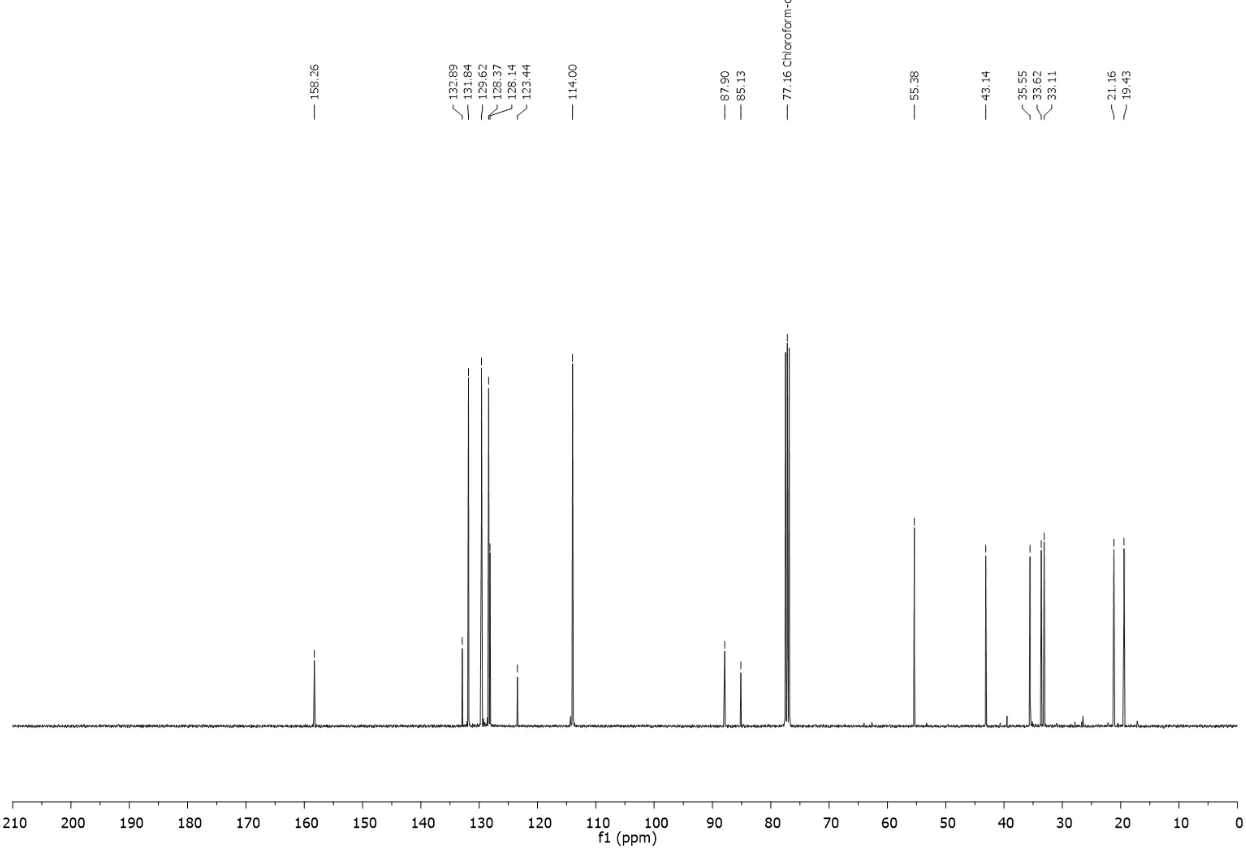
158.26
132.89
131.84
129.62
128.37
128.14
123.44
114.00
87.90
85.13
77.16 Chloroform-d
55.38
43.14
35.55
33.62
33.11
21.16
19.43
210 200 190 180 170 160 150 140 130 120 110 100 90 80 70 60 50 40 30 20 10 0
f1 (ppm)

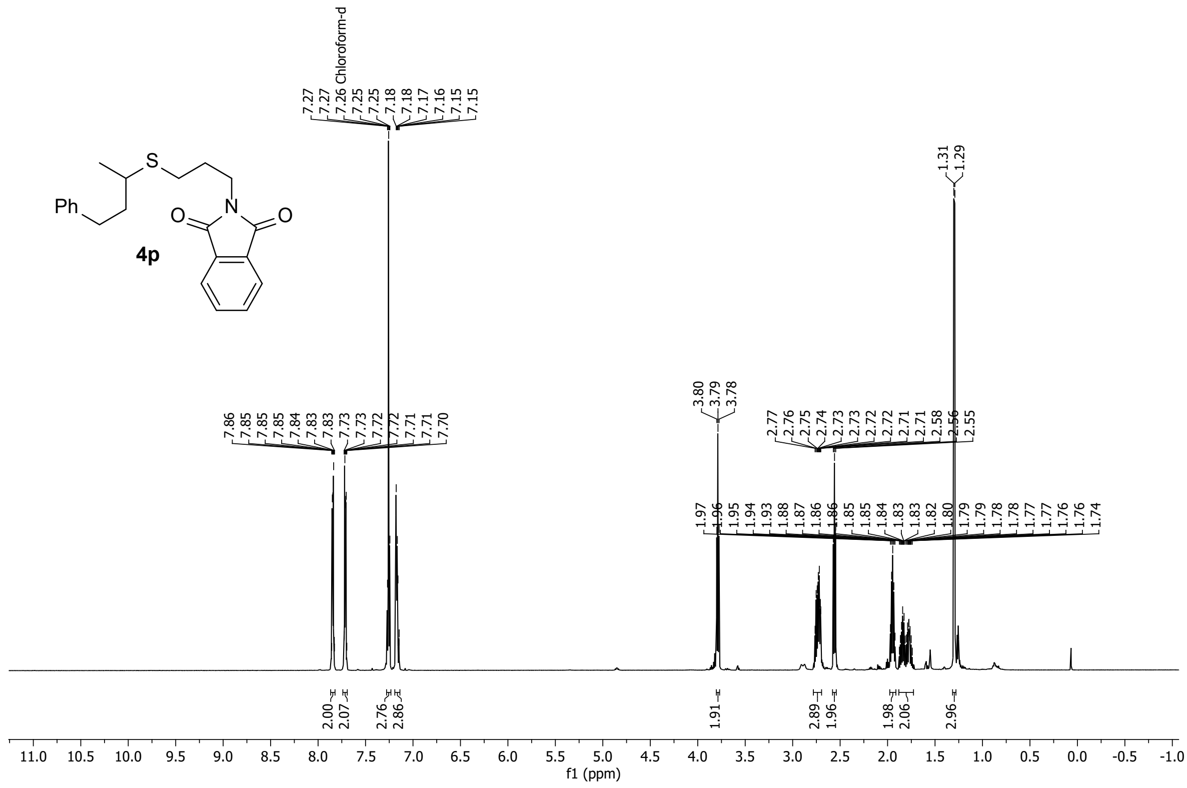
4p

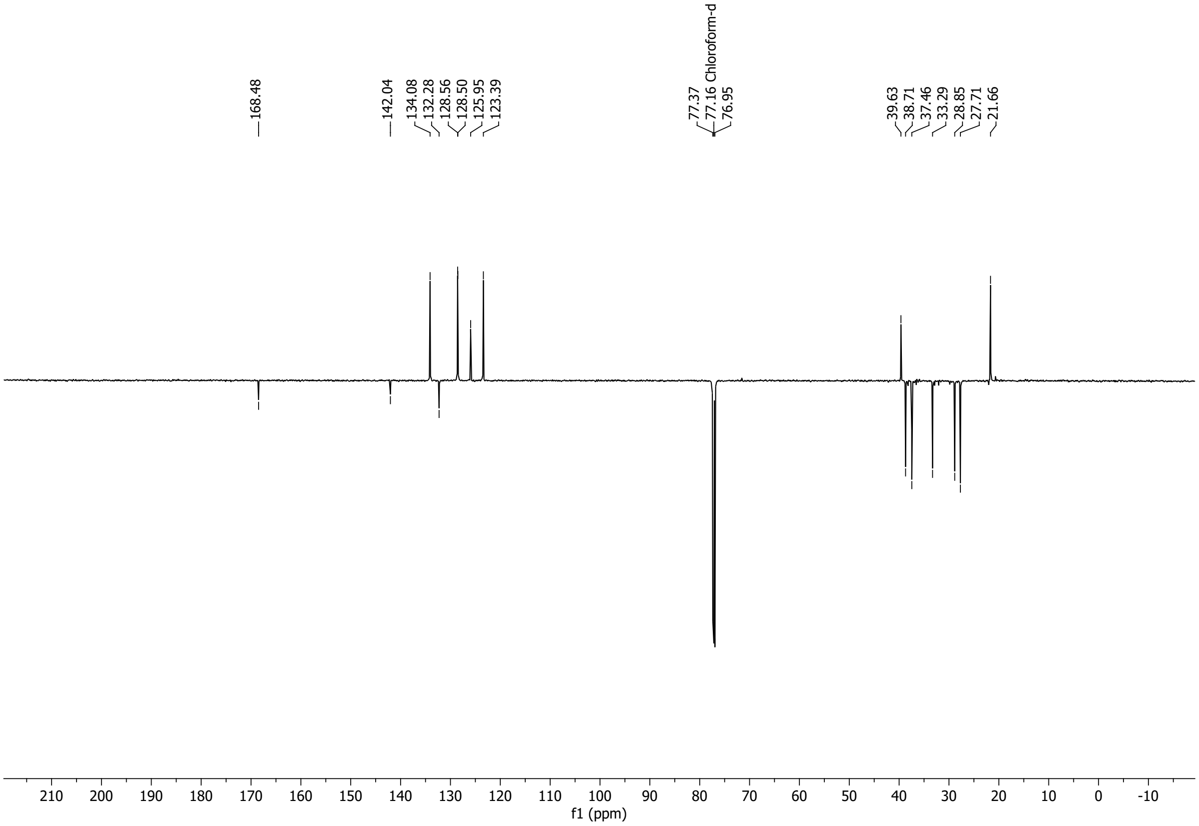

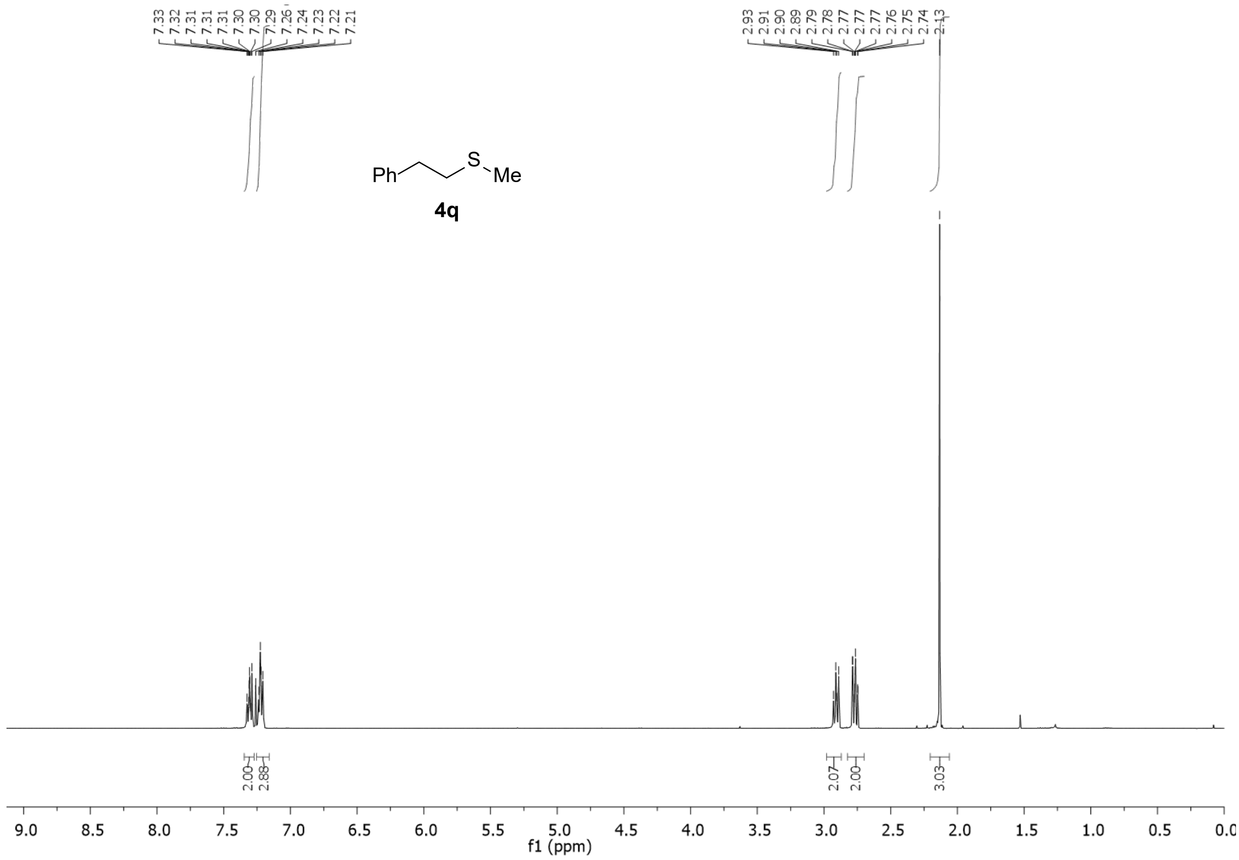

4q

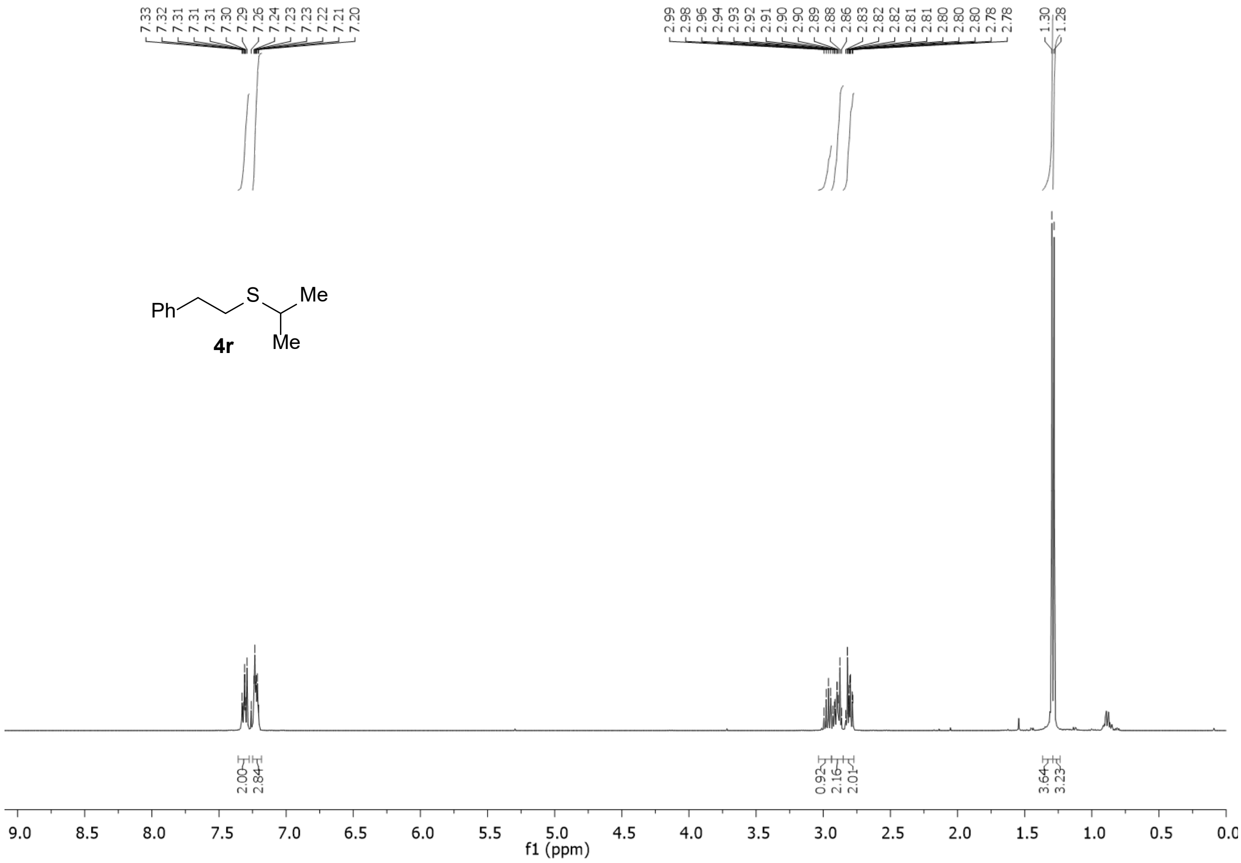

4r

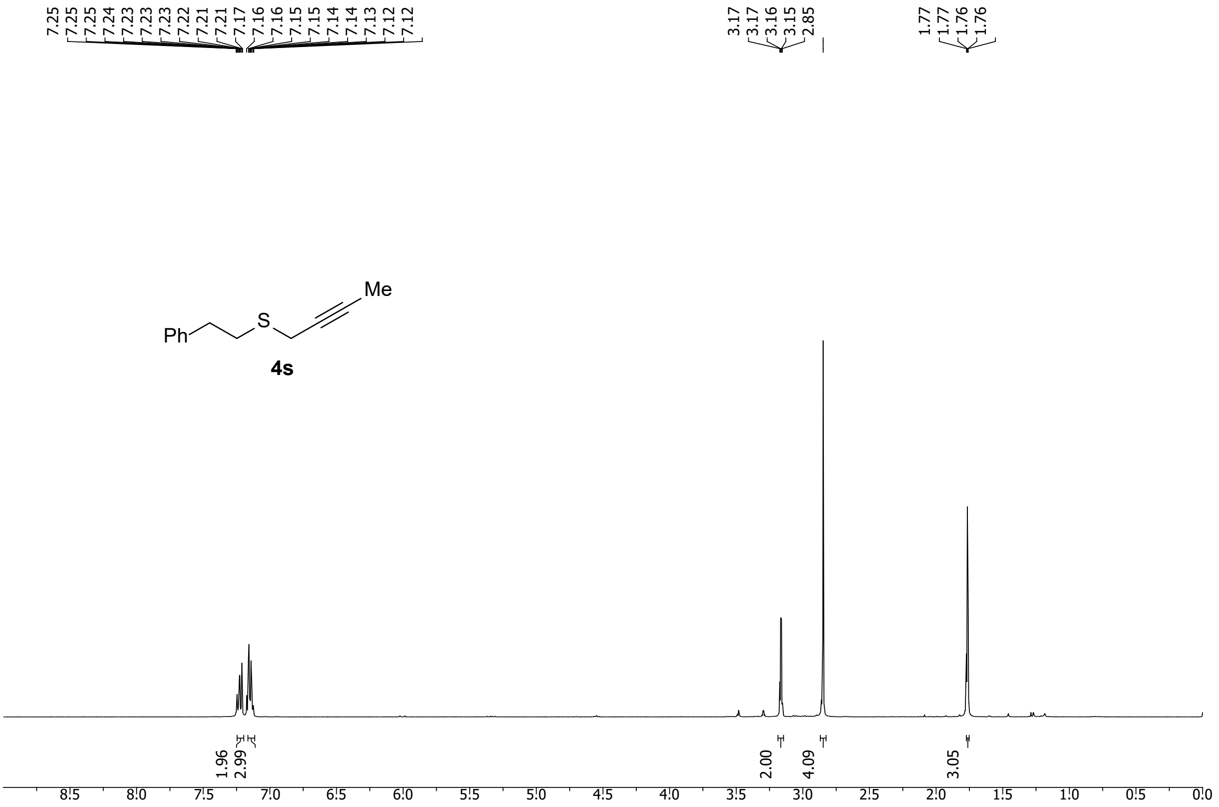

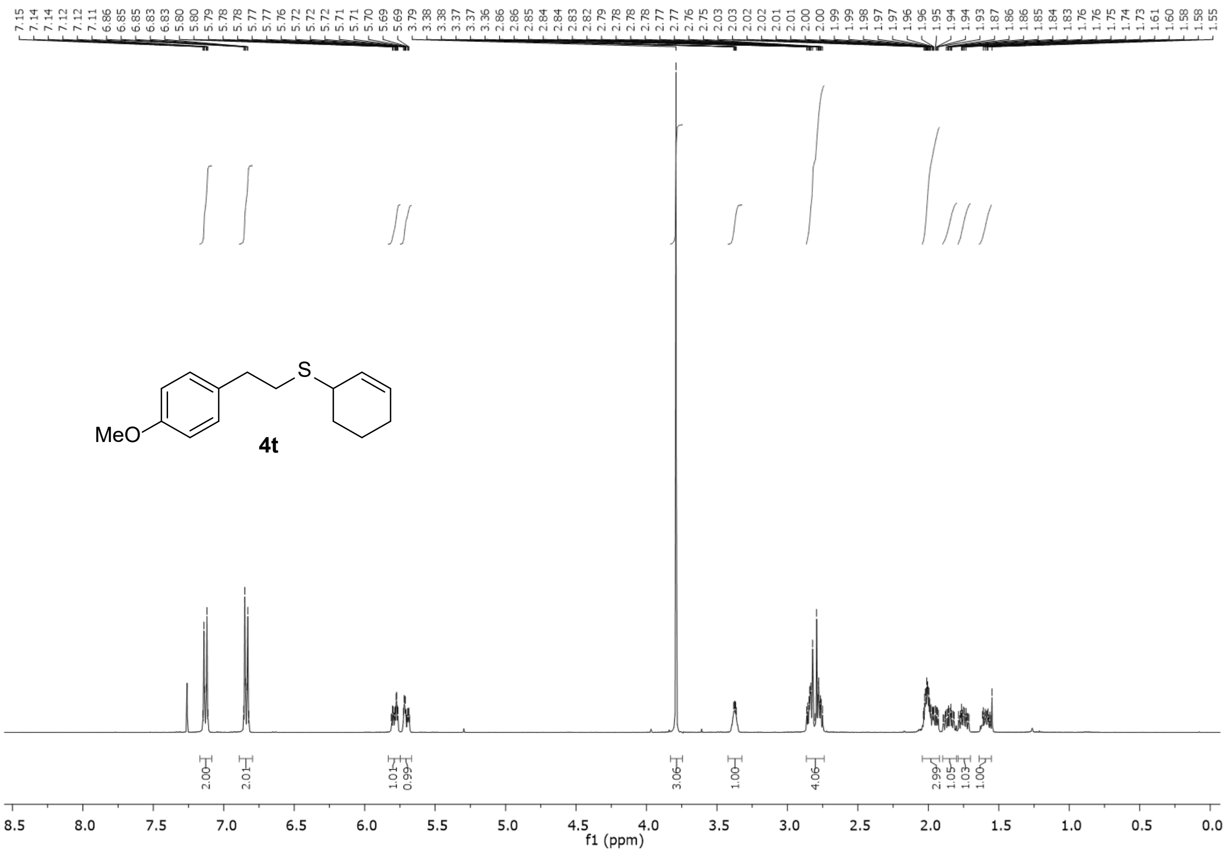
7.15 7.14 7.14 7.12 7.12 7.11 6.86 6.85 6.85 6.83 6.83 5.80 5.79 5.78 5.78 5.77 5.77 5.76 5.72 5.72 5.72 5.71 5.71 5.70 5.69 5.69 3.79 3.38 3.38 3.37 3.37 3.36 2.86 2.86 2.85 2.84 2.84 2.83 2.83 2.82 2.79 2.78 2.78 2.78 2.77 2.77 2.76 2.75 2.03 2.03 2.02 2.02 2.01 2.01 2.00 2.00 1.99 1.99 1.98 1.97 1.97 1.96 1.96 1.95 1.94 1.94 1.93 1.87 1.86 1.85 1.85 1.84 1.83 1.76 1.76 1.75 1.74 1.73 1.61 1.60 1.58 1.55
MeO
S
4t
2.00 2.01 1.01 0.99 3.06 1.00 4.06 2.99 1.05 1.03 1.00
f1 (ppm)

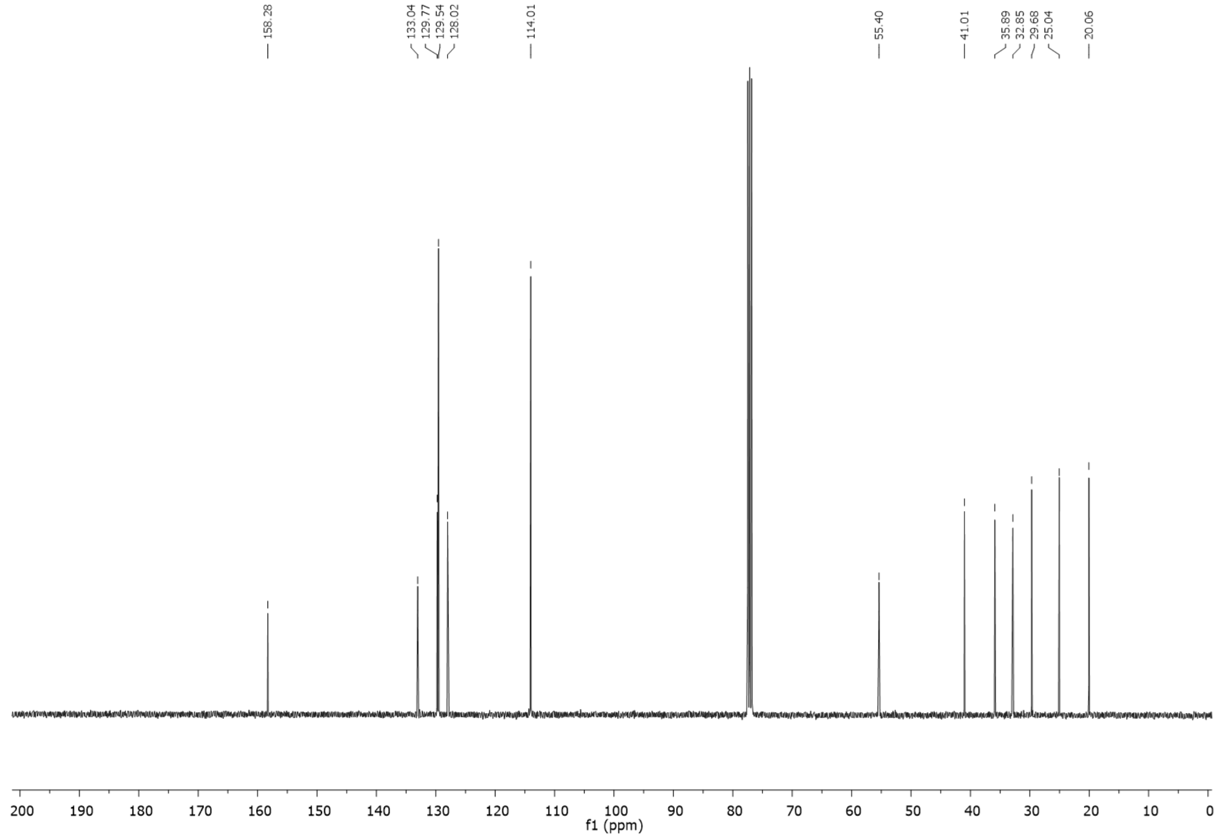
158.28
133.04 129.77 129.54 128.02
114.01
55.40
41.01
35.89 32.85 29.68 25.04
20.06
f1 (ppm)

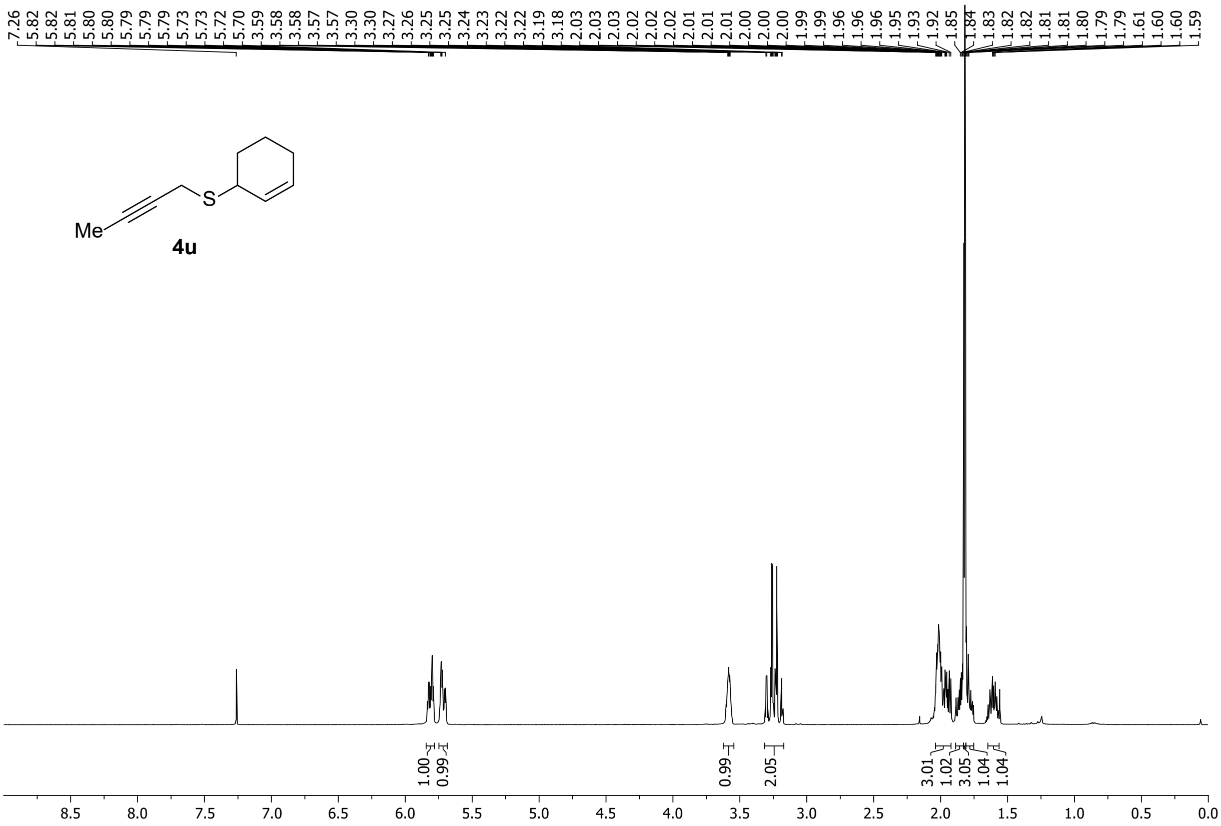
7.26 5.82 5.82 5.81 5.80 5.80 5.79 5.79 5.79 5.73 5.73 5.72 5.70 3.59 3.58 3.58 3.57 3.57 3.30 3.30 3.27 3.26 3.25 3.25 3.24 3.23 3.22 3.22 3.19 3.18 2.03 2.03 2.03 2.02 2.02 2.02 2.01 2.01 2.01 2.00 2.00 2.00 1.99 1.99 1.96 1.96 1.96 1.95 1.93 1.92 1.85 1.84 1.83 1.82 1.82 1.81 1.81 1.80 1.79 1.61 1.60 1.59
Me
S
4u
1.00
0.99
0.99
2.05
3.01
1.02
3.05
1.04
1.04
8.5 8.0 7.5 7.0 6.5 6.0 5.5 5.0 4.5 4.0 3.5 3.0 2.5 2.0 1.5 1.0 0.5 0.0

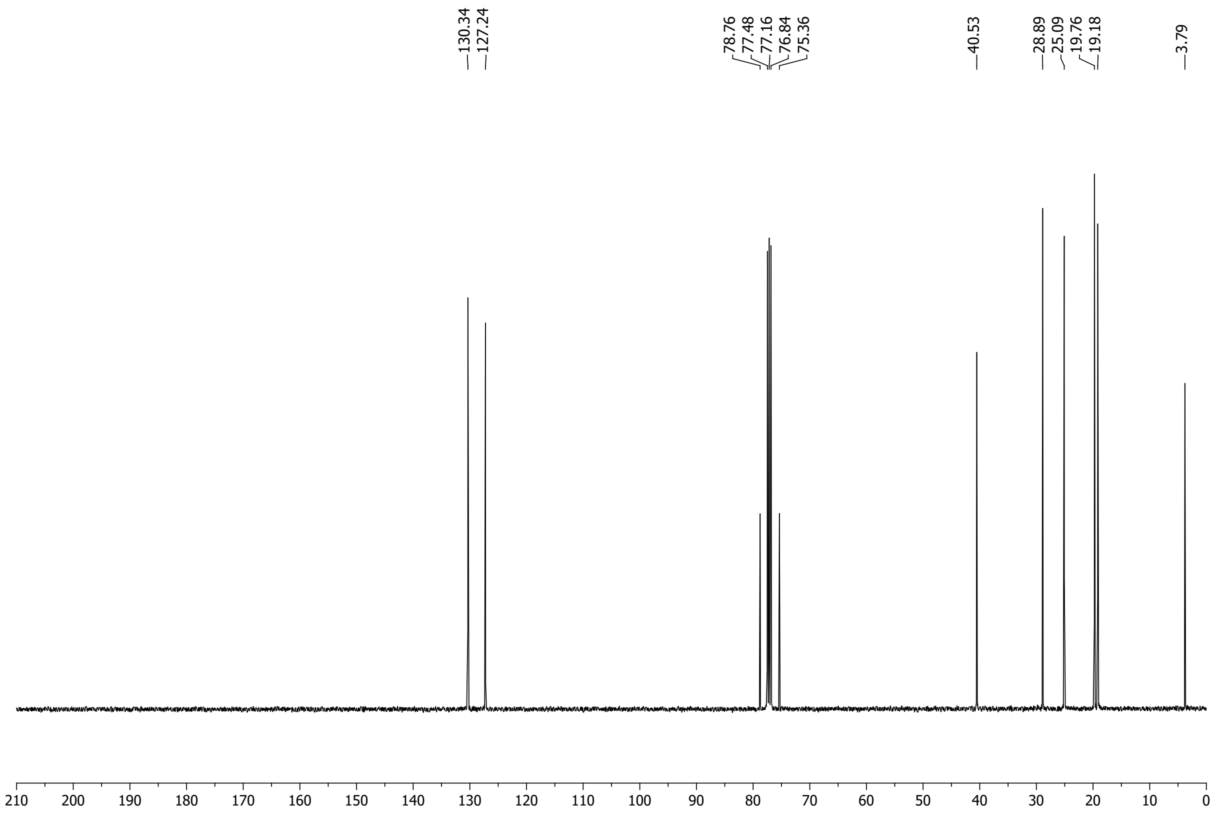
130.34
127.24
78.76
77.48
77.16
76.84
75.36
40.53
28.89
25.09
19.76
19.18
3.79
210 200 190 180 170 160 150 140 130 120 110 100 90 80 70 60 50 40 30 20 10 0

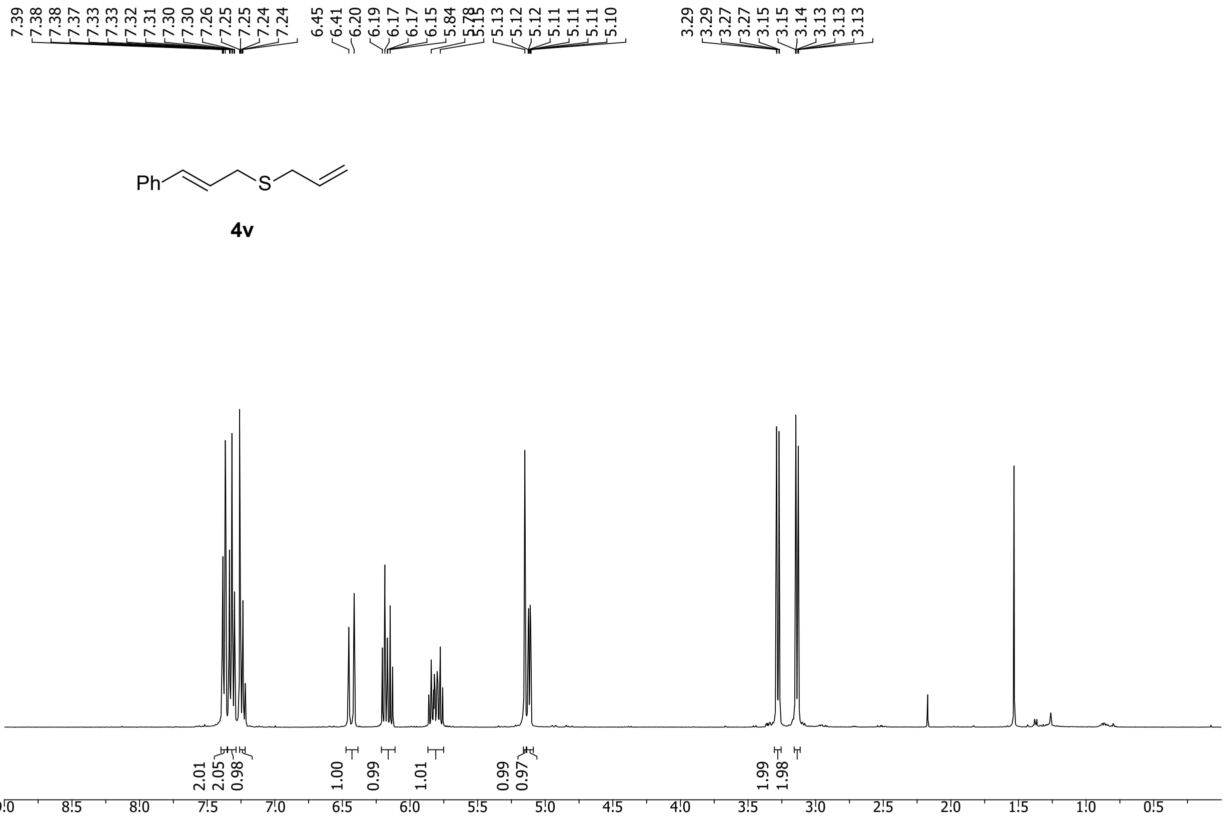
7.39 7.38 7.38 7.37 7.33 7.33 7.32 7.31 7.30 7.26 7.25 7.25 7.24 7.24
6.45 6.41 6.20 6.19 6.17 6.17 6.15 5.84 5.15 5.13 5.12 5.12 5.11 5.11 5.11 5.10
3.29 3.29 3.27 3.27 3.15 3.15 3.14 3.14 3.13 3.13 3.13
Ph
S
4v
2.01 2.05 0.98 1.00 0.99 1.01 0.99 0.97 1.99 1.98
9.0 8.5 8.0 7.5 7.0 6.5 6.0 5.5 5.0 4.5 4.0 3.5 3.0 2.5 2.0 1.5 1.0 0.5

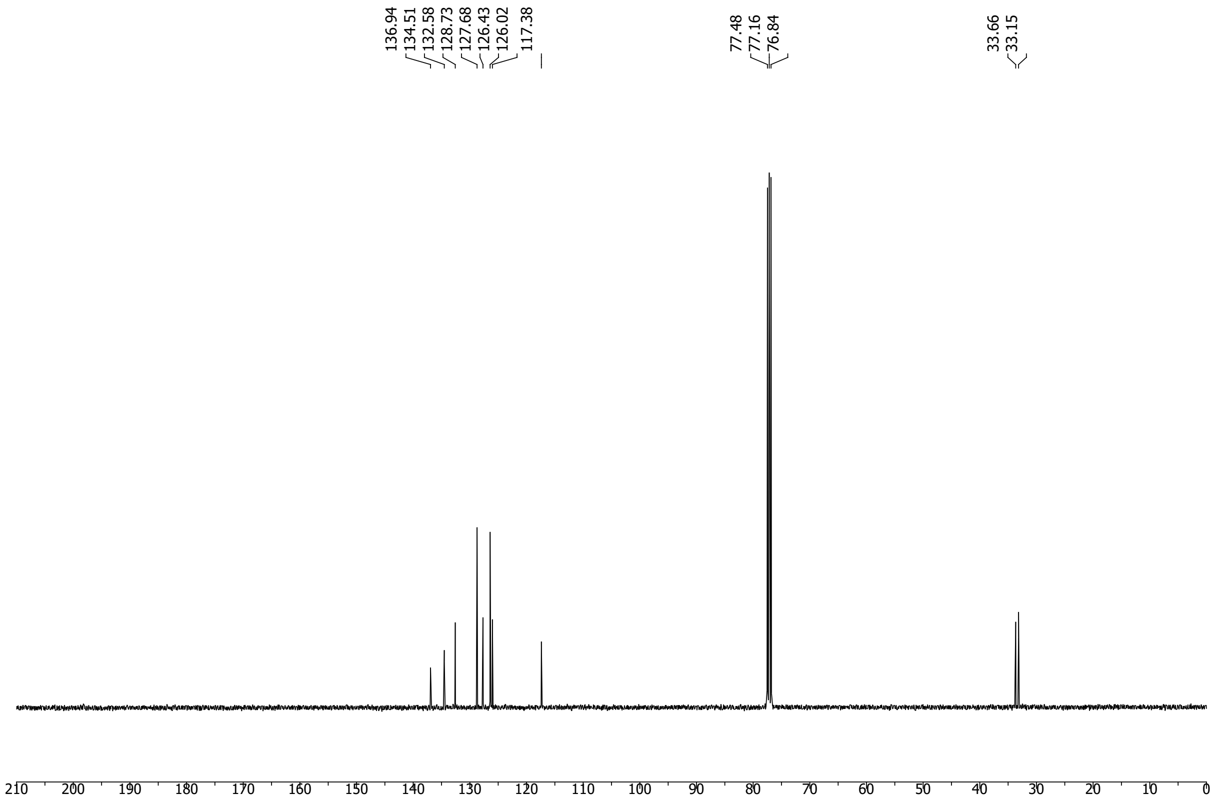
136.94 134.51 132.58 128.73 127.68 126.43 126.02 117.38
77.48 77.16 76.84
33.66 33.15
210 200 190 180 170 160 150 140 130 120 110 100 90 80 70 60 50 40 30 20 10 0

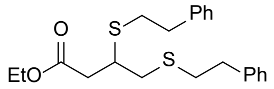
O
S
Ph
EtO
S
Ph

4w

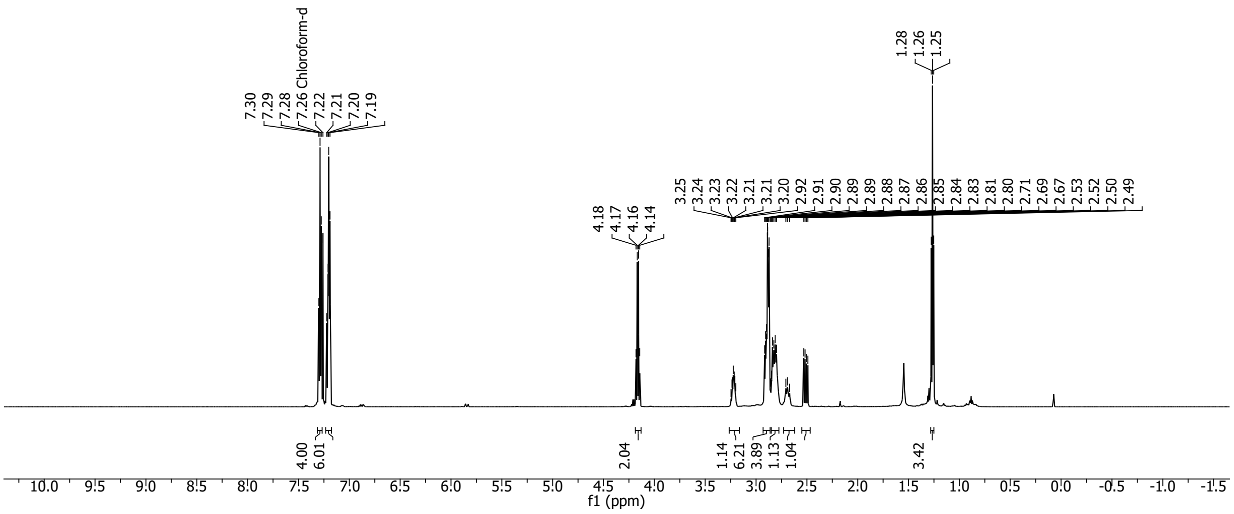
7.30 7.29 7.28 7.26 Chloroform-d 7.22 7.21 7.20 7.19
4.18 4.17 4.16 4.14
3.25 3.24 3.23 3.22 3.21 3.21 3.20 2.92 2.91 2.90 2.89 2.89 2.88 2.87 2.86 2.85 2.84 2.83 2.81 2.80 2.71 2.69 2.67 2.53 2.52 2.50 2.49
1.28 1.26 1.25
4.00 6.01 2.04 1.14 6.21 3.89 1.13 1.04 3.42
10.0 9.5 9.0 8.5 8.0 7.5 7.0 6.5 6.0 5.5 5.0 4.5 4.0 3.5 3.0 2.5 2.0 1.5 1.0 0.5 0.0 -0.5 -1.0 -1.5
f1 (ppm)

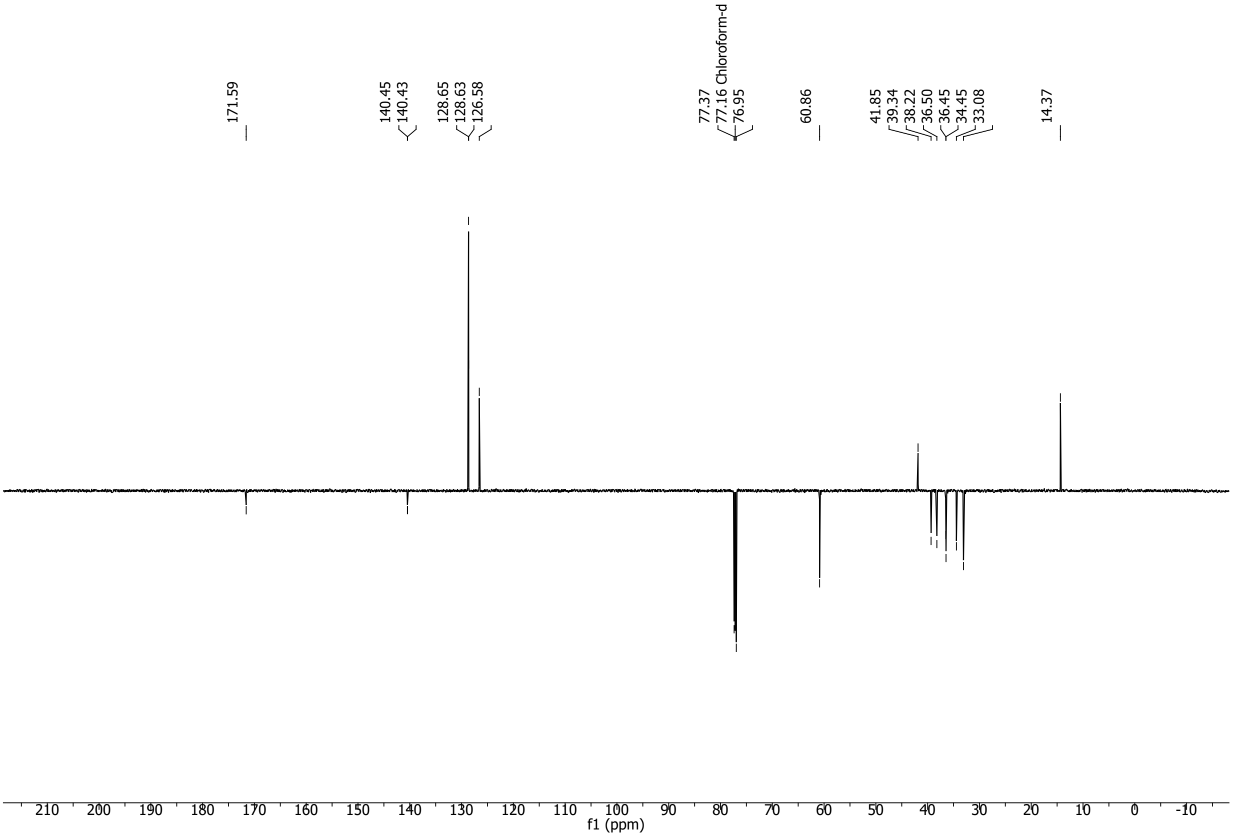
171.59
140.45 140.43
128.65 128.63 126.58
77.37 77.16 Chloroform-d 76.95
60.86
41.85 39.34 38.22 36.50 36.45 34.45 33.08
14.37
210 200 190 180 170 160 150 140 130 120 110 100 90 80 70 60 50 40 30 20 10 0 -10
f1 (ppm)

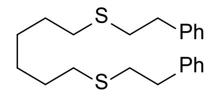

6

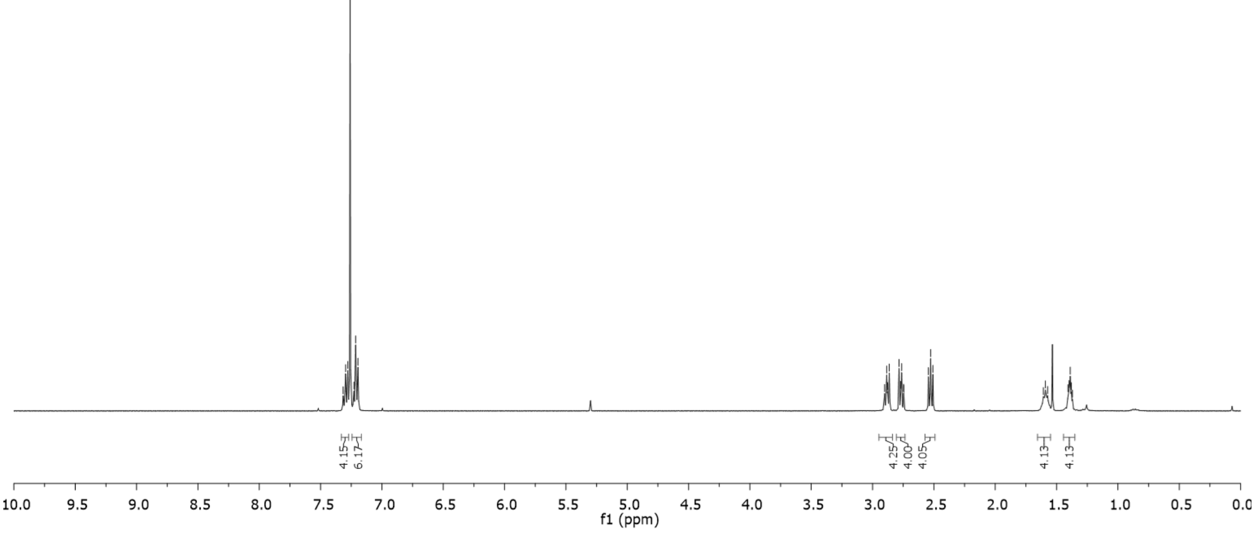
7.32
7.30
7.28
7.26 Chloroform-d
7.23
7.21
7.20
2.90
2.88
2.88
2.86
2.78
2.77
2.76
2.75
2.74
2.54
2.53
2.51
1.61
1.59
1.57
1.41
1.40
1.39
1.38
1.37
4.15
6.17
4.25
4.00
4.05
4.13
4.13
10.0 9.5 9.0 8.5 8.0 7.5 7.0 6.5 6.0 5.5 5.0 4.5 4.0 3.5 3.0 2.5 2.0 1.5 1.0 0.5 0.0
f1 (ppm)

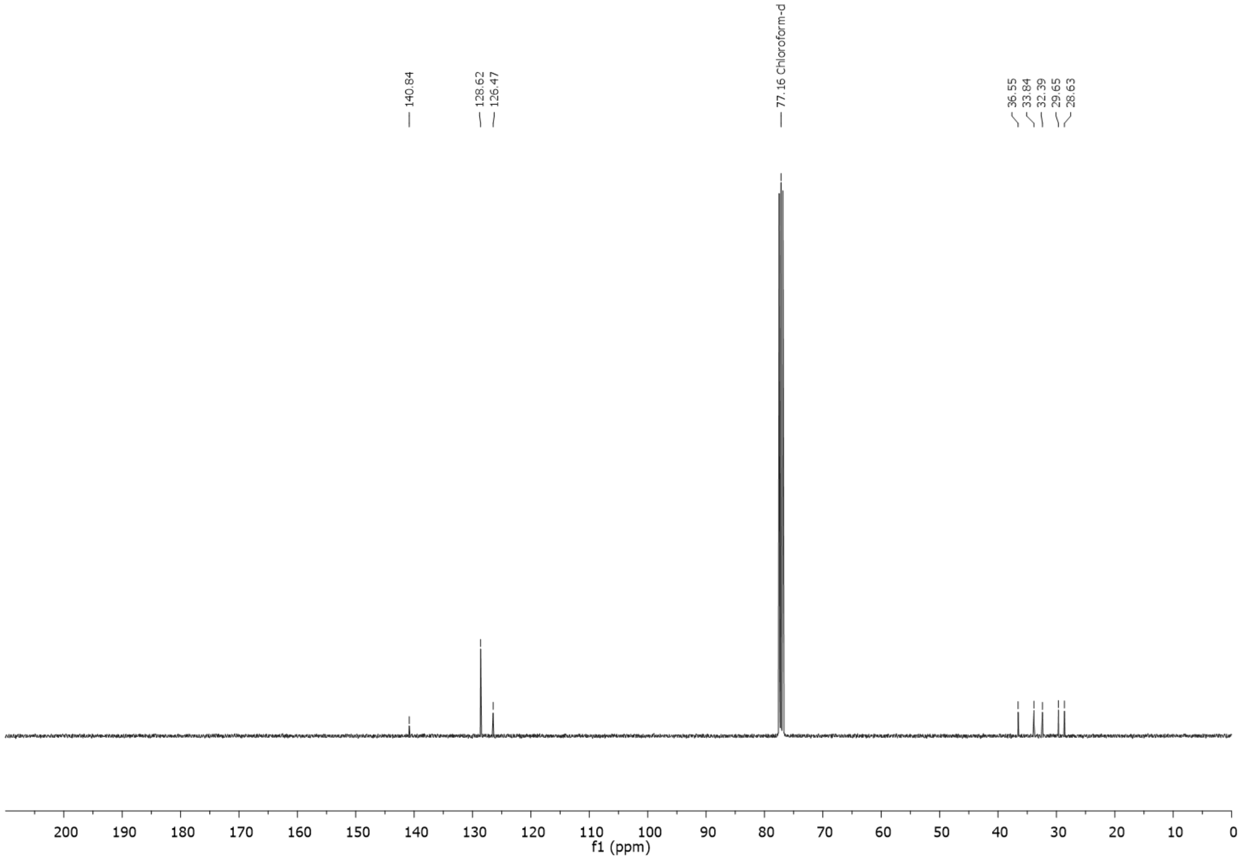
140.84
128.62
126.47
77.16 Chloroform-d
36.55
33.84
32.39
29.65
28.63
200 190 180 170 160 150 140 130 120 110 100 90 80 70 60 50 40 30 20 10 0
f1 (ppm)

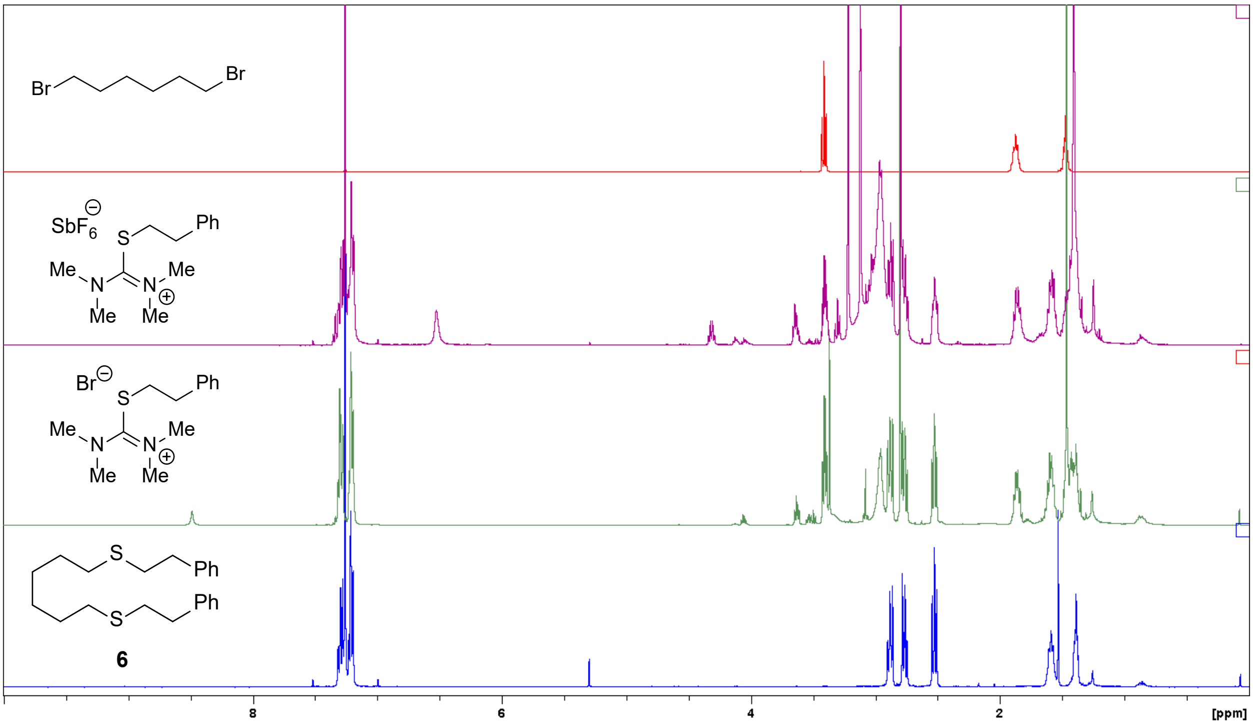

From top to bottom: a) ^{1}H-NMR of 1,6-dibromohexane, b) crude mixture from 6-bromo-1-hexanol and SbF_6^- salt, c) crude mixture from 6-bromo-1-hexanol and Br^- salt, d) double substitution product. Zoomed-in version below:

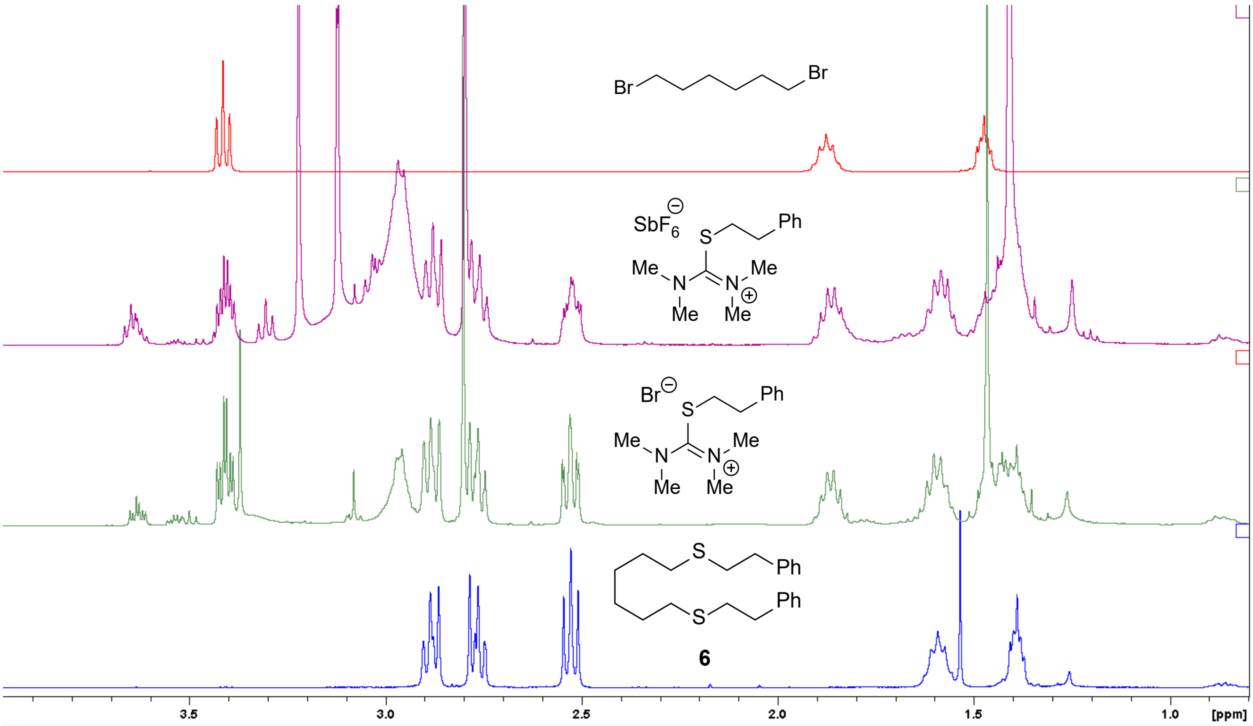

Supplement: SC-012-D1SC01602D-s001 [file SC-012-D1SC01602D-s001.pdf]
